# Genome-wide profiling of Populus small RNAs

**DOI:** 10.1186/1471-2164-10-620

**Published:** 2009-12-20

**Authors:** Daniel Klevebring, Nathaniel R Street, Noah Fahlgren, Kristin D Kasschau, James C Carrington, Joakim Lundeberg, Stefan Jansson

**Affiliations:** 1 School of Biotechnology, Division of Gene Technology, AlbaNova University Center, Royal Institute of Technology, 106 91 Stockholm, Sweden; 2 Umeå Plant Science Centre, Department of Plant Physiology, Umeå University, SE-901 87 Umeå, Sweden; 3 Center for Genome Research and Biocomputing, Oregon State University, Corvallis, Oregon 97331, USA

## Abstract

**Background:**

Short RNAs, and in particular microRNAs, are important regulators of gene expression both within defined regulatory pathways and at the epigenetic scale. We investigated the short RNA (sRNA) population (18-24 nt) of the transcriptome of green leaves from the sequenced *Populus trichocarpa *using a concatenation strategy in combination with 454 sequencing.

**Results:**

The most abundant size class of sRNAs were 24 nt. Long Terminal Repeats were particularly associated with 24 nt sRNAs. Additionally, some repetitive elements were associated with 22 nt sRNAs. We identified an sRNA hot-spot on chromosome 19, overlapping a region containing both the proposed sex-determining locus and a major cluster of *NBS-LRR *genes. A number of phased siRNA loci were identified, a subset of which are predicted to target PPR and *NBS-LRR *disease resistance genes, classes of genes that have been significantly expanded in *Populus*. Additional loci enriched for sRNA production were identified and characterised. We identified 15 novel predicted microRNAs (miRNAs), including miRNA*sequences, and identified a novel locus that may encode a dual miRNA or a miRNA and short interfering RNAs (siRNAs).

**Conclusions:**

The short RNA population of *P. trichocarpa *is at least as complex as that of *Arabidopsis thaliana*. We provide a first genome-wide view of short RNA production for *P. trichocarpa *and identify new, non-conserved miRNAs.

## Background

Plants produce a diverse and dynamic population of small RNAs (sRNAs) that are involved in transcriptional and post-transcriptional gene silencing and directing DNA methylation [1-4]. Distinct sub-populations of sRNAs have been identified and experimental evidence derived from *Arabidopsis thaliana *mutants has shown that each sub-population is derived via distinct biogenesis routes: microRNAs (miRNAs) are produced via the action of the Dicer-like RNAseIII-type ribonuclease DCL1 and are cleaved from precursor near-perfect stem-loop hairpins formed from RNA polymerase II transcripts; 21 nucleotide (nt) endogenous short-interfering RNAs (siRNAs) derive from long, double-stranded RNAs (dsRNA) and are produced via the action of an RNA-dependent RNA (RDR) polymerase; *trans*-acting siRNAs (TAS) are produced primarily by RDR6 together with SGS3 and DCL4, which yield phased 21 nt siRNAs; 24 nt heterochromatic siRNAs are produced by the action of the DNA-dependent RNA polymerase PolIV, RDR2 and DCL3 [1,5]. Work in *A. thaliana *has made extensive use of high throughput sequencing in combination with sRNA silencing mutants to elucidate the roles of genes within the different biogenesis pathways for sRNA classes [6].

The genus *Populus *is now firmly established as the model system for forest trees [7]. *Populus *represents an excellent model, being suitable for studies focusing on commercial traits, such as biomass-yield and wood fibre qualities, as well as association mapping and ecological interaction studies. As seasonal, hard-wood perennials with an extended juvenile phase, *Populus *species undergo a number of processes that could be expected to involve some degree of epigenetic control and re-programming. As such *Populus *represents an ideal system in which to further understanding of traits such as seasonal senescence, dormancy/growth-arrest and juvenile to adult phase transition. *Populus *is also nearly exclusively dioecious, yet currently the mechanism determining gender is unknown. An increasingly compelling body of evidence exists to suggest that chromosome 19 may be in the process of becoming a ZW style sex chromosome [8-11], with the female potentially being the heterogametic sex [9], although this is certainly not clear [8]. Again this is a trait that could involve an epigenetic component. As long-lived, clonally-replicating species, poplars and aspens also represent an excellent opportunity to identify how such long-lived species may have evolved particular means to survive both abiotic and biotic stresses. Particularly in the case of biotic factors, long-lived species face the challenge of surviving repeated attacks from antagonists with short generation times which are therefore capable of more rapid evolutionary change. Indeed the molecular clock of poplar ticks considerably more slowly than that of Arabidopsis thaliana [12].

*Populus *has so far not been exhaustively profiled for sRNAs, especially when compared to *A. thaliana *where there have now been a number of high-throughput sequencing studies performed [6,13-18] and where an excellent web resource exists for viewing the available datasets [19]. Previous work in *Populus *has either consisted of *in silico *studies or has been performed to a lower sequencing depth and has concentrated only on the identification of miRNAs [12,20-22]. To date, none have described the genomic distribution of other classes of sRNAs in *Populus*, despite those representing the majority of sRNAs produced.

We sequenced sRNAs in the sequenced *P. trichocarpa *genotype (Nisqually-1) using a concatenation approach in combination with massively parallel pyrosequencing (454). Using an established analysis pipeline that draws on the accumulated knowledge gained from analysing sRNA data in *A. thaliana *[23], we characterised sRNAs in young leaf material and in particular we concentrated on describing the genomic distribution and context of loci producing numerous sRNAs as well as identifying predicted *trans*-acting siRNA and miRNA loci.

## Results and Discussion

### Sequencing of sRNA using a concatenation strategy

**Figure 1:**
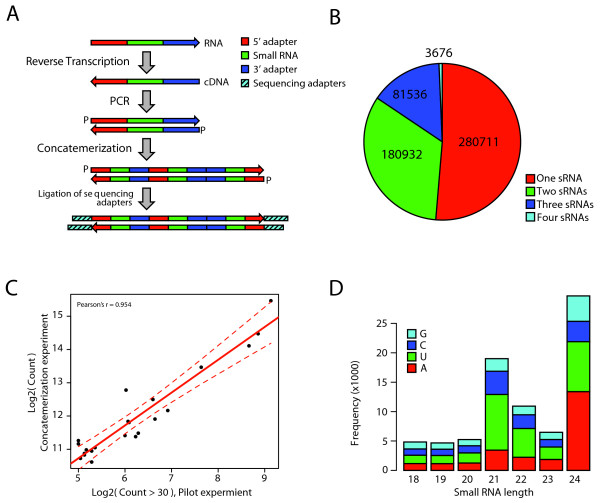
**Overview of sRNA sequencing approach**. **A **Schematic overview of our concatenation procedure. 5' and 3' adapter ligated RNA is reverse transcribed and used as template for PCR. The amplified product is concatenated by blunt ligation in a uncontrolled fashion in which ligation may take place in any direction. The strandedness of the small RNA can still be determined using the original 5' and 3' adapters. **B **Number of reads corresponding to 1-4 small RNA sequences. 901,887 small RNA reads were obtained from 546,855 sequencing reads, representing a 65% increase in throughput. **C **Comparison of expression values in the pilot experiment and concatenation experiment. The Pearson correlation coefficient is 0.954, indicating that very little bias is introduced by applying our concatenation strategy. **D **Frequency distribution of non-redundant sRNA reads with a raw sequence count *>*1. The number of sequences starting with an A (red), U (green), C (dark blue), and G (cyan) for each size class is indicated.

Massively parallel sequencing has provided a technological platform to investigate transcripts far more deeply than was previously possible. As sequencing read lengths increase, slightly modified methods can increase throughput several fold. Here we applied a concatenation strategy as outlined in Figure 1A. After cDNA synthesis and Polymerase Chain Reaction (PCR) amplification, a concatenation step was carried out. Concatenated amplicons were sequenced using 454 pyrosequencing [24]. After this step about half of the number of reads corresponded to single sRNA, 33% to two sRNAs, 15% to three sRNAs and only a minor fraction (≈ 0.7%) to four sRNAs (Figure 1B). In total, this strategy yielded 901,887 small RNA reads from 546,855 sequencing reads, representing a 65% increase in throughput. To investigate whether this approach changed measured sRNA expression levels, we also performed a small-scale experiment using a sub-region of a 454 picotiter plate. Comparison of measured raw read counts between the concatenated and non-concatenated libraries showed a Pearson's correlation coefficient of 0.954, indicating that no major changes were introduced using this approach (Figure 1C). With several platforms now reaching read lengths of 100 bases or more, concatenation of cDNA prior to sequencing is a valuable tool in small RNA sequencing experiments for increasing throughput. One limiting step in our protocol is the blunt concatenation of double-stranded cDNAs. It is possible that the ligation reaction can be improved by introducing a digestion step yielding fragments with protruding ends. Ligation of these fragments could be performed in a similar manner, but likely giving a better yield of longer fragments. Another step that can be introduced is gel purification to facilitate clean-up of fragments longer than, for example 150 base pairs, thereby increasing throughput.

Analysis of the sRNA sequence data was performed using tools from the UEA sRNA toolkit [23]. Three filtering steps (size range and complexity, t/rRNA matches, perfect genomic matches) were employed to extract a subset of sequences for further analysis. From the 901,887 resultant sRNA sequences, 363,619 (≈ 40%) passed all filters yielding 80,538 unique sequences with a perfect-match to the *P. trichocarpa *genome (Table 1). The largest loss of sequences occurred at the first filtering step (complexity and size-range) with many of these sequences likely representing partial degradation products of mRNAs. Filtering for low complexity is not expected to result in the loss of any miRNAs as all *A. thaliana *and *P. trichocarpa *miRNAs currently in miRBase (Release 13.0 [25]) contain at least three different bases. However, some *bona fide *siRNA may be removed, but these, if any, would represent a small minority. A significant percentage (≈ 15%) of sequences passing the first two filters did not have a perfect match to the published genome sequence: The current *Populus *genome sequence does not contain the centromeric regions, is a chimeric fusion of the two sequenced haplotypes and contains gaps. In particular, highly repetitive regions, which typically associate with sRNAs (see below), are under-represented in the assembly. As such it is not unexpected that some *bona fide *sequences would not have perfect matches within the current genome assembly. Future re-mapping of the dataset to updated genome assemblies may recover un-mapped reads. The majority of sequences with a read count above one were 24-mers and 21-mers (Figure 1D). The group of 21 nt sequences were over-enriched for sequences beginning with a Uracil (U) nucleotide when compared to all other size classes (Kolmogorov-Smirnov test p *<*0.000413). Although not significant, there was also a tendency for 24 nt sequences to start with an Adenine (A) nucleotide. These two findings are similar to those found in *A. thaliana *[16] and suggest that the same biogenesis mechanisms are operating in the two species, as is to be expected.

**Table 1:** Sequence reads summary.

	Total reads	Unique Reads
Input	901,887	
Low-complexity (*<*3 different bases), size-range (18-24 nt)	625,361	146,894
t/rRNA	502,277	129,750
Perfect match to Populus genome	363,619	80,538

Summary of redundant and non-redundant sequence counts after different filtering steps

### Comparison to previous Populus studies

There have been three publications relating to sRNAs in *Populus*, two of which used traditional Sanger sequencing [20,21] with the third using 454 pyrosequencing [22]. To allow direct comparison with our data we used the data from [22] (Additional files 1 and 2) to re-create a redundant dataset for analysis using the UEA plant sRNA toolkit. This resulted in 2,459 unique sequences between 18-24 nt after filtering (see Methods), of which 563 were perfect matches and 1846 were overlapping with the 80,538 unique sequence reads in our dataset.

One notable immediate difference between our dataset and that of [22] is the frequency distribution of different sRNA size classes. We found that 24 nt sRNAs were the most abundant size class, with 21 nt sRNAs also being highly abundant (Figure 1D). This is in agreement with previous reports in *A. thaliana *[6,16], maize [26] and *Physcomitrella patens *[27]. In contrast, the results presented by [22] showed clear dominance of 21 nt sRNAs with the second highest size class being 22 nts, followed by 24 nts. Re-analysis using the same filtering criteria as for our data again revealed a clear dominance of 21 nt sRNAs, however 24 nt sequences were the second highest class followed by 22 nt (combining data across leaves and vegetative buds). The reason for this difference may be that [22] used a *P. balsamifera *clone whereas we used the same genetic clone as was used to produce the genome sequence, and the unusually high sequence variation within or between *Populus *species complicates analysis [12].

### Genomic context of sRNA distribution

Since sRNAs often associate with repeats, it was of interest to analyse the distribution of sRNAs in relation to repeats in the *Populus *genome. However, as compared to Arabidopsis, repetitive element annotation in *Populus *is less well developed. We performed a RepeatModeler and RepeatMasker analysis of the *Populus *genome sequence. The results from this analysis are available at PopGenIE [28,29]. For RepeatModeler-identified repeats the majority of sRNAs did not overlap a repeat and only ten repeats had overlap to *>*100 unique sRNA with none having *>*500 overlapping sequences. The maximum number of overlapping sequences was 182 for a repeat that overlaps two NBS-LRR disease resistance gene models. The sRNAs in this region showed a clear enrichment for 21 mers. Within the RepeatMasker data there was clear dominance of 21 nt sRNAs for those repeats with *>*100 overlapping sRNAs. There were also a number of cases where there was dominance of 22 nt sRNAs or near-equal representation of 22 and 24 nt sRNAs with these being overlaps to LTR retrotransposons. For example a repeat on scaffold_132 (position 308756..310627) showed a majority of 22 nt sequences with substantial numbers of 24 nt sequences and few sequences from other size classes. Such examples are potentially interesting considering the findings of [26] where it was found that there is greater overlap of 22 nt sRNAs to LTR elements in maize than in Arabidopsis. Future work using biogenesis mutants will be needed to clarify whether 22 nt LTR-associated sRNAs in *Populus *are more similar to the maize or the Arabidopsis genomes.

In all other repeats there was clear dominance of 24 nt sRNAs with 21 nt sRNAs also having a significant representation. There were also substantial numbers of 22 nt sRNAs while other size classes were insignificant. In general, the RepeatMasker data matched the results that have been reported in *A. thaliana *[6,16].

RepeatMasker was used to identify all *Populus *and Rosid repeats in RepBase [30] (release 14.03). RepBase contains 169 lineage specific *Populus *repeats and 1018 Rosid repeats (of which 516 are Arabidopsis specific). There are additionally 176 ancestral/ubiquitous repeats. There were extremely few examples of overlap to repeats within the Rosids dataset with only 212 sRNAs overlapping currently annotated RepBase repeats. Three of these overlapped to a Medicago LINE element and the remainder overlapped with eight annotated LTR elements, five of which were *Populus *specific. The contrast between these overlap results and those of the RepeatModeler data strongly suggest that there is currently a paucity of public data relating to the repetitive elements in the *Populus *genome.

To obtain a genomic overview of loci producing siRNAs we used the UEA plant sRNA toolkit siLoCo tool to identify loci producing significant numbers of siRNAs (Additional File 1). This analysis identified many of the predicted phased loci (Table 2) and predicted and known miRNAs. As well as identifying siRNA clusters within the entire dataset, we used the siLoCo tool using only the 21 nt and 24 nt data subsets separately (Additional File 2 and 3 respectively). In both cases we examined subsets of identified loci with the highest number of unique hits and the highest raw read count values. The nature of the loci identified was distinctly different between the two size classes: retrotransposons, and in particular LTR retrotransposons, accounted for the majority of 24 nt loci. In contrast the majority of 21 nt loci represented miRNAs and predicted phased loci. One known miRNA (ptc-mir398c) was included in the 24 nt loci but in this case the 24 nt sequence originated within the miRNA* region.

**Table 2:** Predicted phased and trans-acting loci.

Location	Sequences	Phased sequences	Number of Targets (perfect match)	Phasing trigger	Genomic context
LG_III:13629357..13629608^1^	7	6	0		CDS
LG_III:13638142..13638393^1^	7	6	0		CDS
LG_III:13654756..13655007	7	5	1(0)		CDS/intergenic
LG_VI:551356..551607	25	9	4(0)	si39621	Intergenic
LG_VI:14747098..14747349	13	6	5(2) MYB	ptc- miR828	CDS
LG_X:19646453..19647187^2^	62	14	8(1)	ptc- miR482.2	UTR
LG_XII:9812366..9812617	20	7	2(0)		CDS/intergenic
LG_XV:5567507..5567758	13	6	3(0)		CDS/intergenic
LG_XIX:138605..138856	12	7	28(1) NBS- LRR		Intergenic
scaffold_70:624107..624358^3^	13	6	10(1) PPR	ptc- miR475c	CDS
scaffold_70:867516..867767	4	4	10(3) PPR	ptc- miR475c	CDS
scaffold_180:454350..454601	7	5	16(7) NBS- LRR		Intergenic

Predicted phased and trans-acting loci with p *<*0.00001. Where a locus targets many genes from a particular class, this is shown after the number of targets. The number of predicted targets at each locus is shown with the number of perfect-match siRNA-targets indicated in parenthesis. Target predictions were made using the psRNATarget web tool. ^1^These two loci are duplicates. ^2^Manually corrected coordinates, as described in Materials and Methods. ^3^There is a second locus just adjacent to this (scaffold_70:624403..624654) with p 0.000026, suggesting that the two may represent a single locus.

The genomic context of sRNAs was examined by identifying overlap to predicted gene models and repeats. The vast majority of sRNAs did not overlap gene-coding loci and most of those that did overlapped with *<*10 unique sRNAs. However, a small number of genes overlapped with a large numbers of sRNAs. For example, the two genes with the highest overlap to sRNAs were estExt_Genewise1_v1.C_91780006 (1,646 sRNAs) and gw1.7267.9.1 (1,351 sRNAs). Nearly all of these overlapping sRNAs map anti-sense to the gene and in both cases there is a ribosomal DNA (rDNA)-like repeat and an LTR retrotransposon within the gene coding sequence. In both cases there was near-equal distribution of sRNAs in all size classes (18-24 mers) suggesting that these are not siRNAs. In contrast other genes with high numbers of overlapping sRNAs showed clear enrichment for a particular size class. For example fgenesh4_pg.C_scaffold_6025000001 showed enrichment for 22 nt and to a lesser extent 24 nt sRNAs and eugene3.00102261 for 21 nt sRNAs. There were also cases where there was near-equal distribution across size classes but with a peak at the smaller sizes (e.g. gw1.376.2.1 and gw1.422.22.1). In these cases, all reads derived from the same strand as the gene and therefore likely represent degradation products of highly expressed genes. These two genes (and two additional genes in the list with *>*100 overlapping sRNAs) encode psbA, with all four having maximal homology to the Arabidopsis gene ATCG00020, which also shows a similar pattern of sRNA overlap. There were 27 genes with *>*100 unique overlapping sRNAs (Additional File 4) and 2,969 genes overlapped to at least a single mapped sRNAs. Excluding those genes with *>*100 overlapping sRNAs, the majority of overlapping sRNAs were 21 mers. Examination of the annotation of the 27 genes with *>*100 overlapping sRNAs did not reveal any functional over-representation of Gene Ontology categories or the presence of particular types of genes.

### TAS3 targets a subset of the ARF (auxin response factor) family

**Figure 2:**
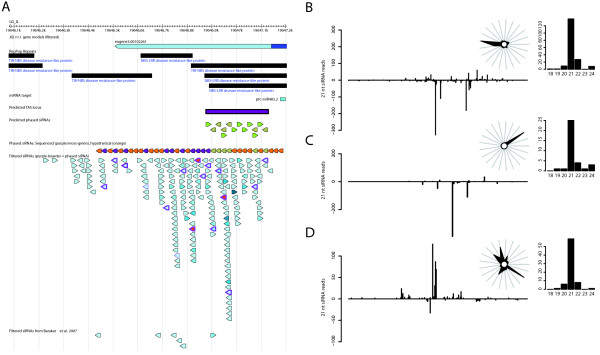
**Populus produces phased siRNAs**. **A **Putative non-conserved trans-acting siRNA locus (LG_X:19646453..19647187). Hypothetical and sequenced small RNAs are shown in orange and purple/moss-green respectively. Regions detected by RepPop are shown as black boxes, predicted miRNA target sites are shown in dark blue and the predicted TAS locus is shown as a purple box. Sequenced siRNA are shown with a black border, with sequences in phase with the TAS locus with a purple border. siRNAs are shaded by raw count value: light-blue to dark blue in the range 1-100 and orange *>*100. **B **Sequence counts for 21 nt siRNAs in the region in A. Inset: Phasing register expression plot showing clear phasing and small RNA size distribution showing a majority of 21-mers from the region. **C **Sequence counts for 21 nt siRNAs within (LG_VI:551356..551607) including phase distributions count and small RNA size distribution. **D **Sequence counts for 21 nt siRNAs within the *Populus *ortholog of Arabidopsis thaliana *TAS3a *(LG_X:14146030..14146540), including phase distributions count and small RNA size distribution.

Both [31] (Figure 4 of [31]) and [15] have previously shown conservation across a number of species for both the *TAS3 *miR390 target sites and two trans-acting siRNAs (TAS). *TAS3 *targeting of a gene coding for an Auxin Response Factor (*ARF3*) is important for developmental timing and patterning in *A. thaliana *[32]. This is the only Arabidopsis TAS locus with even limited homology to *Populus *as well as being the only *A. thaliana *TAS with an Exonerate alignment in the PopGenIE genome browser [29]. In the current study, the homologous region in *Populus *produced primarily 21 nt siRNAs (Figure 2D) but was not one of the predicted phased loci. There were three to four dominant phasing peaks produced from the locus (Figure 2D). It is likely that statistical significance for the ta-siRNA prediction tool was not reached for this region due to inadequate sequencing depth, most likely resulting from the locus being weakly expressed: In the Barakat *et al*. dataset [22], only two siRNAs from this locus were found. Examination of the locus in the PopGenIE genome browser showed that there is a distinct, narrow peak of sequence conservation between *A. thaliana *and *Populus *shown in the VISTA [33,34] alignment plots that peaks at the position of the *A. thaliana *D7(+) *TAS3a *sequence and remains high at the D8(+) sequence. There is complete conservation of the D7 and D8 sequences between Arabidopsis and *Populus*. The D8(+) siRNA sequence has four perfect hits in the *Populus *genome. VISTA alignment plots (available via PopGenIE) show that there is a duplicated region on LG_VIII (LG_X and LG_VIII are almost exact duplicates). Despite the high level of conservation between the duplicated regions, there does not appear to have been conservation of the siRNA sequences themselves nor of the miR390 target sites. None of the other perfect hit locations appear to represent duplicated copies of this locus as only the D8(+) sequence is present at each. Conservation of *TAS3 *in rice was reported by [35], suggesting early evolution and strong conservation of this locus. As was the case in rice, the most abundant siRNA produced was not the conserved and functional siRNA.

Target prediction for the 21 nt siRNAs from this locus identified four *ARF *genes (two *ARF3 *homologs, an *ARF2 *homolog and an *ARF4 *homolog) suggesting that both target site and target are conserved. Based on our own examination of the *ARF *family, both *ARF2 *and *ARF3 *appear to be duplicated in *Populus*, with *ARF4 *existing as a single ortholog. In the case of *ARF3 *we identified two additional predicted gene models (eugene3.08470003 and eugene3.150910001) that lie within the same branch of the phylogenetic tree generated for the entire *ARF *family (Additional File 5) but they are truncated and lack the target recognition site for the *TAS3a *tasiRNA. In the case of *ARF2*, one of the duplicates (estExt_fgenesh4_pm.C_LG_XII0386) contains three SNPs within the target site. As one of these SNPs is at the 11th base pair of the target sequence, it is quite probable that this copy of the gene is no longer targeted. The other copy (eugene3.00150845) maintains complete homology to *A. thaliana *within the target site. It would be interesting to examine the functional role of this locus in *Populus*, as in Arabidopsis it has been shown to control vegetative phase transition [32]. This is a trait of particular interest in *Populus *as the long juvenile phase represents a significant limitation to breeding programmes. [36] examined the *ARF *family in detail and identified six potential *ARF2 *genes. However only two of these (the two we also identified as duplicated copies) were from the Jamboree gene model set (v1.1) and as a result the remaining four models that they predicted as *ARF2 *genes were not included in the current analysis.

### Populus produces many phased siRNAs

In *A. thaliana*, all TAS loci identified to date produce phased 21 nt sRNAs, with the phase being set by miRNA or siRNA cleavage [15,31,37,38]. *TAS1 *and *TAS2 *transcripts are targeted by miR173, *TAS3 *by miR390 and *TAS4 *by miR828. While miR390 and miR828 are conserved in *Populus*, there is no evidence of miR173 conservation. [39] showed that there appears to be little evidence for the production of phased siRNAs of other lengths in *A. thaliana*. The UEA plant sRNA toolkit ta-siRNA tool implements the algorithm from [39] to identify potential TAS/phased loci on the basis of such phasing. Using a p value cut-off of 0.001 (see [39]), 28 potential phased loci were identified containing between three and 14 phased sequences (Additional File 6). In the case of the predicted phased locus on LG_X (Table 2) we could extend the predicted phased locus beyond the region identified (Figure 2A) by assuming that 'missing' sequences in relation to a predicted miRNA target site were produced but not present in the current dataset. The production of potentially phased siRNAs alone is, however, not enough to class a locus as *trans*-acting so we concentrated on the 12 predicted highly significant loci with p values *<*0.00001 and used the psRNATarget prediction tool [40] to identify potential targets for phased siRNAs (Table 2, Additional File 6). Seven of these 12 loci were within predicted protein-coding regions, five are intergenic and one spans across the end of a predicted gene model. Of the five loci located in intergenic regions, all but one overlapped to NBS-LRR repetitive elements and we therefore do not classify these as TAS loci. To date, all eight of the confirmed *A. thaliana *TAS loci (*TAS1a-c*, *TAS2*, *TAS3a-c*, *TAS4*) are produced from non protein-coding regions. Phased loci within coding regions have also been reported but are not classed as *trans*-acting as they typically target in *cis *or target other members of the gene family from which they are produced [14,39]. Using this criterion only the locus at LG_VI:551356..551607 (Table 2, Figure 2B) would be classified as a TAS locus.

Among the identified loci are some interesting examples. On LG_II (LG_II:14322242..14322493, p 0.000014), phased sRNAs are produced specifically from the exons of a predicted *No Apical Meristem *(*NAM*) gene (gw1.II.959.1) with a cluster of sRNAs being produced from both of the two exons and none from the intervening intron, suggesting that this locus is specific to the transcribed RNA. The loci on LG_III and scaffold_80 contain phased siRNAs that do not have unique hits within the genome. In the case of the three loci on LG_III, all three are homologous and contain largely the same set of siRNAs. From the current study it is not possible to determine whether all three loci actually produce siRNAs. In the case of the locus on scaffold_80 there are numerous genomic hits for each siRNA.

[14] found that *Populus PPR-P *genes were predicted to be targeted singularly or dually by either miR475 or miR476. Our data show that *Populus PPR-P *loci do produce siRNAs, with a subset producing phased siRNAs. However, the gene presented in Figure 5 of [14] (eugene3.00062011) contains targets sites for both miR475 and miR476 but did not produce siRNAs in the current study or in that of [22]. The *A. thaliana TAS1 *and *TAS2 *loci specifically target *PPR *genes but there is no evidence of homology between these loci and the ones identified in this study. Therefore it would seem that similar evolutionary mechanisms have been deployed to silence the same gene families in both species. Previously miR475 and miR476 predictions were based on the considerably smaller datasets of [20,21] and had not been well characterised.

We found that particularly miR476 has strong sequence support as a *bona fide *miRNA. The stem-loop structure and sRNA read distribution for the four miR475 and three miR476 loci can been found in Additional File 7 and 8 respectively.

The phased locus on LG_VI generates siRNAs that are predicted to target the *MYB *transcription factor gene from which they are generated. Expression of the siRNAs was low within the sample we sequenced and it is therefore hard to predict whether such a potential loop of transcriptional regulation has functional significance. *TAS4 *in *A. thaliana *specifically targets MYB transcription factors [16] however there is no apparent significant homology between the *TAS4 *locus and the *Populus *locus identified here. However, it is interesting that phasing of *TAS4 *is set by miR828 and that there is a miR828 target site in phase with the locus we have identified.

We also performed a search for predicted genomic target sites of miR390, miR475, miR476, miR482.2, and miR828 to examine whether any predicted targets matched the location of identified phased loci. We included miR482.2 because it potentially sets the phase of the locus identified on LG_X:19646453..19647187 (Table 2). However, this did not yield any additional loci producing sRNAs.

### Chromosome XIX contains a sRNA hotspot

**Figure 3:**
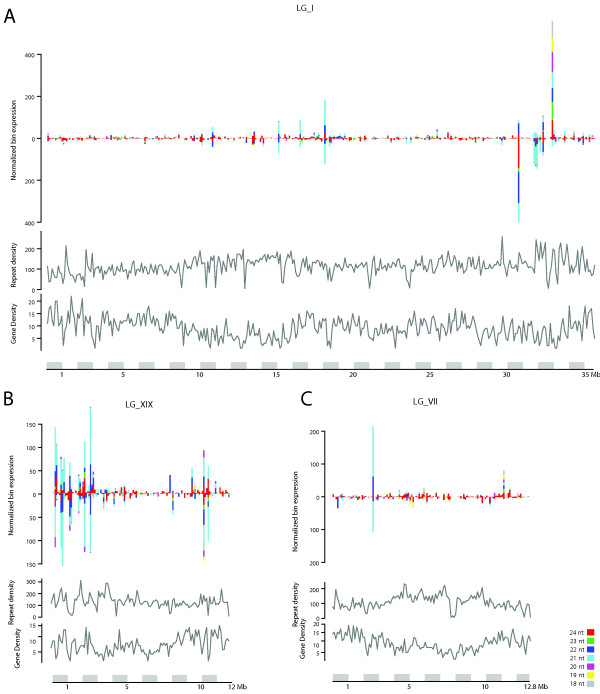
**Genomic distribution of sRNAs in a subset of the 19 Populus chromosomes**. Coloured bars, above the axis for plus strand and below the axis for minus strand, show expression counts in 0.1 Mb windows for 18 (grey), 19 (yellow), 20 (purple), 21 (cyan), 22 (dark blue), 23 (green) and 24 (red) nucleotide sequences along LG_I (**A**), LG_XIX (**B**) and LG_VII (**C**). Below each plot the frequency distribution in 0.1 Mb windows for gene (top) and repeat density (bottom) is shown. Repeat density was calculated using RepeatMasker data from the PopGenIE web resource [29].

The genomic distribution of sRNAs was examined by plotting read counts for each sRNA size class within 0.1 Mb windows across the entire genome. As has been observed in *A. thaliana *[6,16], the location of sRNAs was not evenly distributed across the genome or along chromosomes. Three of the 19 chromosomes are shown as examples in Figure 3 (all 19 chromosomes are displayed in Additional File 9). In general there was a low-level background production of 24 nt sRNAs with the background punctuated by regions of significantly higher production. In the majority of cases, increased sRNA expression was enriched for a particular size class; for example, about half way along LG_X and 1/3rd along LG_VII there were distinct 21 nt peaks. In the majority of cases, these peaks represent highly-expressed miRNAs. There were also regions that produced high numbers of a combination of size classes, for example towards the end of LG_I there was a distinct peak enriched for 21, 22 and 24 nt sRNAs. Specific regions of chromosomes had considerably higher sRNA production compared to the genome-wide background. In particular, the first third of LG_XIX produced significantly greater numbers of sRNAs than did the remainder of the chromosome and the rest of the genome. Interestingly, this is the same region proposed to be in the process of developing characteristics associated with a sex chromosome [8,9] and that also has reduced recombination and enrichment of *NBS-LRR *disease resistance genes [9].

Using the findings presented in [9], we examined this region in greater detail. The available genome sequence for the heterozygous *P. trichocarpa *female represents only one haplotype at each location, with each chromosome sequence representing a chimeric combination of scaffolds from both haplotypes. For the peritelomeric region of LG_XIX, the alternative haplotype is represented by scaffold_117 [9], a fact that can be confirmed by examining the presence of the mapped genetic markers ORPM 276 and ORPM 277 (see [9] for details). The two haplotypes for this region are highly divergent, with contrasting gene content [9]. Of the genes that are in common between the two haplotypes, the vast majority are *NBS-LRR *genes. Here we show that only the haplotype represented in LG_XIX contains the identified hotspot for sRNA production, with sRNA production being minimal for scaffold_117 (Additional files 10 and 11). The phased locus identified on LG_XIX is also specific to this haplotype. Target predictions for the phased siRNAs and for other sRNAs that had unique alignments to this region of LG_XIX showed almost exclusive targeting of *NBS-LRR *genes. Of genes targeted by non-phased sRNAs and located on linkage groups (interpreting genes on scaffolds is ambiguous and these are therefore ignored), 26 of 51 genes were located on LG_XIX, 18 of which are located within the first 1 Mb of the linkage group and all of which are *NBS-LRR *genes. Although the majority of genes shared between the two haplotypes for this region are *NBS-LRR *genes, no target sites were identified for genes on scaffold_117 and examination of paralog data at PopGenIE suggests that the targeted genes are not those with high similarity to genes on scaffold_117. The notable over-represented production of sRNAs from this region in both the 21 and 24 nt size classes and the pattern of *NBS-LRR *gene targeting leaves open the possibility that sRNAs and *NBS-LRR *genes within this region have a role to play in sex determination or the maintenance of reduced recombination. The observed haplotypic divergence for these features is certainly intriguing.

### Prediction of novel non-conserved miRNAs

Comparison of all siRNA reads to Viridiplantae mature miRNA sequences in miRBase (Release 13.0) identified matches to 45 miRNA families (allowing two mismatches including 5' and 3' overhang, Additional File 12). The number of matching families increased to 65 when matches were searched for in the miRNA precursor sequences. There are currently 43 *Populus *miRNA families in miRBase. Perfect matches to 38 of these were identified when matching to precursor sequences and 34 when matching to mature miRNAs. Matching to precursor sequences was performed as we noticed a number of cases where we sequenced a high copy number 21 nt sequence that was predicted as a miRNA but did not match the current miRBase mature miRNA entries. As an example, we identified a sequence matching ptc-miR827 with an expression count of one. At the corresponding genomic locus a miRNA was predicted within our dataset but with the miRNA and miRNA* sequences reversed compared to the current miRBase entry. In our dataset, the miRNA* had a read count of 12. A miRNA corresponding to the current miRBase entry was predicted in [22], in which no miRNA* sequence was found. This suggests that the two sequences may be under differential control.

Sequences matching the mature miRNA sequence of two conserved families (miR1511 and miR858) that do not currently have *Populus *mirBase entries were found with sequence counts of 36 and 17 respectively. Neither of the regions where these sequences map to were predicted as miRNAs within our dataset. The sequence matching miR1511 has 19 perfect hits within the genome and two hits containing two mismatches. The majority of these hits are within LTR retrotransposons (based on RepPop annotations) and none of the locations has evidence of a miRNA* sequence. This makes it unlikely that any of the loci represent an actual miRNA, although there is still the potential that the generated sRNA could serve a targeting function. The sequences matching miR858 have two perfect hits within the genome. The matching sequence was also present at a high sequence count (312) in the data of [22], however no potential miRNA* sequence was found in either dataset. Neither locus produces a convincing hairpin structure, making it unlikely that these are actual miRNAs, despite the perfect homology to mature miRNA sequences in other plant species. Further work is needed to determine whether these loci produce siRNAs that result in target cleavage.

The UEA plant sRNA toolkit miRCat tool identified 414 potential miRNAs (Additional File 13). In *A. thaliana*, miRCat has been shown to have *>*90% sensitivity for detecting known miRNAs and to have a specificity of *>*99% [23] when applied to the comparable dataset of [16]. Target predictions were run for all predicted miRNAs. All predicted miRNAs matching existing miRBase entries had predicted targets. Predicted miRNAs matched 143 of the 237 *Populus *miRNAs currently in miRBase, with members from 29 of the 43 families included. Of the remaining sequences, 156 had no predicted targets and 115 had predicted targets. None of the predicted miRNAs without predicted targets appear to be conserved. Functional enrichment for Gene Ontology (GO [41]) Biological Process categories was carried out for all predicted targets of existing miRBase miRNAs and for all novel predicted miRNAs from this study. In both cases there was dramatic and nearly exclusive over-representation of processes associated with development and pattern formation/specification.

Although all of the predicted miRNAs fulfil the requirements of expression and foldback, the need for stringent annotation criteria has become increasingly evident. [42] argue that small RNAs that derive from regions apparently able to form hairpins could very well represent false-positives, and that further evidence such as DCL1 dependency or detection of a miRNA* is required to classify a locus as a *bona fide *miRNA [43]. Since we do not have data on DCL1 dependency, we used the presence of a miRNA* sequence as a classification criterion. However, for predicted miRNAs matching existing miRBase entries only 66 of 143 sequences (46%) had a miRNA* sequence present in our dataset and the majority of current miRBase entries have no miRNA* support.

For novel predicted miRNAs with predicted targets, 12 of 115 sequences (10%) had a miRNA* sequence (Table 3, Additional File 14), and 8 of 156 sequences (5%) with no predicted targets had a miRNA* sequence. Details of all predicted miRNAs are given in Additional File 13 and all secondary structures in Additional File 15.

**Table 3:** Novel predicted miRNA loci.

Sequence	Location	Count	Hairpin length	Predicted Targets
TCTTTCCGAGTCCTCCCATACC	scaffold_155:237816..237837	147	171	Yes
TCGTAATGCTTCATTCTCACAA^1^a	scaffold_148:201299..201320	49	90	Yes
TCGAATTTGGGCTTGAGATTGa	LG_III:7559439..7559459	107	101	Yes
TTGTAAGGGAAGCCCACATGG^3^a	LG_I:1810419..1810439	971	119	Yes
TCTTGCTCAAATGAGTATTCCA^1^	LG_XV:698203..698224 (miR828)	55	148	Yes
TTCATTCCTCTTCCTAAAATGG^1^, ^3^	LG_XII:8278996..8279017	647	142	Yes
TCTTGCTCAAATGAGTATTCCA	LG_XII:2137739..2137760 (miR828)	55	146	Yes
CATCTGCAGACTACTTGCCTTa	LG_XIV:3657623..3657643	5	108	Yes
TTCATTCCTCTTCCTAAAATGG^3^	scaffold_129:380652..380673	647	128	Yes
TCATGAATTCAACCTGATTGGa	LG_II:14180794..14180814	106	255	No
AGCTCCGAGCTCTAATTATGTGGG^2^	LG_II:22547443..22547466	20	174	No
TTCCTACAGTTATGATGGCCC^3^a	LG_VI:6175922..6175942	18	115	No
TCTGTCGCTGGAAAGATGGTACa	LG_XVII:3400803..3400824	77	147	No
GGCATGAGGTGTTTGGCAAGA^1^a	LG_II:21846112..21846132	13	126	No
ATATGATGGGTCTCATTTAGTAGA	LG_XIV:2363320..2363343	32	240	No

Novel predicted miRNA loci with an identified miRNA* sequence and a minimum miRNA read count of 5, as identified using the UEA Plant sRNA toolkit miRCat tool. ^1^Overlaps an aligned EST. ^2^Overlaps a repeat. ^3^Predicted in [56]. ^*a*^Shows no homology to existing plant miRNAs.

Within the three classes of data (matching miRBase; not-matching and with predicted targets; not-matching and no predicted targets) there were distinctly different sequence length frequencies. The vast majority (76%) of sequences matching miRBase entries were 21 mers. For novel miRNAs with predicted targets, the largest size class was 21 mers (43%), however a notable percentage of 22 mers (12%) and 24 mers (22%) were included. For sequences with no predicted targets, there was clear over-representation of 24 mers (72%), with 21 mers being the only other notable size group (20%). There were also differences in the frequency of sequences starting with a U, which is characteristic of the majority of miRNAs in *A. thaliana *[16]. For the three classes of predicted miRNAs the percentage of sequences starting with an A, U, C or G respectively was: matching miRBase 5, 79, 15, 1; not-matching, with targets 22, 57, 13, 8; not-matching, no targets 32, 40, 17, 12 (Additional file 16). In all three cases the majority of sequences begin with a U, however the distribution is clearly far more biased towards U in those sequences matching current miRBase entries. It was also the case that most cases not matching miRBase but having an identified miRNA* started with a U. Despite caveats, at least some of the predicted miRNAs will represent non-conserved, young miRNAs but are certainly expressed at low levels and would require considerably increased depth of coverage in order to locate a miRNA* sequence for which a lack of predicted targets is not unexpected [44].

**Figure 4:**
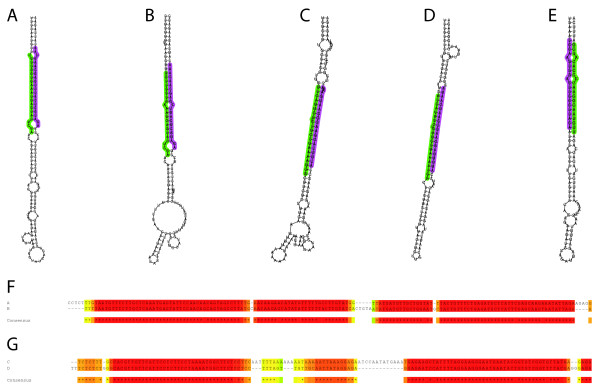
**Novel predicted miRNAs**. **A-B **Hairpin structure for predicted paralogous miRNA with homology to *Arabidopsis thaliana *and *Vitis vinifera *miR828. The two loci are located on LG_XII and LG_XV respectively. **C-D **Hairpin structure for two paralogous novel predicted miRNAs. The two loci are located on LG_XII and scaffold_129 respectively. **E **Hairpin structure for a novel predicted miRNA located on LG_I. In each case the miRNA sequence is indicated in green and the miRNA* in purple. **F **T-Coffee alignment of the two hairpin sequences in A-B. **G **T-Coffee alignment of the two hairpin sequences in C-D. The highest quality alignments are shaded in red and lower quality alignments in yellow. A box shows the regions representing the miRNA (black) or miRNA* (grey).

We examined the three novel predicted miRNAs with the highest sequence counts in more detail (Figure 4A-E). Many of the scaffolds within the current genome assembly are likely to represent haplotypes rather than unassembled sequences. This is particularly true of the shorter scaffolds. VISTA alignment results suggest that the two loci represented in Figure 4C-D lie within duplicated regions, or a least regions that are highly similar and syntenic and Figure 4G shows that they have highly conserved hairpin sequences.

Further work will be needed to clarify whether scaffold_129 represents the other haplotype of the relevant region of LG_XII. Equally, the two miR828 loci represented in Figure 4A-B likely represents paralogs and have highly similar hairpin sequences (Figure 4F).

It is likely that several novel miRNA candidates remain to be found in *Populus*, and deeper sequencing of small RNA libraries from additional tissues, in particular flowers, and conditions is needed. It would, for example, be interesting to sample material before and after the juvenile phase transition. The small number of novel non-conserved miRNAs identified in combination with the evidence that we obtained considerably greater depth of coverage of the small RNA population than previous work suggests that greater depth will now be required to identify miRNAs with moderate to low abundance due to the requirement for a sequenced miRNA*. Sequencing sRNAs from additional tissues and conditions will also help clarify which of the current miRBase ptc-miRNAs can be experimentally confirmed. As was also reported to be the case in rice [35], many of the current *Populus *miRNAs in miRBase lack any experimental evidence and at least some of the predicted miRNAs more likely represent siRNAs.

### A novel locus may encode dual miRNAs

**Figure 5:**
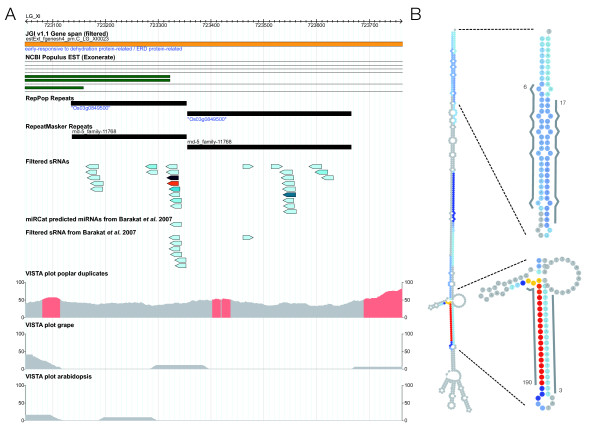
**Potential non-conserved miRNA**. **A **Genomic context of a locus enriched in 21 and 24 nt sRNAs on LG_XI between base pairs 723162..723632. **B **Hairpin structure of the region marked as a red bar in A including the location of potential 21 and 24 nt miRNA and miRNA*sequences. Sequence counts for sRNAs mapping to the hairpin are indicated by colour of shading (blue through to red). The two regions shown in detail indicate predicted miRNA and miRNA* sequences (marked as grey lines) with the corresponding sequence counts shown. Each coloured circle within the hairpin represents one base pair.

An additional 24 nt locus (LG_XI:723162..723620, Figure 5A) that lies within a tandemly-duplicated repeat was located very close to a cluster of unique-hit 21 nt siRNAs, one of which has an expression count *>*100, and for which the siLoCo tool identified a 21 nt loci (LG_XI:723162..723632). Figure 5B shows the hairpin structure for this region and the position of sequenced siRNAs along the hairpin. Evidence for a dual production from transposable elements has been previously reported [45] and production of 21 nt sRNAs from hairpin-like structures has been observed in *A. thaliana *[1], where it was suggested that these may represent recently evolved, or still evolving, miRNAs. The potential mature miRNA sequence shows no similarity to any current entries in miRBase and none of the sequences lie in regions showing evidence of cross-species conservation (Figure 5A). The potential 21 nt miRNA had a detectable miRNA* sequence (Figure 5) and there was also a potential miRNA* for the most highly-expressed 24 nt sRNA. Several variants that differ by one base on either side of this sequence were also highly expressed. It is possible that this hairpin produces two mature miRNAs through different cleavage reactions, or that the 21 nt and 24 nt sequences represent different classes of sRNAs. The hairpin structure for this genomic region results from the high degree of complementarity between a pair of annotated repeats (Figure 5A). The 21 nt potential miRNA has two predicted NBS-LRR targets, both of which are on scaffold_44 (eugene3.00440201 and fgenesh4_pg.C_scaffold_44000194). Viewing the Exonerate alignments of *Populus *proteins for the gene model available at PopGenIE showed that none of the aligned paralogous sequences contain repeats in the same exon and correspondingly none contain any mapped sRNAs.

### miRNA targeting in duplicated genes

Since the *Populus *genome has undergone a relatively recent whole-genome duplication, we were interested in how many pairs of duplicated genes had retained miRNA targeting in both copies. Using psRNATarget predictions of miRNA targeting for all miRBase miRNAs (Release 13.0) in combination with data made available on the JGI *Populus *genome ftp site [46], an analysis of recent duplicated genes was used to identify those duplets (duplicated gene pairs) where only one of the two copies is predicted to be targeted by a miRNA. Across all of the 45,555 predicted *Populus *gene models, 167 (≈ 0.4%) were predicted to be targeted by a miRNA. The duplicated dataset contains 6,699 duplicated gene pairs of which the vast majority (6,663) are not predicted to be targeted by miRNAs. Of the 36 duplets remaining (≈ 0.5%) there were 21 where both duplets are predicted to be targeted by miRNAs and 15 where only one of the two duplet copies is targeted. Alignments of these showed that six duplets lost target predictions due to gene models being highly truncated compared to the other copy with the target site entirely missing. There were SNPs or single base pair insertions in seven cases but none of these were at the 10th or 11th position and so target predictions were lost because the threshold score we set (2) was not met. In these cases it is not clear whether the duplet would still be targeted or not. There was one example of an insertion and one of a deletion that did affect the 10th and 11th position of the target site, which would almost certainly result in a loss of miRNA binding.

## Conclusions

We profiled the sRNA population in *Populus *to a depth far exceeding previous efforts. This allowed identification of novel, non-conserved miRNA loci, phased siRNAs and characterisation of the genomic distribution of sRNAs. We identified a region of LG_XIX overlapping the sex determination region and a major cluster *NBS-LRR *genes as a hot-spot for sRNA production Whether or not sRNAs are more important regulators of developmental transitions in long-lived species such as *Populus *compared to annual species remains to be established.

## Methods

Young, but fully expanded, leaves from *Populus trichocarpa *'Nisqually-1' trees of ≈ 1.5 metres grown in pots in the Umeå University greenhouses under natural light regime were sampled at noon on March 23 2007. Total RNA was prepared using a modified Trizol protocol [47]. Enrichment and RNA cloning of RNAs in the 18 to 24-nts size range was performed as previously described [18] with the following modifications: The 3' adapter (Linker1, CTGTAGGCACCATCAAT) was 5'-adenylated and 3'-dedioxymodified (IDT) and the 5' adapter (Acceptor1, atcgtAGGCACCUGAUA, lower case is DNA, upper case is RNA) was chimeric (IDT). The primer for reverse transcription was ATTGATGGTGCCTACAG (IDT). Re-suspension of first-strand cDNA was carried out in 20 *μ*l 0.1 × TE.

### Pilot experiment

After first-strand cDNA synthesis, amplification was carried out in a 20-*μ*l reaction using 2 U of phusion polymerase (New England Biolabs), 5 *μ*l first-strand cDNA and 5 pmol each of the primers FusionA1 (GCCTCCCTCGCGCCATCAGATCGTAGGCACCTGATA) and FusionB1 (GCCTTGCCAGCCCGCTCAGATTGATGGTGCCTACAG). The amplification product was precipitated using 70% ethanol in the presence of 20 *μ*g glycogen (Ambion), washed once in 500 *μ*l ice-cold 70% ethanol and re-suspended in 20 *μ*l of nuclease-free water (Qiagen). The purified product was subjected to emulsion Polymerase chain reaction (PCR) and massively parallel pyrosequencing on 1/16th of a plate in a GS-FLX instrument following the manufacturer's instructions (Roche).

### Concatenation experiment

After first-strand cDNA synthesis, six parallel amplification reactions were carried out using 1 *μ*l of cDNA template per reaction 5'-monophosphorylated primers. After pooling, nucleic acids were purified using ethanol precipitation as described above. Two 20-*μ*l concatenation reactions were set up, each containing 5 *μ*l purified amplification product, 4 *μ*l 5 × T4 DNA Ligase buffer, 1 *μ*l T4 DNA Ligase (High concentration, Invitrogen) and 10 *μ*l sterile deionized water. The reactions were incubated for 3 hours at room temperature (≈ 20°C). Concatenated DNA was ethanol precipitated as described above, and subjected to ligation of sequencing adapters according to the manufacturer's instructions (Roche). After emulsion PCR, sequencing on a GS-FLX was carried out following the manufacturer's instructions (Roche).

To extract the small RNA sequences from the data we matched tags of the 10 last and 10 first bases of the 5'- and 3'-tags respectively and extracted the sequence between them. After initial tag searching on the sequenced strand, all reads were converted to their reverse complement and the tag searching was repeated. This was done since the blunt concatenation can take place in any direction (as shown in Figure 1A).

### Data processing

All data filtering and analysis was performed using the UEA plant sRNA toolkit [23]. This is an sRNA analysis pipeline for second-generation sequencing data analysis and implements a number of algorithms based on knowledge obtained primarily from sRNA studies in *A. thaliana*. Sequences were filtered to only include 18-24 nt entries, to remove known r/tRNAs and low complexity sequences and to keep only those with perfect matches to the published *P. trichocarpa *genome sequence [12]. The sequenced *P. trichocarpa *clone is the same one used for the current study.

The overlap between sRNAs and genomic features was examined using the data provided at the PopGenIE web resource [29]. All sRNA sequences have been deposited at the PopGenIE web-resource [29] and can be viewed within the main genome browser.

### Phased siRNA and siRNA loci detection

The redundant sequence data was used as input for the siLoCo and ta-siRNA prediction tools. For these tools, all options were left at their defaults. A p value of 0.001 was used for the ta-siRNA prediction tool. The output from the ta-siRNA prediction tool was visualised at PopGenIE and loci with p values *<*10^-^5 were manually inspected to identify whether the loci could be further extended by allowing for sequences missed due to low expression/lack of sequencing depth.

### miRNA prediction

The miRCat tool was used to identify putative miRNA sequences using the redundant filtered sequence data. A minimum sRNA abundance of 2 was set with a maximum of 16 genomic hits and a size-range of 18-24. This tool also generates a GFF-format file containing details of all predicted miRNAs and non-miRNA sequences. This file was uploaded to the PopGenIE web resource for visualisation. Matches to existing, known *Populus *miRNAs (miRBase Release 13.0 [25]) were identified using PatMan [48]. Two searches were run to identify matches to mature miRNAs, one allowing 2 mismatches and another allowing two gaps. The second was used to allow overhang at the beginning or the end of the miRNA sequence as in many cases, we identified a 21 nt sequence for predicted miRNAs where the current miRBase entry is shorter. No gaps within the mature sequence were allowed. We also searched for matches using the predicted hairpin sequence to miRBase hairpin sequences as there were cases where the predicted mature miRNA represented the currently deposited miRNA* sequence.

### Target prediction

miRNA target predictions were performed for all current *P. trichocarpa *miRNAs in miRBase (Release 13.0) in addition to novel predicted miRNAs and TAS siRNAs from this study. We compared target predictions made using the UEA plant sRNA toolkit target prediction tool, the WMD2 target search tool (Web miRNA Designer [49]), the TargetFinder tool from the Carrington lab [13,31] and the psRNATarget tool available at [40]. For psRNATarget the maximum expectation was set to two and all other options left as default. The psRNATarget tool reports all potential complementary regions between miRNA/ta-siRNA and target sequences using an improved iterative parallel Smith-Waterman algorithm and a weighted scoring schema allowing each mismatch to be weighted according to the mismatch type and position in the query small RNA (cf [40]). We found that there was near-complete overlap between the psRNATarget predictions and those from TargetFinder and the UEA toolkit tool but with fewer predicted targets using the maximum expectation values of two. As this likely represents a situation of having some false-negatives but few false-positives, we decided to use the psRNATarget tool. There was also extensive overlap between the predictions from the TargetFinder and UEA target search tool with less overlap between prediction from the WMD2 target search tool on average (data not shown).

Target predictions for miRBase (Release 13.0) sequences from all tools have been made available at the PopGenIE ftp site and are already included in the genome browser.

Potential genomic target sites (i.e. not within the coding sequence of gene models) were identified using PatMan by searching for complementary matches with up to 2 mismatches or by using the psRNATarget tool to search for targets within over-lapping 3.4 Kb genomic regions. Although likely to exclude a number of *bona fide *targets, we used a maximum expectation value threshold of two in order to minimise false-positives.

### Repeat Masking

We performed both repeat masking of repeats in RepBase [30] using RepeatMasker [50] as well as performing *de novo *repeat identification using the RepeatModeler pipeline [51]. RepeatModeler uses RECON [52], RepeatScout [53] and Tandem Repeat Finder [54] to perform *de novo *repeat identification. The consensus repeat library generated by RepeatModeler was then used with RepeatMasker. The data contained in the RepPop database [55] were also used. All repeat runs performed have been made available at the PopGenIE web resource and can be visualised in the genome browser or downloaded from the ftp site.

### Comparison to previous data

In order to be able to directly compare our data to that of [22], we downloaded their supplementary data and created a redundant sequence file to use as input to the UEA plant sRNA toolkit. This was then used to perform exactly the same analysis as described for our dataset. Comparisons to the Barakat data are therefore based on the results from the UEA plant sRNA toolkit tools rather than the results presented in the text of [22].

All files produced by the UEA plant sRNA toolkit tools for both our data and that of [22] are available for download from the PopGenIE ftp site.

## Abbreviations

nt: nucleotide; U: Uracil; A: Adenine; C: Cytosine; G: Guanine; miRNA: microRNA; sRNA: short RNA; siRNA: short-interfering RNA; TAS: trans-acting siRNA; LG: Linkage Group; Arabidopsis refers to *Arabidopsis thaliana *throughout.

## Authors' contributions

NRS drafted the manuscript and performed sequence analysis. DK performed the sequencing experiments, performed data analysis and drafted the manuscript. NF, KDK and JCC helped draft the manuscript and provided training for short RNA library preparation. JL and SJ conceived and supervised the project and assisted in drafting the manuscript.

## Supplementary Material

Additional file 1 **Significant loci of short RNAs**. Loci identified across all short RNA size classes using the siLoCo tool from the UEA sRNA toolkit. Click here for file

Additional file 2 **Significant loci of short 21 nt short RNAs**. Loci identified for 21 nt short RNAs using the siLoCo tool from the UEA sRNA toolkit. Click here for file

Additional file 3 **Significant loci of short 24 nt short RNAs**. Loci identified for 24 nt short RNAs using the siLoCo tool from the UEA sRNA toolkit. Click here for file

Additional file 4 **Genes with *>*100 Over-lapping siRNAs**. short RNA distribution within genes having *>*100 over-lapping sequences. The frequency within the gene is shown on the plus strand (above) and the minus strand (below) the gene structure. Exons are shown as solid bars and introns as connecting lines. The size class (18-24 nt) frequency distribution is also shown. Click here for file

Additional file 5 **Phylogenetic tree of predicted ARF gene family members**. Phylogenetic tree of predicted members of the ARF gene family. Family members were identified using an HMM model search for the presence of the ARF domain within the Jamboree gene model set. Phylogenetic tree was used using [57]. Click here for file

Additional file 6 **Predicted phased loci**. Phased loci with a p values *<*0.001 using the UEA sRNA toolkit. Click here for file

Additional file 7 **Hairpin structure of miR475**. Diagrammatic representation of the predicted stem-loop hairpin structure of miR475. The read count of sequences along the sequence is indicated using coloured shading. Click here for file

Additional file 8 **Hairpin structure of miR476**. Diagrammatic representation of the predicted stem-loop hairpin structure of miR475. The read count of sequences along the sequence is indicated using coloured shading. Click here for file

Additional file 9 **Chromosome distribution plots**. short RNA, gene and repeat density plots for all chromosomes. Coloured bars, above the axis for plus strand and below the axis for minus strand, show expression counts in 0.1 Mb windows for 18 (grey), 19 (yellow), 20 (purple), 21 (cyan), 22 (dark blue), 23 (green) and 24 (red) nucleotide sequences. Below each plot the frequency distribution in 0.1 Mb windows for gene (top) and repeat density (bottom) is shown. Repeat density was calculated using RepeatMasker data from the PopGenIE web resource [29]. Click here for file

Additional file 10 **Chromosome distribution plot for 1st 1 Mb of chromosome 19**. short RNA, gene and repeat density plot for the 1st 1 Mb of chromosome 19 (LG_XIX). Coloured bars, above the axis for plus strand and below the axis for minus strand, show expression counts in 0.1 Mb windows for 18 (grey), 19 (yellow), 20 (purple), 21 (cyan), 22 (dark blue), 23 (green) and 24 (red) nucleotide sequences. Below each plot the frequency distribution in 0.1 Mb windows for gene (top) and repeat density (bottom) is shown. Repeat density was calculated using RepeatMasker data from the PopGenIE web resource [29]. Click here for file

Additional file 11 **Chromosome distribution plot of scaffold_117**. short RNA, gene and repeat density plots of scaffold_117. Coloured bars, above the axis for plus strand and below the axis for minus strand, show expression counts in 0.1 Mb windows for 18 (grey), 19 (yellow), 20 (purple), 21 (cyan), 22 (dark blue), 23 (green) and 24 (red) nucleotide sequences. Below each plot the frequency distribution in 0.1 Mb windows for gene (top) and repeat density (bottom) is shown. Repeat density was calculated using RepeatMasker data from the PopGenIE web resource [29]. Click here for file

Additional file 12 **Matches to mature miRNA sequences in miRBase**. Counts of sequences matches mature miRNAs for all plant species within miRBase (release 13.0). Click here for file

Additional file 13 **Predicted miRNA loci**. All loci predicted as miRNA using the miRCat tool from the UEA sRNA toolkit. Click here for file

Additional file 14 **Predicted miRNA loci with no match the miRBase**. All loci predicted as miRNA using the miRCat tool from the UEA sRNA toolkit that do not match existing miRBase entries and are therefore candidate non-conserved miRNAs. Click here for file

Additional file 15 **Predicted miRNA hairpin structures**. Predicted stem-loop hairpin structures for all miRNA loci represented in Additional File 11. The location of the miRNA and miRNA*sequence is indicated in green and purple respectively. Click here for file

Additional file 16 **Frequency of starting base pairs for different classes of predicted miRNAs**. The frequency of predicted miRNAs starting with an A, U, C or G for miRNA loci matching existing miRBase entries, not-matching and with predicted targets and not-matching with no predicted targets. Click here for file
