# Supplementary material for: Genome-wide profiling of Populus small RNAs
Source: BMC Genomics. 2009 Dec 20;10:620. doi: 10.1186/1471-2164-10-620 (PMC2811130; doi:10.1186/1471-2164-10-620)
Supplement: Additional file 4 — Genes with >100 Over-lapping siRNAs. short RNA distribution within genes having >100 over-lapping sequences. The frequency within the gene is shown on the plus strand (above) and the minus strand (below) the gene structure. Exons are shown as solid bars and introns as connecting lines. The size class (18-24 nt) frequency distribution is also shown. [file 1471-2164-10-620-S4.PDF]

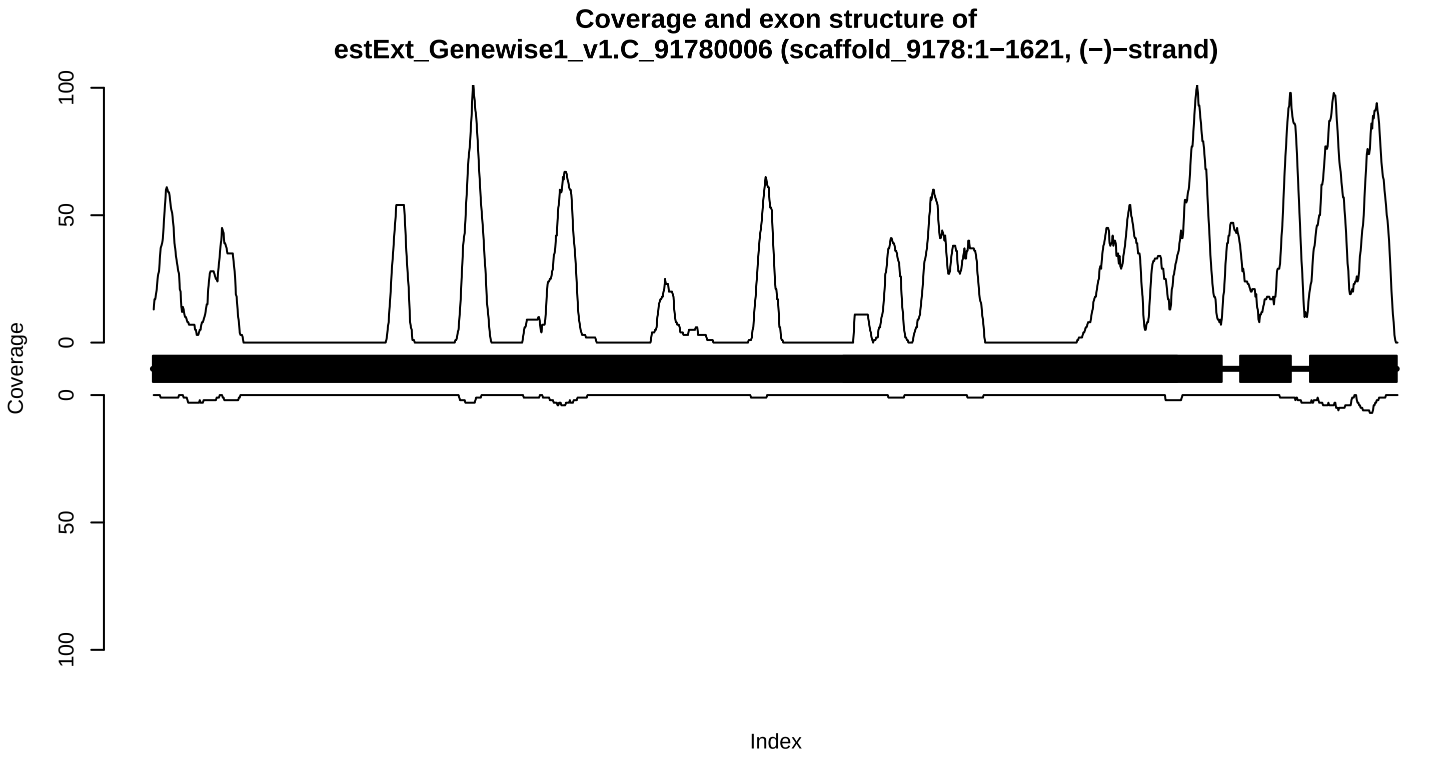
Coverage and exon structure of
estExt_Genewise1_v1.C_91780006 (scaffold_9178:1−1621, (−)−strand)
Coverage
Index
100
50
0
0
50
100

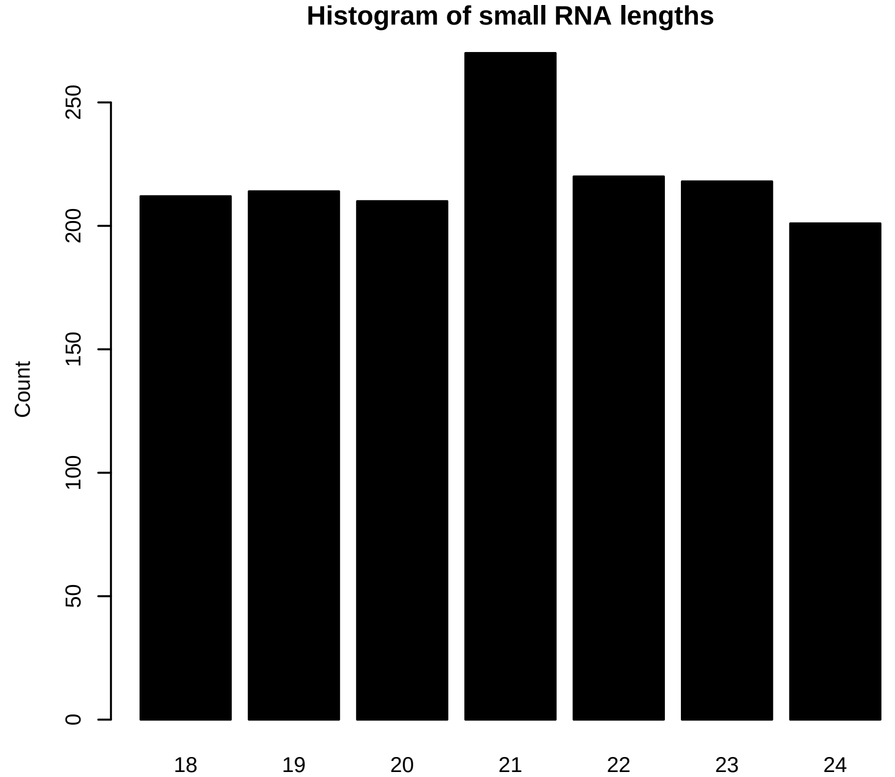
Histogram of small RNA lengths
Count
0
50
100
150
200
250
18
19
20
21
22
23
24

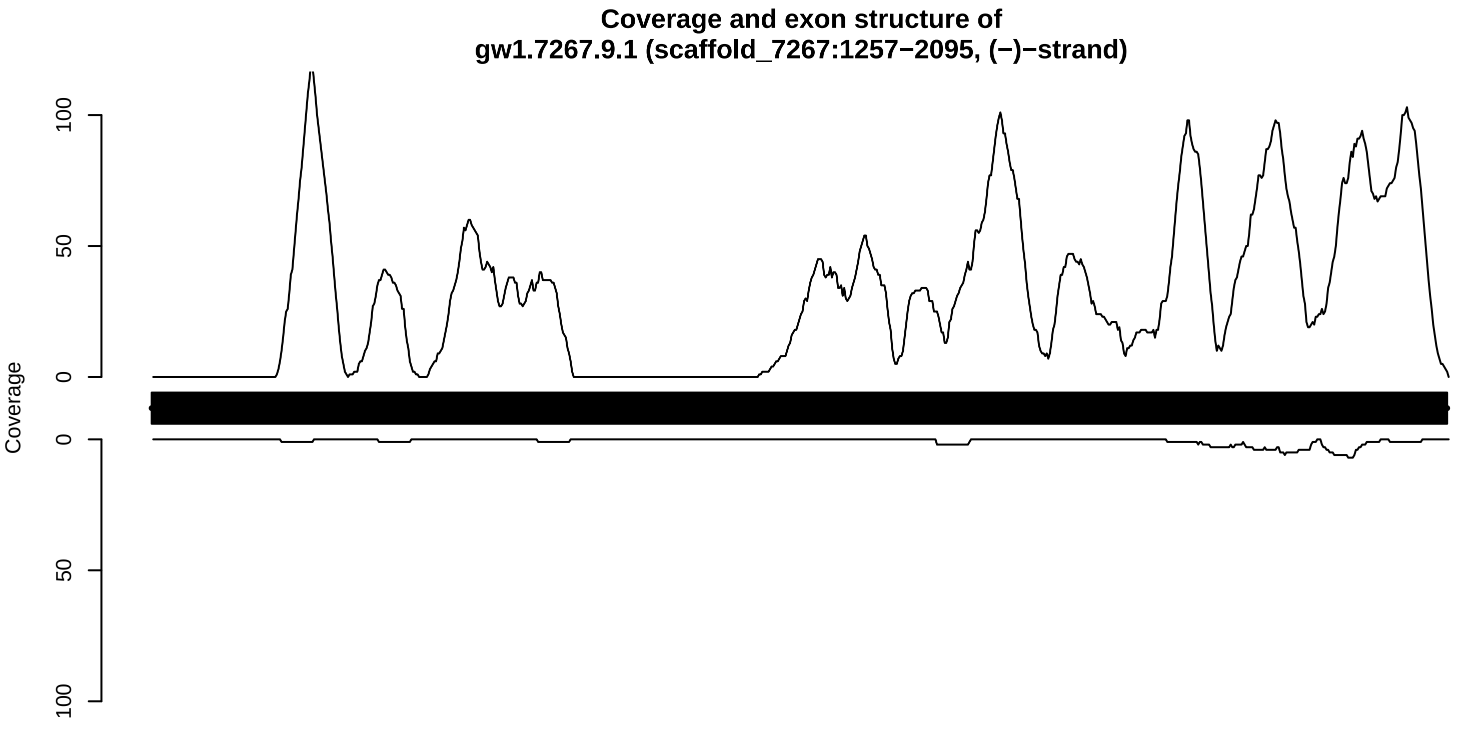

Coverage and exon structure of
gw1.7267.9.1 (scaffold_7267:1257−2095, (−)−strand)
Coverage
100
50
0
0
50
100

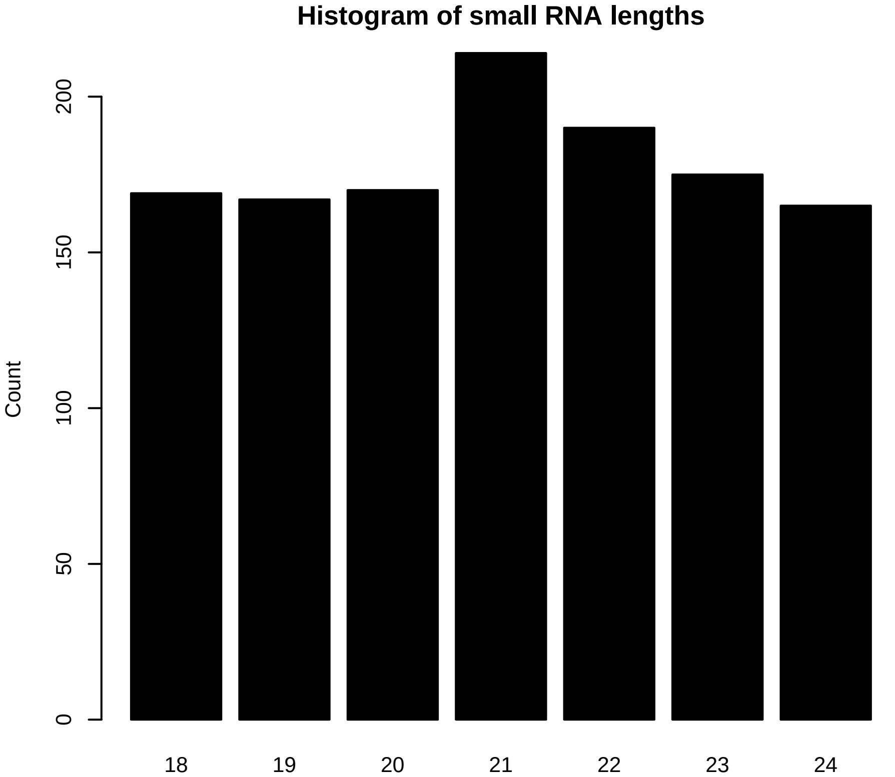

Histogram of small RNA lengths
Count
200
150
100
50
0
18
19
20
21
22
23
24

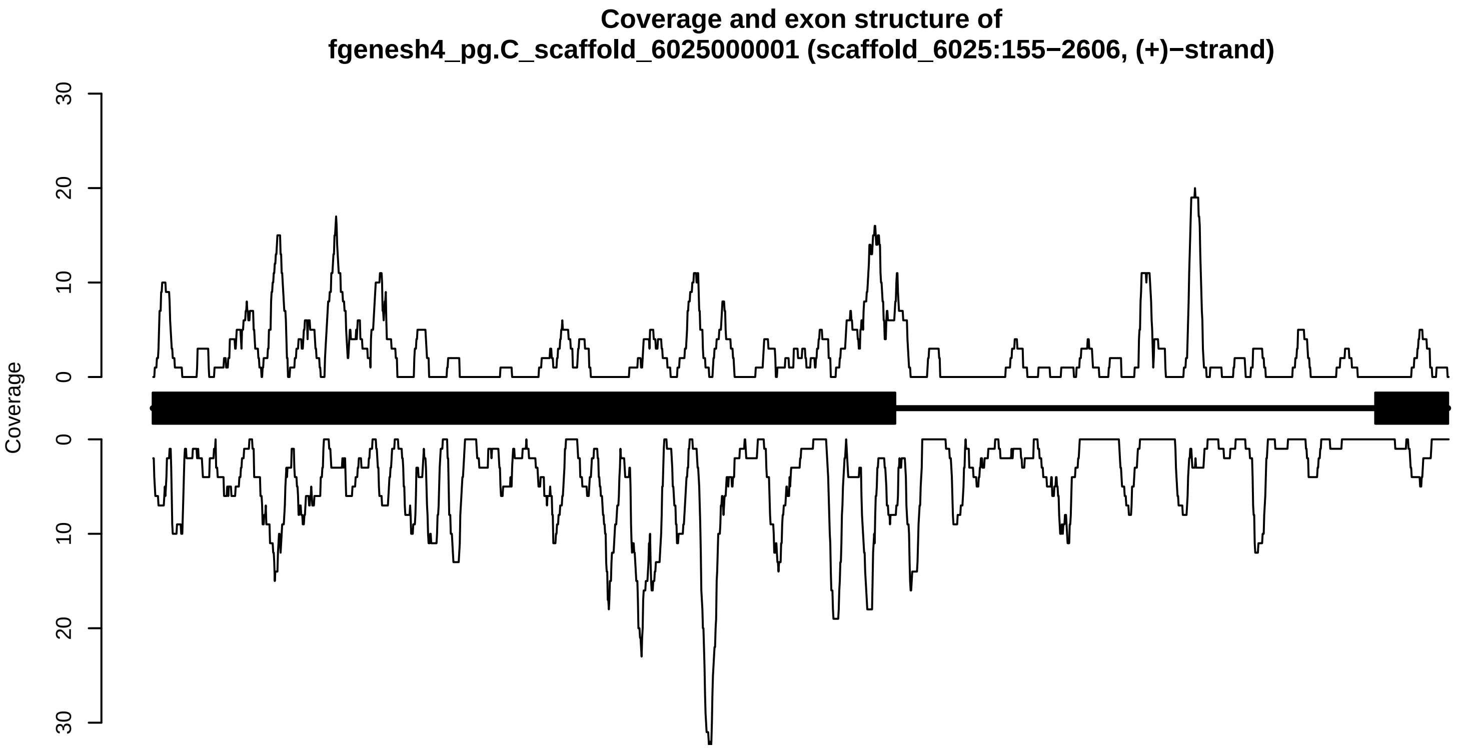
Coverage and exon structure of
fgenesh4_pg.C_scaffold_6025000001 (scaffold_6025:155−2606, (+)−strand)
Coverage
30
20
10
0
0
10
20
30

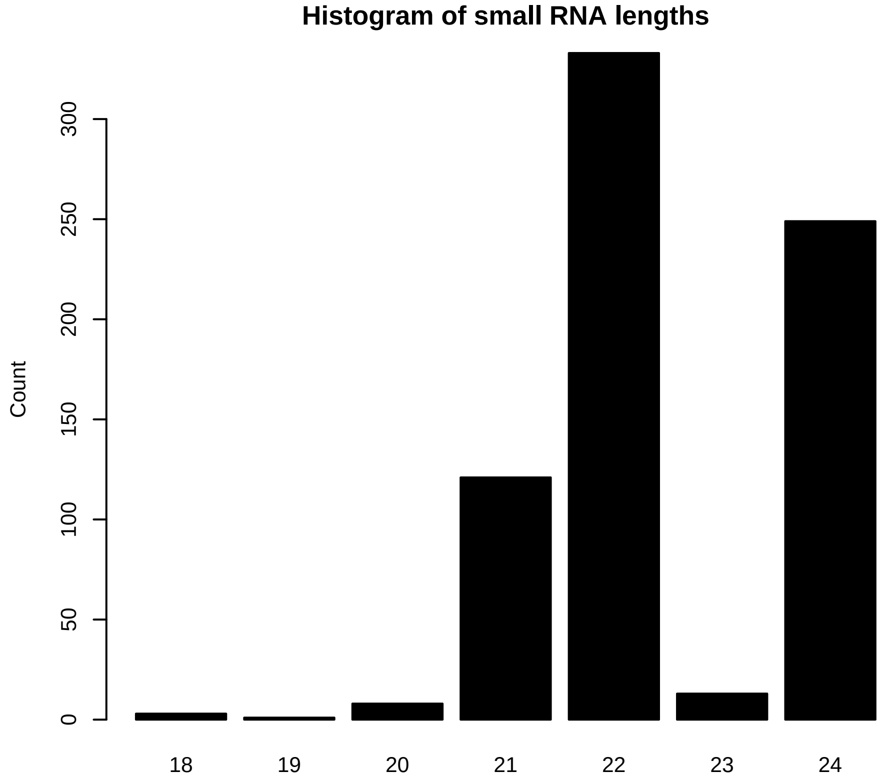
Histogram of small RNA lengths
Count
300
250
200
150
100
50
0
18
19
20
21
22
23
24

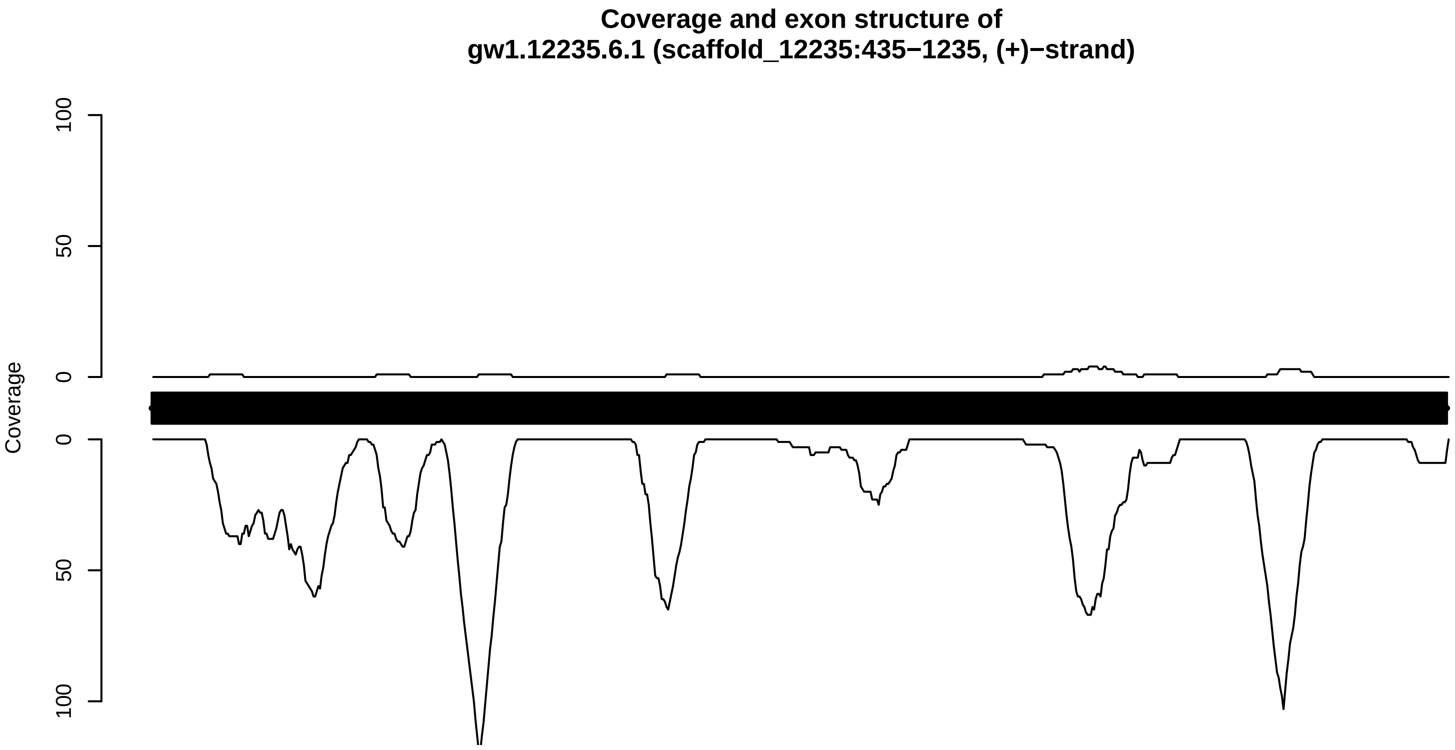

Coverage and exon structure of
gw1.12235.6.1 (scaffold_12235:435−1235, (+)−strand)
Coverage
100
50
0
0
50
100

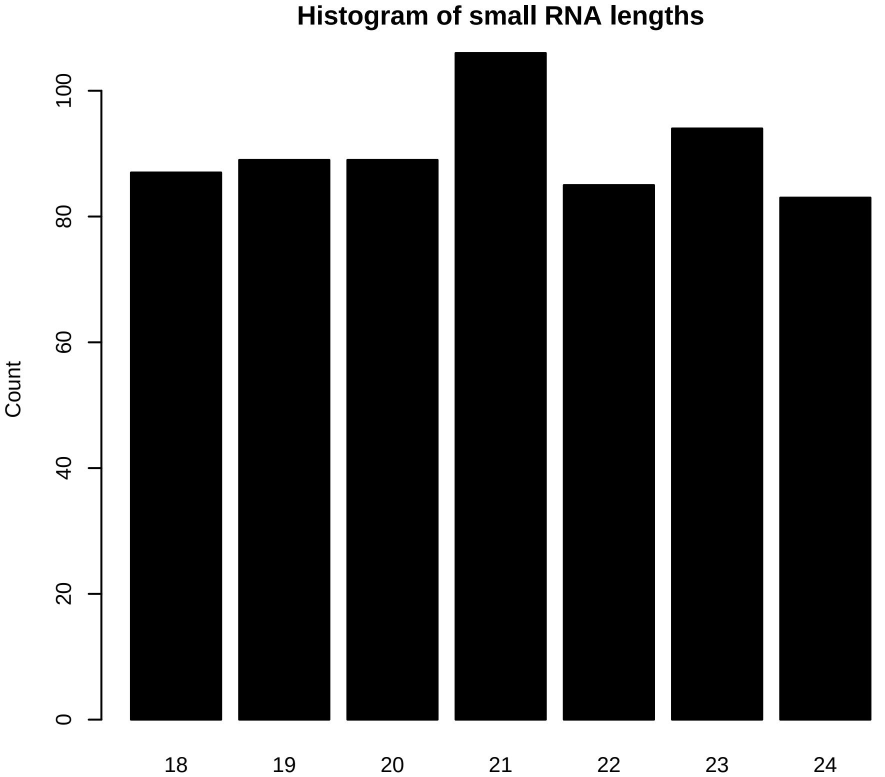

Histogram of small RNA lengths
Count
0
20
40
60
80
100
18
19
20
21
22
23
24

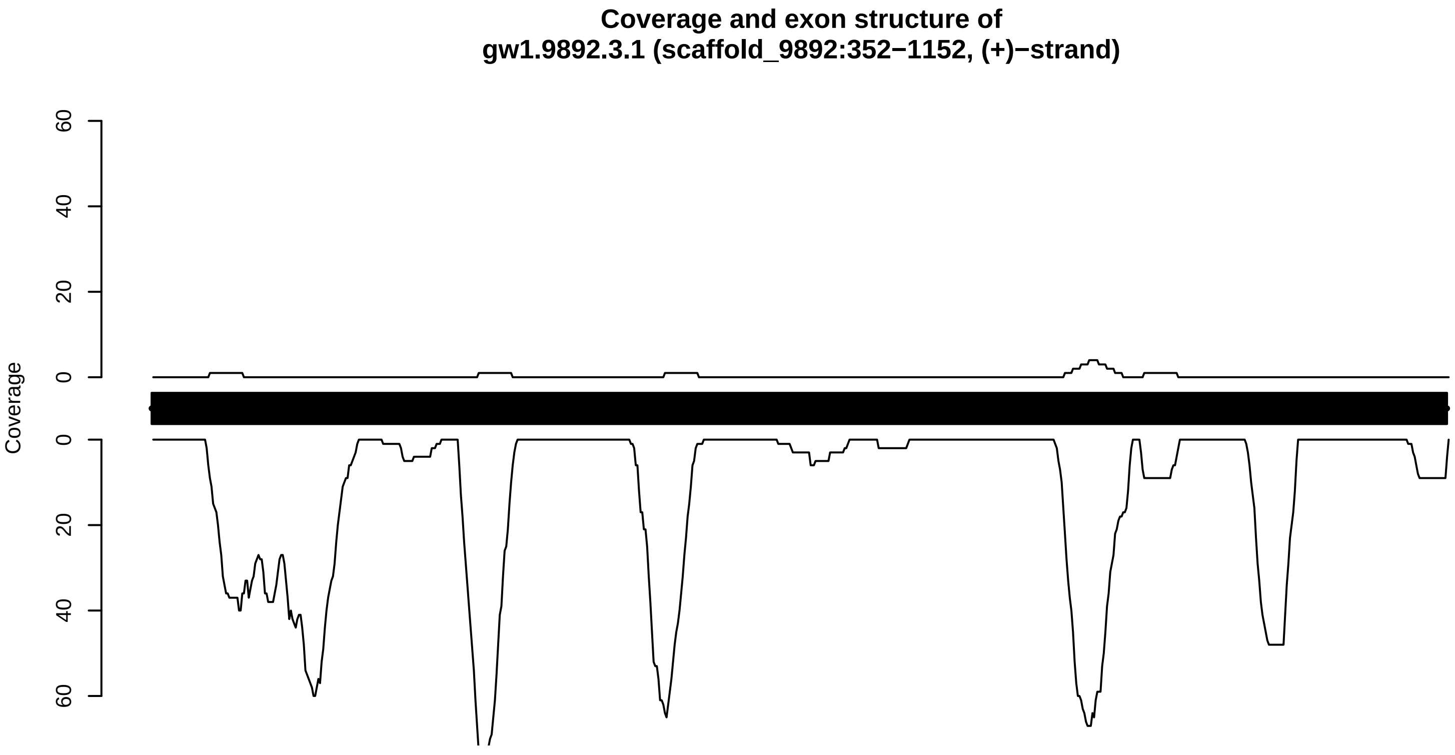
Coverage and exon structure of
gw1.9892.3.1 (scaffold_9892:352−1152, (+)−strand)
Coverage
60
40
20
0
0
20
40
60

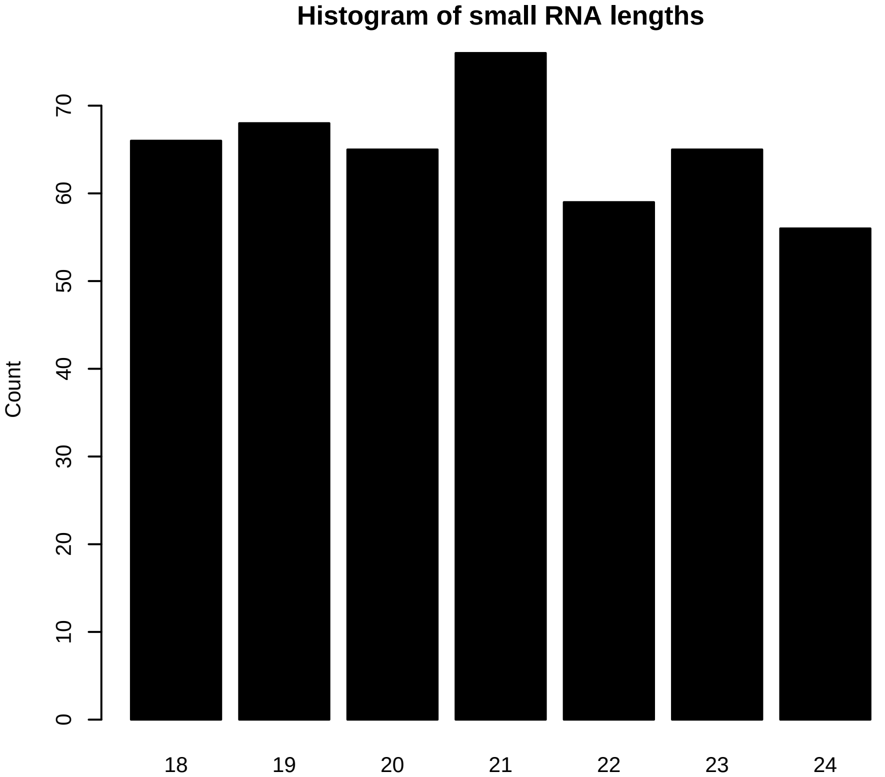
Histogram of small RNA lengths
Count
0
10
20
30
40
50
60
70
18
19
20
21
22
23
24

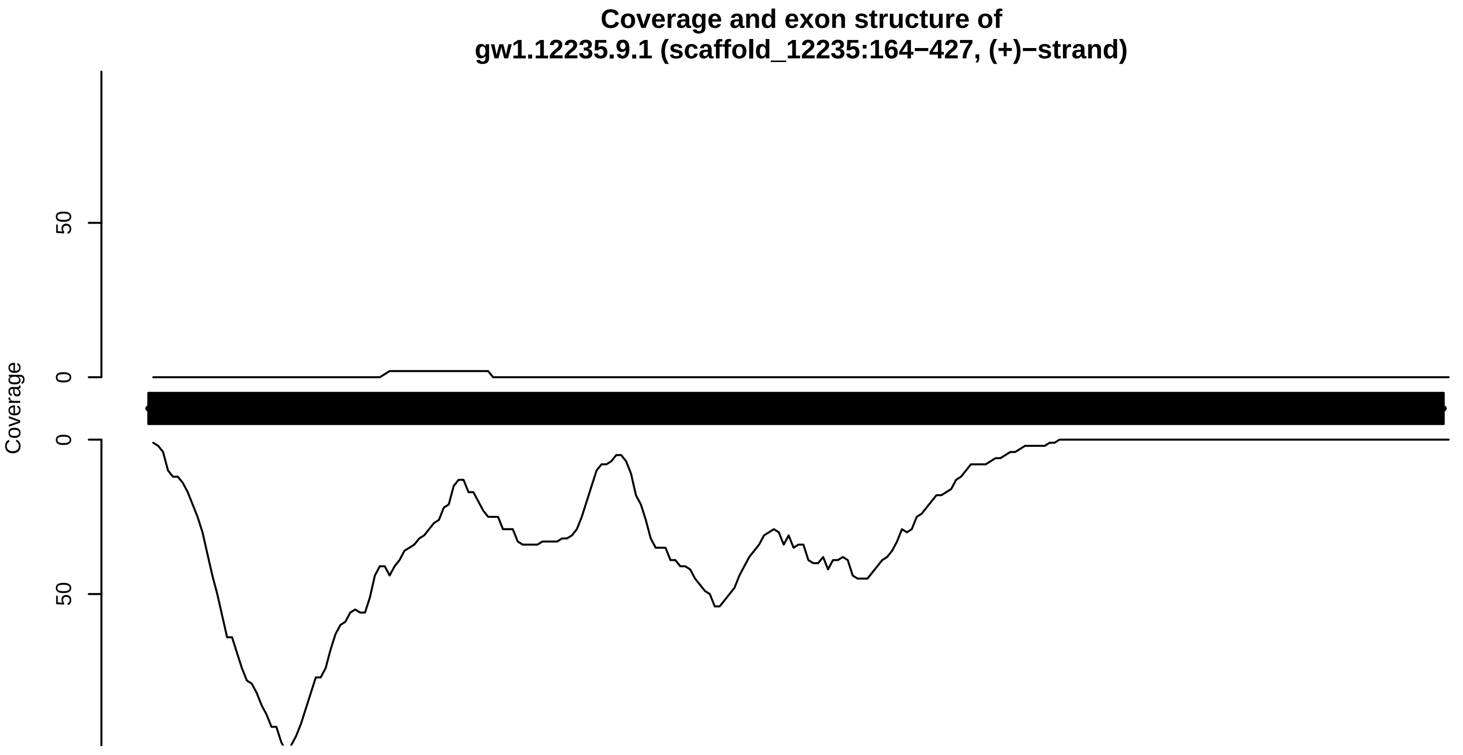
Coverage and exon structure of
gw1.12235.9.1 (scaffold_12235:164−427, (+)−strand)
Coverage
50
0
0
50

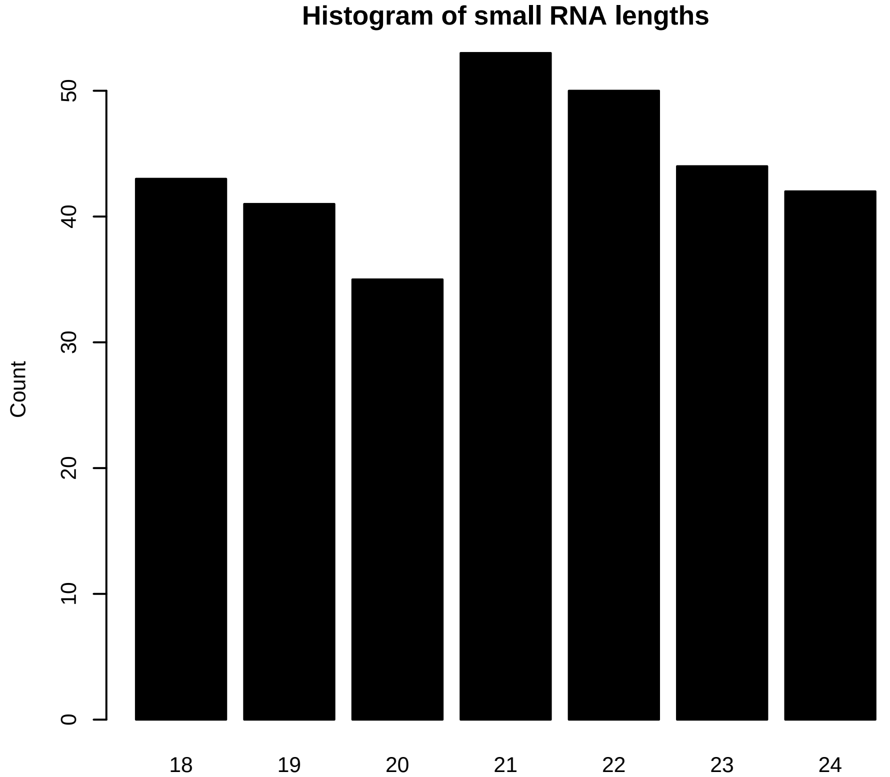
Histogram of small RNA lengths
Count
0
10
20
30
40
50
18
19
20
21
22
23
24

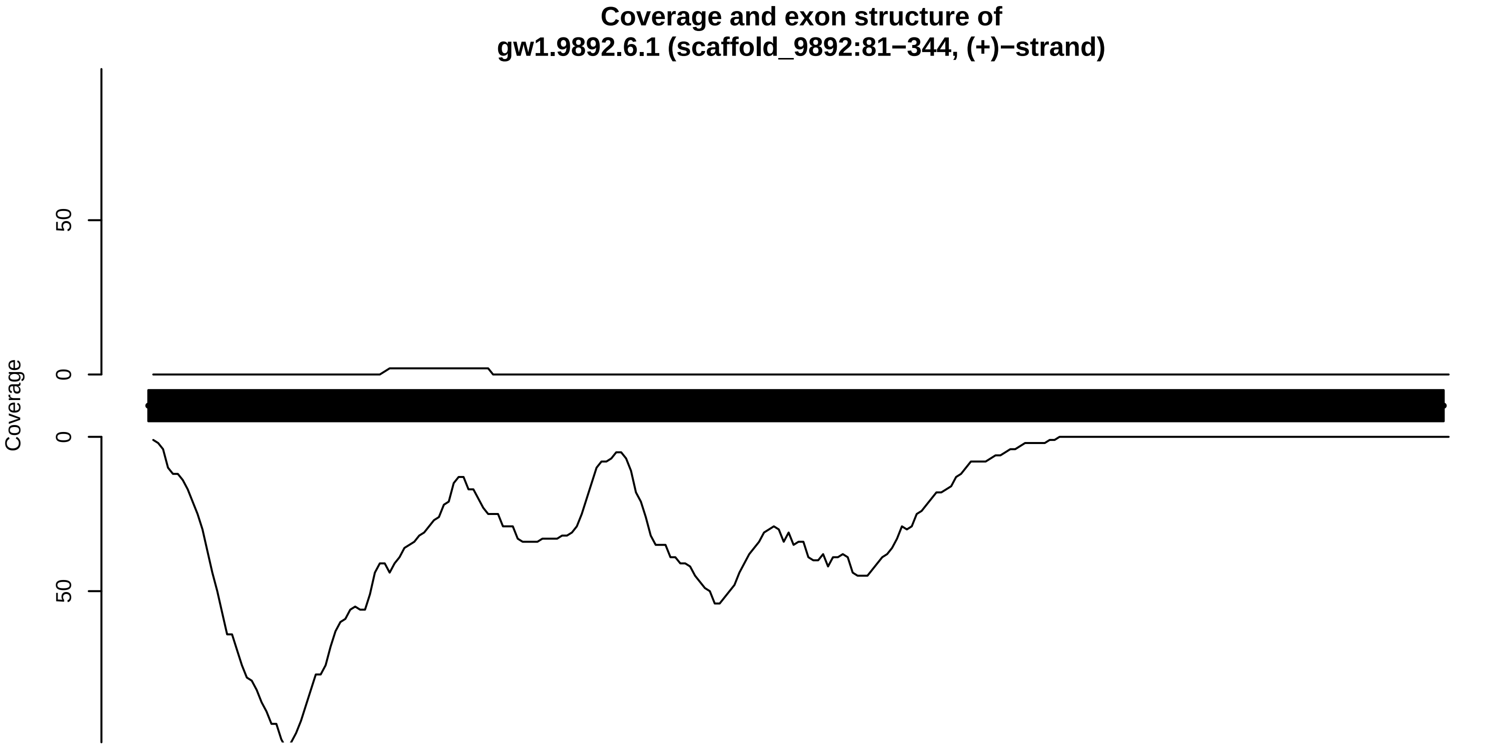
Coverage and exon structure of
gw1.9892.6.1 (scaffold_9892:81−344, (+)−strand)
Coverage
50
0
0
50

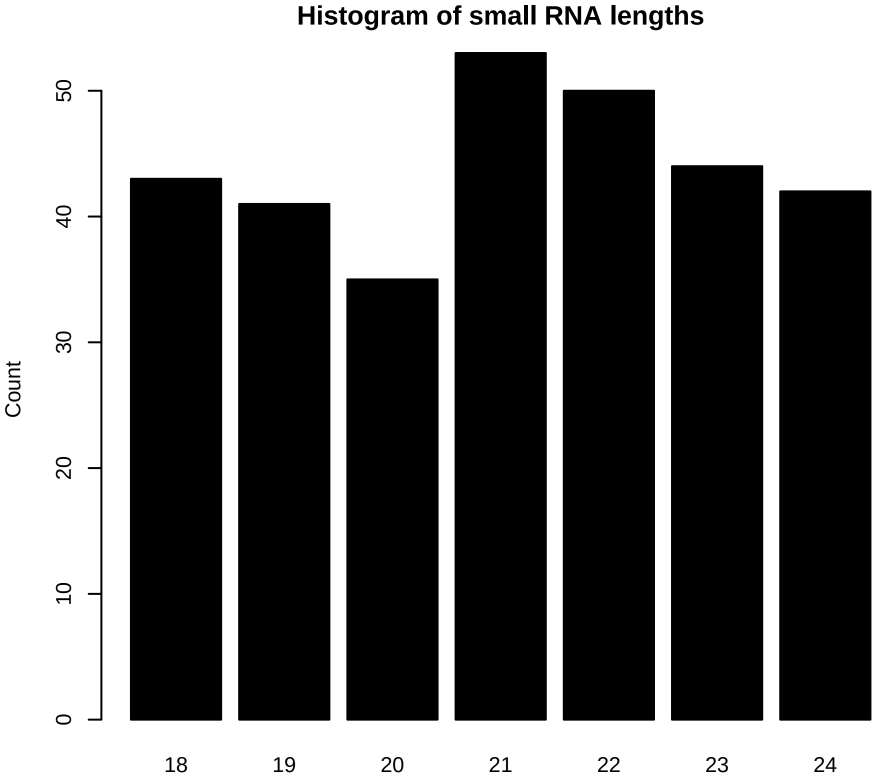
Histogram of small RNA lengths
Count
0
10
20
30
40
50
18
19
20
21
22
23
24

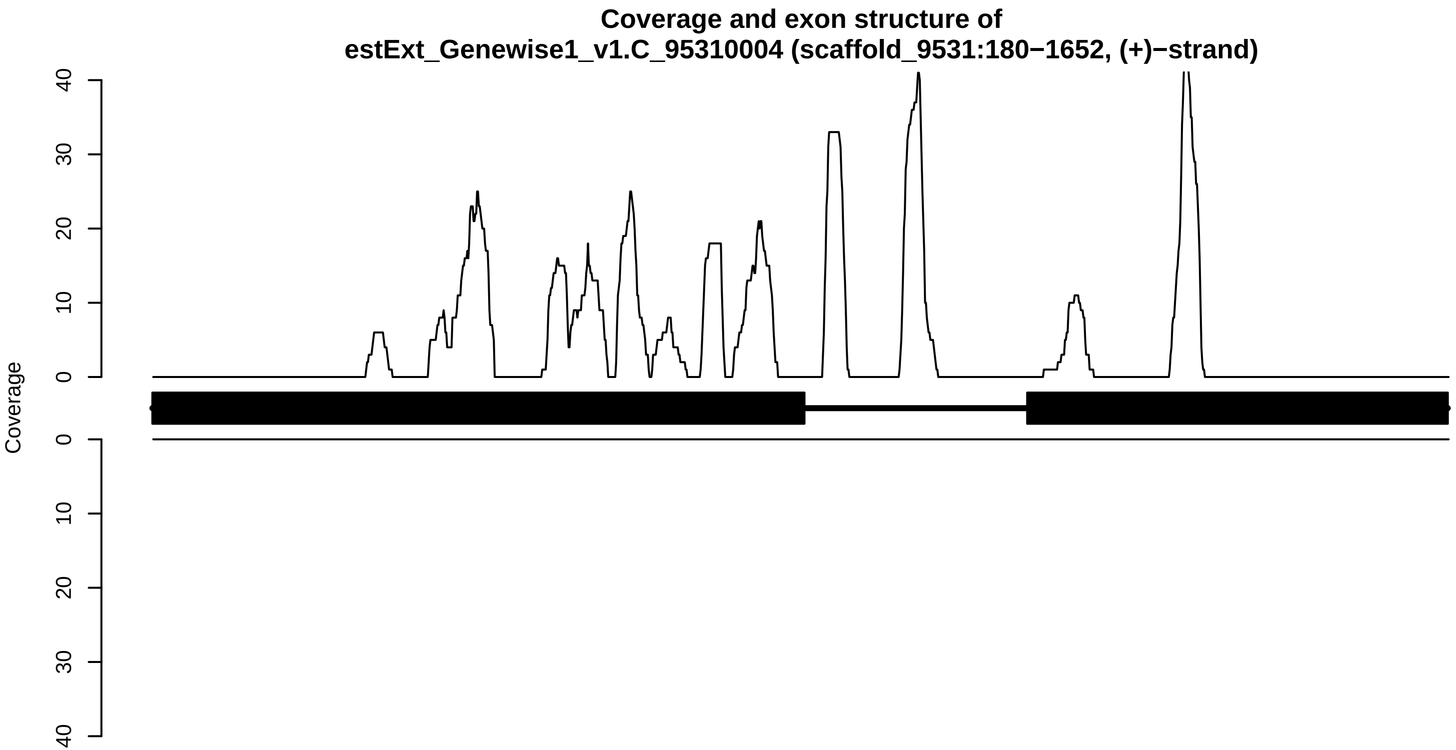
Coverage and exon structure of
estExt_Genewise1_v1.C_95310004 (scaffold_9531:180−1652, (+)−strand)
Coverage
0
10
20
30
40

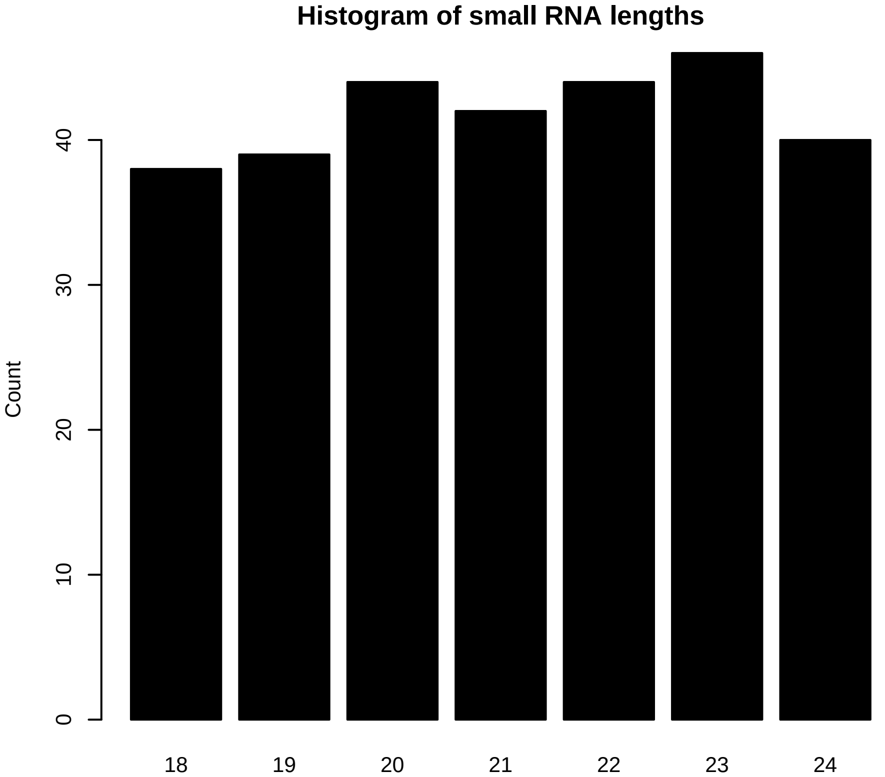
Histogram of small RNA lengths
Count
0
10
20
30
40
18
19
20
21
22
23
24

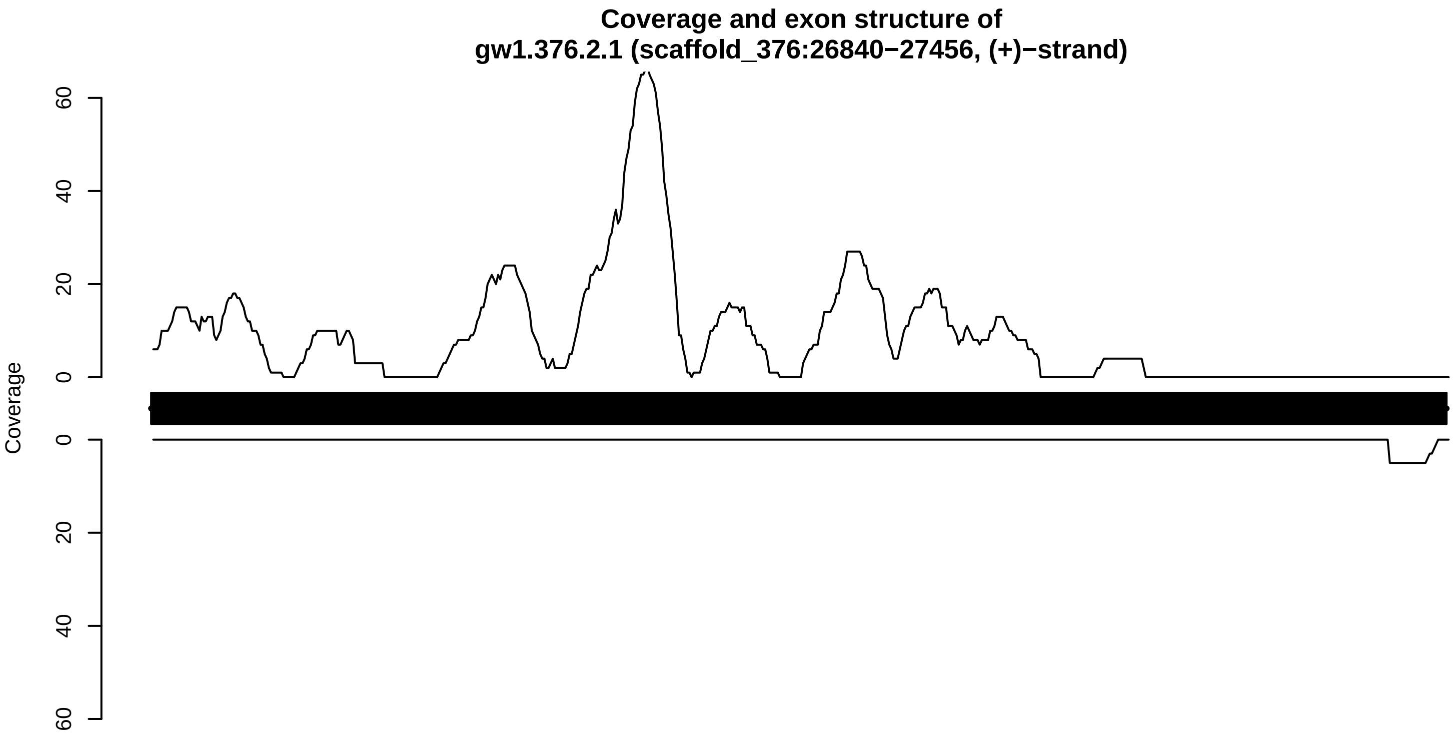
Coverage and exon structure of
gw1.376.2.1 (scaffold_376:26840−27456, (+)−strand)
Coverage
60
40
20
0
0
20
40
60

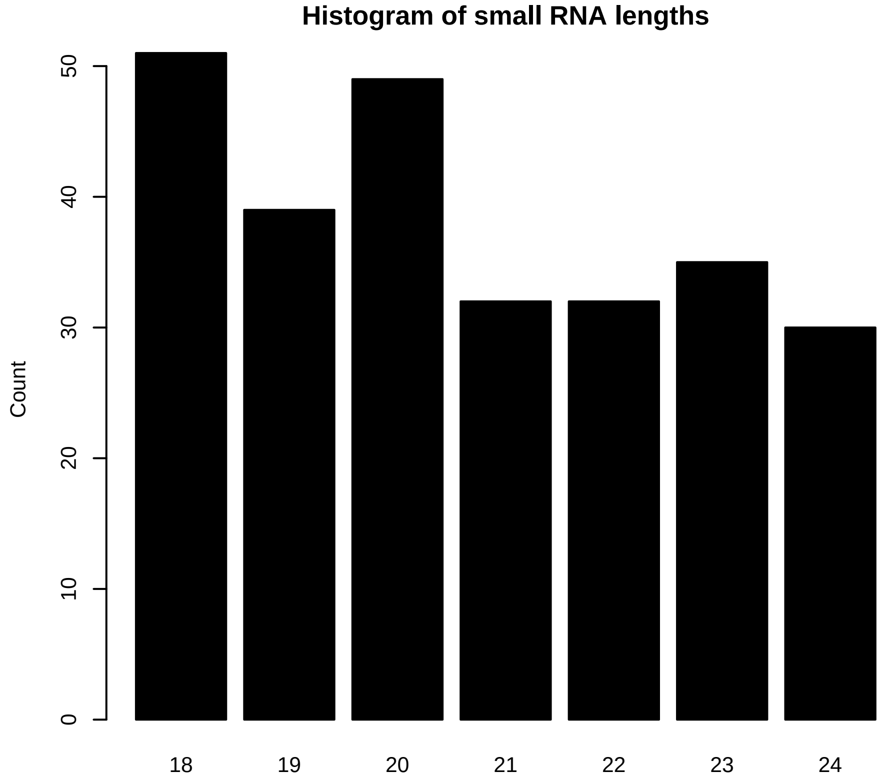
Histogram of small RNA lengths
Count
50
40
30
20
10
0
18
19
20
21
22
23
24

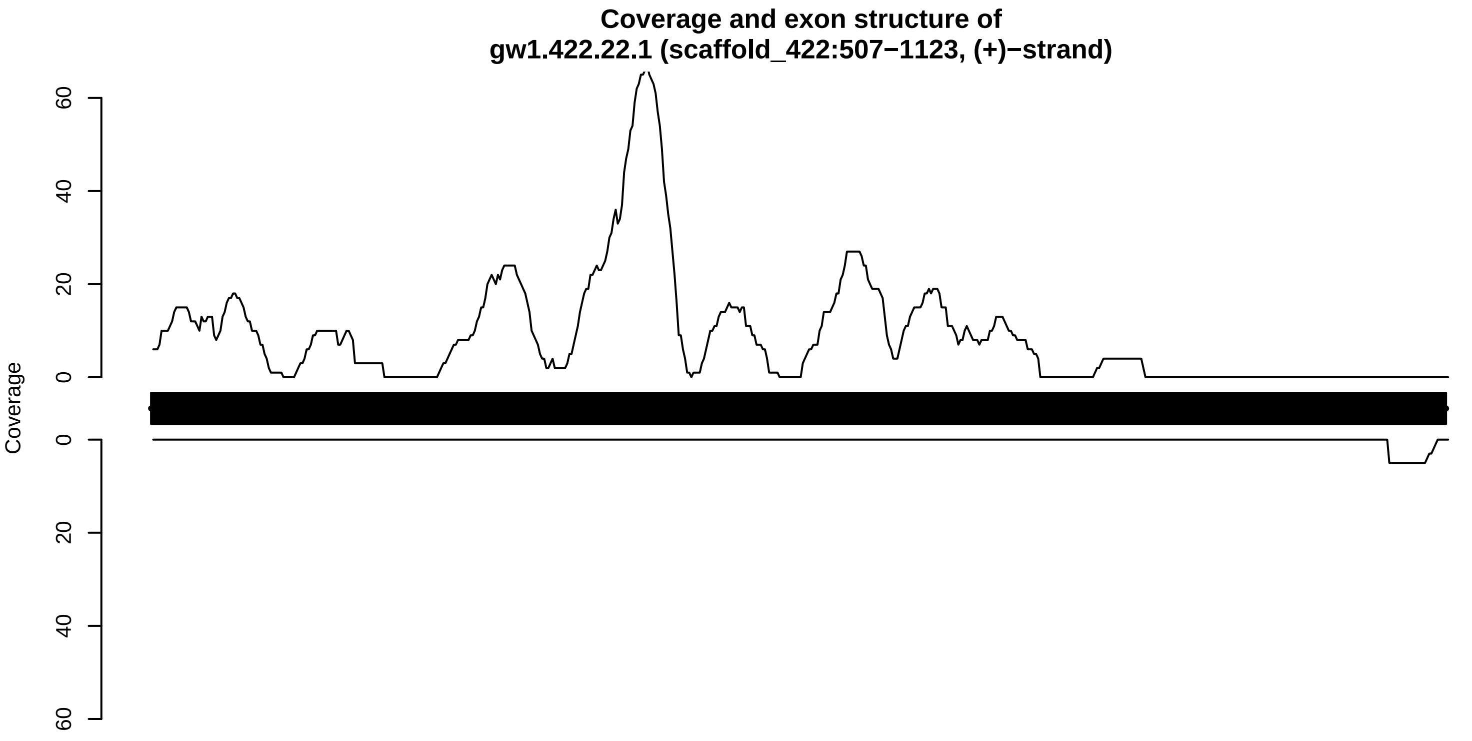
Coverage and exon structure of
gw1.422.22.1 (scaffold_422:507−1123, (+)−strand)
Coverage
60
40
20
0
0
20
40
60

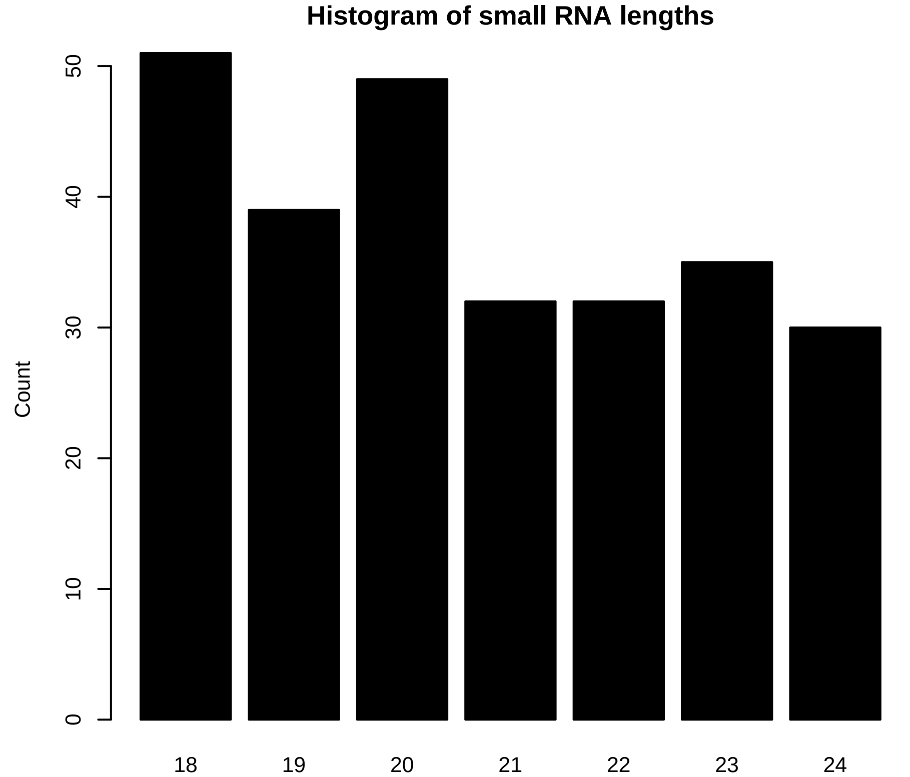
Histogram of small RNA lengths
Count
50
40
30
20
10
0
18
19
20
21
22
23
24

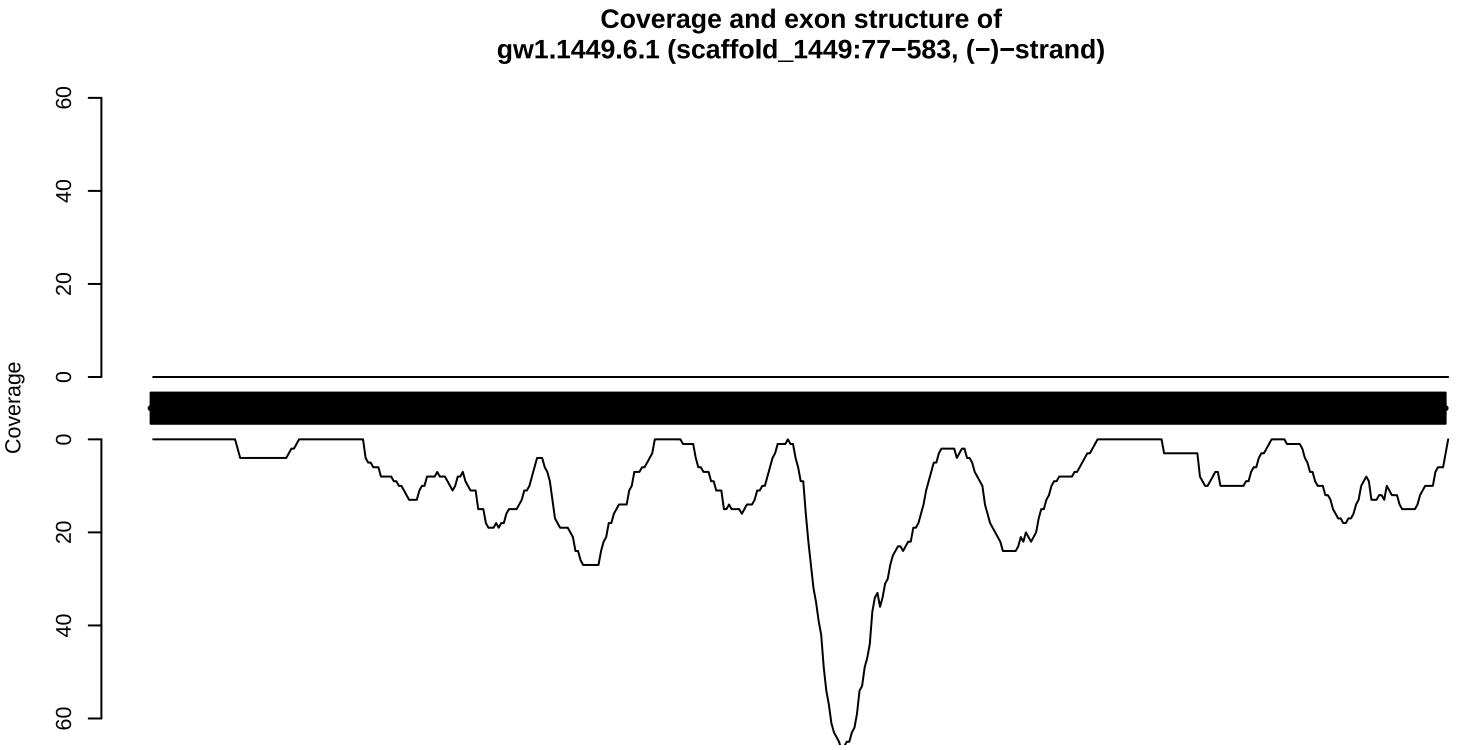
Coverage and exon structure of
gw1.1449.6.1 (scaffold_1449:77−583, (−)−strand)
Coverage
60
40
20
0
0
20
40
60

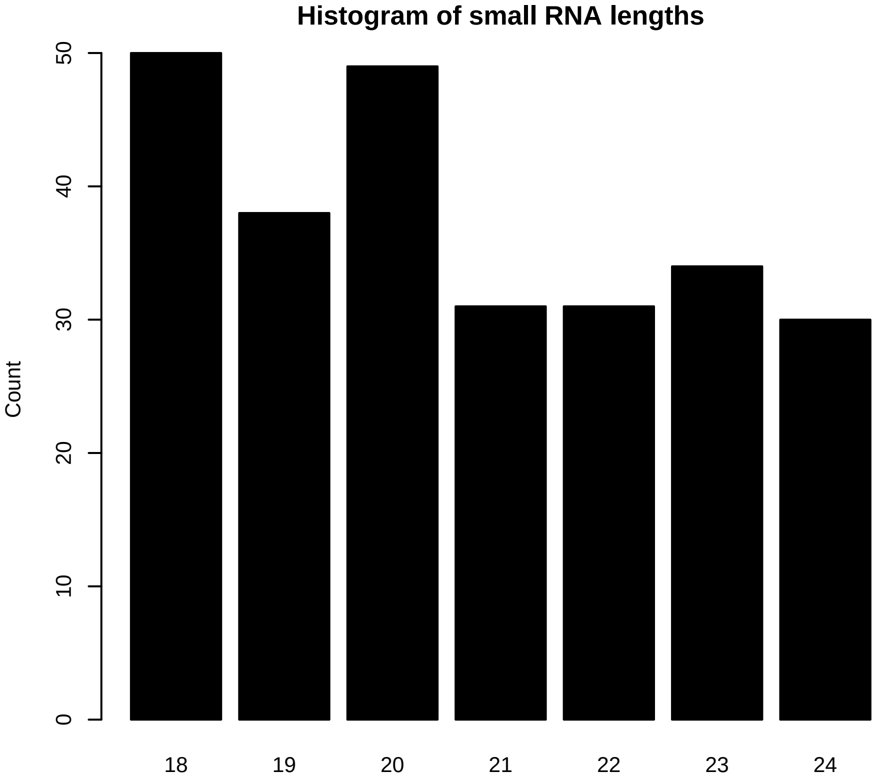
Histogram of small RNA lengths
Count
0
10
20
30
40
50
18
19
20
21
22
23
24

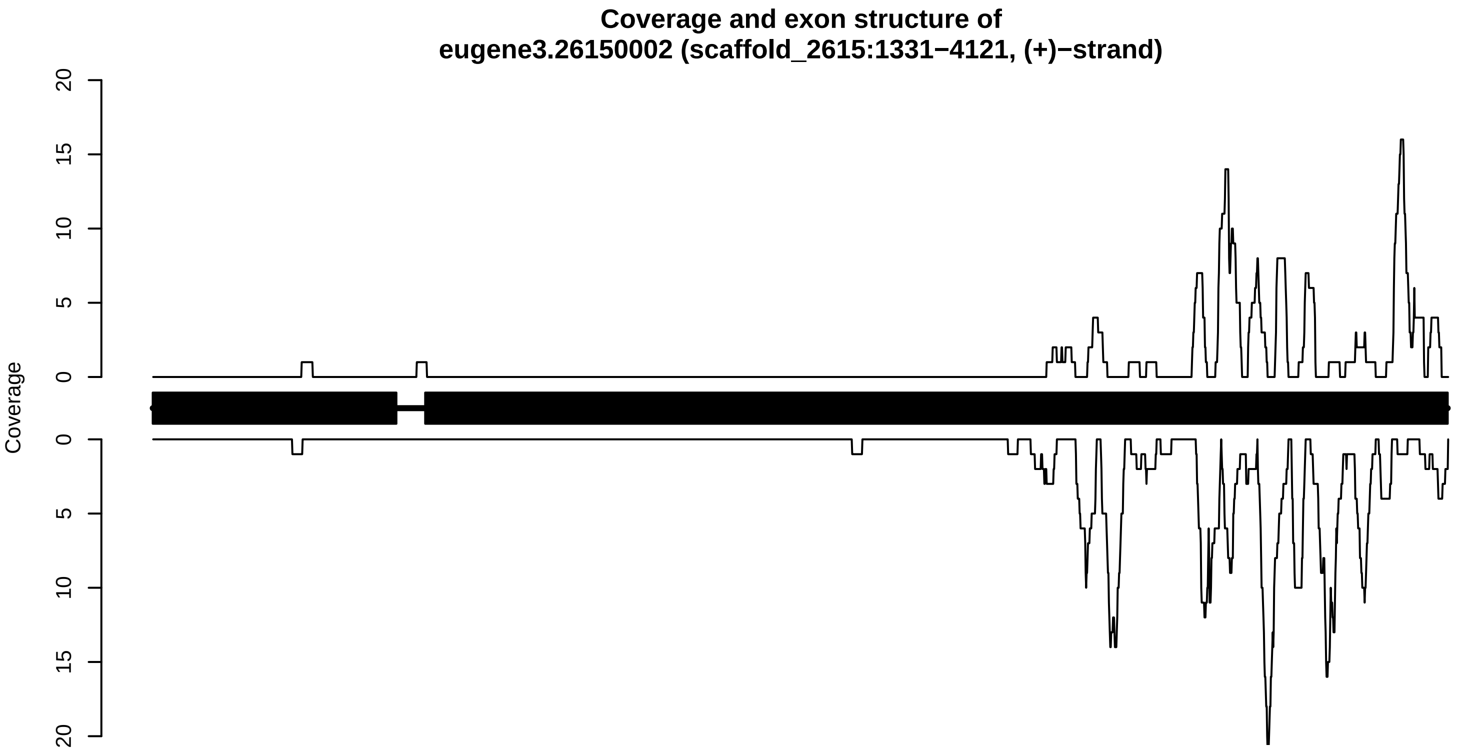

Coverage and exon structure of
eugene3.26150002 (scaffold_2615:1331−4121, (+)−strand)
Coverage
20
15
10
5
0
0
5
10
15
20

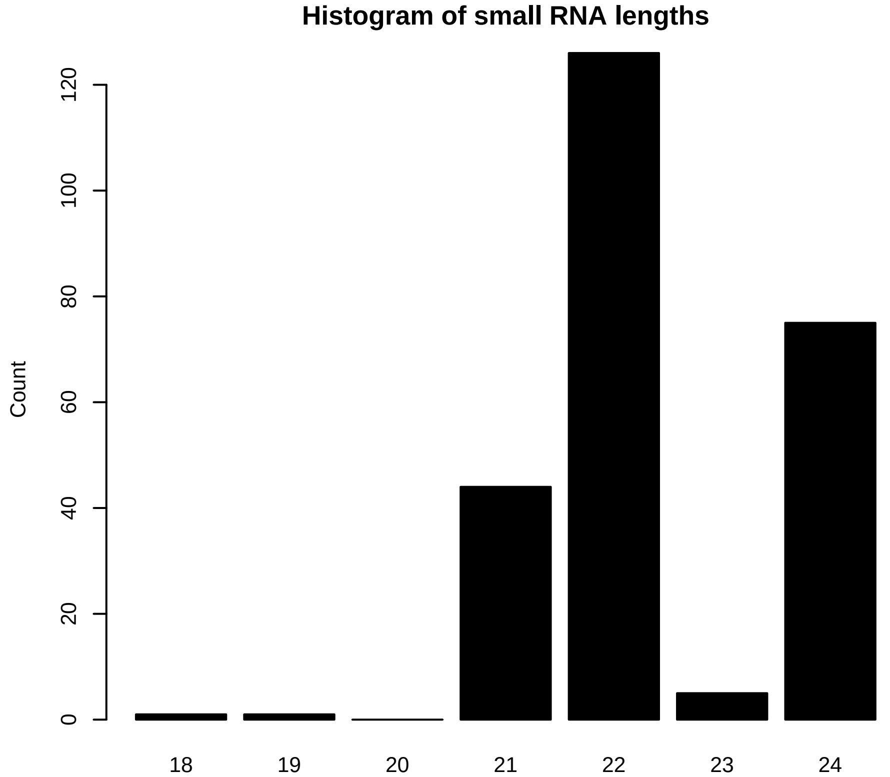

Histogram of small RNA lengths
Count
0
20
40
60
80
100
120
18
19
20
21
22
23
24

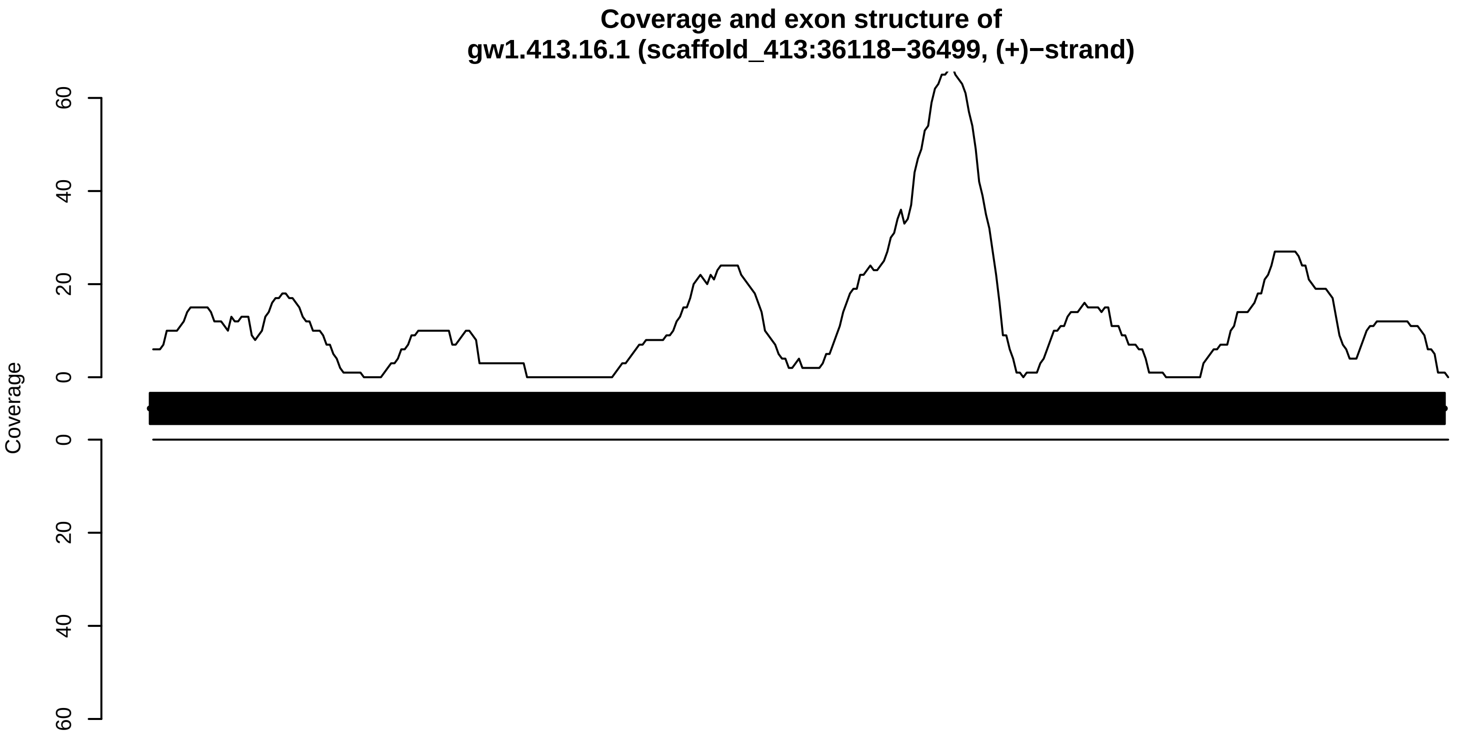
Coverage and exon structure of
gw1.413.16.1 (scaffold_413:36118−36499, (+)−strand)
Coverage
60
40
20
0
0
20
40
60

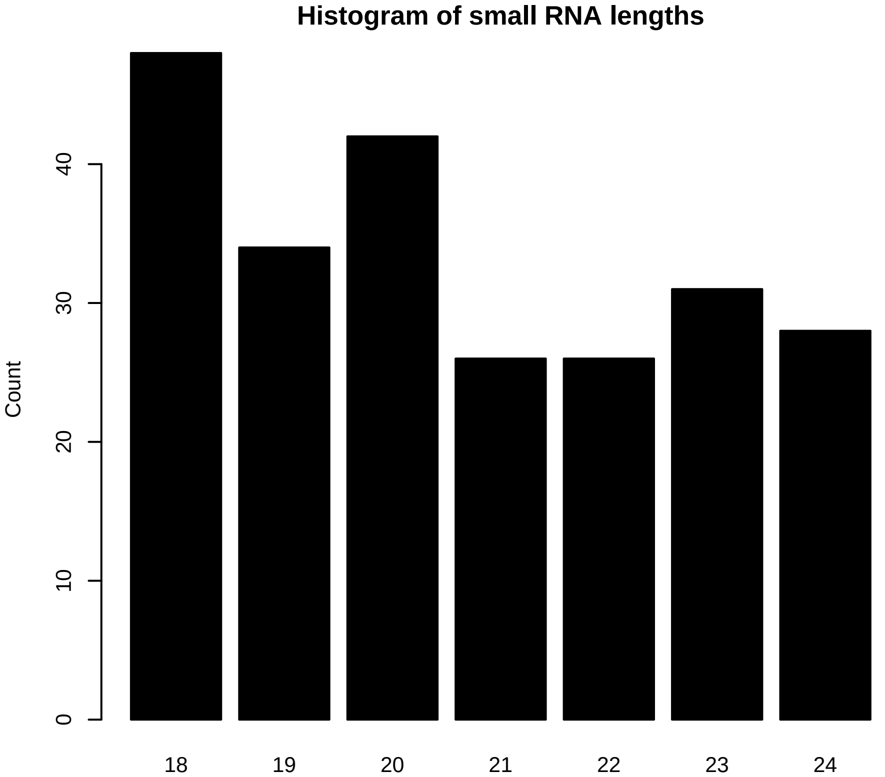
Histogram of small RNA lengths
Count
0
10
20
30
40
18
19
20
21
22
23
24

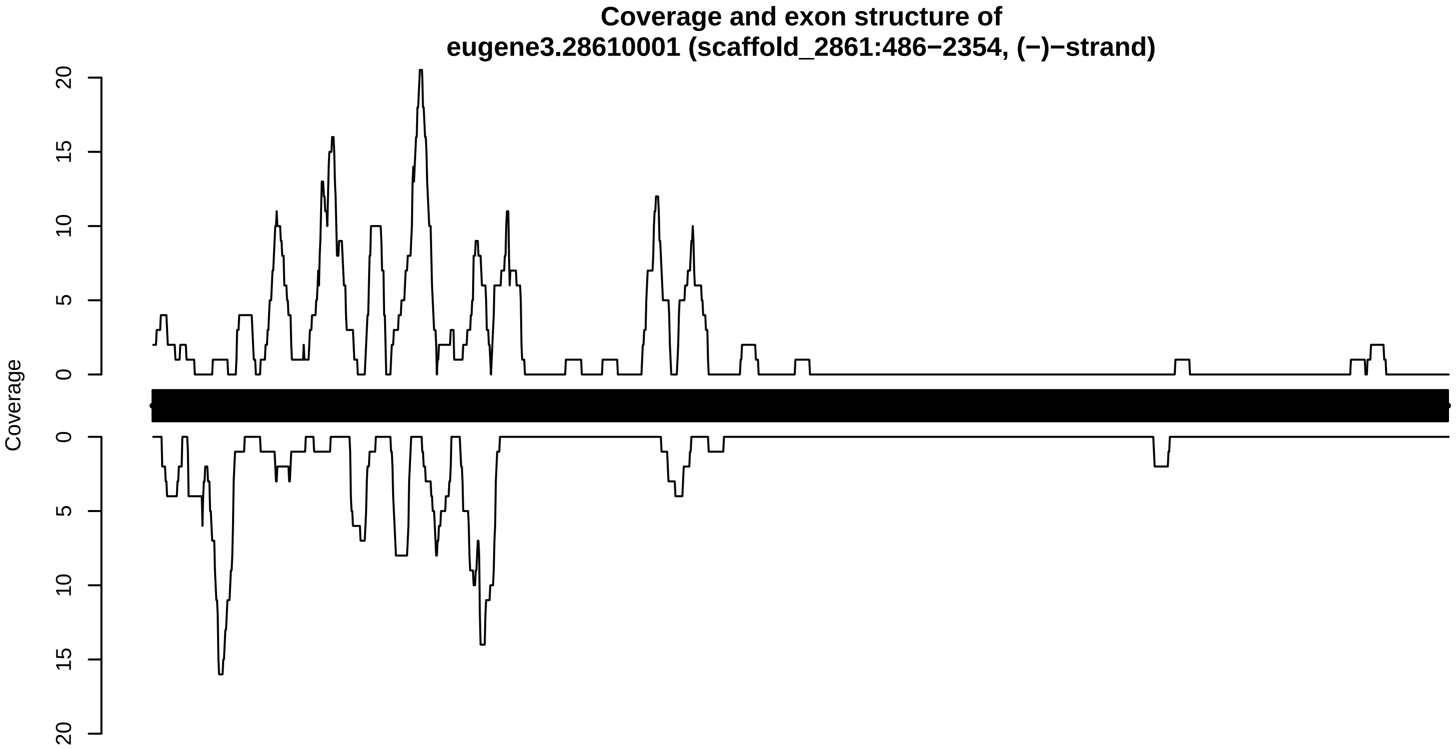
Coverage and exon structure of
eugene3.28610001 (scaffold_2861:486−2354, (−)−strand)
Coverage
20
15
10
5
0
0
5
10
15
20

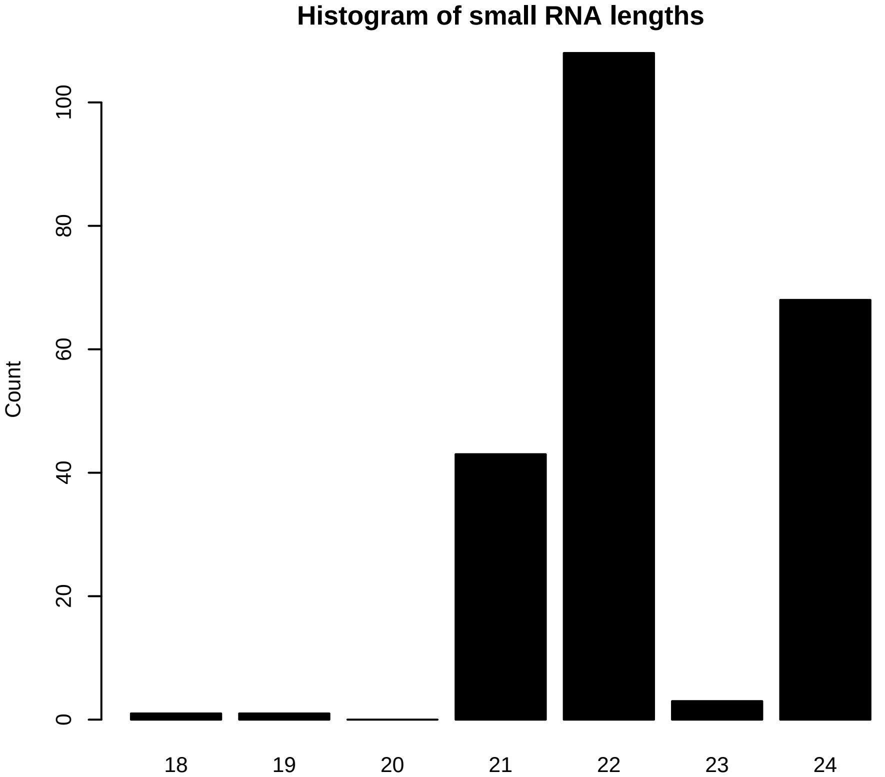
Histogram of small RNA lengths
Count
100
80
60
40
20
0
18
19
20
21
22
23
24

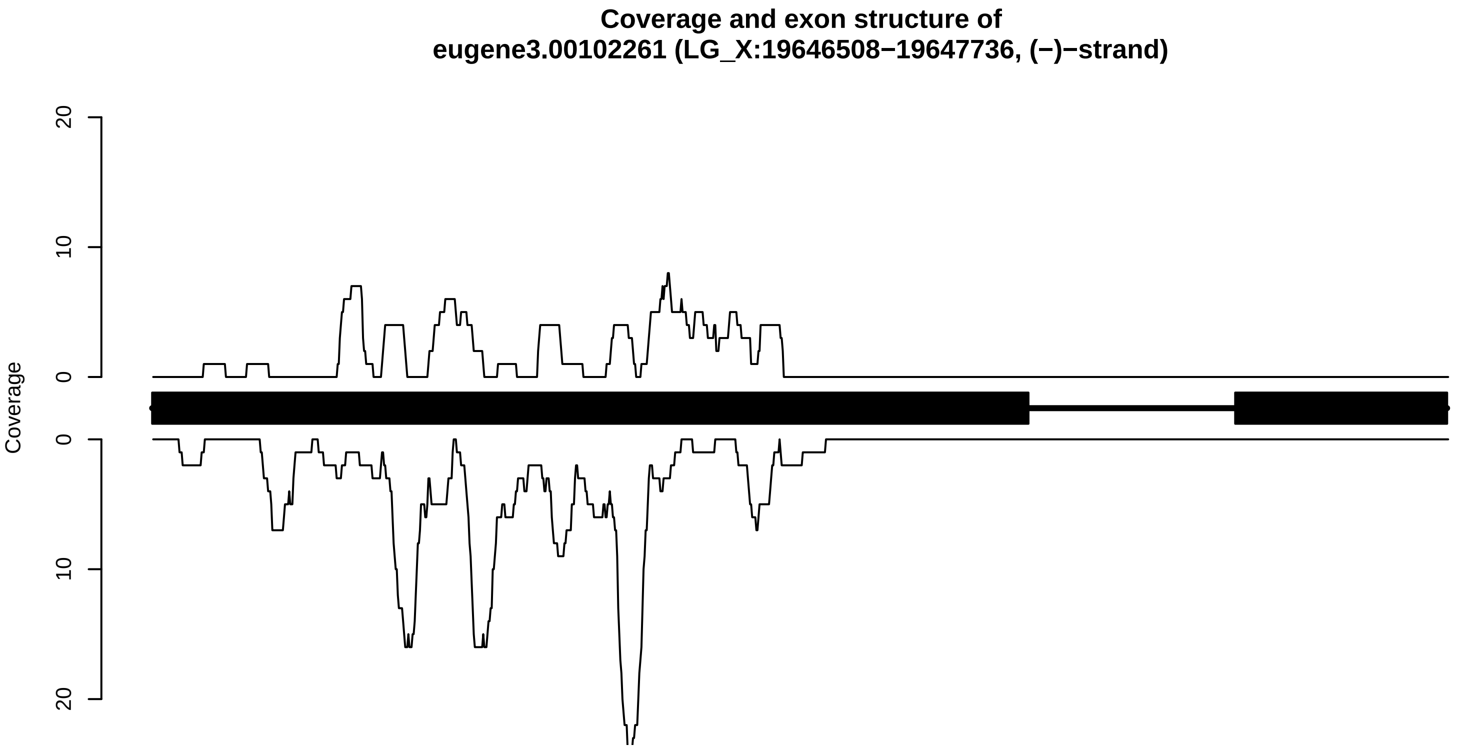

Coverage and exon structure of
eugene3.00102261 (LG_X:19646508−19647736, (−)−strand)
Coverage
20
10
0
0
10
20

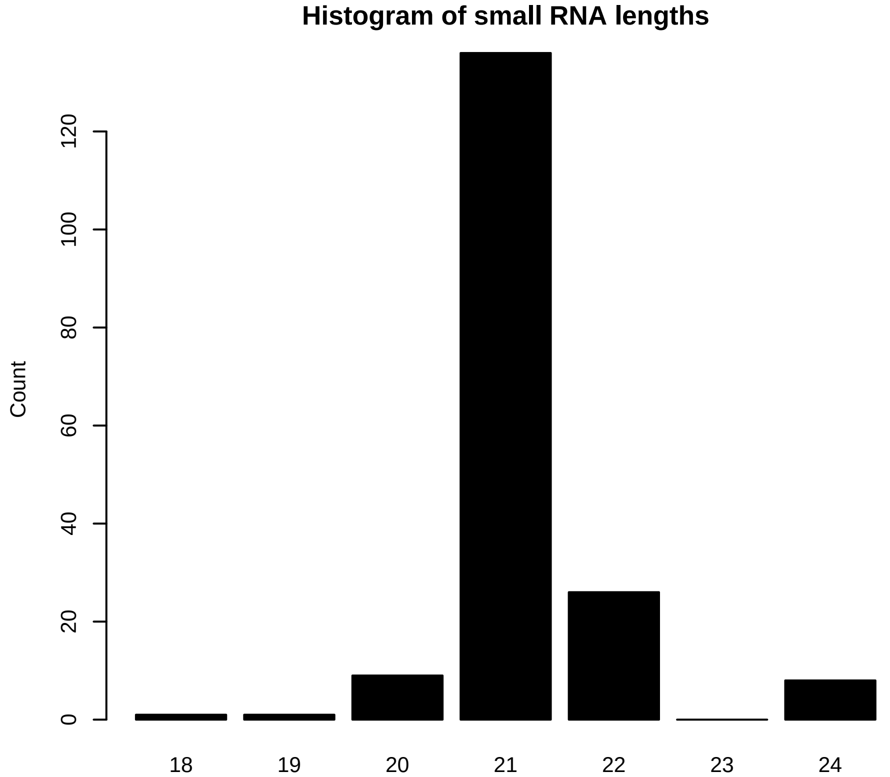

Histogram of small RNA lengths
Count
0
20
40
60
80
100
120
18
19
20
21
22
23
24

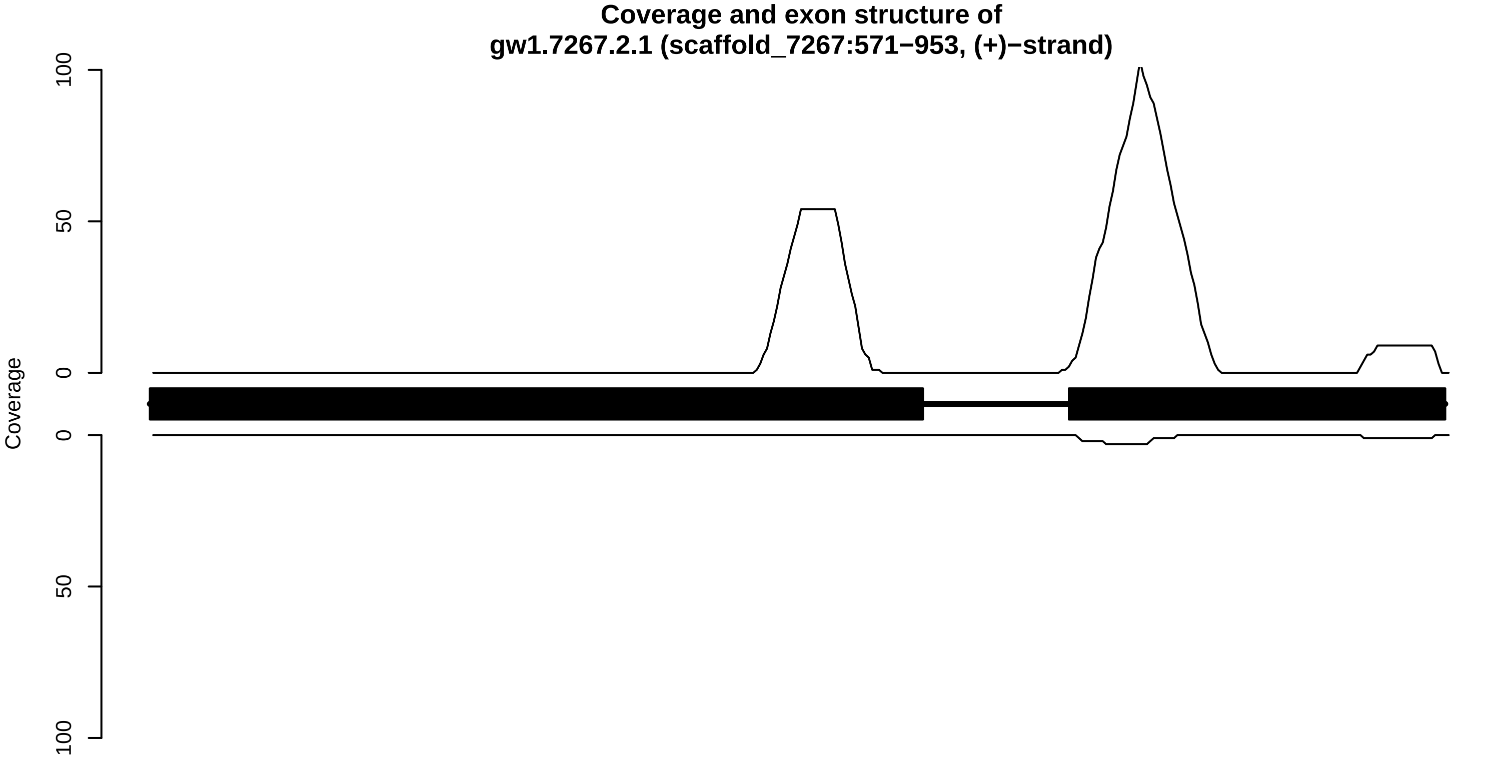

Coverage and exon structure of
gw1.7267.2.1 (scaffold_7267:571−953, (+)−strand)
Coverage
100
50
0
0
50
100

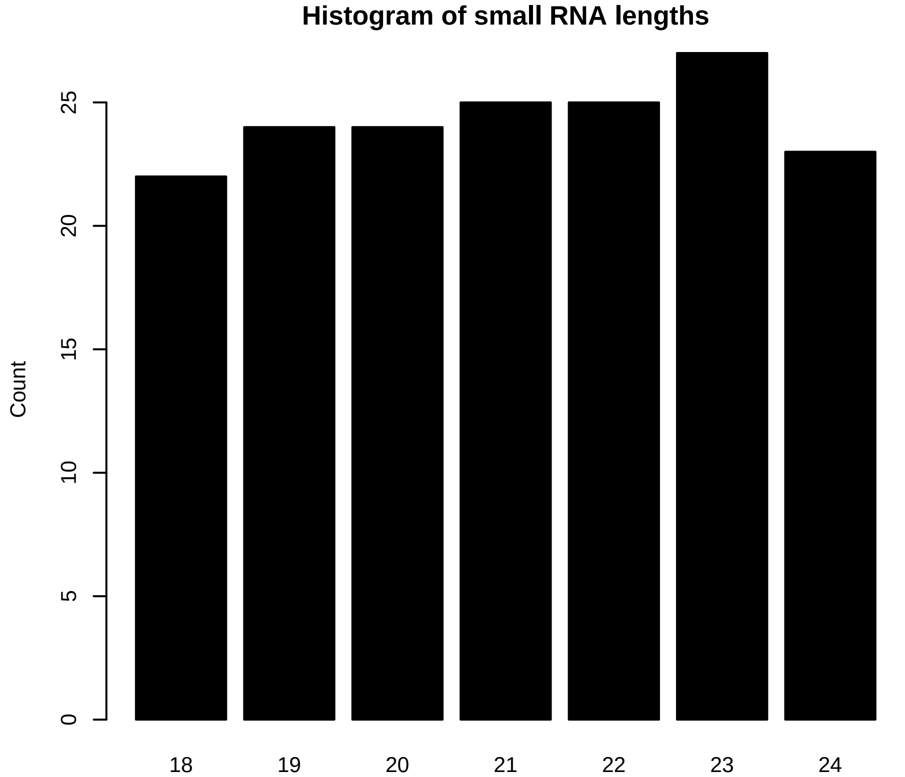

Histogram of small RNA lengths
Count
0
5
10
15
20
25
18
19
20
21
22
23
24

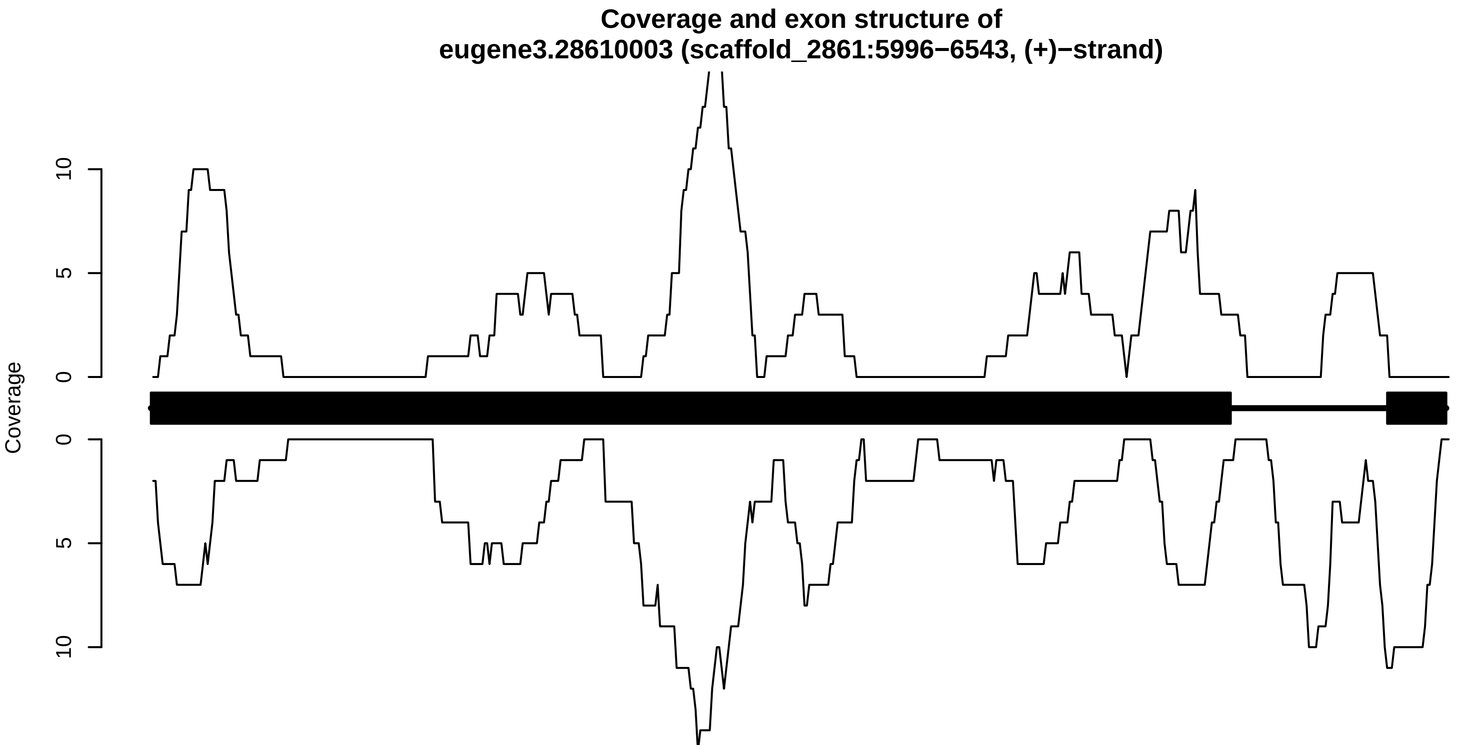
Coverage and exon structure of
eugene3.28610003 (scaffold_2861:5996−6543, (+)−strand)
Coverage
10
5
0
0
5
10
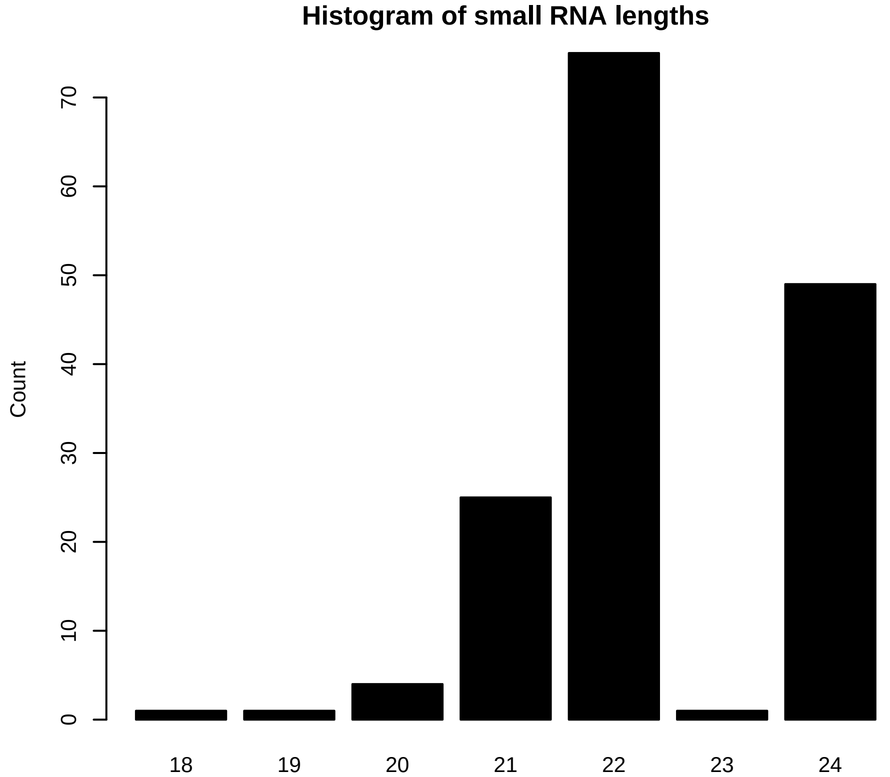
Histogram of small RNA lengths
Count
70
60
50
40
30
20
10
0
18
19
20
21
22
23
24

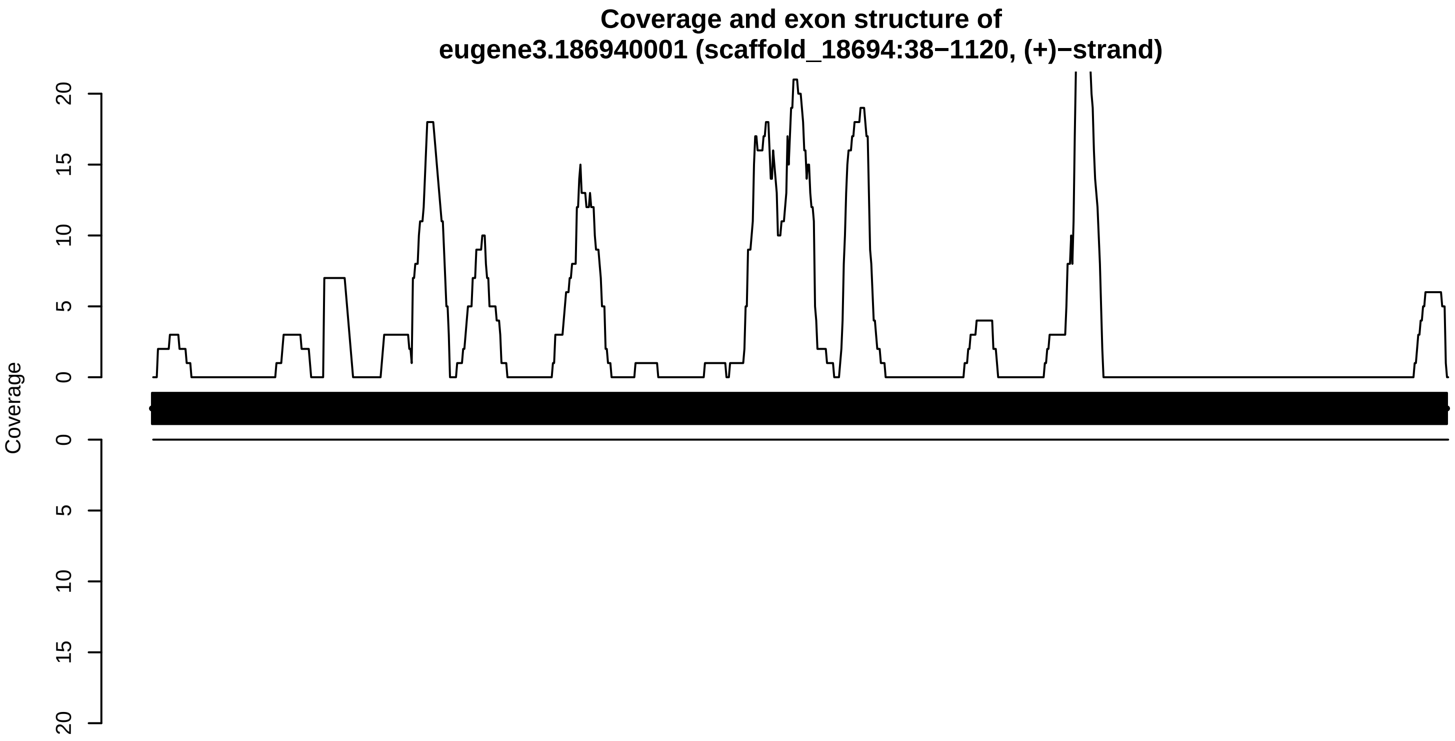

Coverage and exon structure of
eugene3.186940001 (scaffold_18694:38−1120, (+)−strand)
Coverage
0
5
10
15
20
0
5
10
15
20

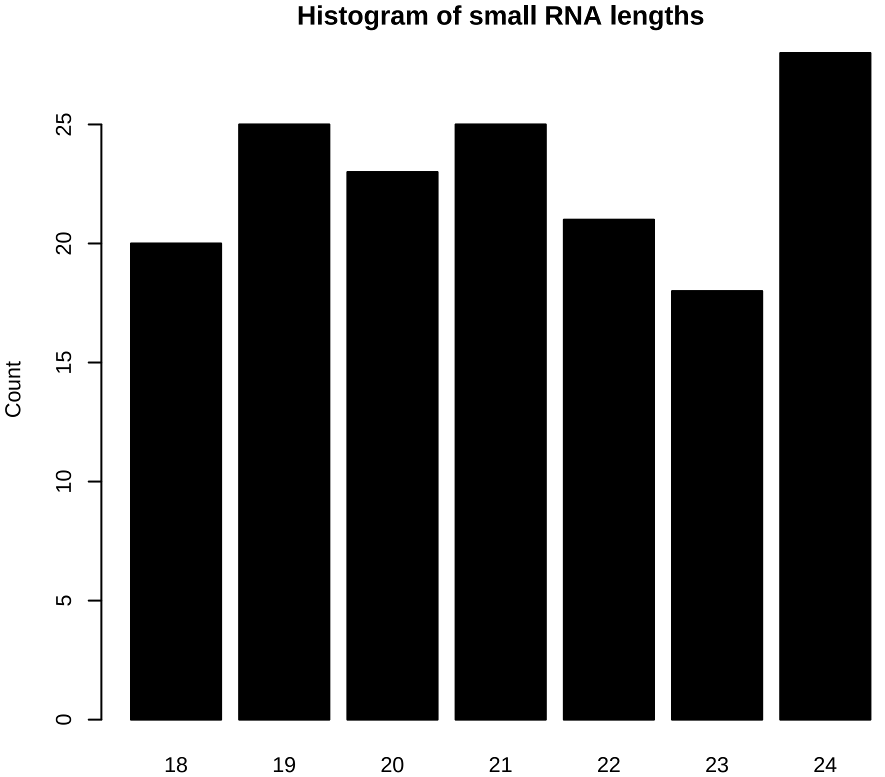

Histogram of small RNA lengths
Count
0
5
10
15
20
25
18
19
20
21
22
23
24

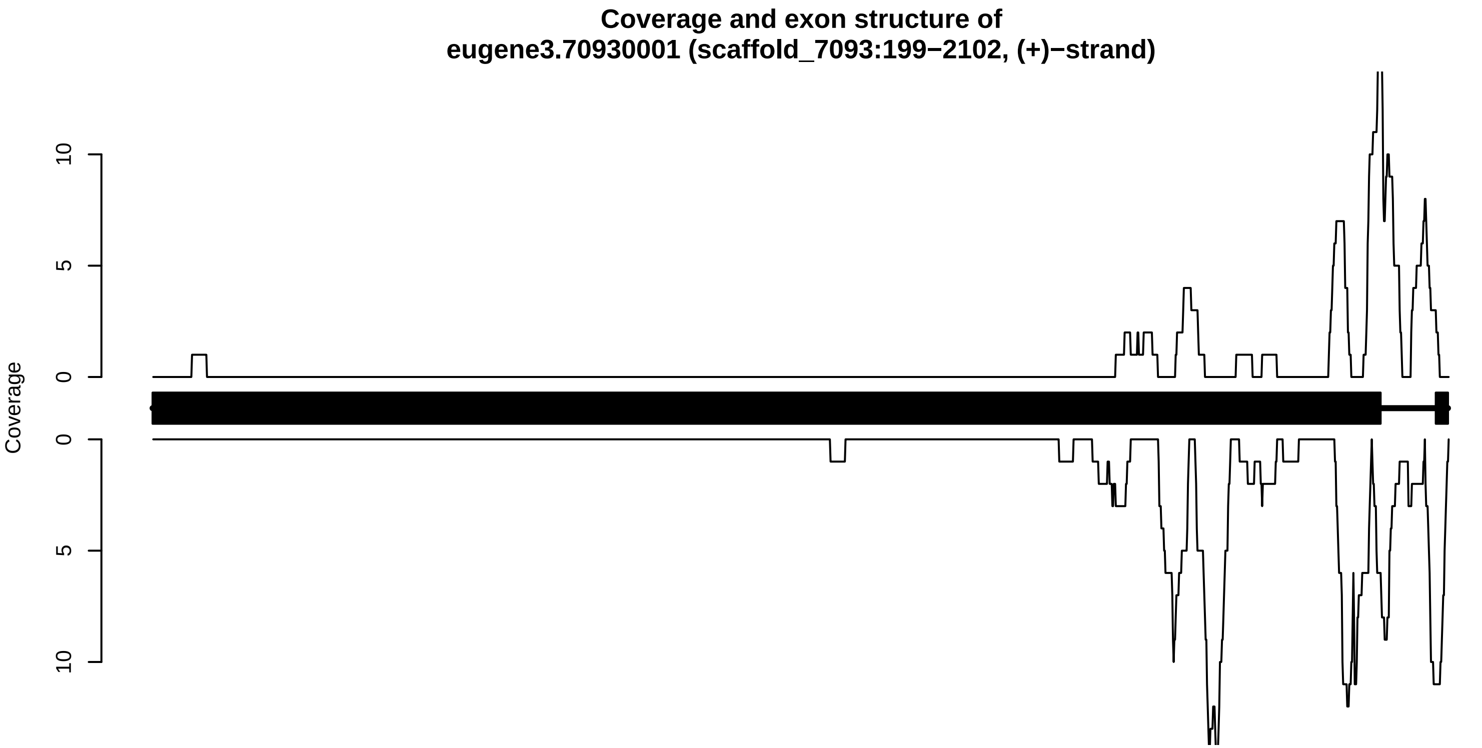

Coverage and exon structure of
eugene3.70930001 (scaffold_7093:199−2102, (+)−strand)
Coverage
0
5
10
0
5
10

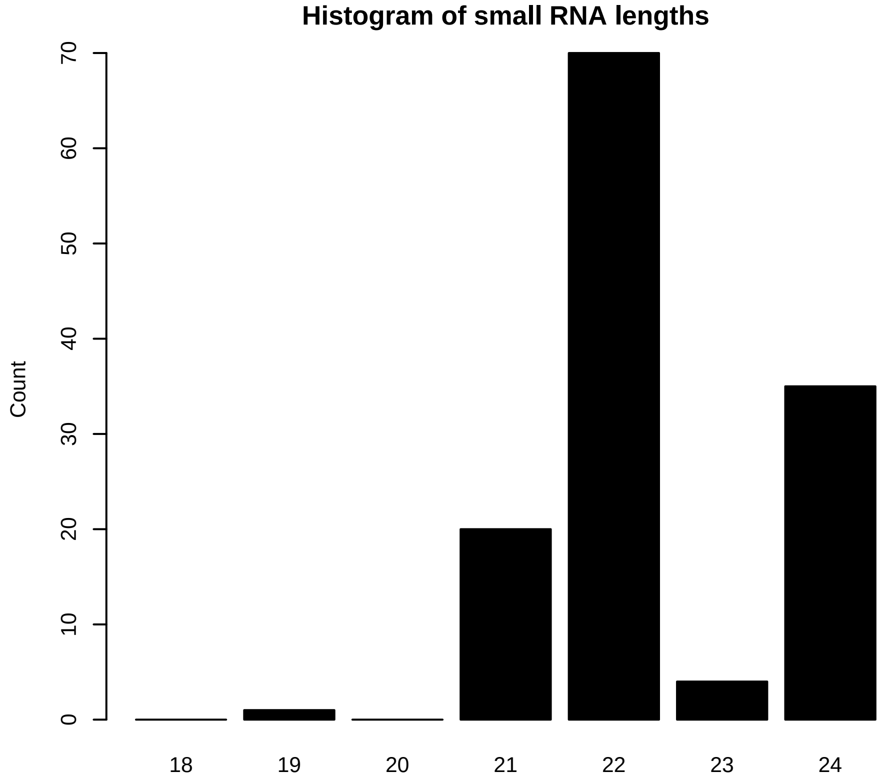

Histogram of small RNA lengths
Count
0
10
20
30
40
50
60
70
18
19
20
21
22
23
24

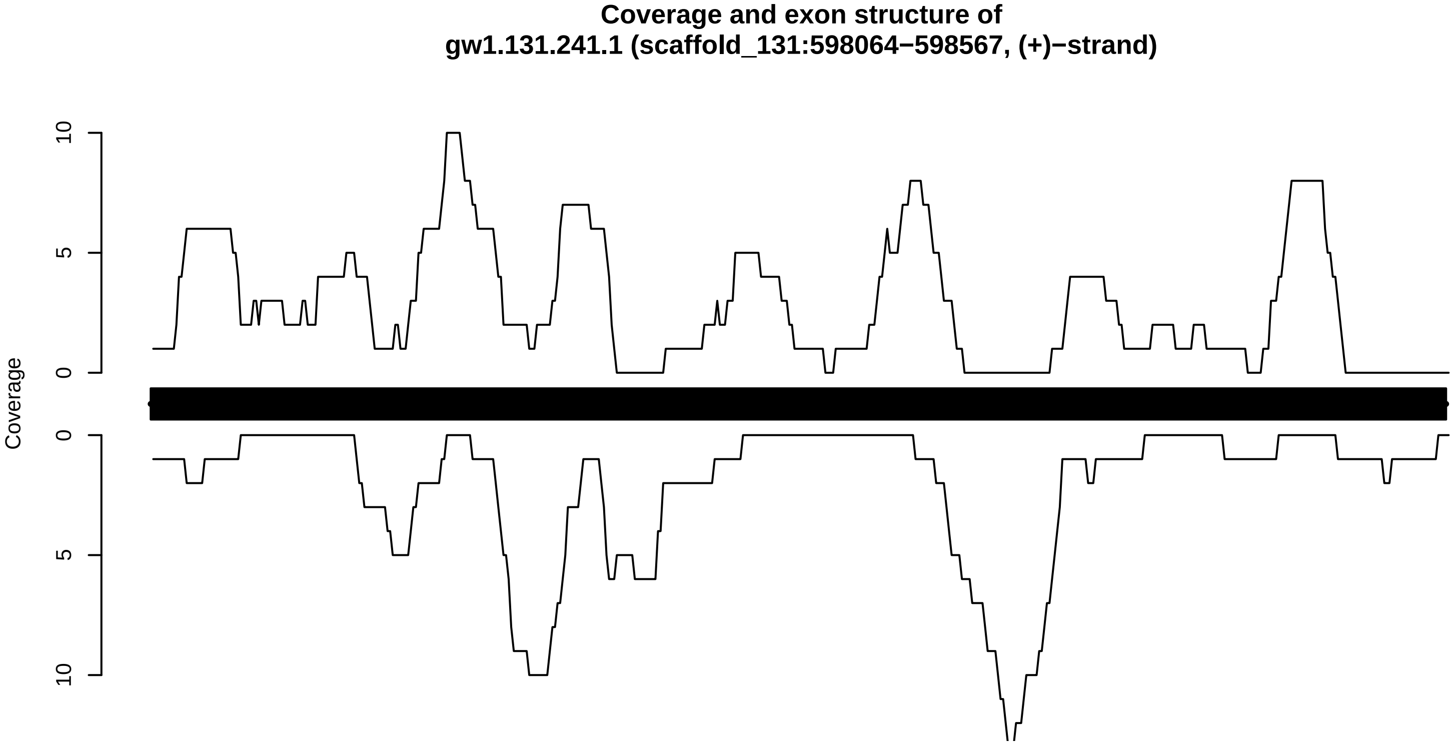
Coverage and exon structure of
gw1.131.241.1 (scaffold_131:598064−598567, (+)−strand)
Coverage
10
5
0
0
5
10

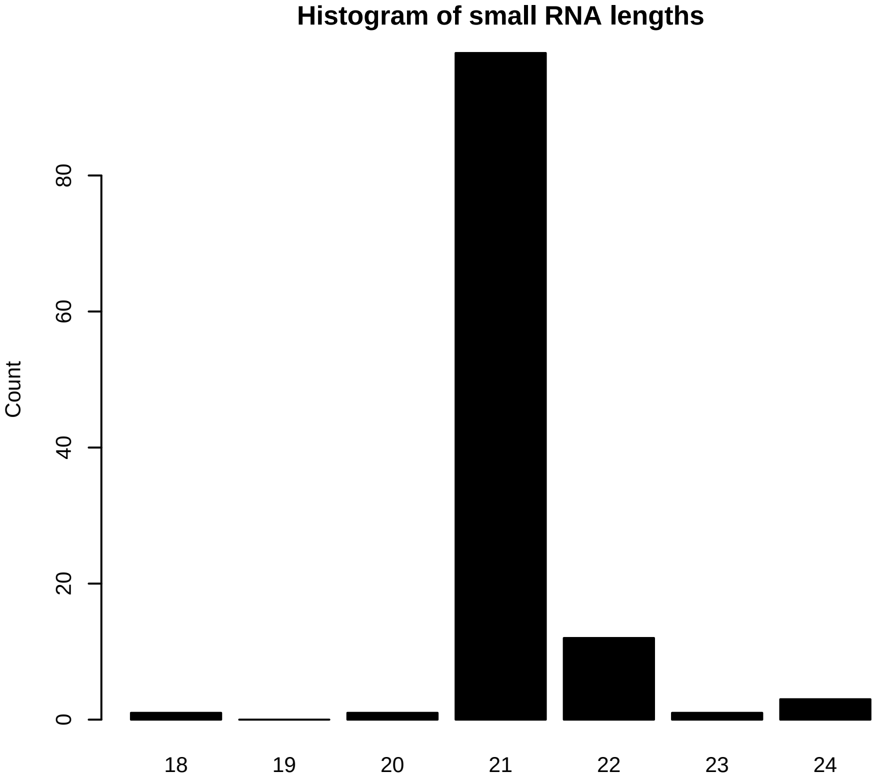
Histogram of small RNA lengths
Count
80
60
40
20
0
18
19
20
21
22
23
24

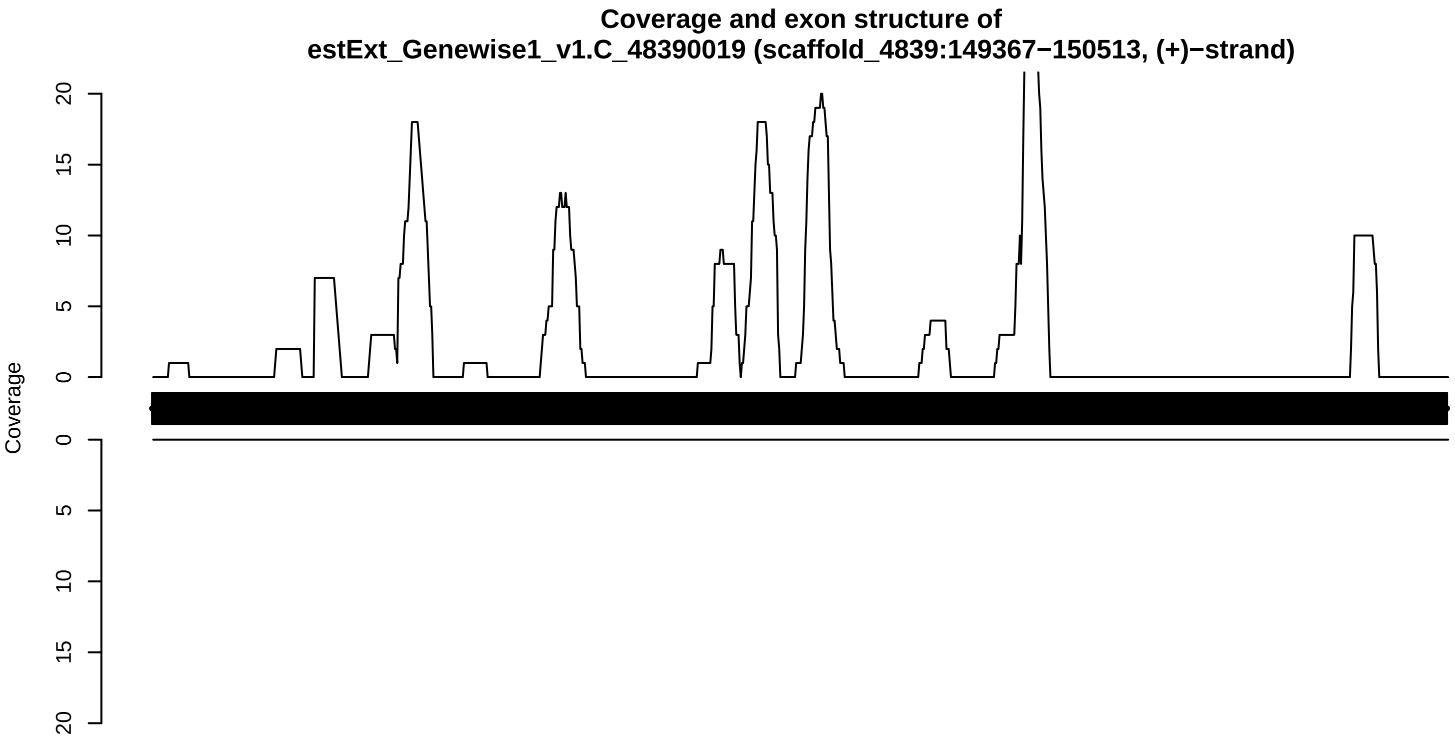
Coverage and exon structure of
estExt_Genewise1_v1.C_48390019 (scaffold_4839:149367−150513, (+)−strand)
Coverage
20
15
10
5
0
0
5
10
15
20

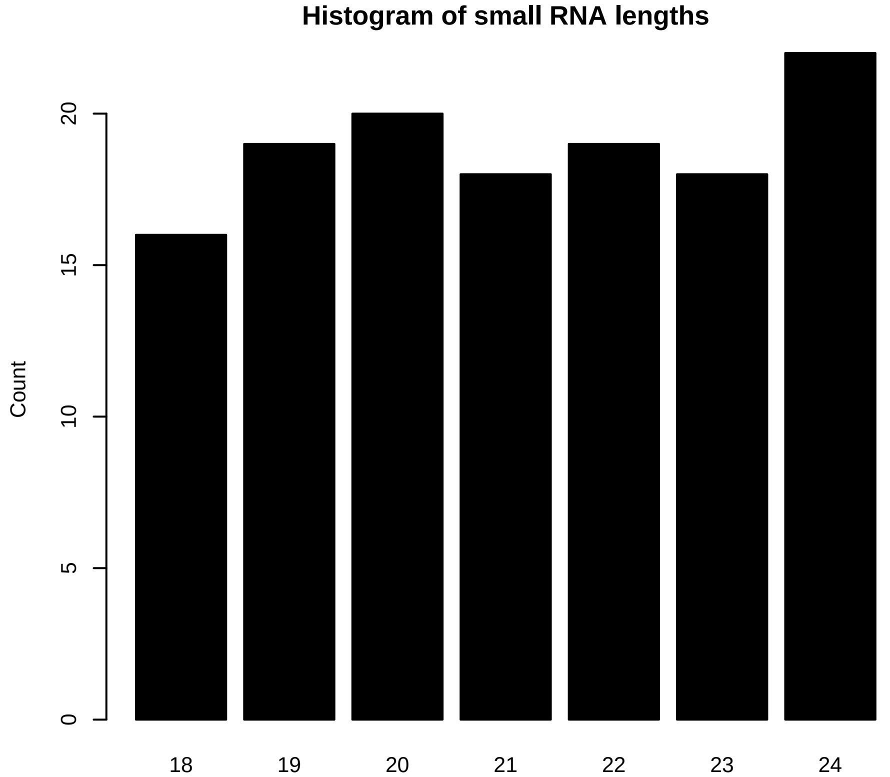
Histogram of small RNA lengths
Count
0
5
10
15
20
18
19
20
21
22
23
24

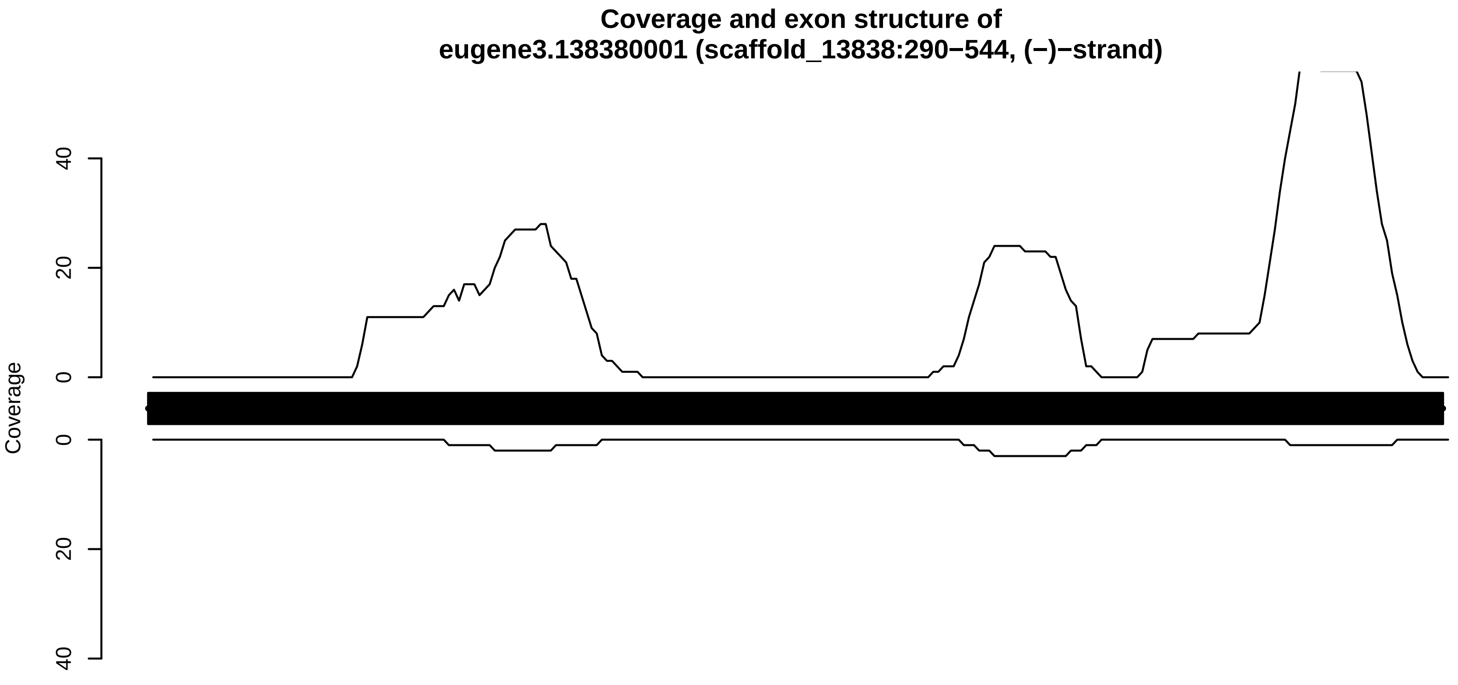
Coverage and exon structure of
eugene3.138380001 (scaffold_13838:290−544, (−)−strand)
Coverage
40
20
0
0
20
40

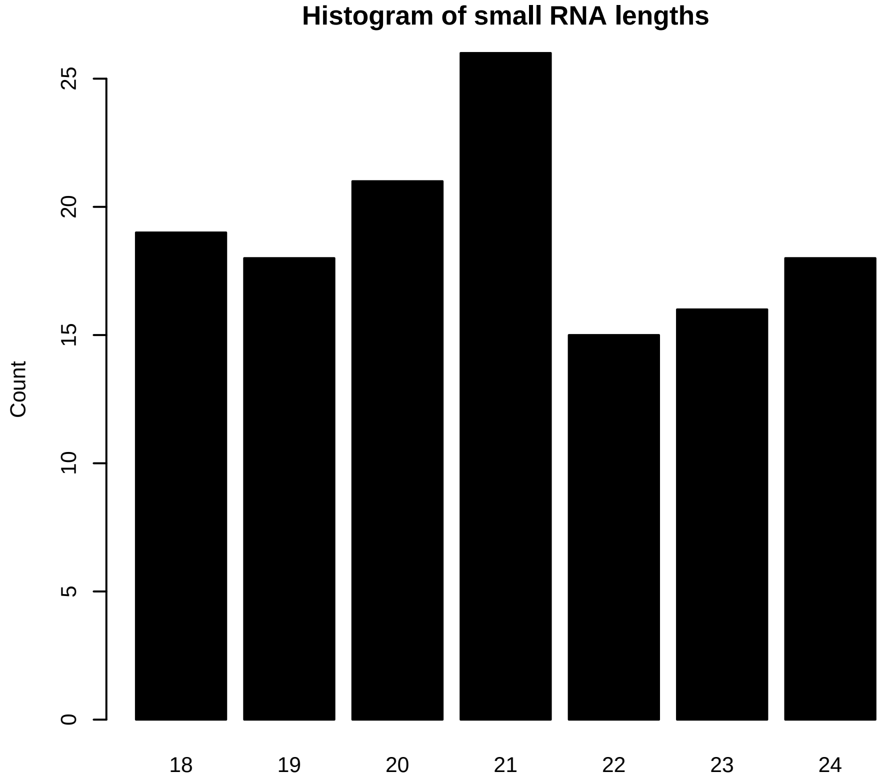
Histogram of small RNA lengths
Count
0
5
10
15
20
25
18
19
20
21
22
23
24

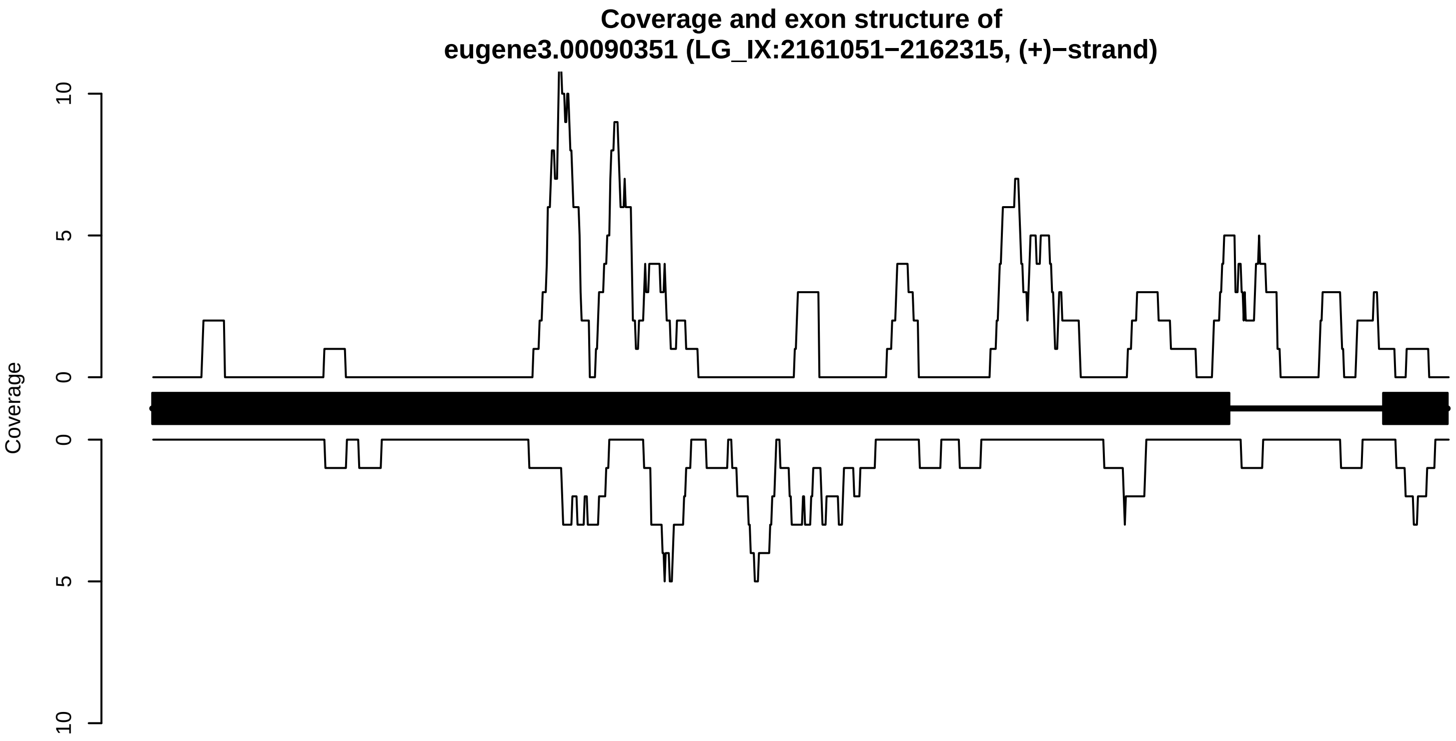
Coverage and exon structure of
eugene3.00090351 (LG_IX:2161051−2162315, (+)−strand)
Coverage
10
5
0
0
5
10

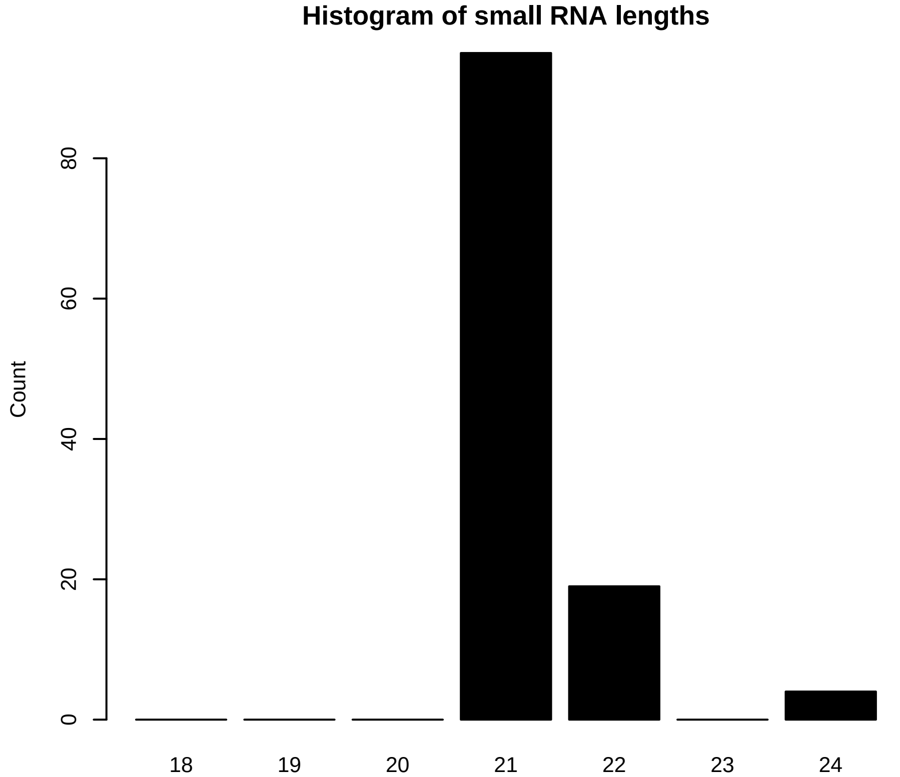
Histogram of small RNA lengths
Count
0
20
40
60
80
18
19
20
21
22
23
24

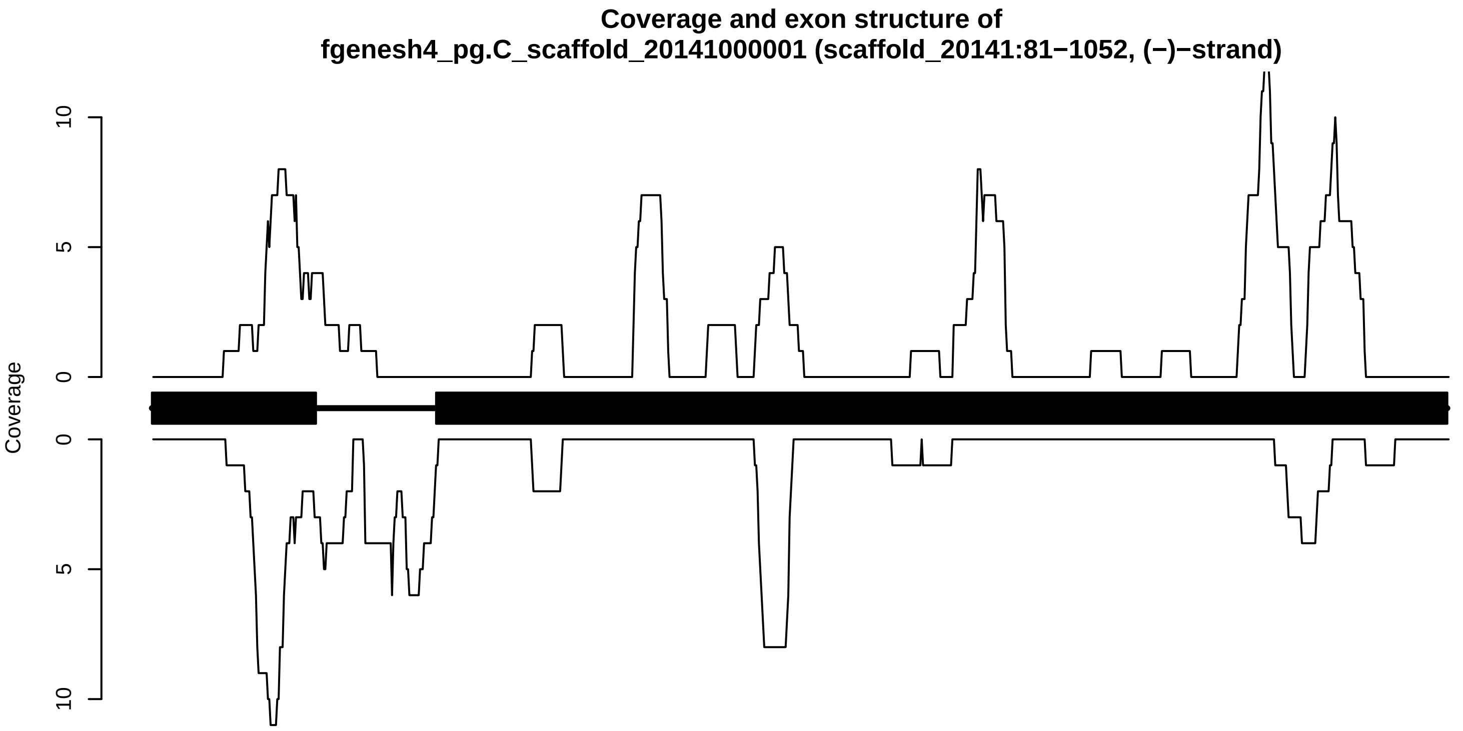
Coverage and exon structure of
fgenesh4_pg.C_scaffold_20141000001 (scaffold_20141:81−1052, (−)−strand)
Coverage
0
5
10
0
5
10

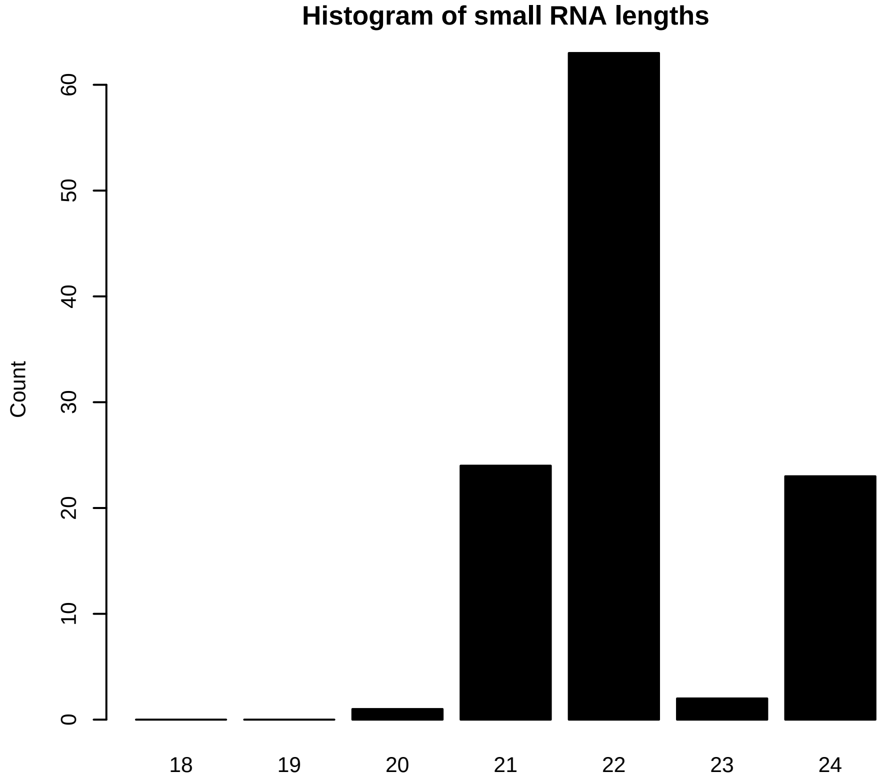
Histogram of small RNA lengths
Count
0
10
20
30
40
50
60
18
19
20
21
22
23
24

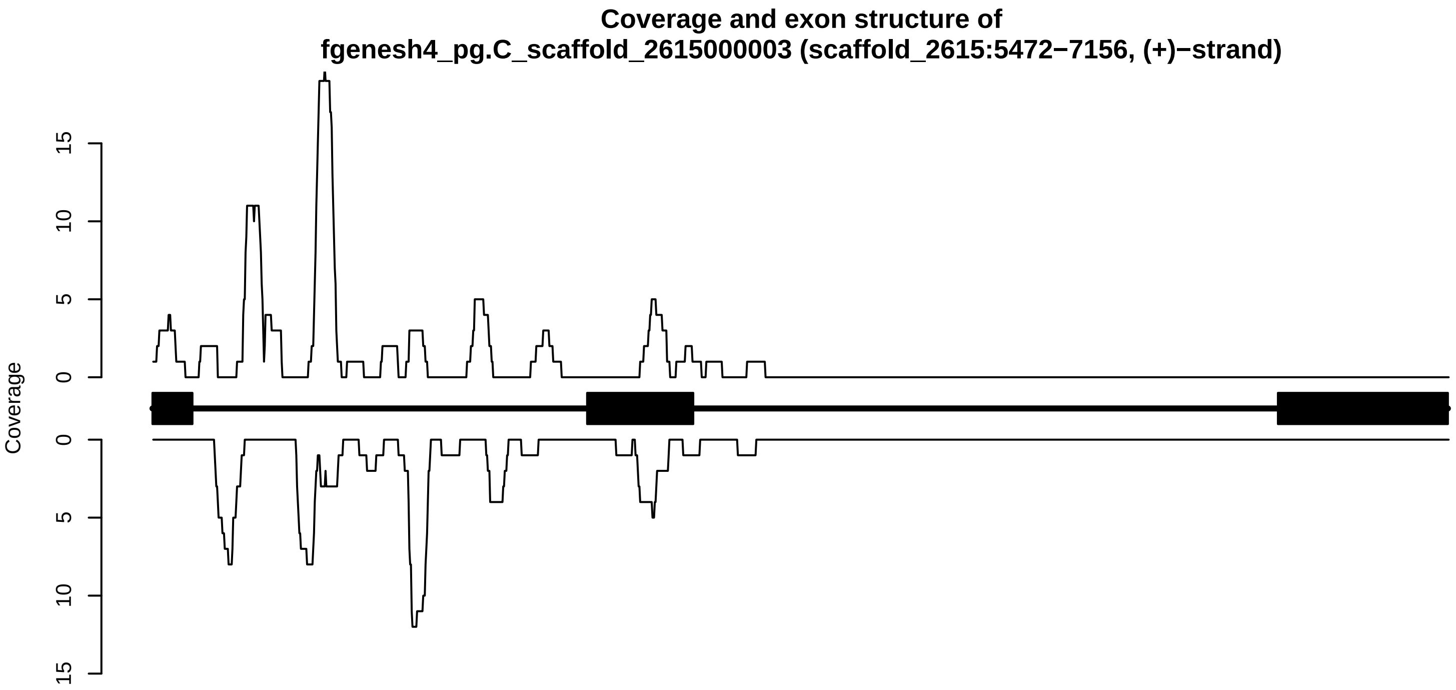

Coverage and exon structure of
fgenesh4_pg.C_scaffold_2615000003 (scaffold_2615:5472−7156, (+)−strand)
Coverage
0
5
10
15

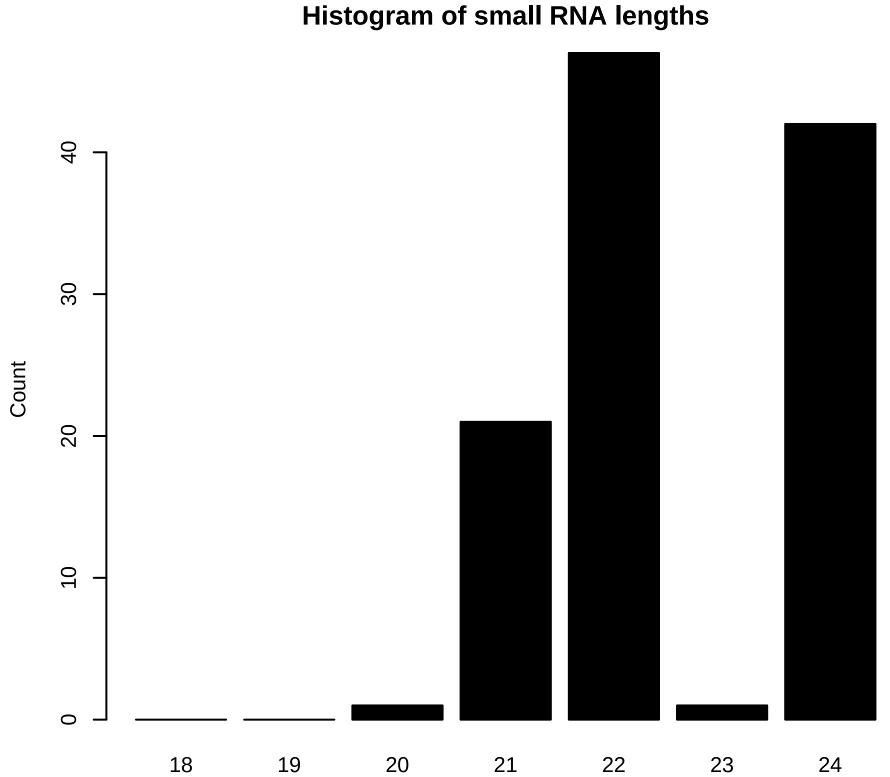

Histogram of small RNA lengths
Count
0
10
20
30
40
18
19
20
21
22
23
24

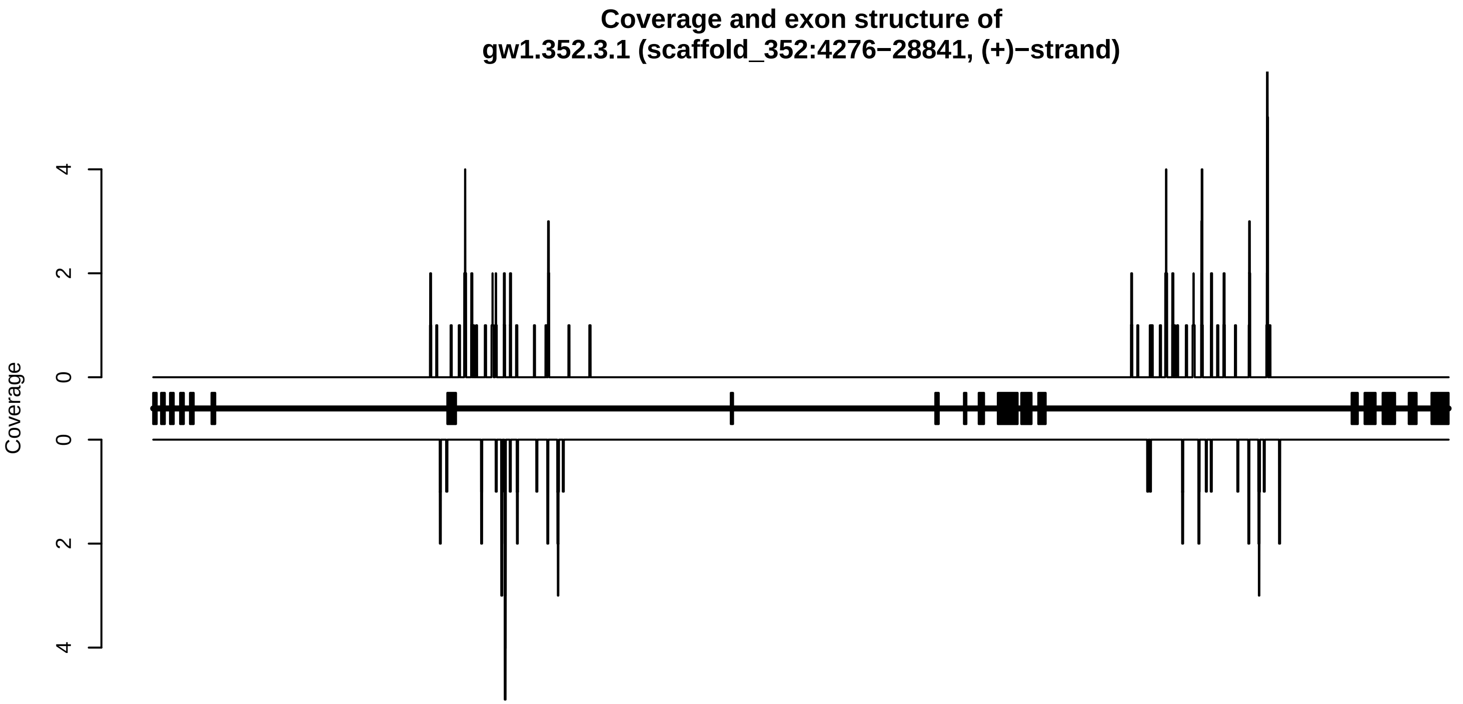

Coverage and exon structure of
gw1.352.3.1 (scaffold_352:4276−28841, (+)−strand)
Coverage
4
2
0
0
2
4

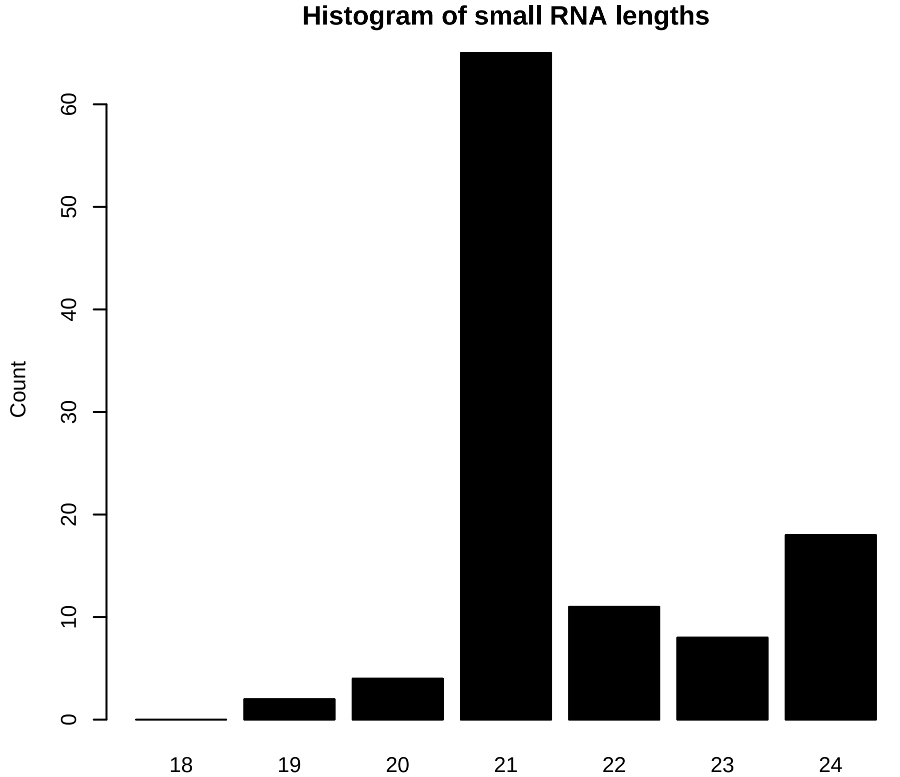

Histogram of small RNA lengths
Count
0
10
20
30
40
50
60
18
19
20
21
22
23
24

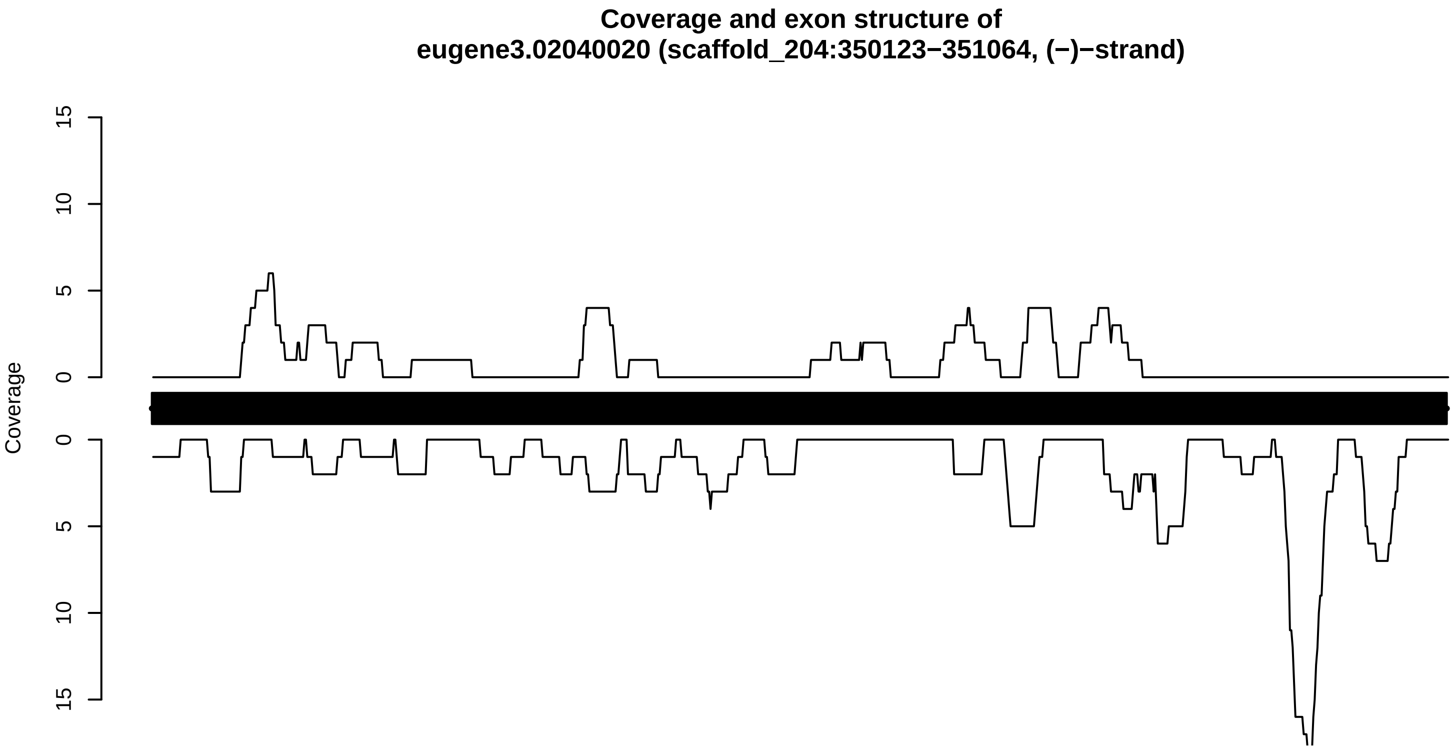

Coverage and exon structure of
eugene3.02040020 (scaffold_204:350123−351064, (−)−strand)
Coverage
15
10
5
0
0
5
10
15

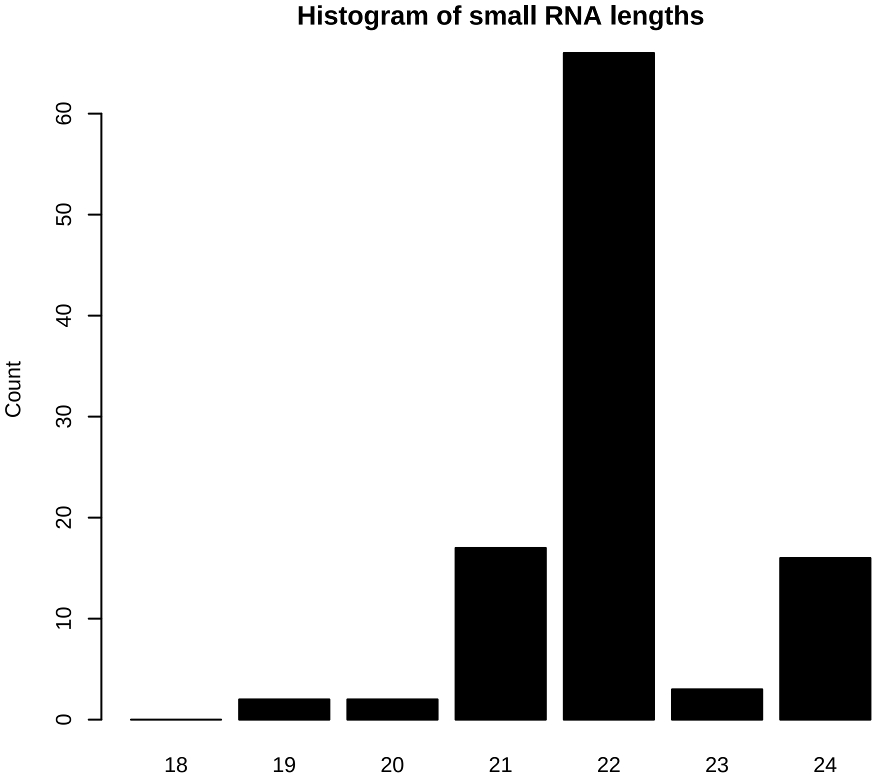

Histogram of small RNA lengths
Count
0
10
20
30
40
50
60
18
19
20
21
22
23
24
