# Supplementary figures and images for: Genome-wide profiling of Populus small RNAs
Source: BMC Genomics. 2009 Dec 20;10:620. doi: 10.1186/1471-2164-10-620 (PMC2811130; doi:10.1186/1471-2164-10-620)

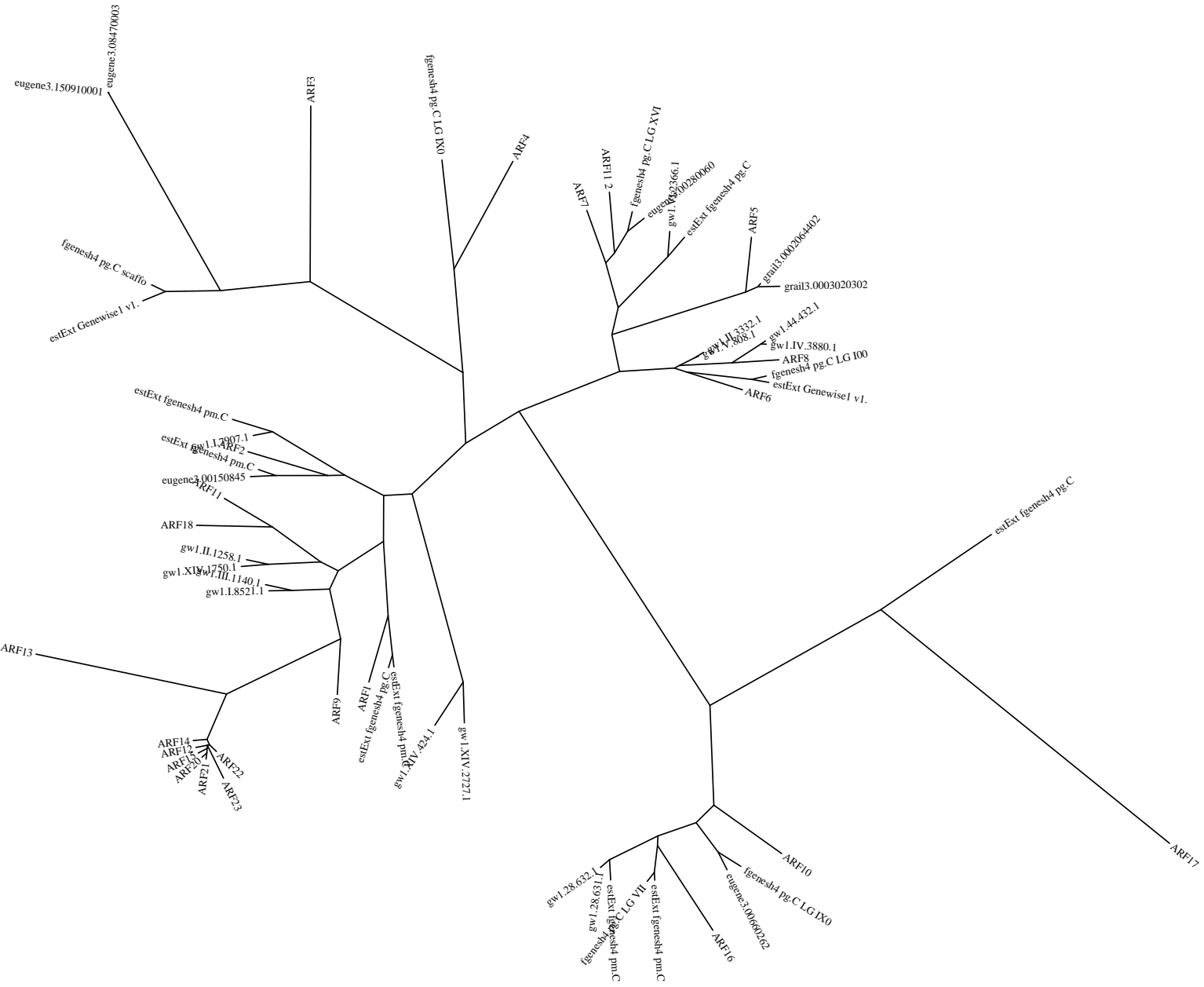

Supplement: Additional file 5 — Phylogenetic tree of predicted ARF gene family members. Phylogenetic tree of predicted members of the ARF gene family. Family members were identified using an HMM model search for the presence of the ARF domain within the Jamboree gene model set. Phylogenetic tree was used using [57]. [file 1471-2164-10-620-S5.PDF]

LG\_II:20949418..20949541

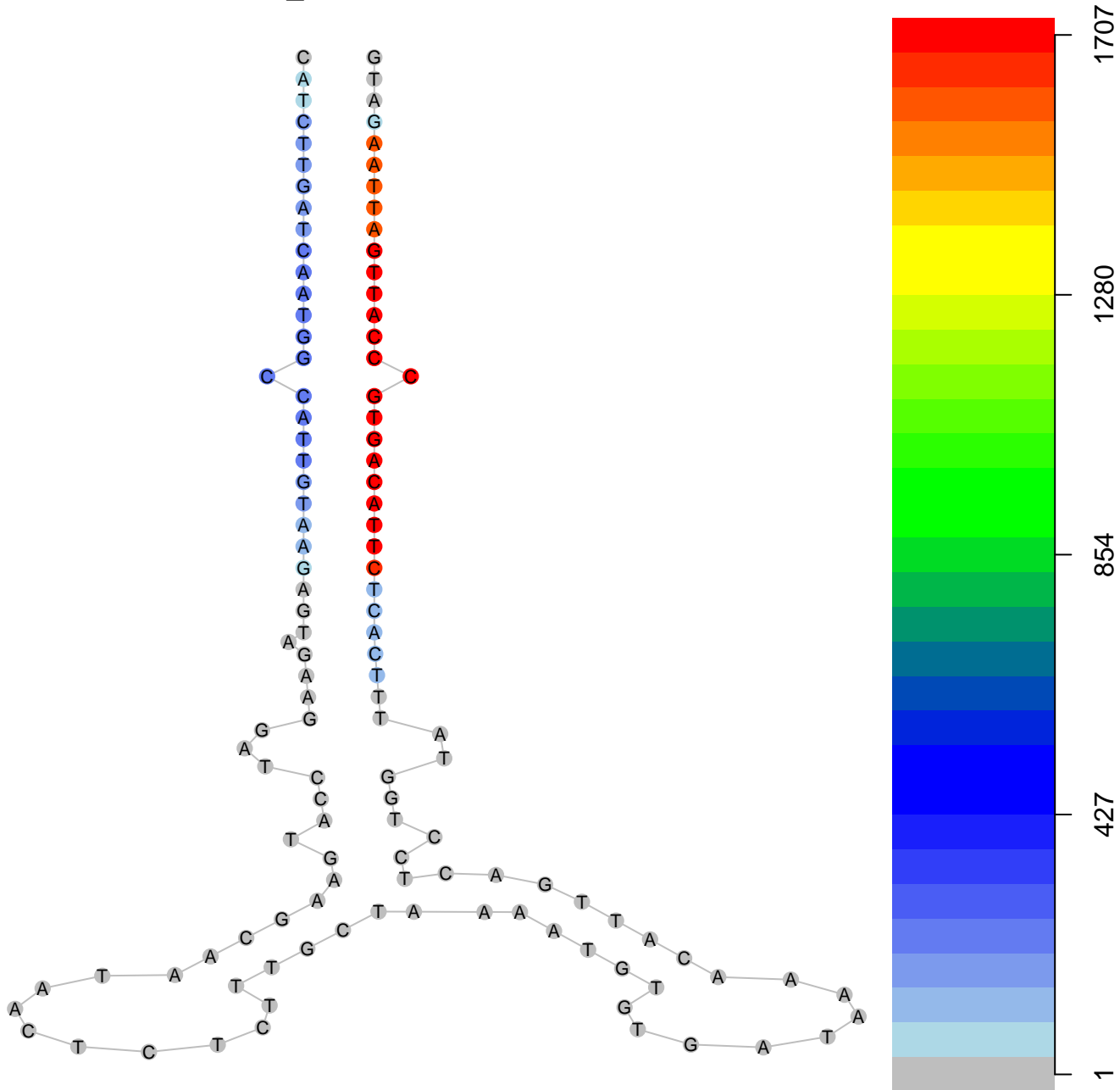

LG\_VIII:13883556..13883675

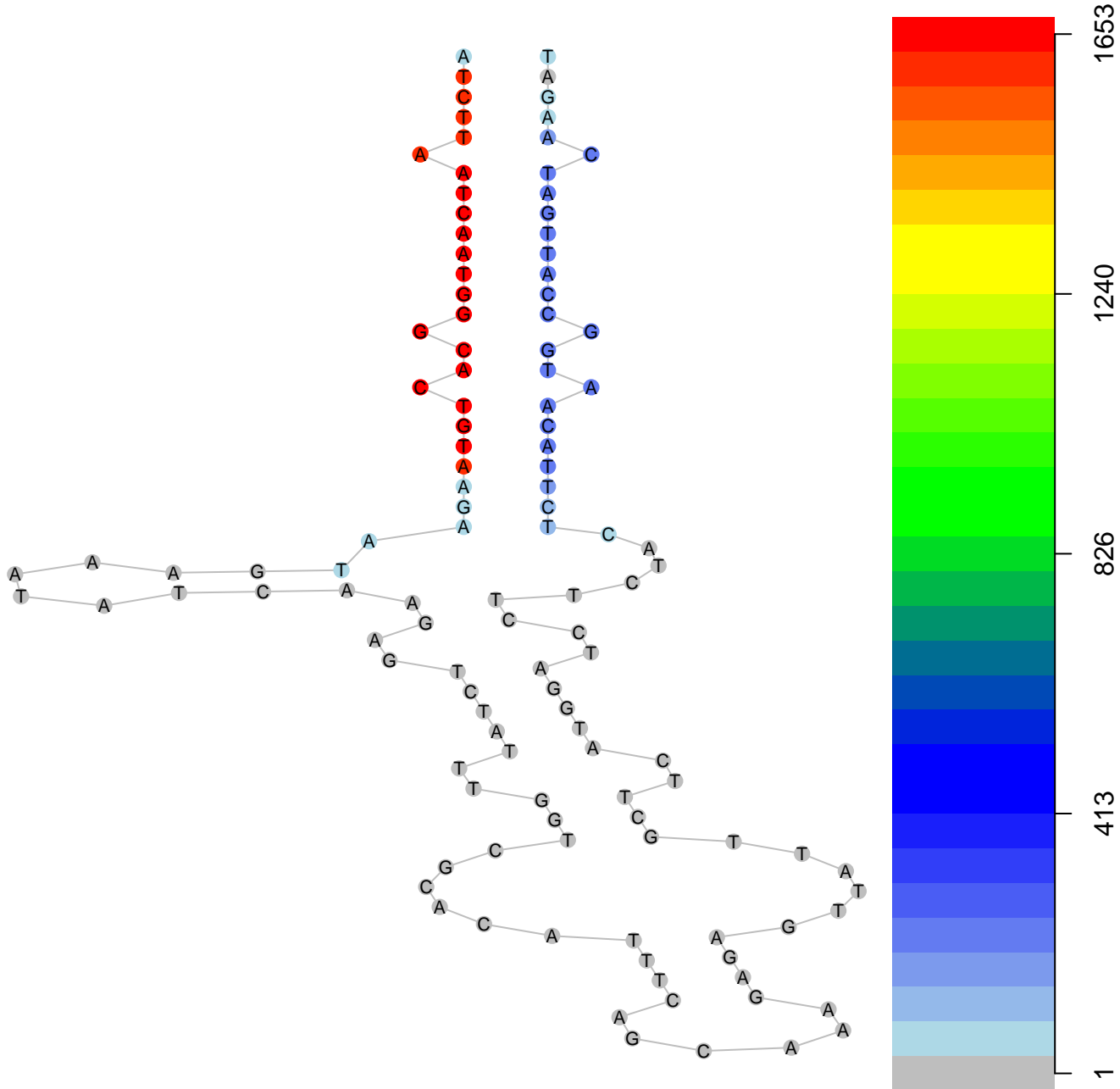

LG\_X:12045765..12045889

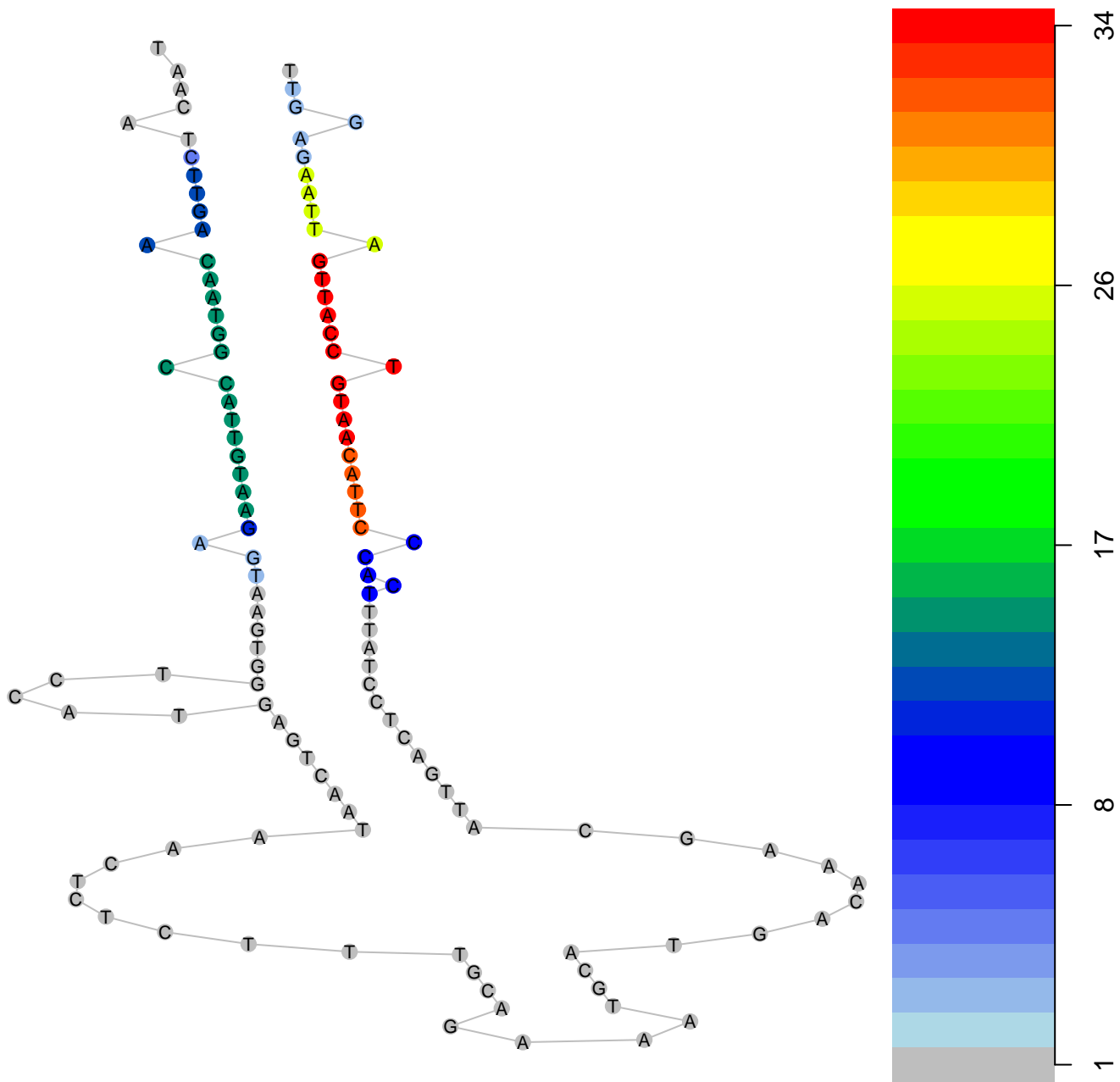

# LG\_VIII:8026870..8026972

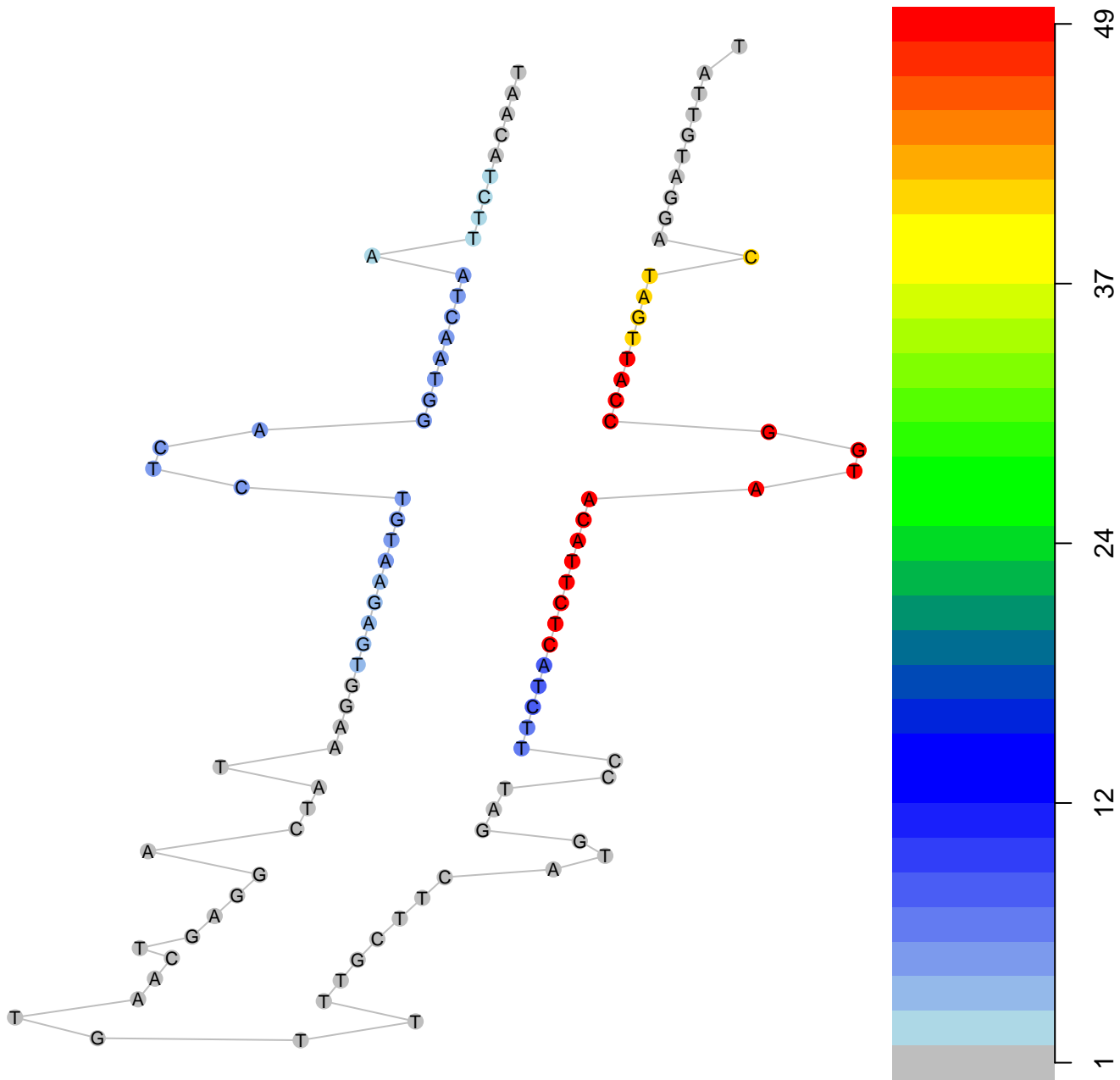

Supplement: Additional file 7 — Hairpin structure of miR475. Diagrammatic representation of the predicted stem-loop hairpin structure of miR475. The read count of sequences along the sequence is indicated using coloured shading. [file 1471-2164-10-620-S7.PDF]

LG\_XIX:11339581..11339676

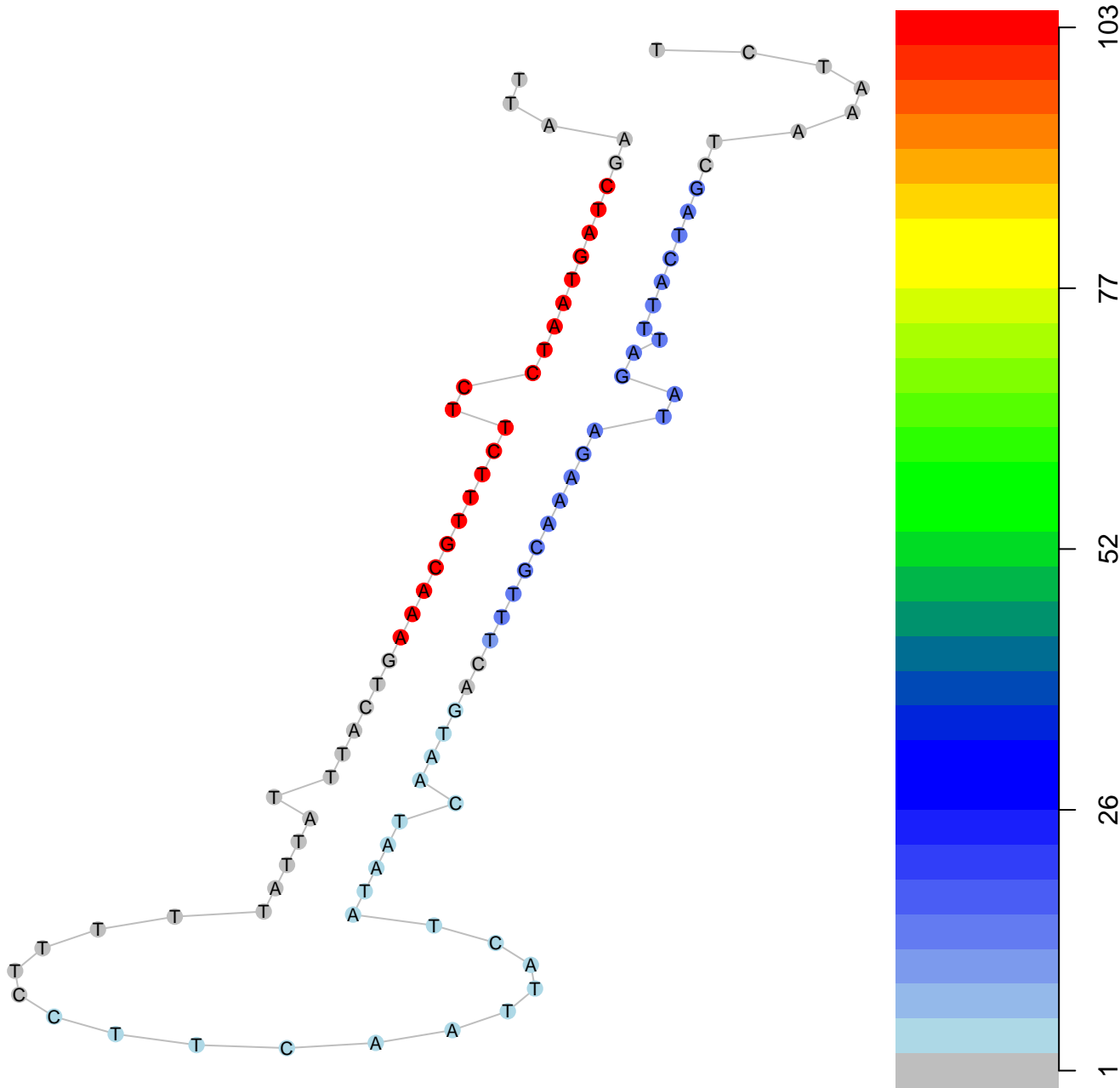

LG\_III:15321547..15321639

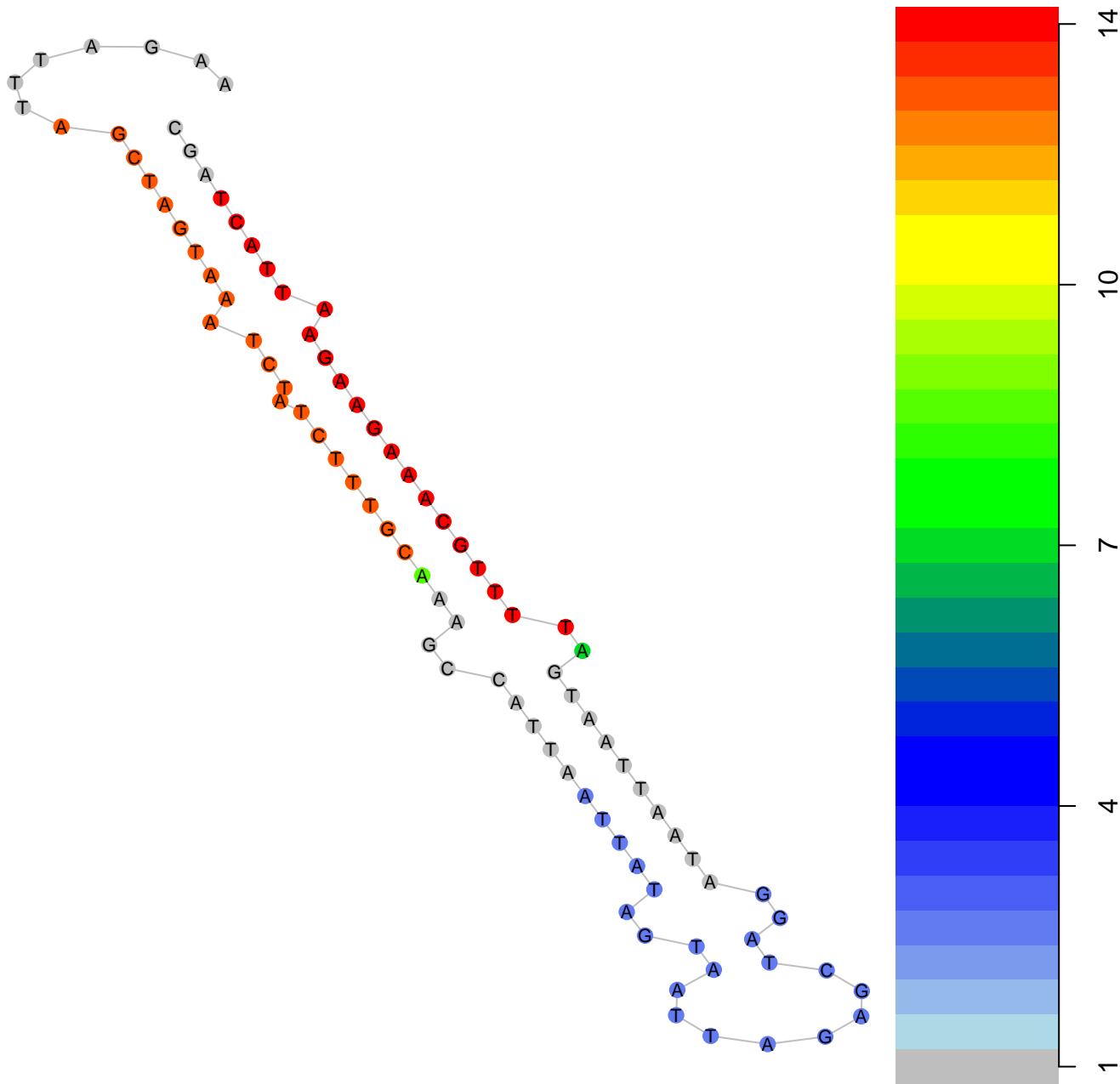

scaffold\_2286:2817..2909

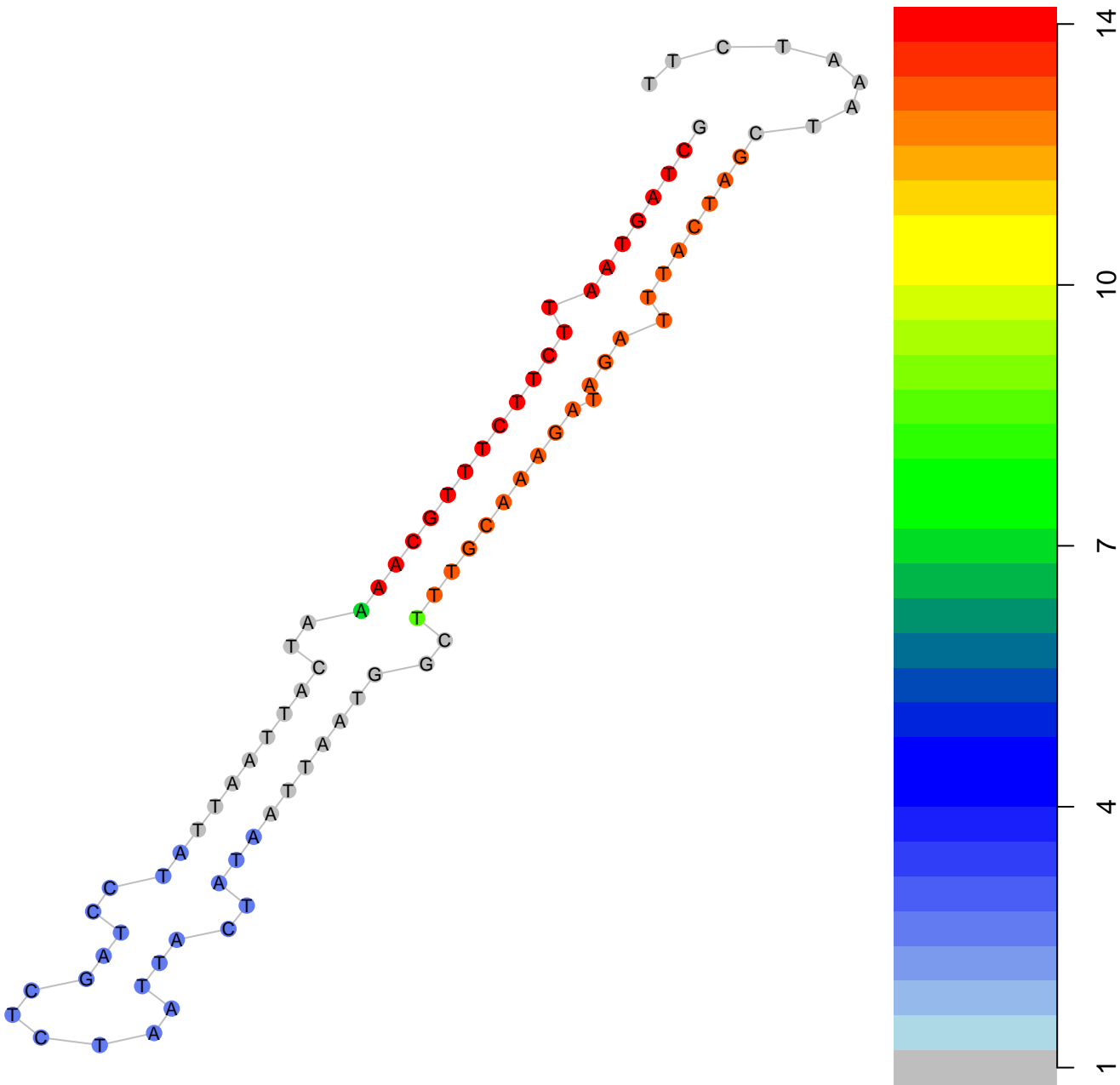

Supplement: Additional file 8 — Hairpin structure of miR476. Diagrammatic representation of the predicted stem-loop hairpin structure of miR475. The read count of sequences along the sequence is indicated using coloured shading. [file 1471-2164-10-620-S8.PDF]

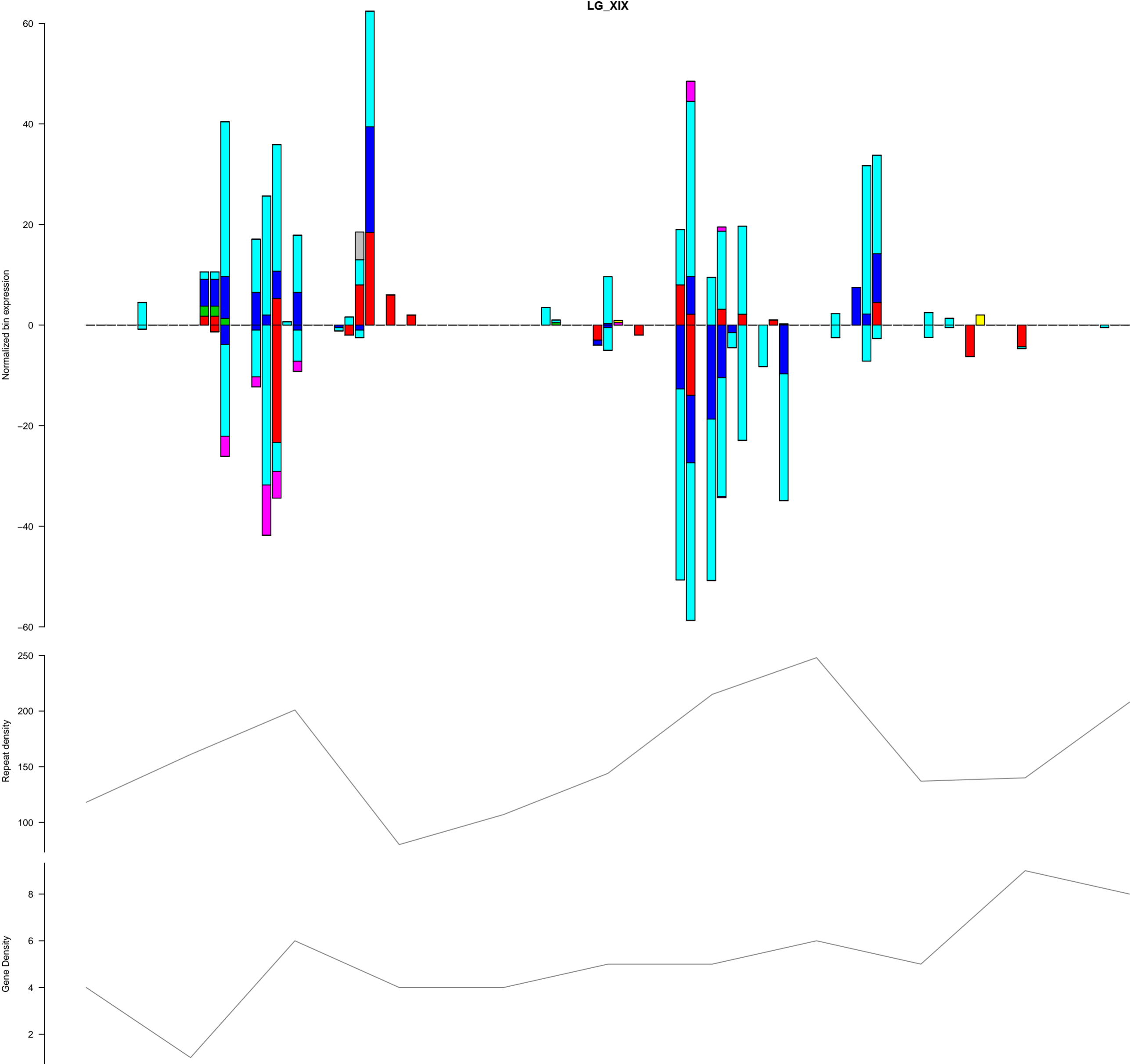

Supplement: Additional file 10 — Chromosome distribution plot for 1st 1 Mb of chromosome 19. short RNA, gene and repeat density plot for the 1st 1 Mb of chromosome 19 (LG_XIX). Coloured bars, above the axis for plus strand and below the axis for minus strand, show expression counts in 0.1 Mb windows for 18 (grey), 19 (yellow), 20 (purple), 21 (cyan), 22 (dark blue), 23 (green) and 24 (red) nucleotide sequences. Below each plot the frequency distribution in 0.1 Mb windows for gene (top) and repeat density (bottom) is shown. Repeat density was calculated using RepeatMasker data from the PopGenIE web resource [29]. [file 1471-2164-10-620-S10.PDF]

scaffold\_117

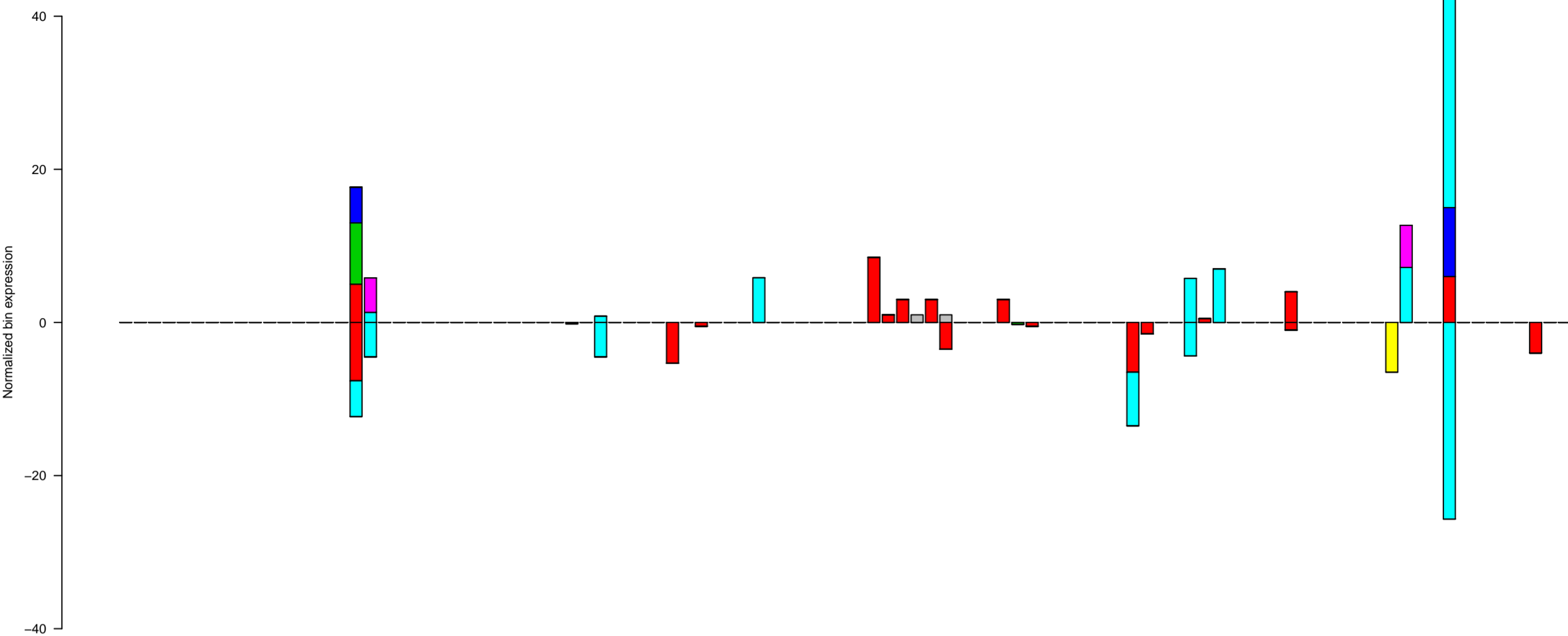

Supplement: Additional file 11 — Chromosome distribution plot of scaffold_117. short RNA, gene and repeat density plots of scaffold_117. Coloured bars, above the axis for plus strand and below the axis for minus strand, show expression counts in 0.1 Mb windows for 18 (grey), 19 (yellow), 20 (purple), 21 (cyan), 22 (dark blue), 23 (green) and 24 (red) nucleotide sequences. Below each plot the frequency distribution in 0.1 Mb windows for gene (top) and repeat density (bottom) is shown. Repeat density was calculated using RepeatMasker data from the PopGenIE web resource [29]. [file 1471-2164-10-620-S11.PDF]
