# Supplementary material for: Genome-wide profiling of Populus small RNAs
Source: BMC Genomics. 2009 Dec 20;10:620. doi: 10.1186/1471-2164-10-620 (PMC2811130; doi:10.1186/1471-2164-10-620)
Supplement: Additional file 9 — Chromosome distribution plots. short RNA, gene and repeat density plots for all chromosomes. Coloured bars, above the axis for plus strand and below the axis for minus strand, show expression counts in 0.1 Mb windows for 18 (grey), 19 (yellow), 20 (purple), 21 (cyan), 22 (dark blue), 23 (green) and 24 (red) nucleotide sequences. Below each plot the frequency distribution in 0.1 Mb windows for gene (top) and repeat density (bottom) is shown. Repeat density was calculated using RepeatMasker data from the PopGenIE web resource [29]. [file 1471-2164-10-620-S9.PDF]

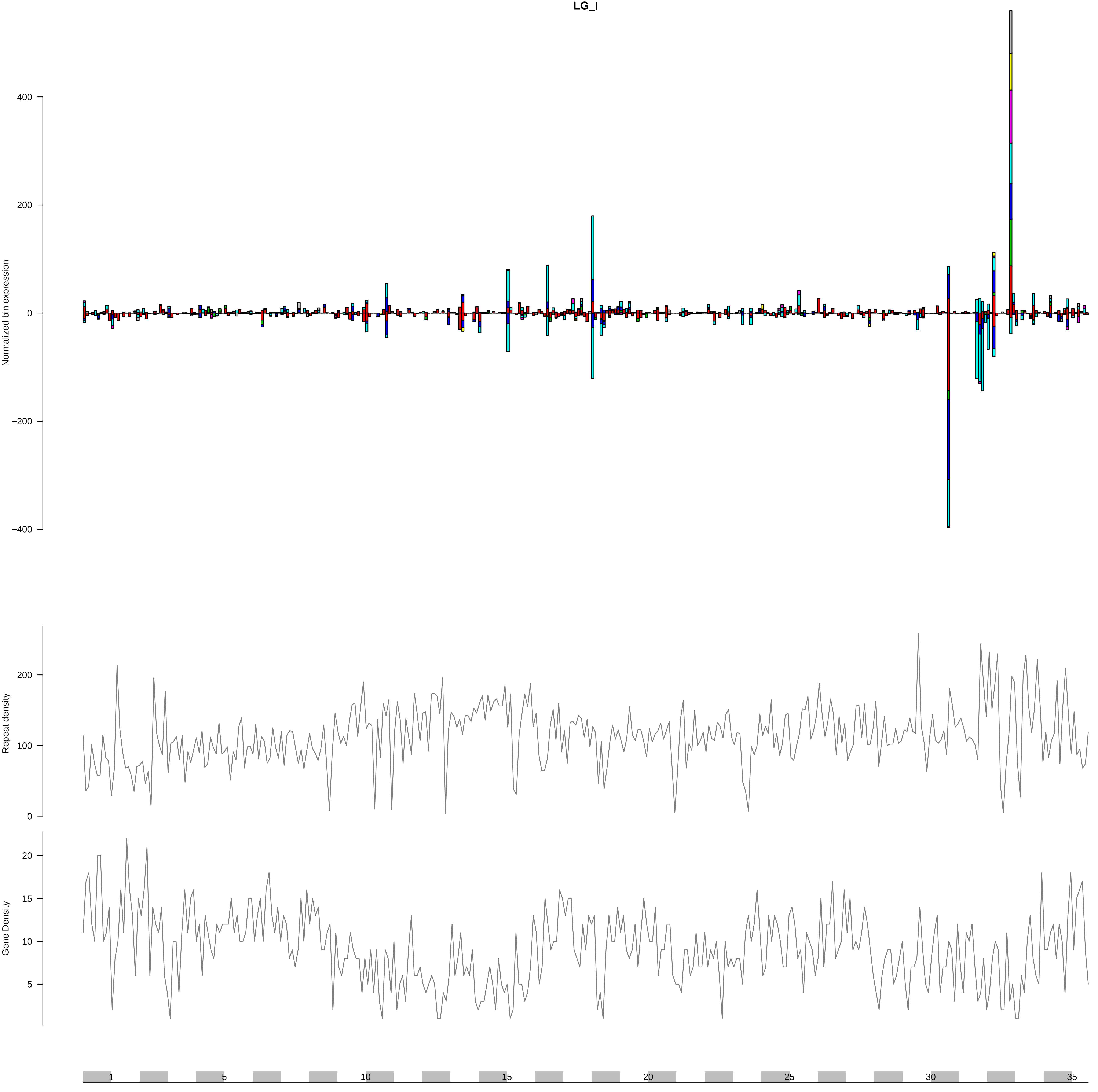
LG_I
Normalized bin expression
400
200
0
−200
−400
Repeat density
200
100
0
Gene Density
20
15
10
5
1
5
10
15
20
25
30
35

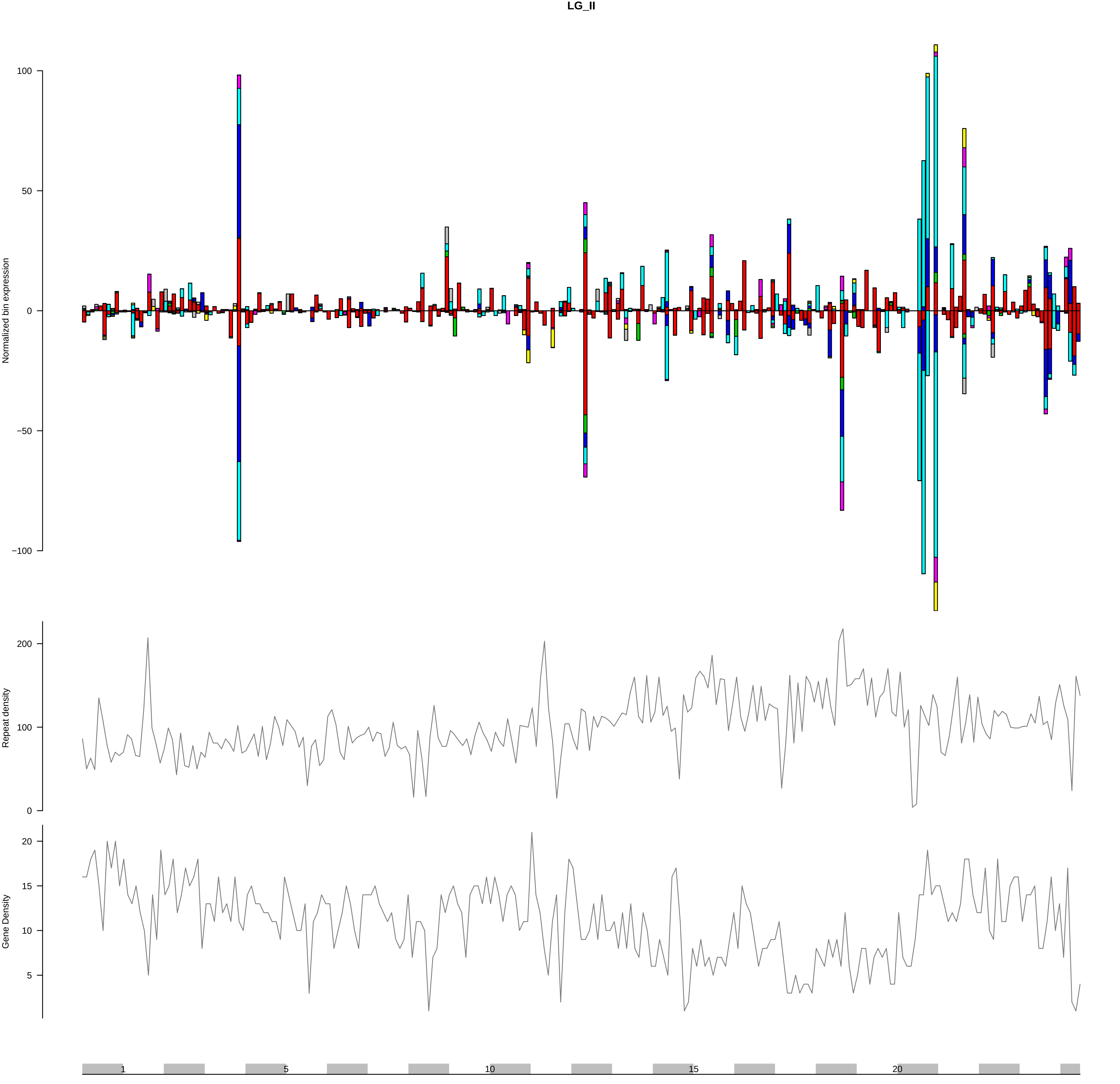
LG_II
Normalized bin expression
100
50
0
−50
−100
Repeat density
200
100
0
Gene Density
20
15
10
5
1
5
10
15
20

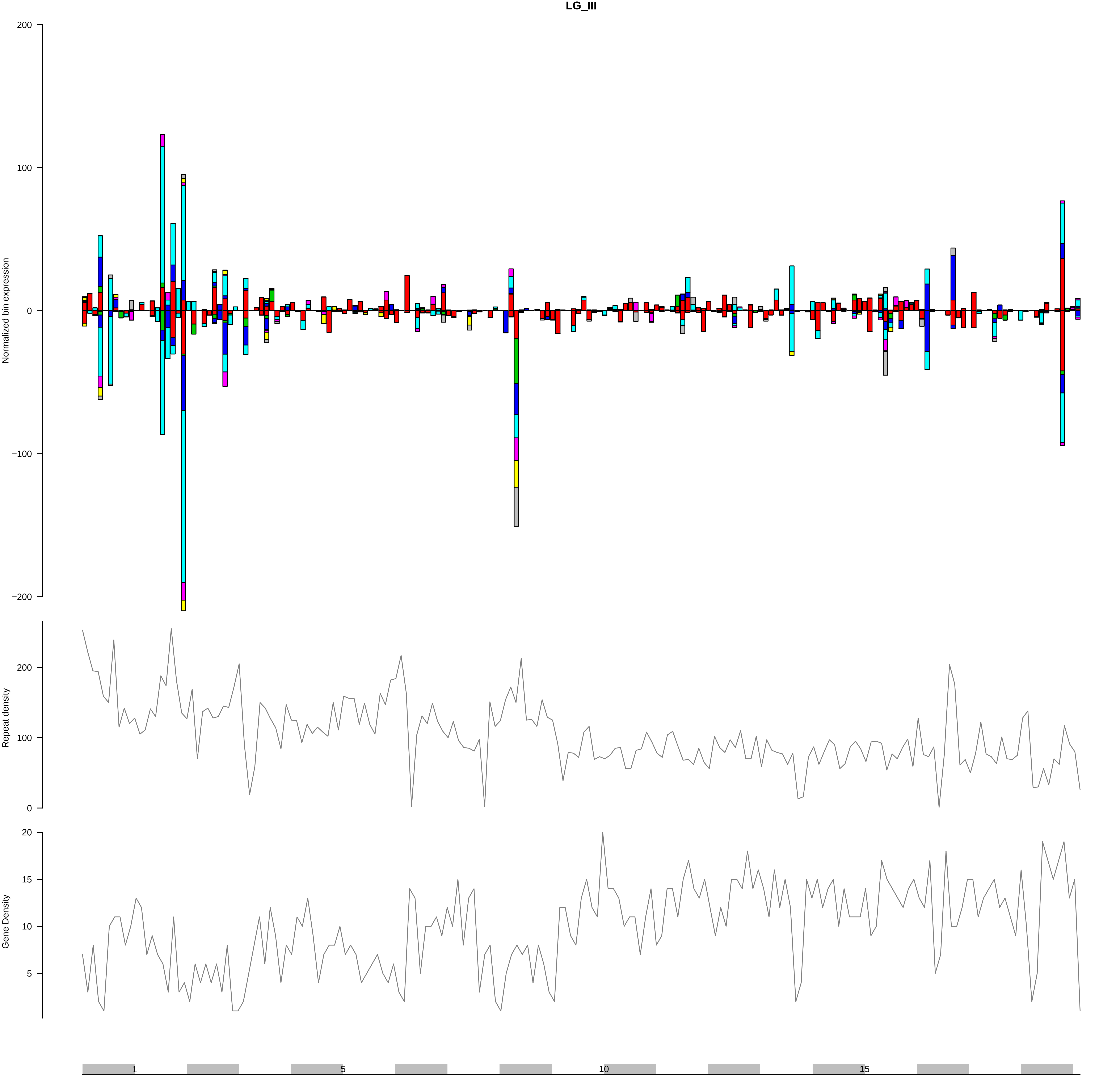
LG_III
Normalized bin expression
200
100
0
-100
-200
Repeat density
200
100
0
Gene Density
20
15
10
5
1
5
10
15

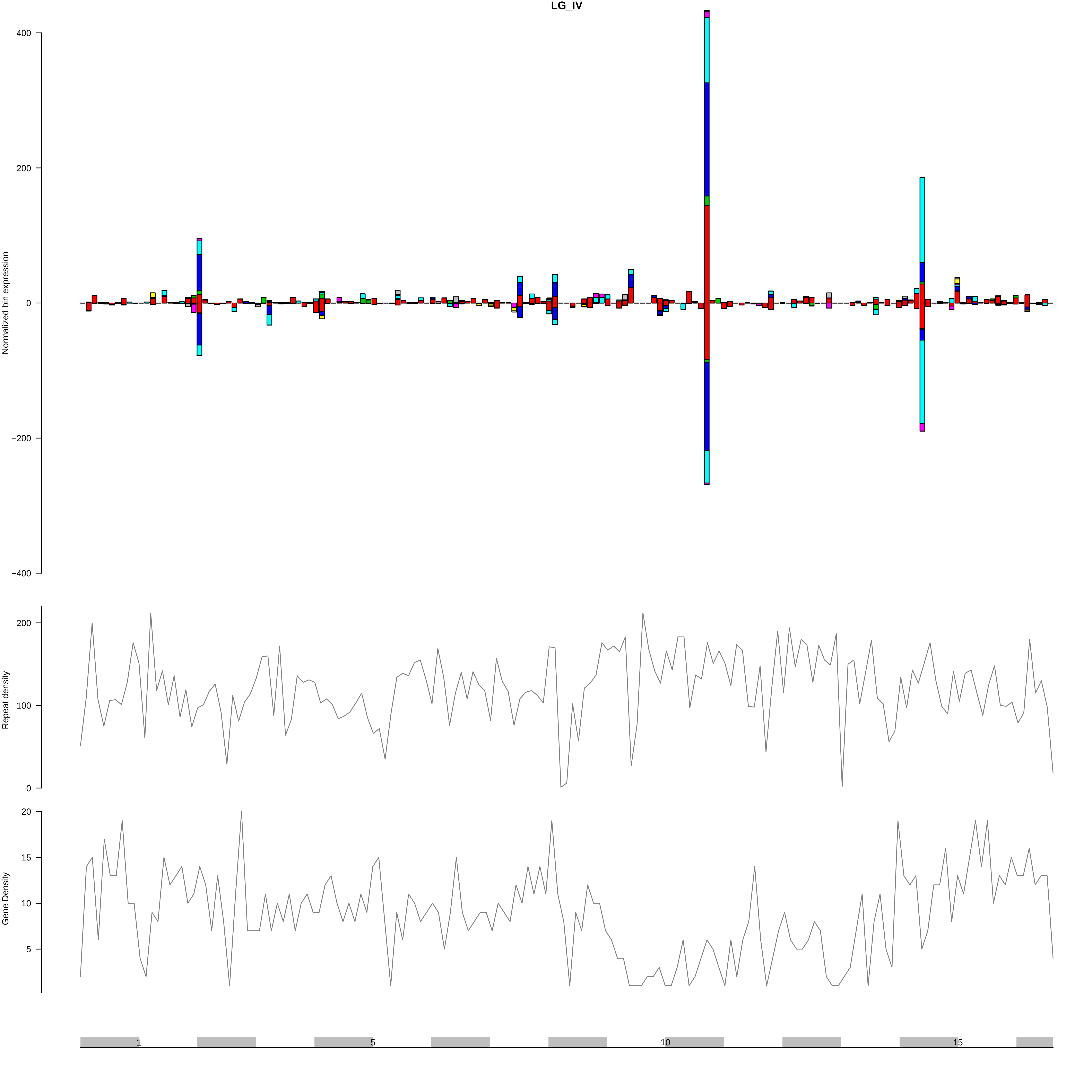

LG_IV
Normalized bin expression
400
200
0
-200
-400
Repeat density
200
100
0
Gene Density
20
15
10
5
1
5
10
15

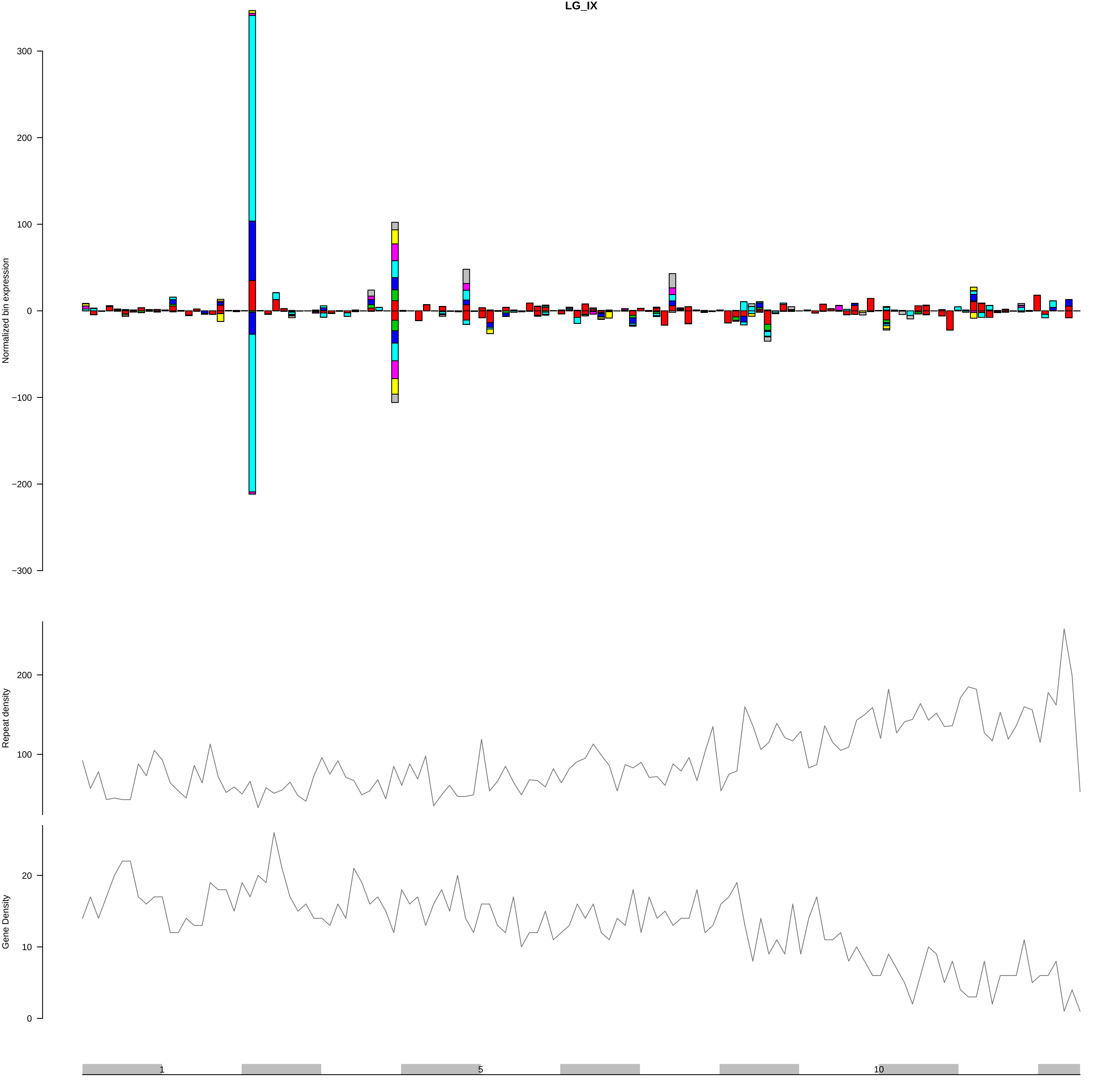
LG_IX
Normalized bin expression
300
200
100
0
−100
−200
−300
Repeat density
200
100
Gene Density
20
10
0
1
5
10

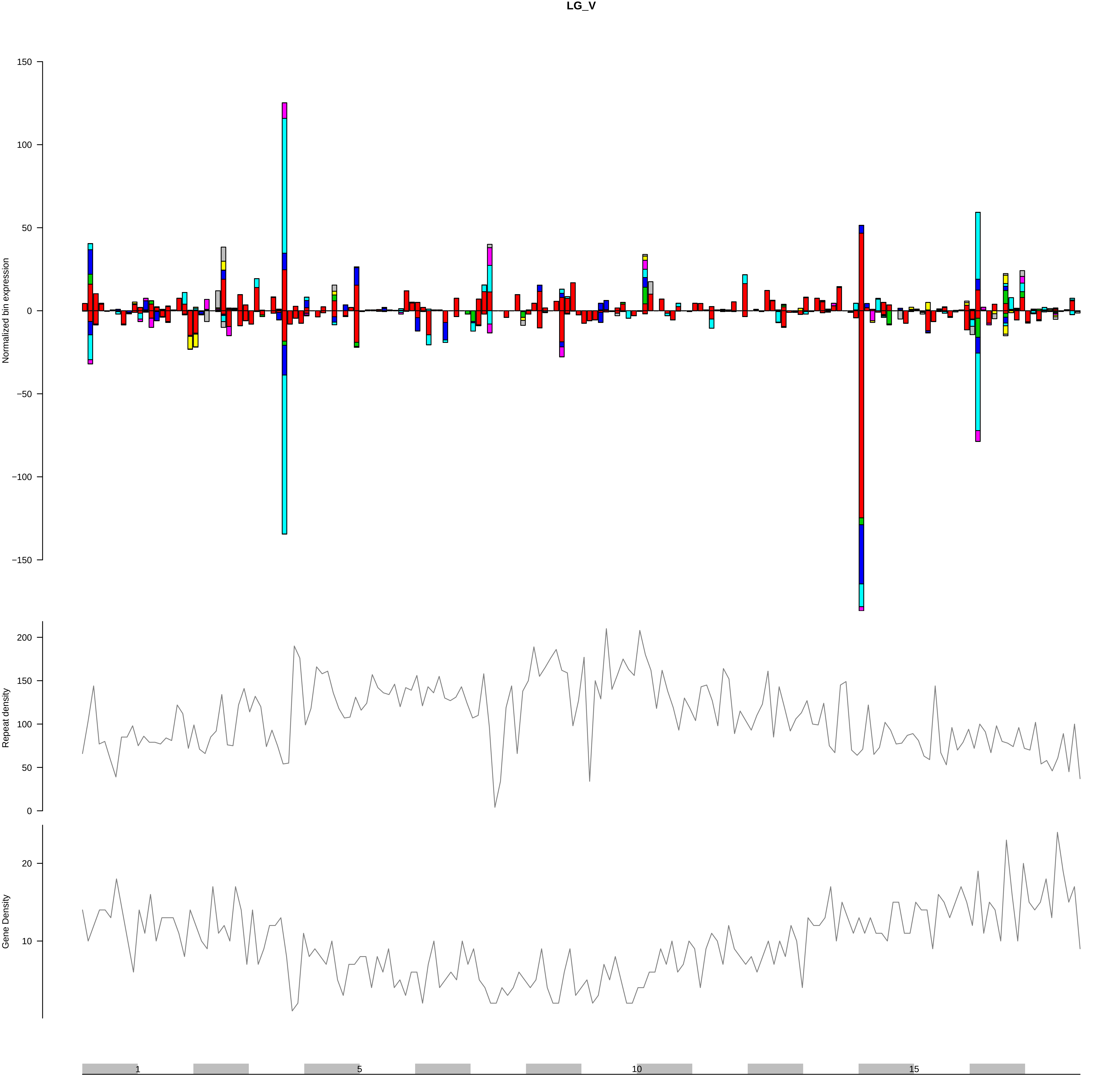
LG_V
Normalized bin expression
150
100
50
0
−50
−100
−150
Repeat density
200
150
100
50
0
Gene Density
20
10
1
5
10
15

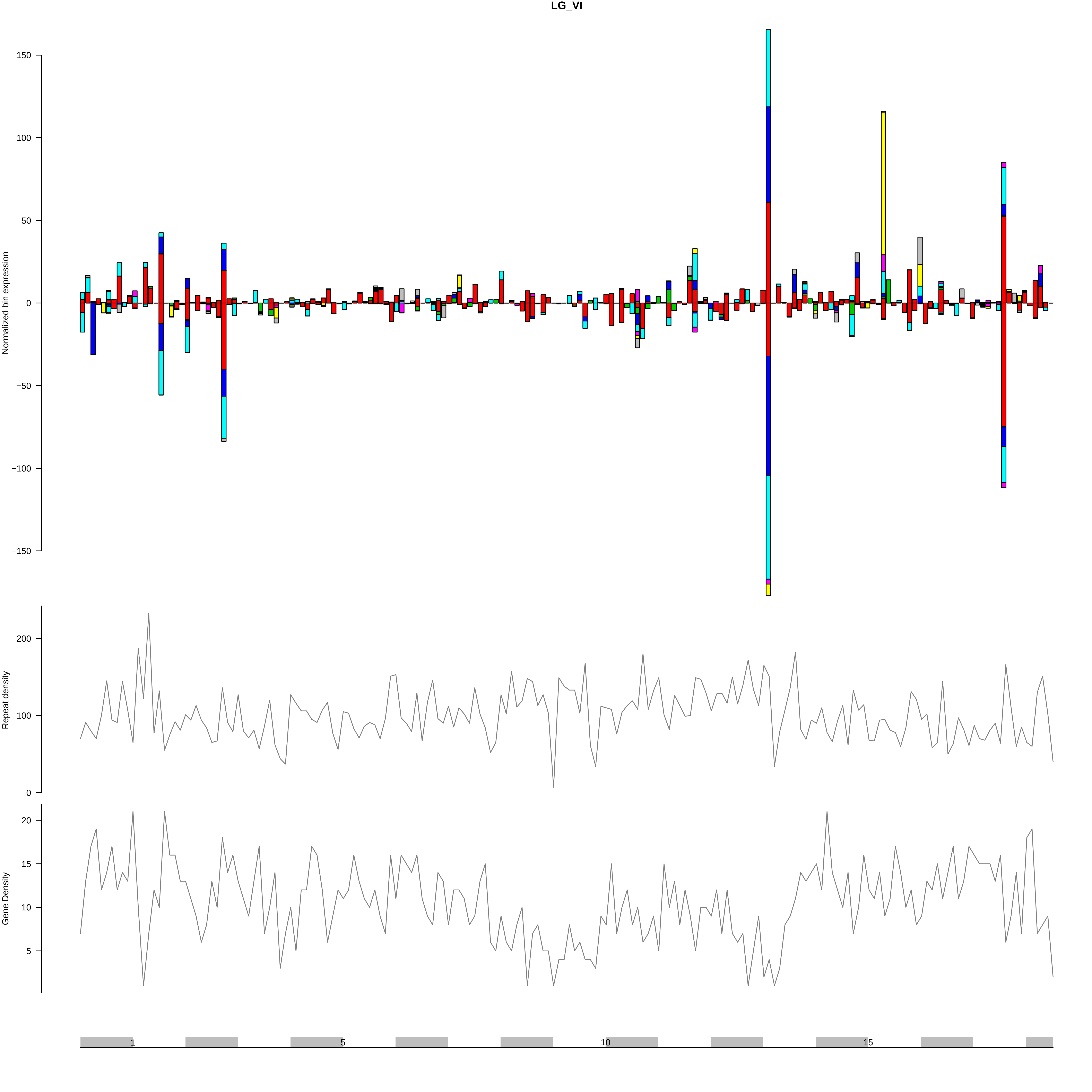

LG_VI
Normalized bin expression
150
100
50
0
−50
−100
−150
Repeat density
200
100
0
Gene Density
20
15
10
5
1
5
10
15

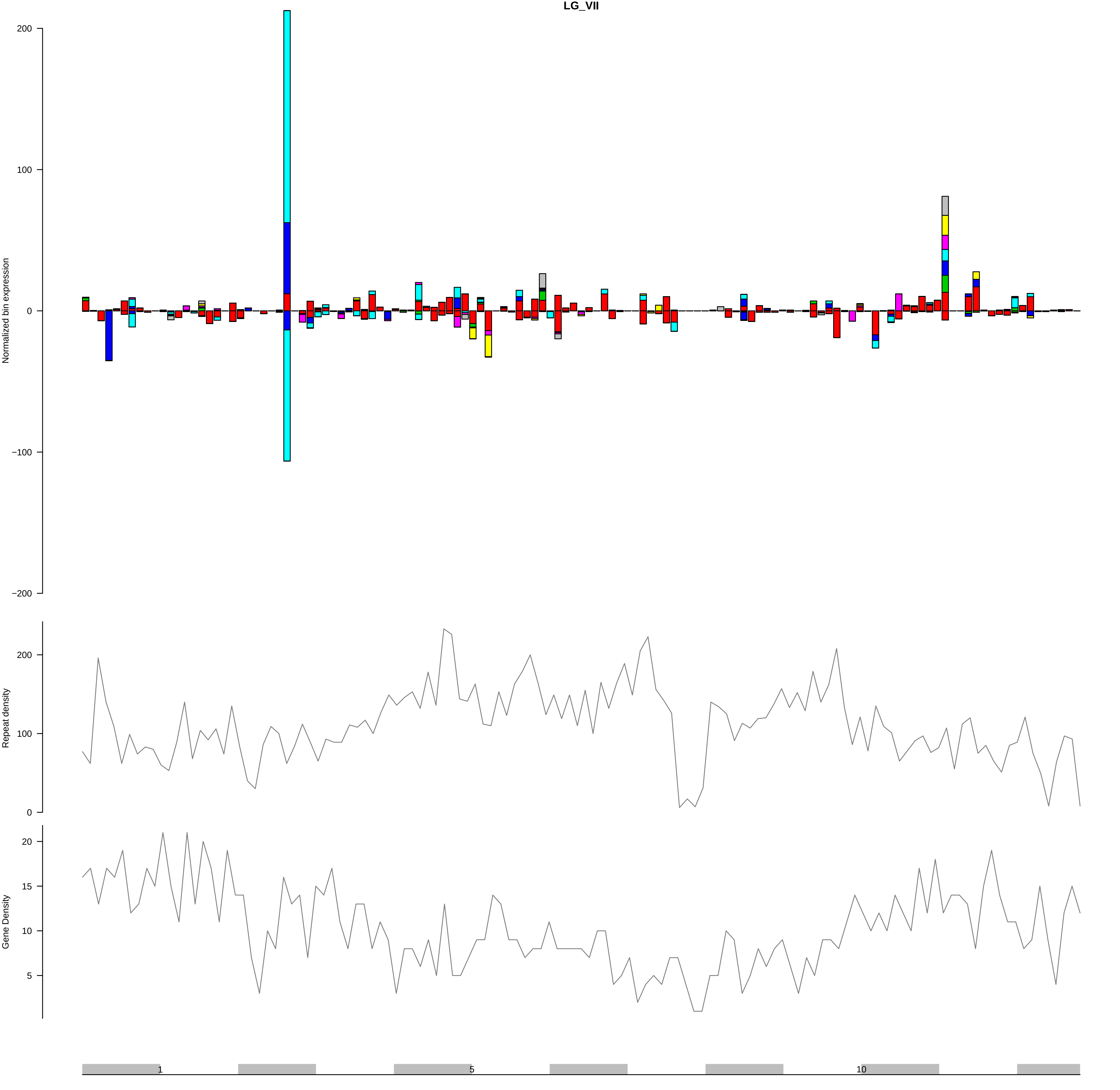
LG_VII
Normalized bin expression
200
100
0
-100
-200
Repeat density
200
100
0
Gene Density
20
15
10
5
1
5
10

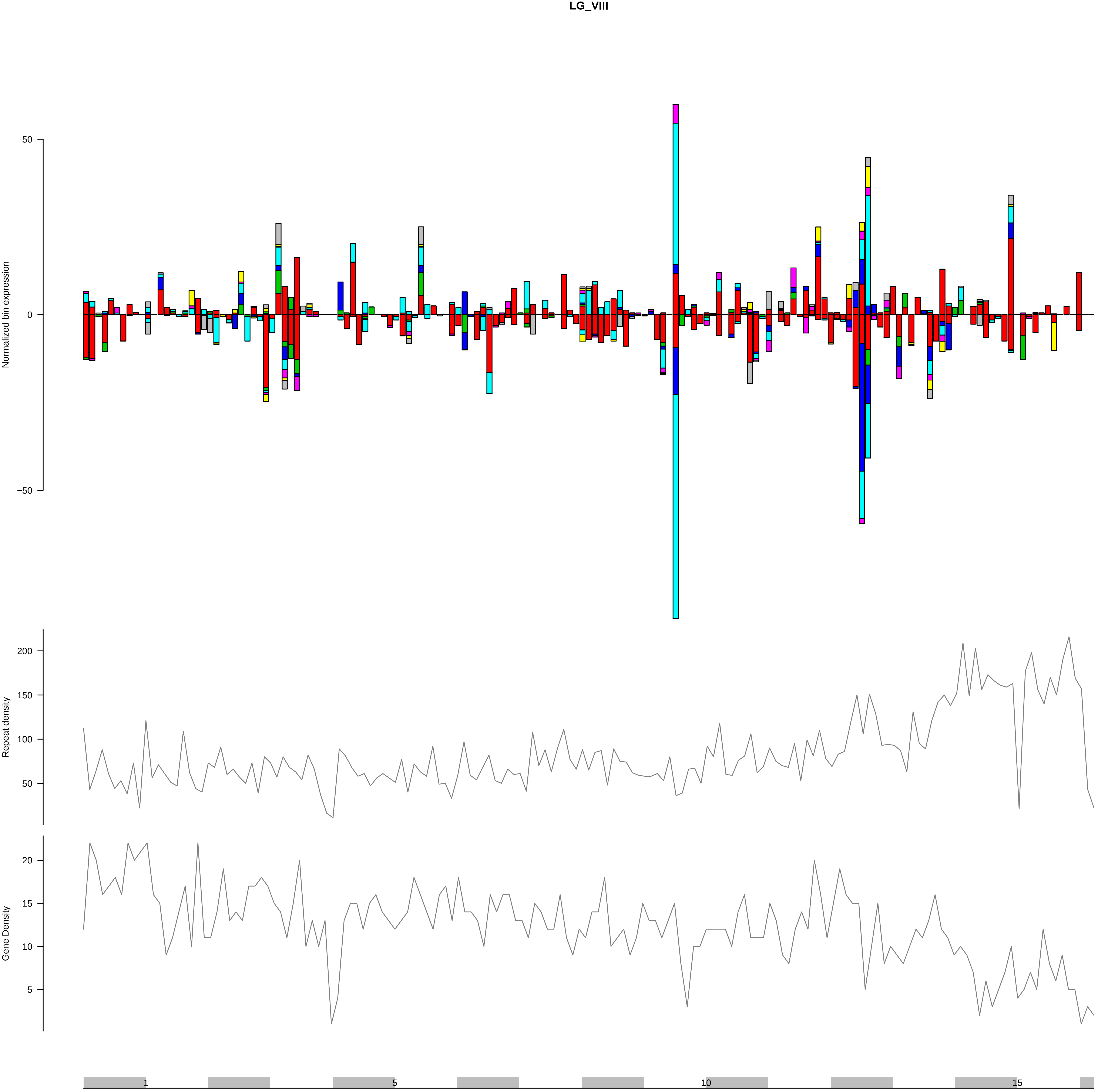

LG_VIII
Normalized bin expression
50
0
−50
Repeat density
200
150
100
50
Gene Density
20
15
10
5
1
5
10
15

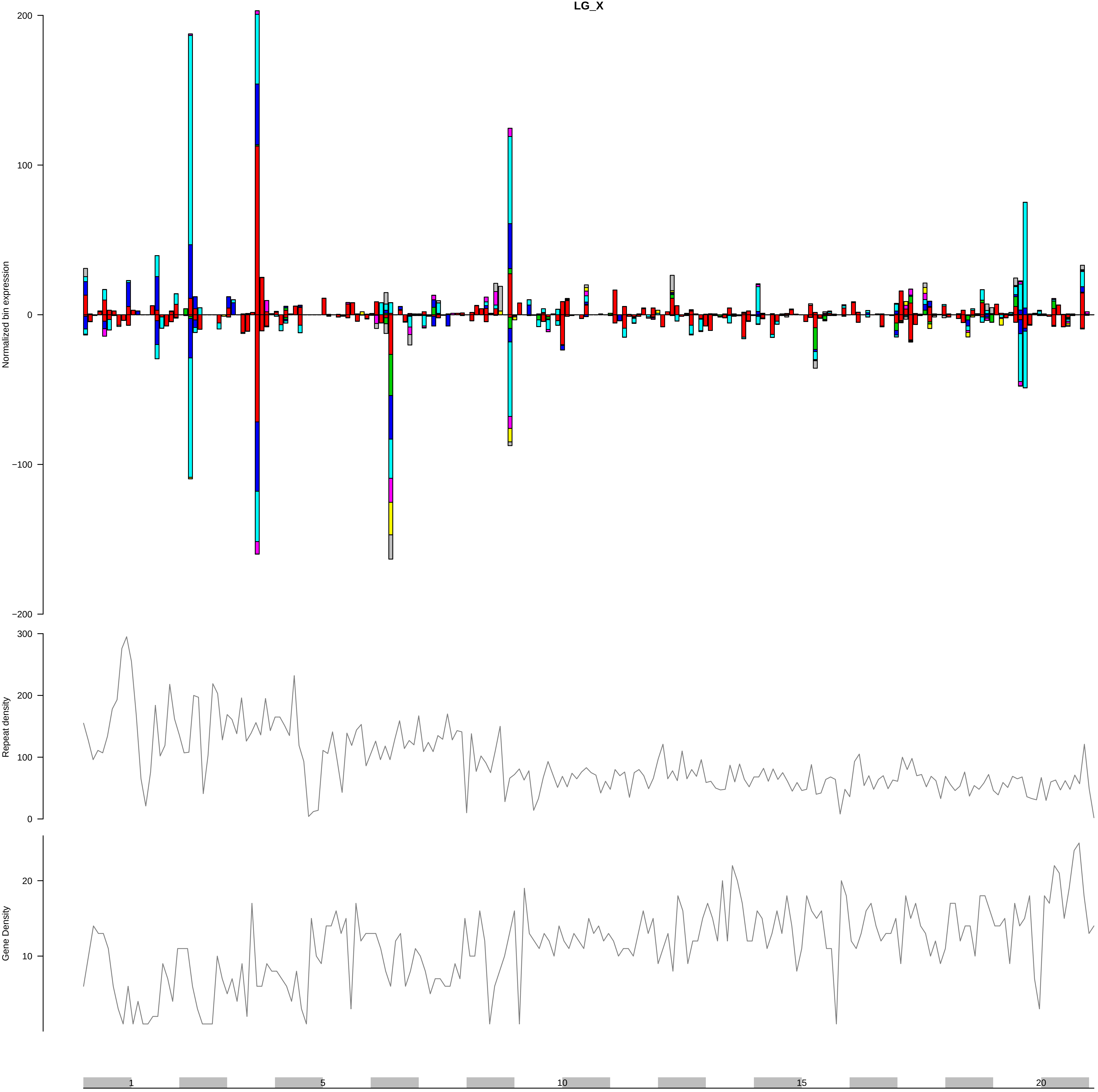

LG_X
Normalized bin expression
200
100
0
−100
−200
Repeat density
300
200
100
0
Gene Density
20
10
1
5
10
15
20

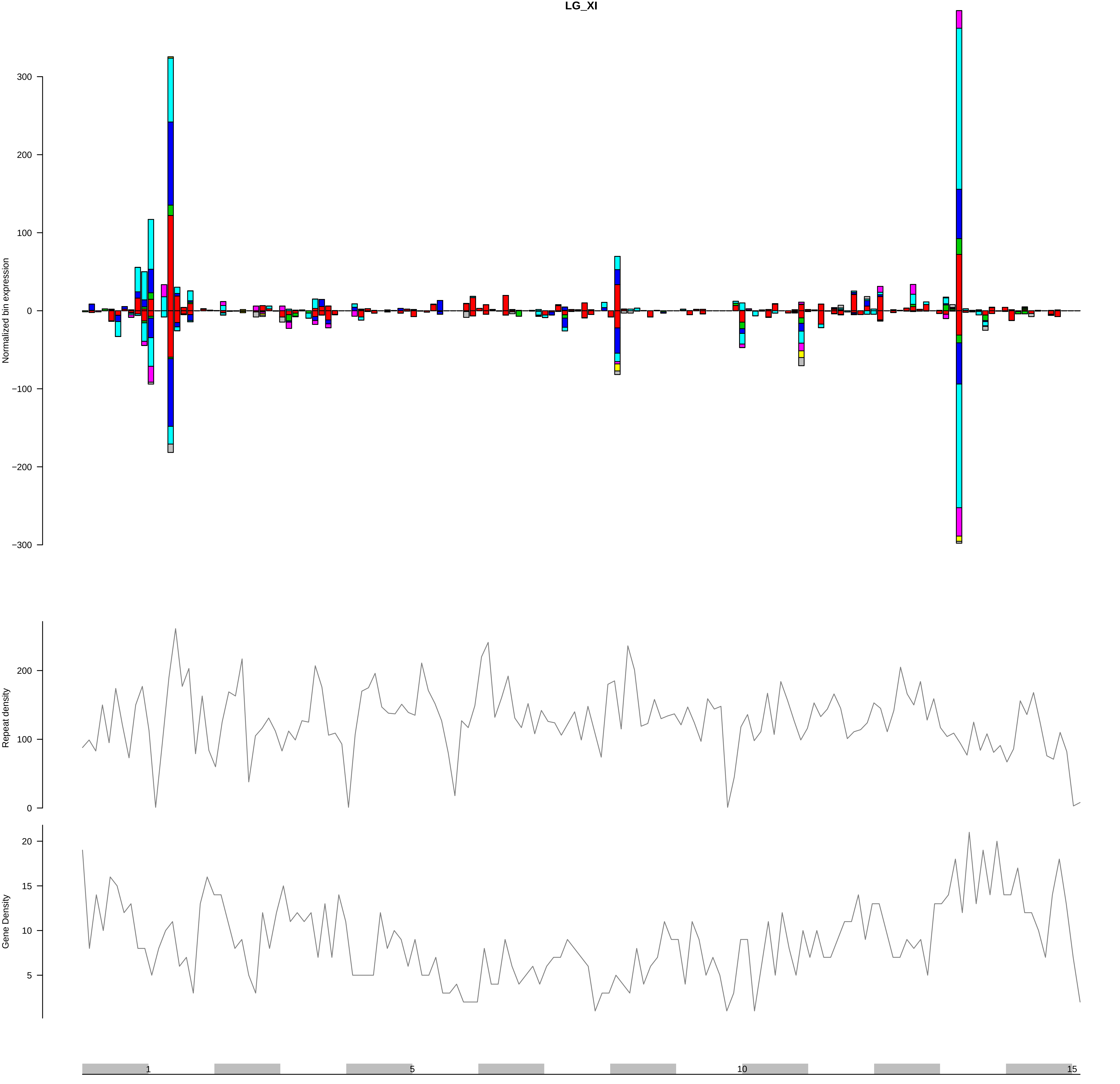

LG_XI
Normalized bin expression
300
200
100
0
−100
−200
−300
Repeat density
200
100
0
Gene Density
20
15
10
5
1
5
10
15

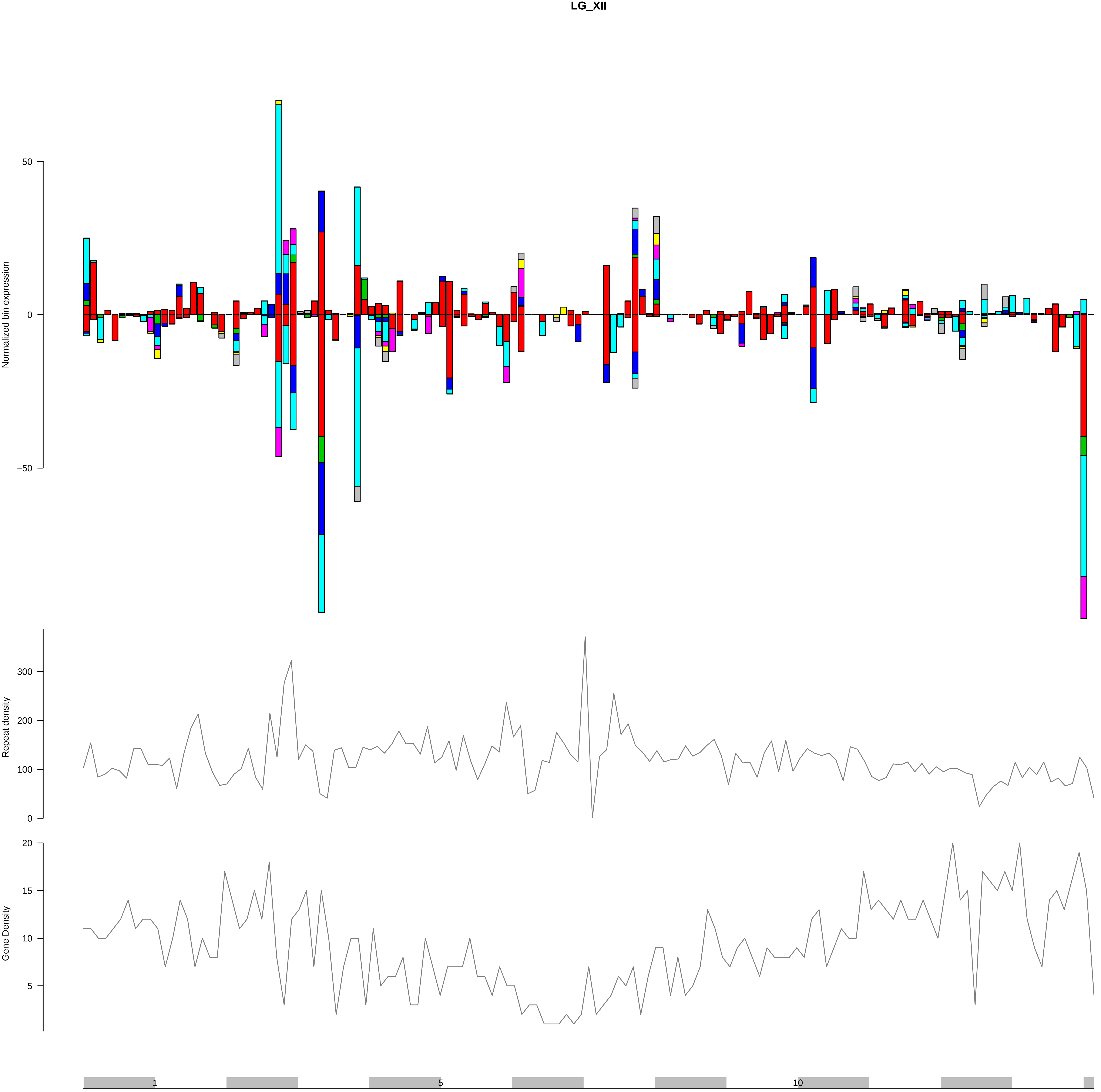

LG_XII
Normalized bin expression
50
0
-50
Repeat density
300
200
100
0
Gene Density
20
15
10
5
1
5
10

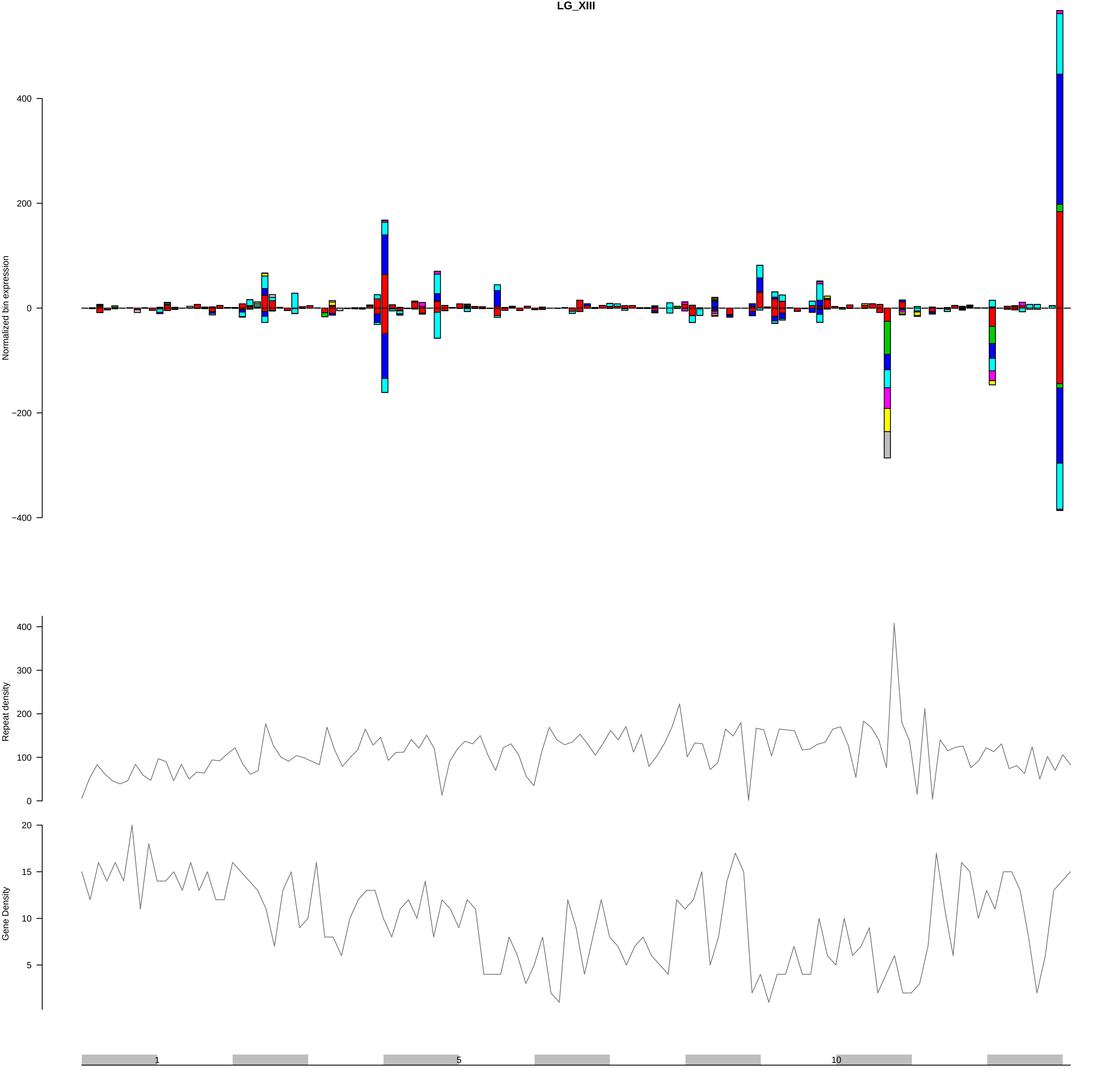

LG_XIII
Normalized bin expression
400
200
0
−200
−400
Repeat density
400
300
200
100
0
Gene Density
20
15
10
5
1
5
10

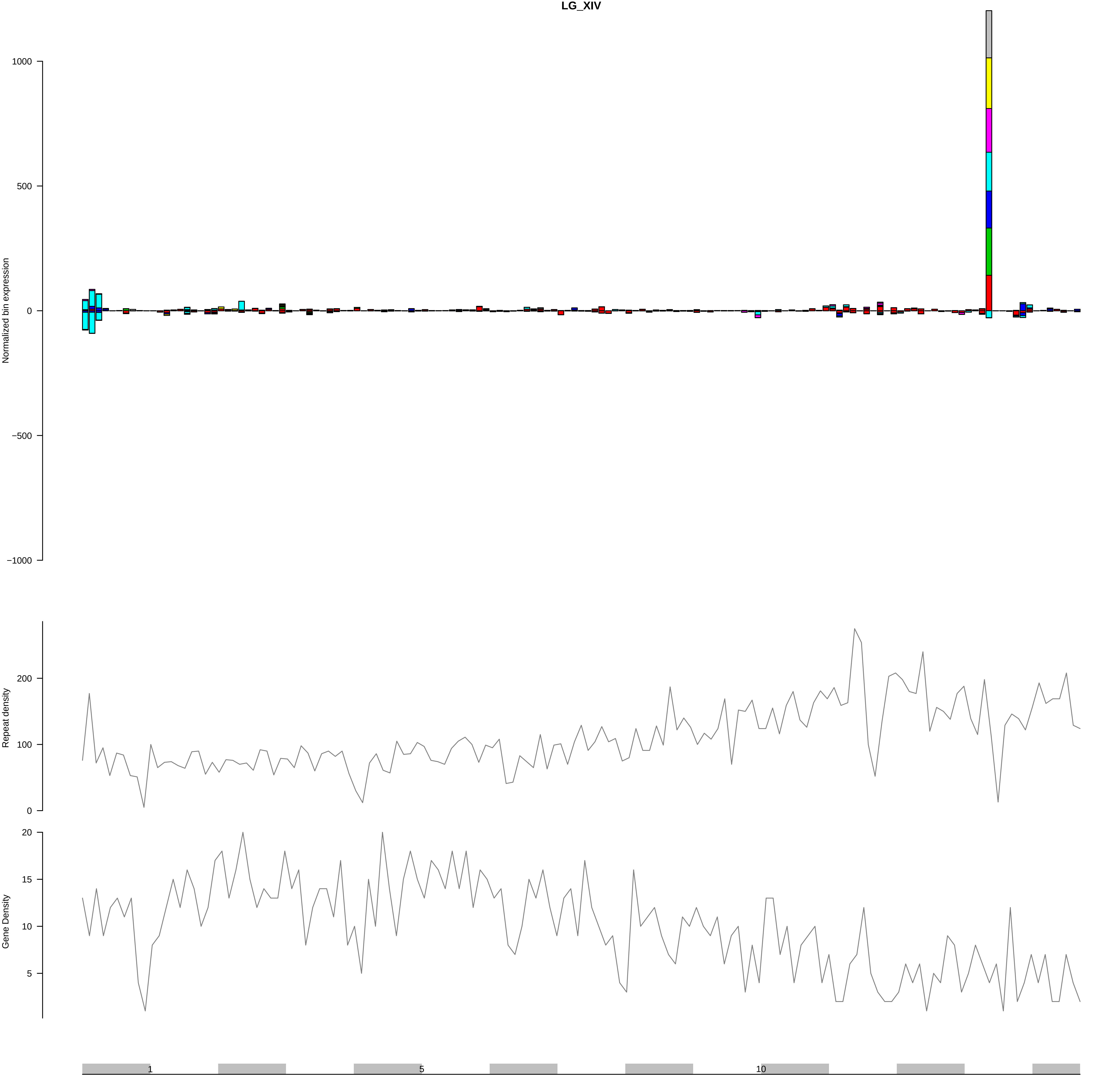
LG_XIV
Normalized bin expression
1000
500
0
-500
-1000
Repeat density
200
100
0
Gene Density
20
15
10
5
1
5
10

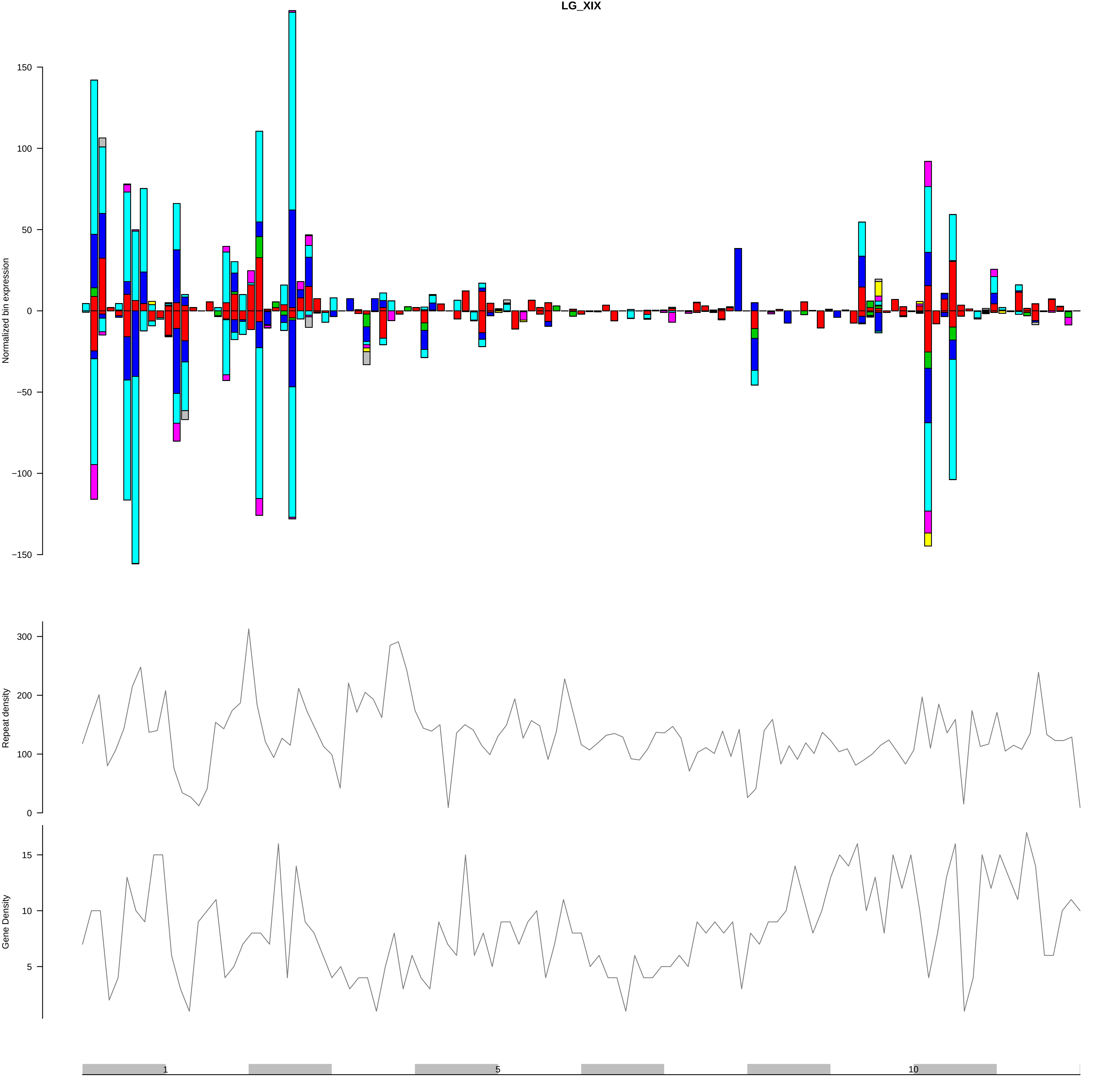
LG_XIX
Normalized bin expression
150
100
50
0
−50
−100
−150
Repeat density
300
200
100
0
Gene Density
15
10
5
1
5
10

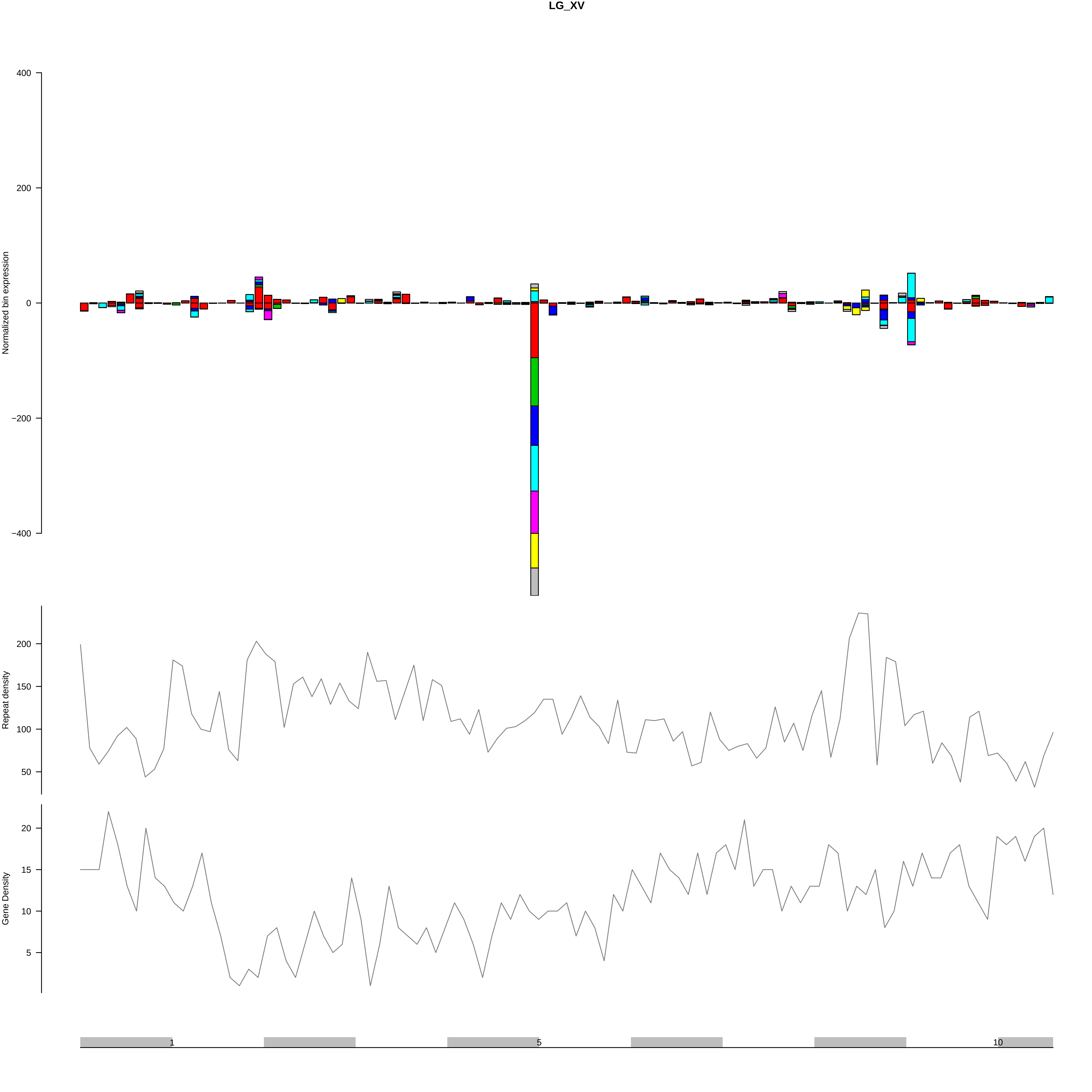
LG_XV
Normalized bin expression
400
200
0
−200
−400
Repeat density
200
150
100
50
Gene Density
20
15
10
5
1
5
10

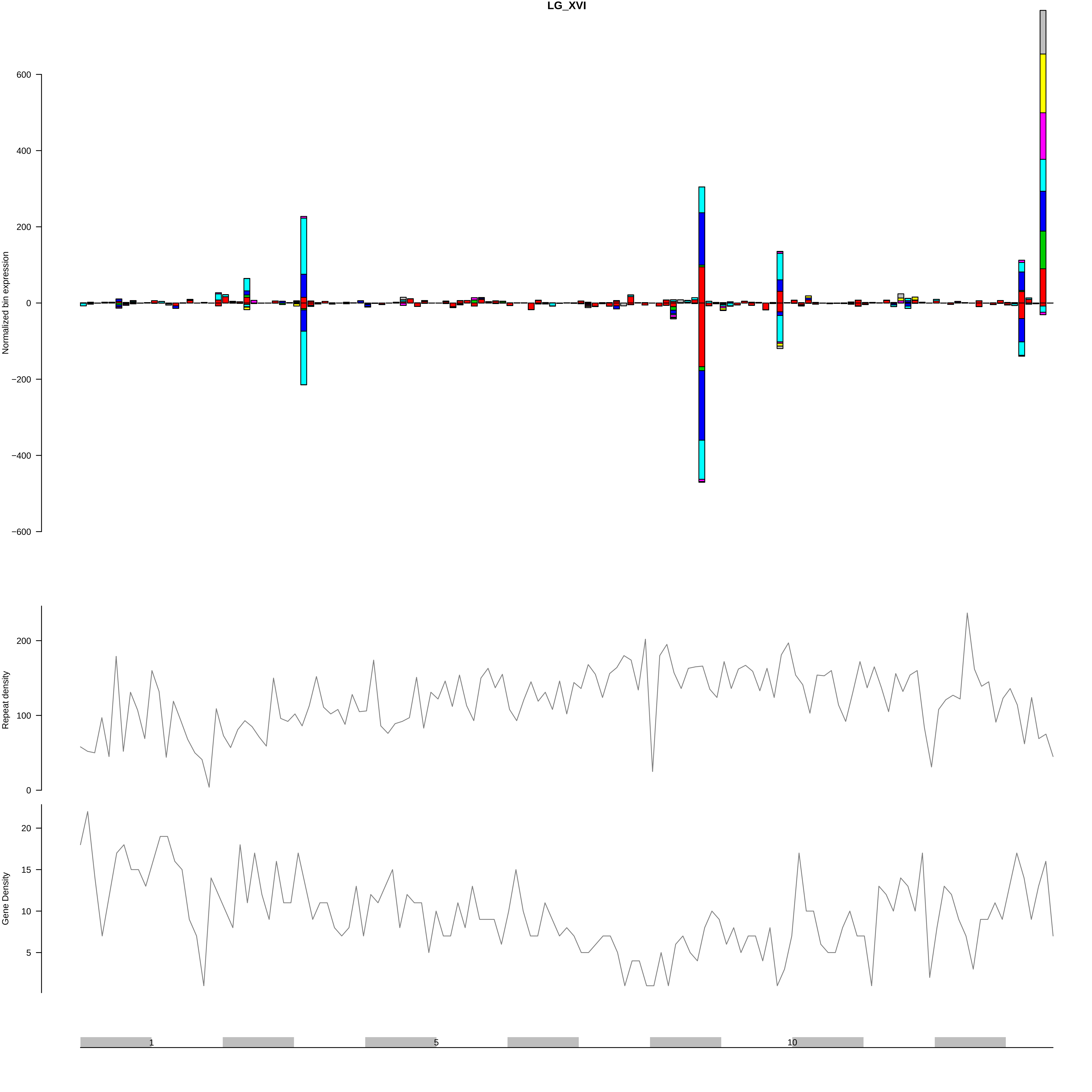
LG_XVI
Normalized bin expression
600
400
200
0
−200
−400
−600
Repeat density
200
100
0
Gene Density
20
15
10
5
1
5
10

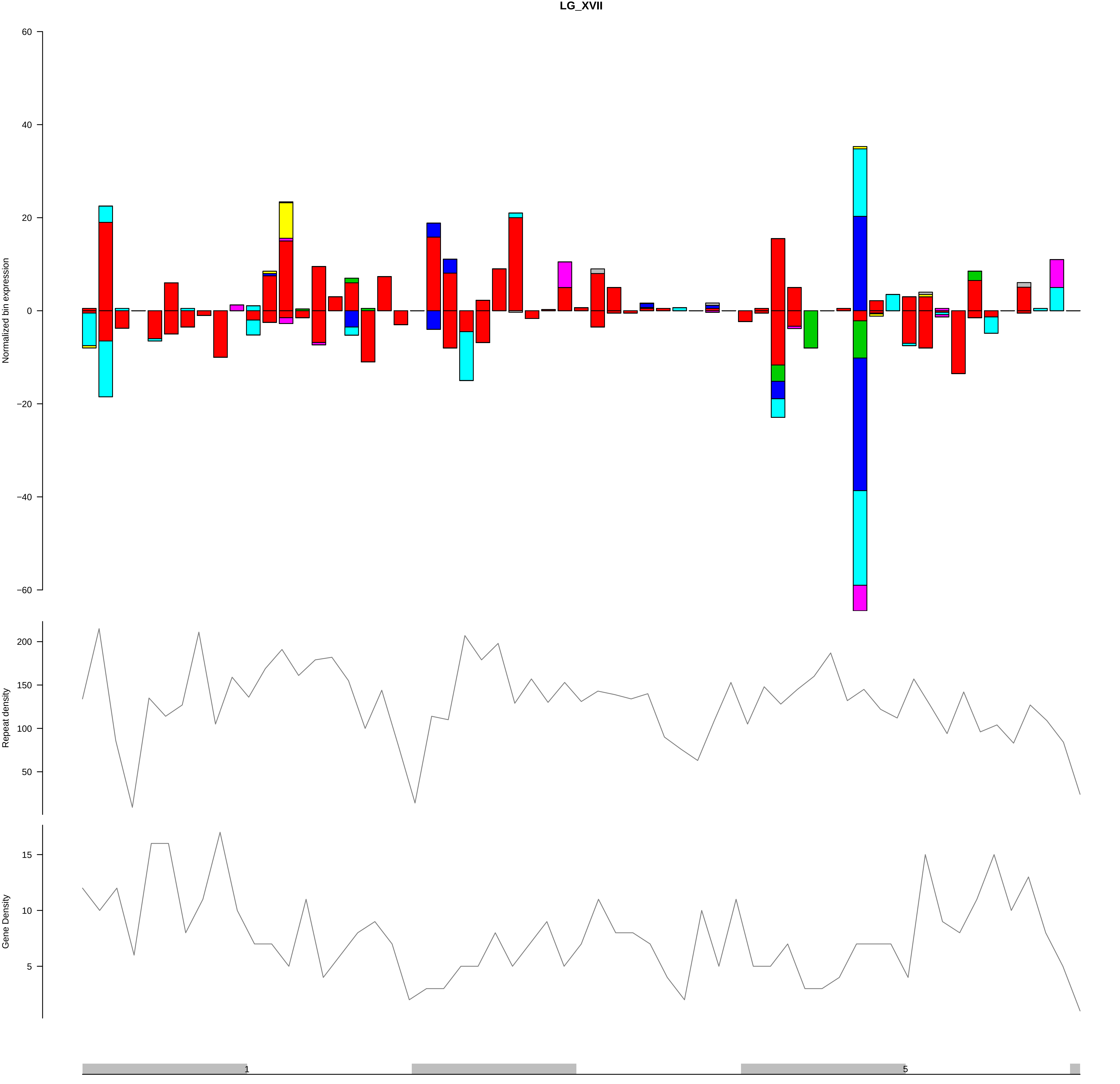
LG_XVII
Normalized bin expression
60
40
20
0
-20
-40
-60
Repeat density
200
150
100
50
Gene Density
15
10
5
1
5

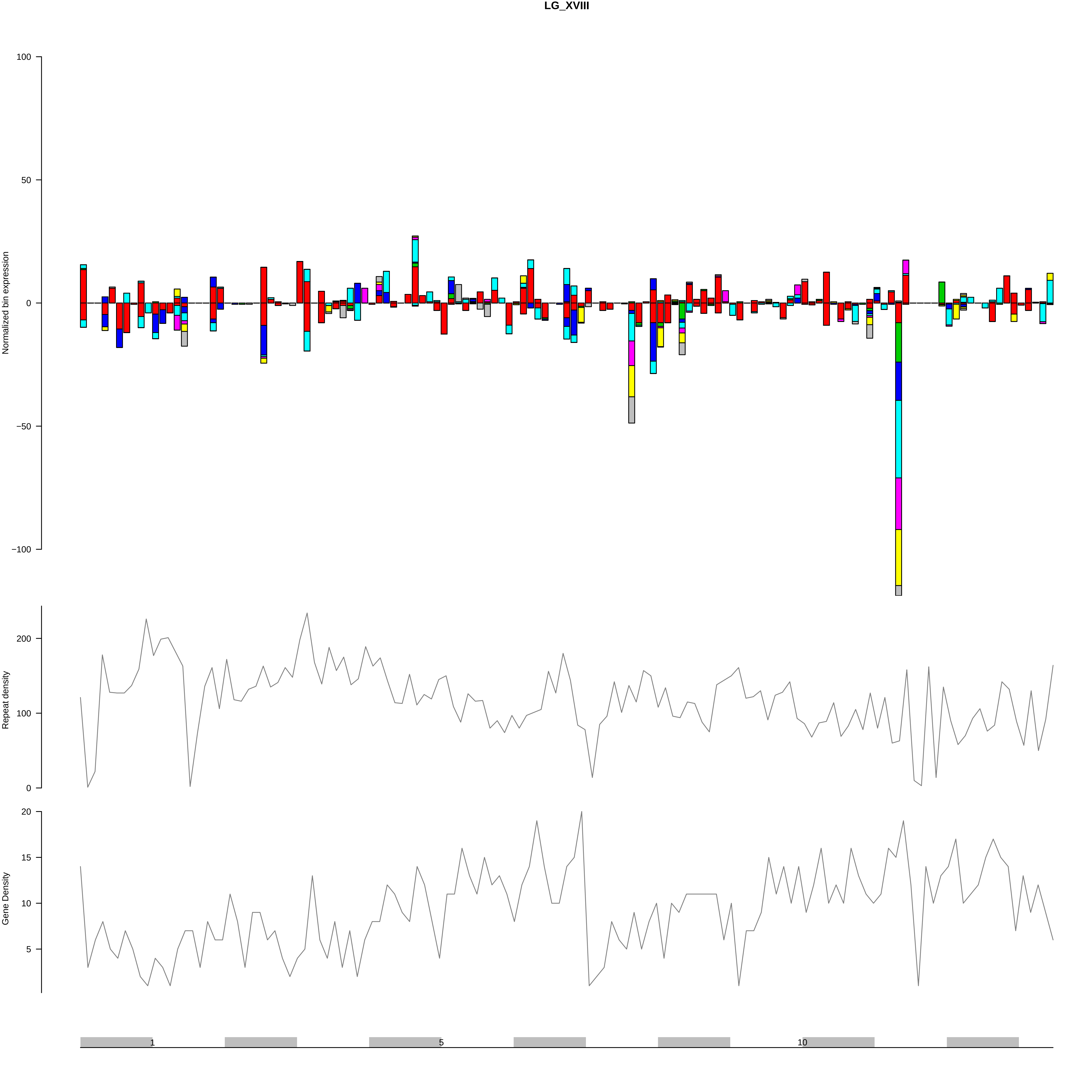
LG_XVIII
Normalized bin expression
100
50
0
−50
−100
Repeat density
200
100
0
Gene Density
20
15
10
5
1
5
10
