# Supplementary material for: Genome-wide profiling of Populus small RNAs
Source: BMC Genomics. 2009 Dec 20;10:620. doi: 10.1186/1471-2164-10-620 (PMC2811130; doi:10.1186/1471-2164-10-620)
Supplement: Additional file 15 — Predicted miRNA hairpin structures. Predicted stem-loop hairpin structures for all miRNA loci represented in Additional File 11. The location of the miRNA and miRNA*sequence is indicated in green and purple respectively. [file 1471-2164-10-620-S15.PDF]

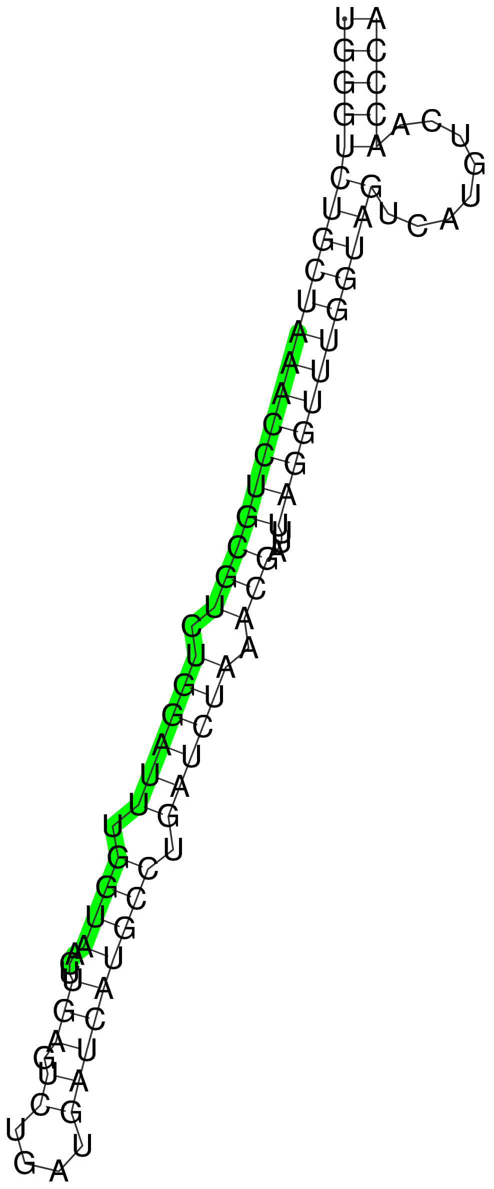

AAACCTGCGTCTGGATTTGGTAAC_LG_VI_6919031-6919054

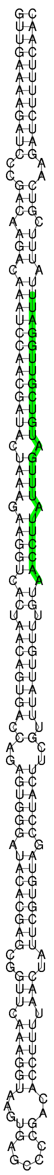

AACCTTATTTGATGTCGTTGGATT_scaffold_168_179570-179593

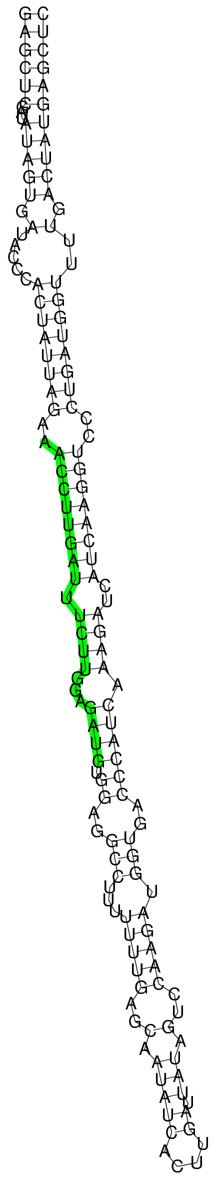

AACCTTGATTTCTTGGAGATG_LG_X_14539951-14539971

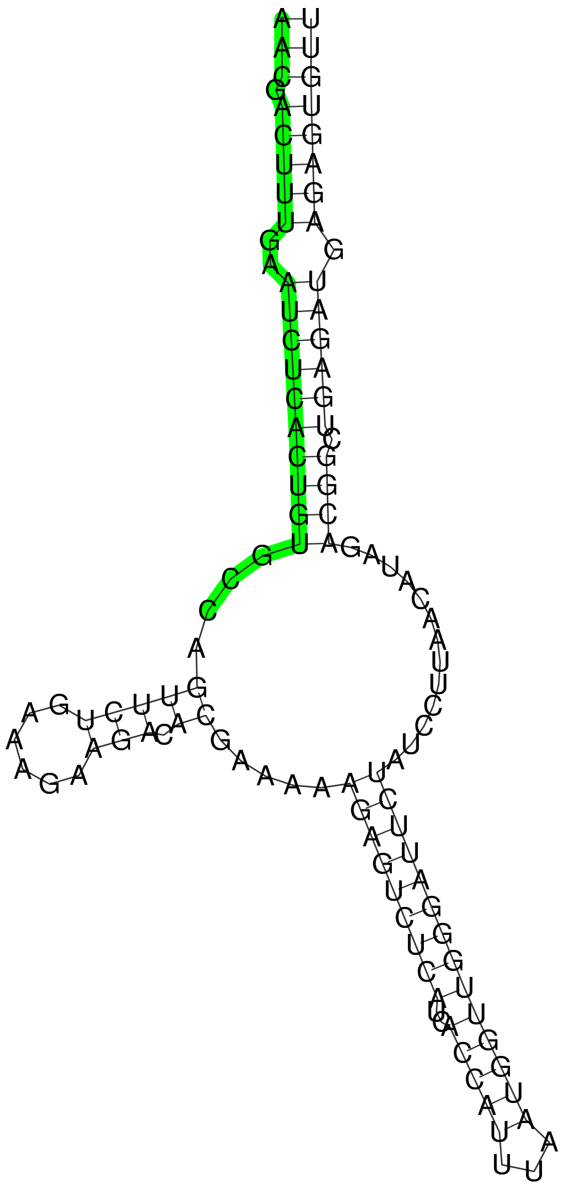

AACGACTTTGAATCTCACTGTGCC_LG_XV_9614437-9614460

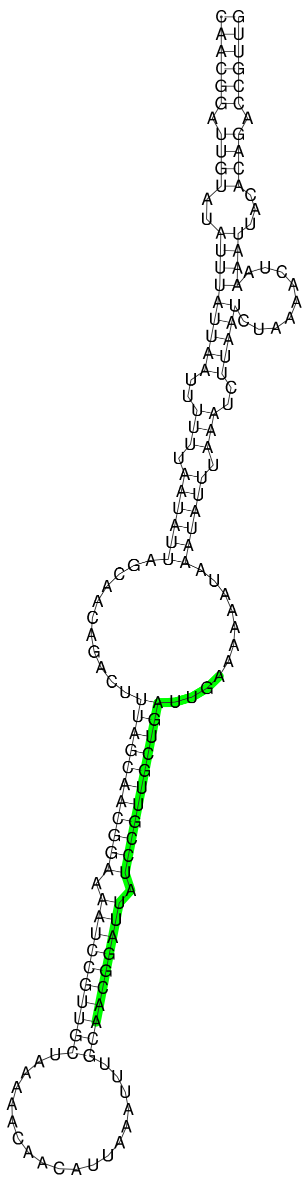

AACGGATTATCCGTTGCTGATTGA_LG_XIII_6525128-6525151

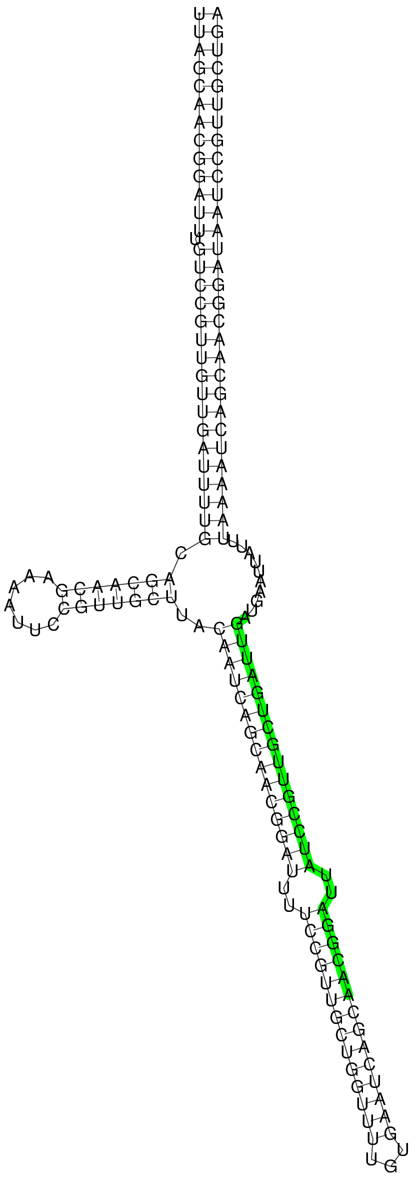

AACGGATTATCCGTTGCTGATTGA_scaffold_64_153710-153733

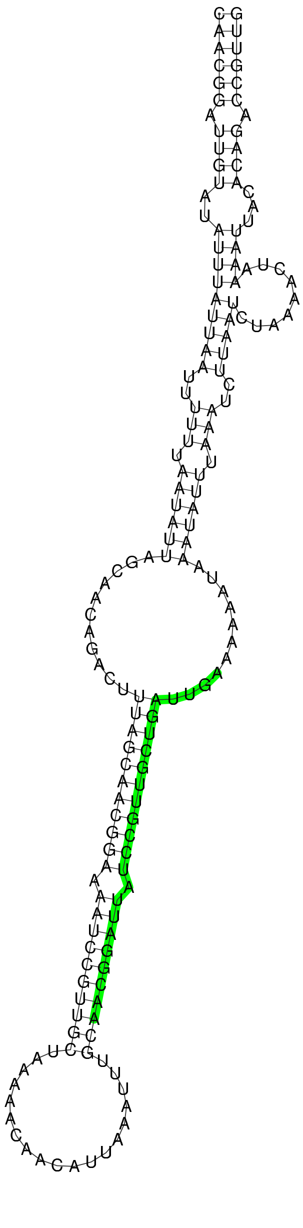

AACGGATTATCCGTTGCTGATTGA_scaffold_7520_1563-1586

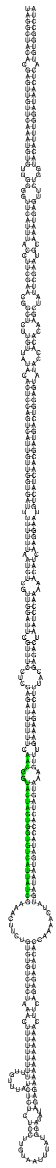

AACGGTACTAGTGGTGTCATTATC_LG_X_15540477-15540500

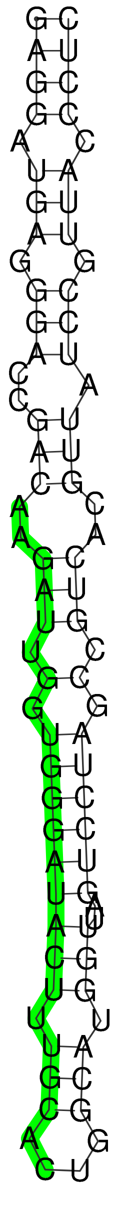

AAGATTGGTGGGATACTTTGCAC_LG_VI_14822763-14822785

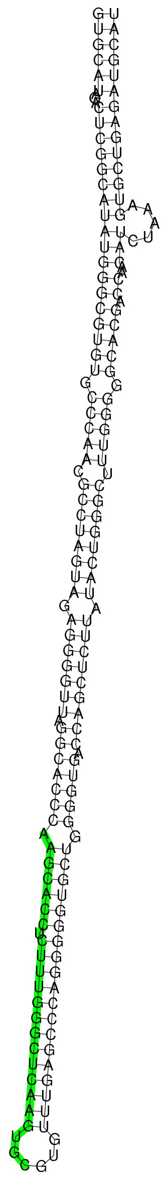

AAGCACCTCTTTGGGCTCAAGTGC_scaffold_14049_995-1018

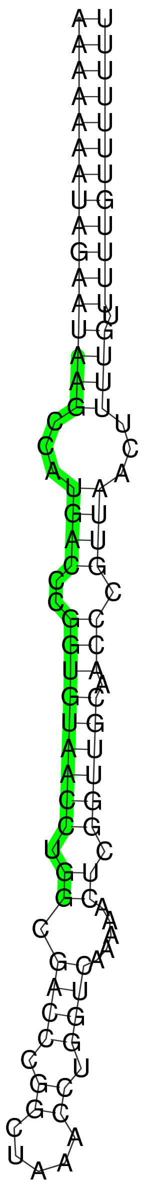

AAGCCATGACCCGGTGTAACCTGG_LG_XII_12974643-12974666

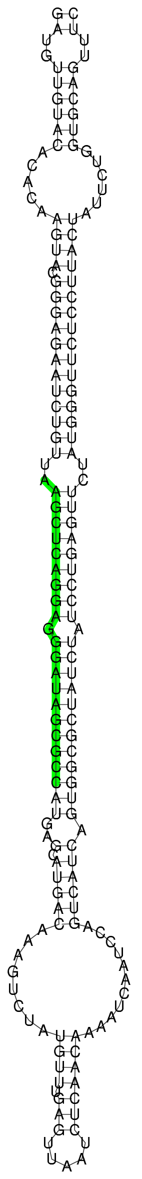

AAGCTCAGGAGGGATAGCGCC_LG_I_20745322-20745342

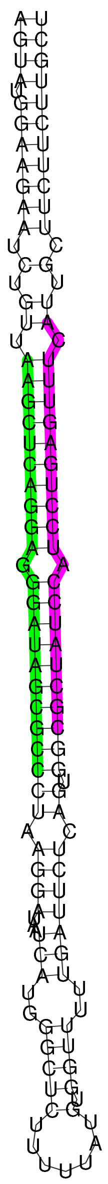

AAGCTCAGGAGGGATAGCGCC_LG_VI_7266292-7266312

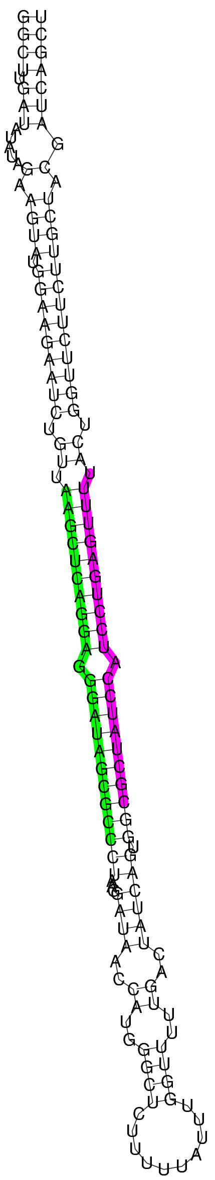

AAGCTCAGGAGGGATAGCGCC_LG_XVI_11828749-11828769

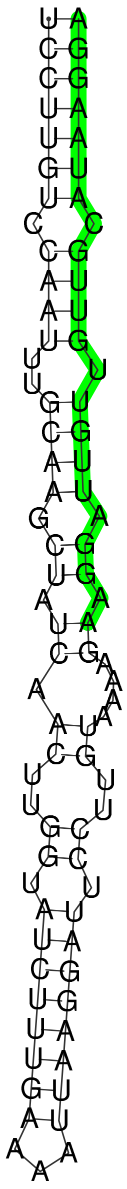

AAGGATTGTTGTTGCATAAGGAAT_scaffold_77_366565-366588

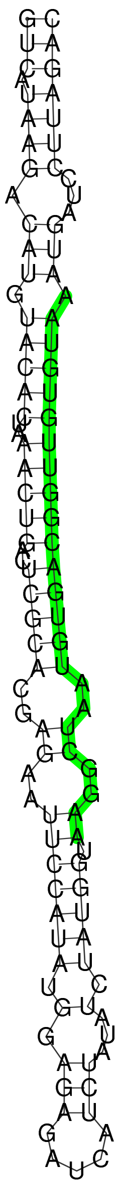

AAGGCTAATGTGACGGTTGTGTAA_LG_XVI_7175434-7175457

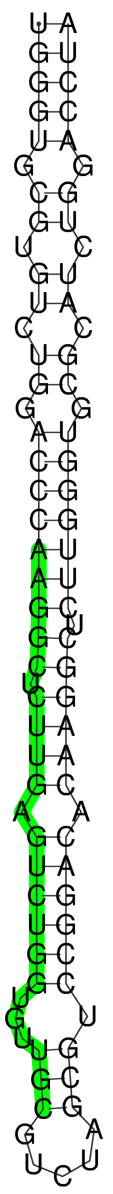

AAGGCTCTTGAGTCTGGTGTTGC_scaffold_201_212279-212301

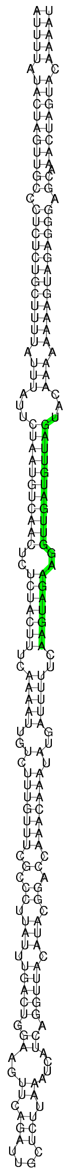

AAGTAGAAGGTTGATGTTAGT_LG_XIX_455313-455333

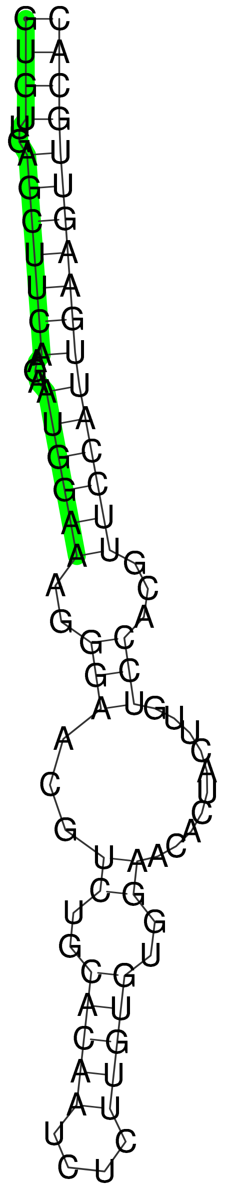

AAGTGTTGAGCTTCAAGAATGGAA_scaffold_1337_6340-6363

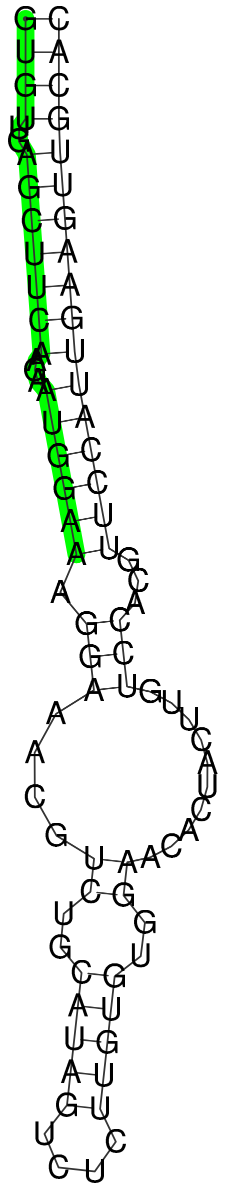

AAGTGTTGAGCTTCAAGAATGGAA_scaffold_6473_1418-1441

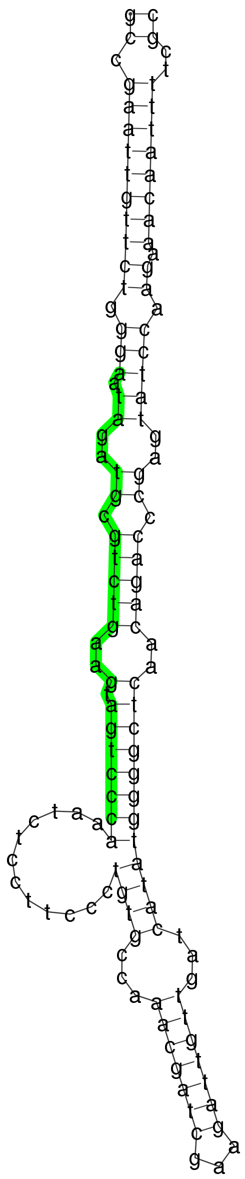

AATAGATGCGTCTGAAGTAGTCCC_LG_II_20980130-20980153

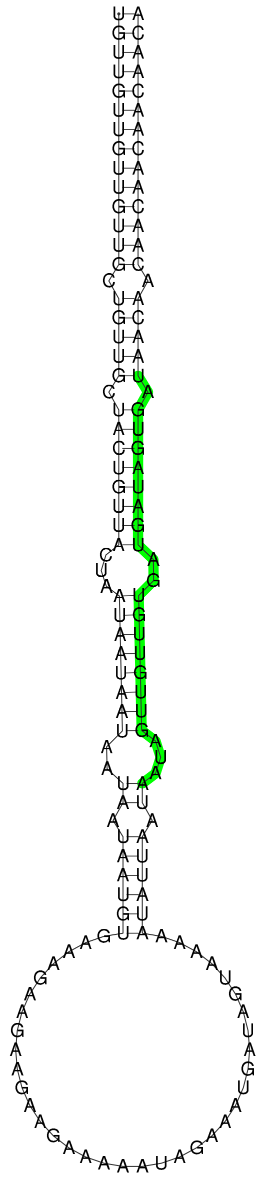

AATAGTTGTTGTGATGATAGTGAT_scaffold_2480_6474-6497

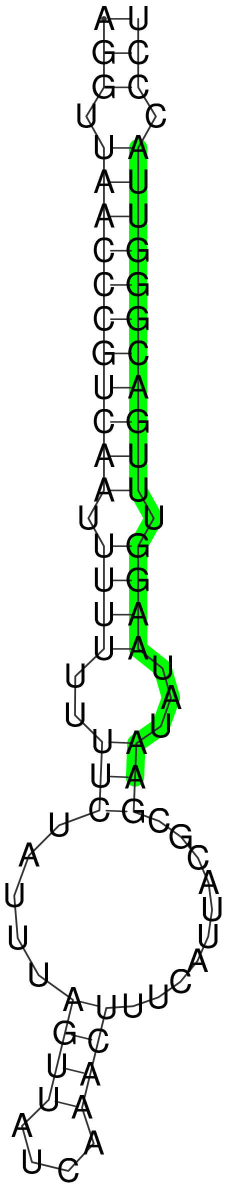

AATATAAGGTTTGACGGGTTA_scaffold_13529_478-498

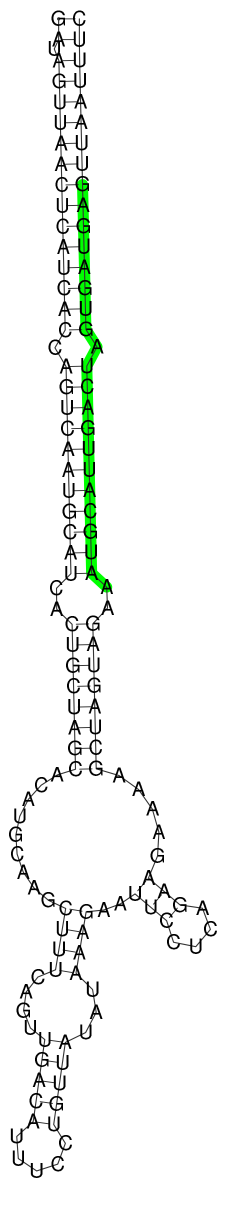

AATGCATTGACTAGTGATGAG_LG_I_17431088-17431108

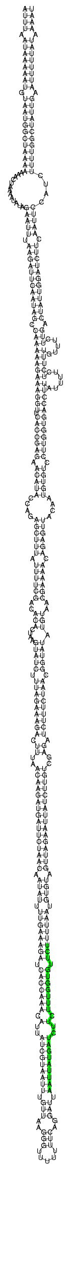

AATTATGATCTTCTTTGGTGTTCT_LG_II_4102440-4102463

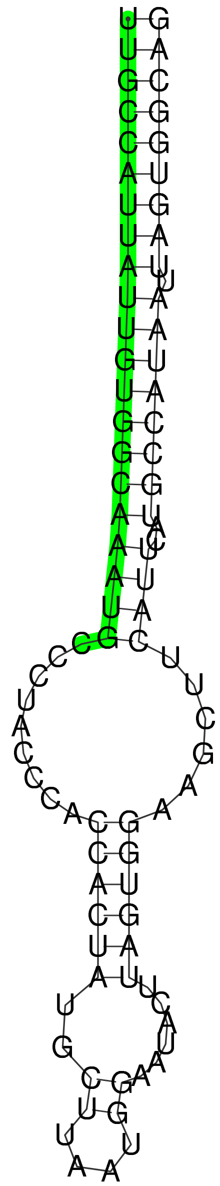

AATTGCCATTATTGTGGCAAATGC_LG_III_14480424-14480447

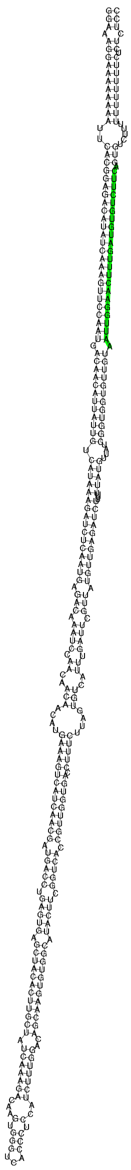

AATTGGAACTTTGATGTGTCTTCA_scaffold_66_909798-909821

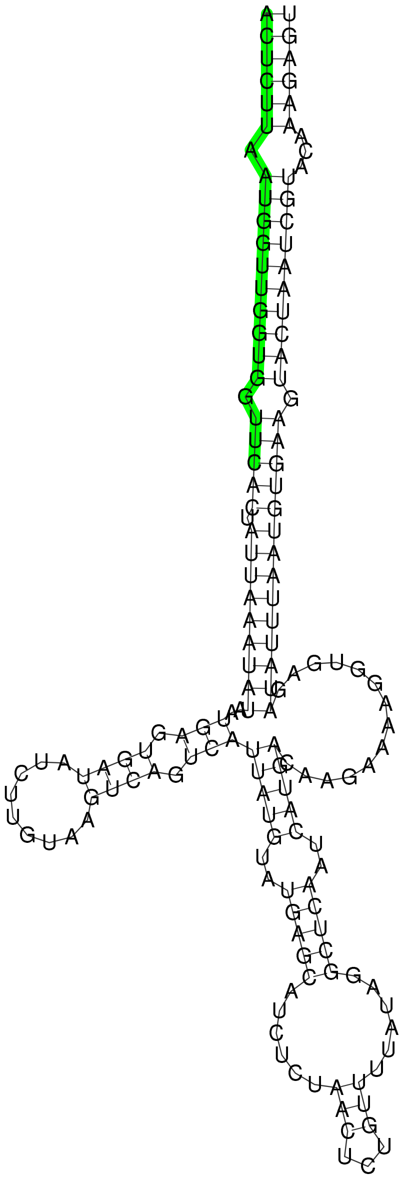

ACAACTCTTAATGGTTGGTGGTTC_LG_XVIII_6172381-6172404

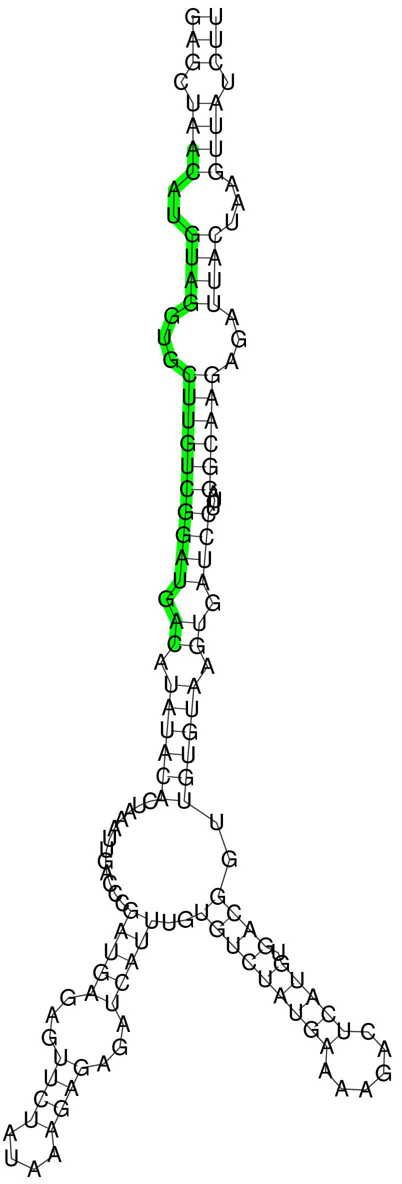

ACATGTAGGTGCTTGTCGGATGAC_LG_VII_10741106-10741129

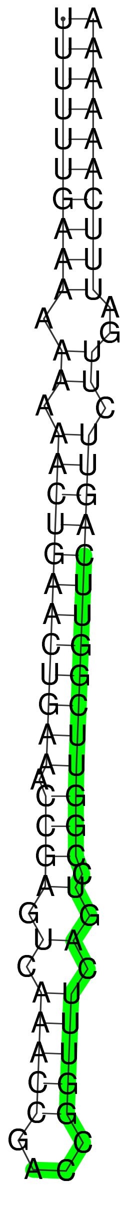

ACCGGTTTCAGTCCGGTTCGGTTC_LG_V_17794360-17794383

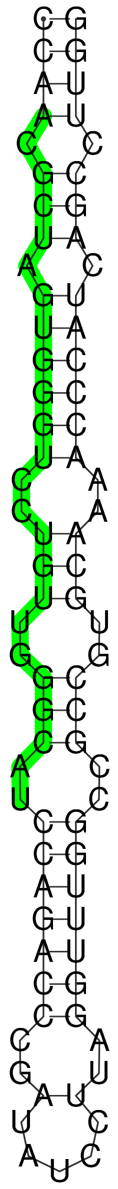

ACGCTAGTGGGTCCTGTTGGGCAT_scaffold_704_14436-14459

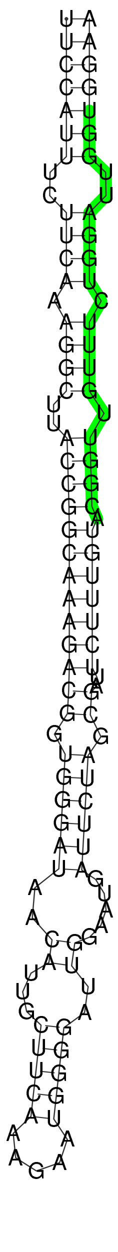

ACGGTTGTTTCTGGATTGGT_LG_I_21244980-21244999

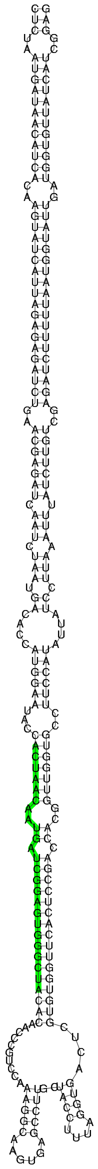

ACTAACAATGATCGGAGTGGGCTA_LG_II_12962784-12962807

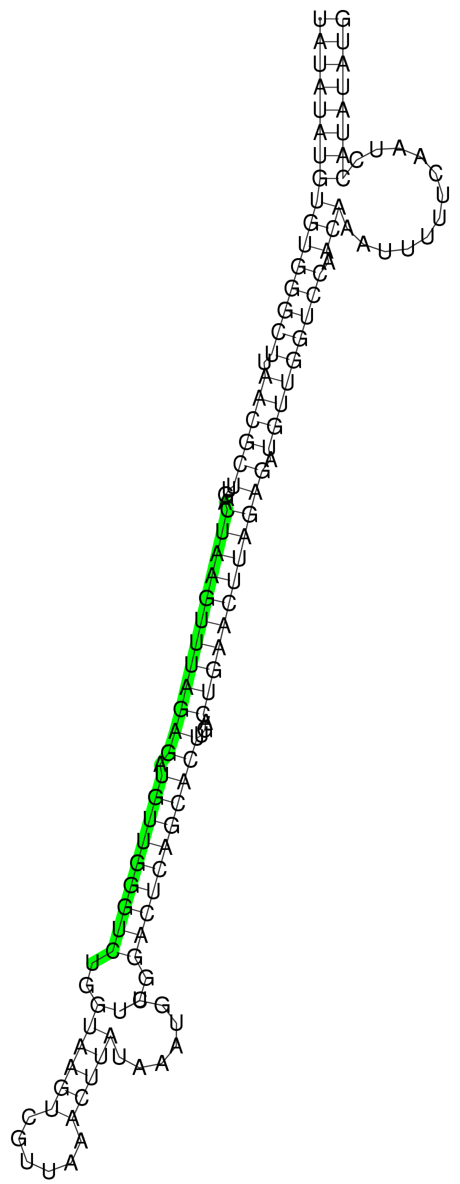

ACTAAGTTTAGAGATGTTGGGTCT_LG_III_13243109-13243132

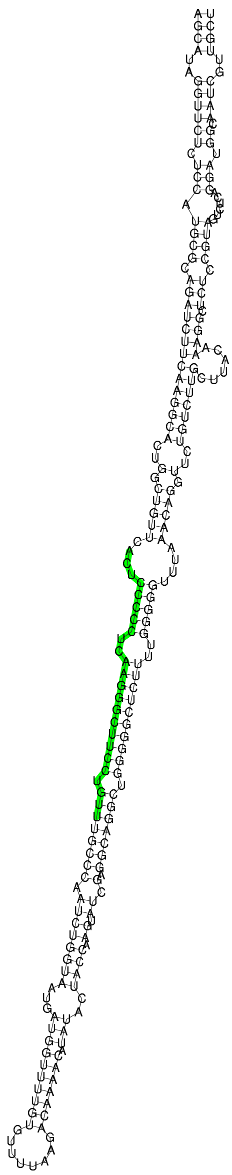

ACTCCCCCTCAAGGGCTTCCTGTT_LG_II_12980901-12980924

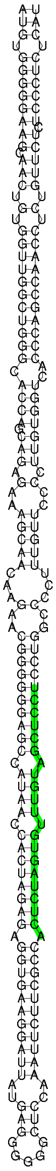

ACTCTAGTGTTTGTAGCTCCT_LG_XIV_11739179-11739199

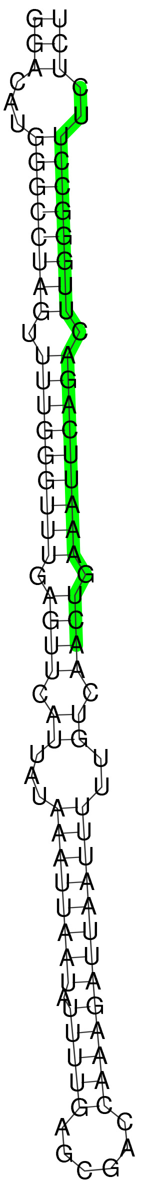

ACTGAAATTCAGACTTGGGCCTTC_LG_II_18377881-18377904

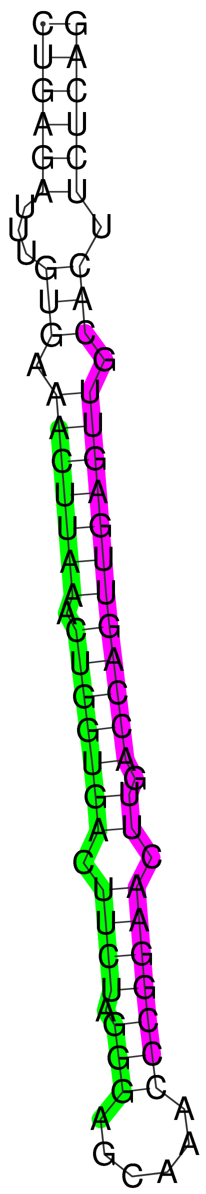

ACTTAAACTGGTGACTTCTAGGGA_LG_VI_15052298-15052321

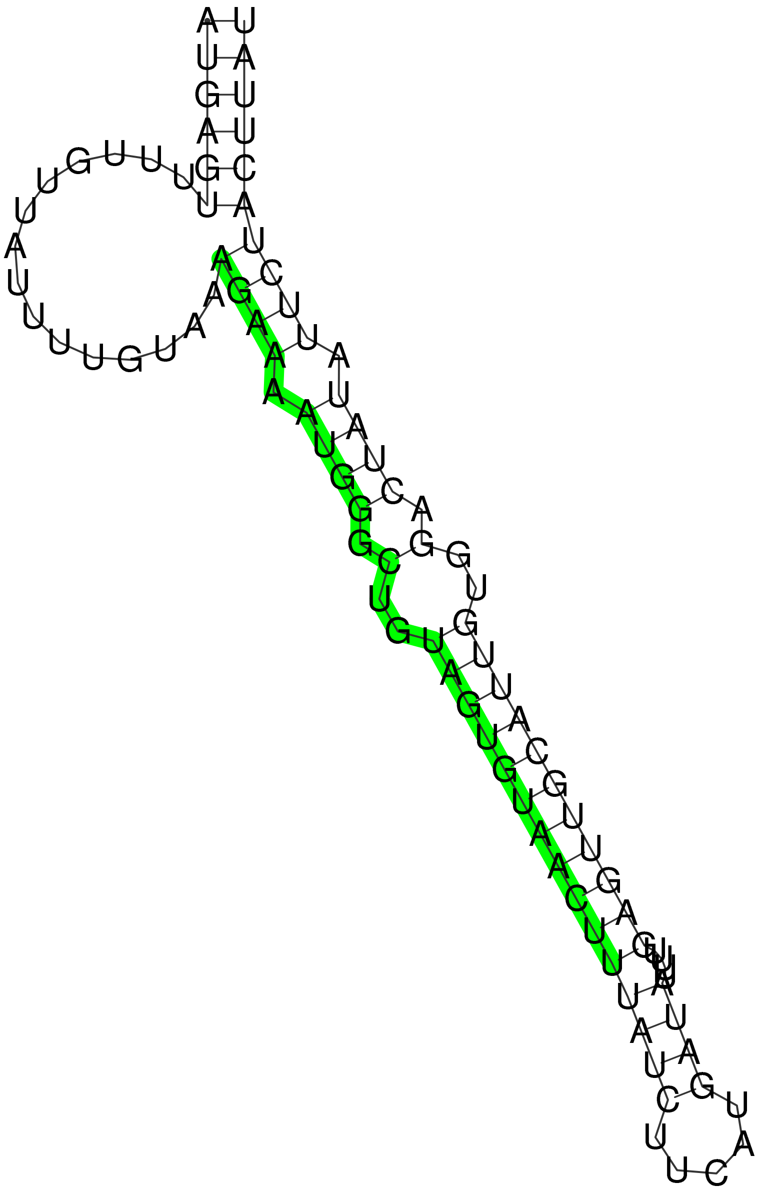

AGAAAATGGGCTGTAGTGTAACTT_LG_III_18780951-18780974

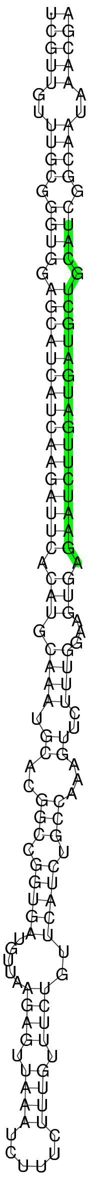

AGAATCTTGATGATGCTGCAT_LG_IX_11209231-11209251

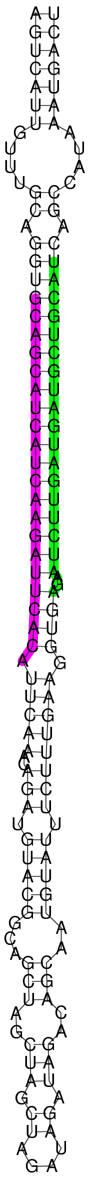

AGAATCTTGATGATGCTGCAT_LG_X_19299613-19299633

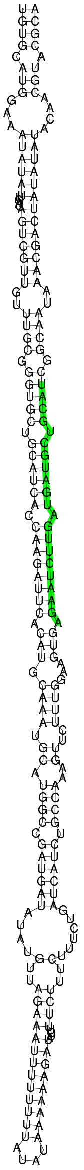

AGAATCTTGATGATGCTGCAT_scaffold_66_1988743-1988763

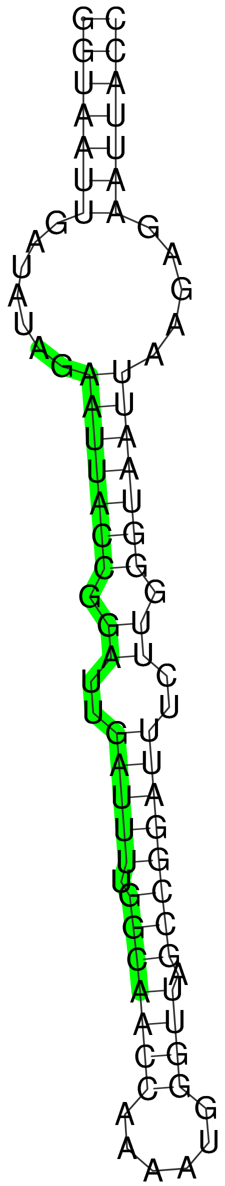

AGAATTACCGGATTGATTTTGGCA_LG_I_18696896-18696919

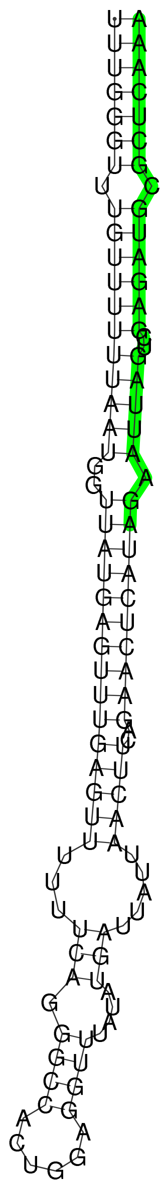

AGAATTAGTCGAGATGCGCTCAAA_LG_XII_10532736-10532759

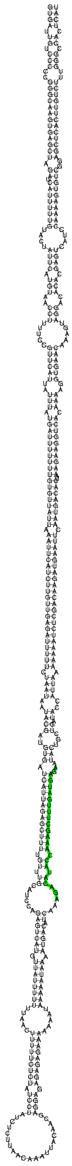

AGACATACCAAAGCTTTGATGAGA_LG_II_1150598-1150621

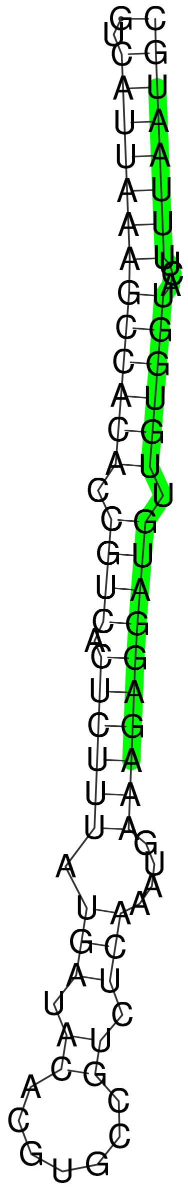

AGAGGATGTTGTGGTACTTTTAAT_LG_IV_4293708-4293731

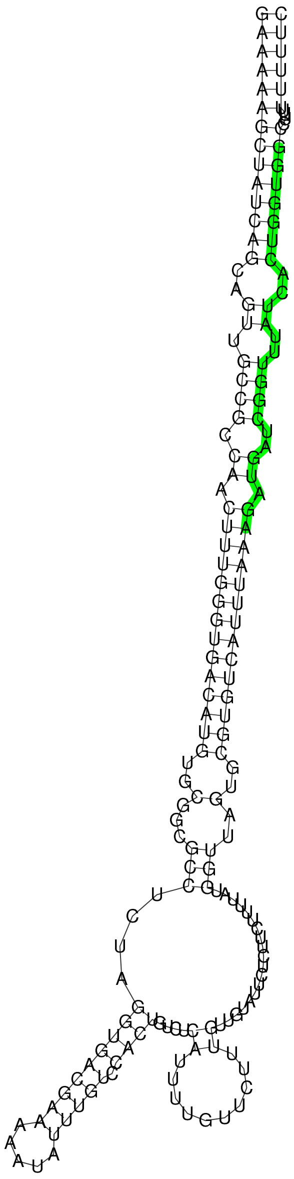

AGATGATCGGTTTATCACTGGTGG_LG_XV_7227746-7227769

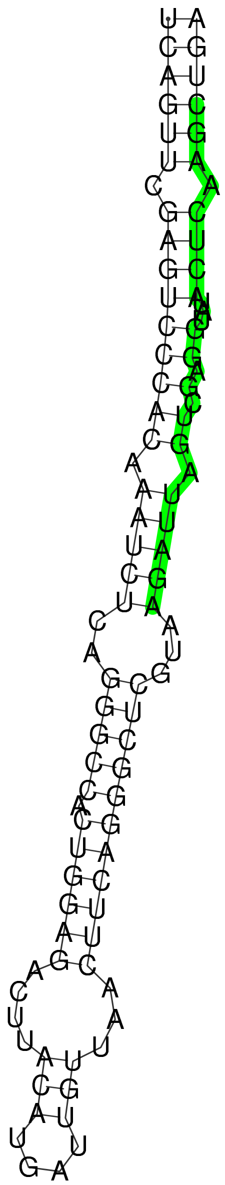

AGATTAGTCGAGGTATACTCAAGC_scaffold_155_434914-434937

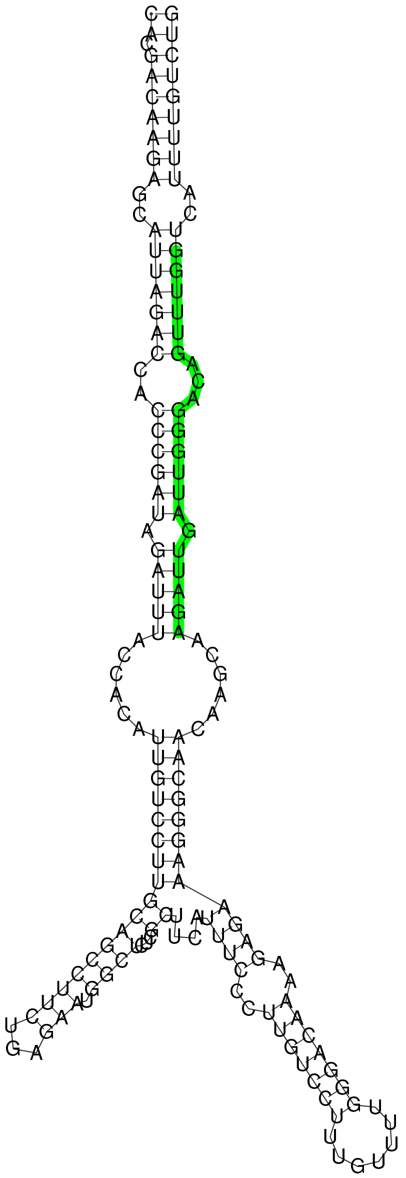

AGATTGATTGGGACAGTTTGG_LG_VII_95672-95692

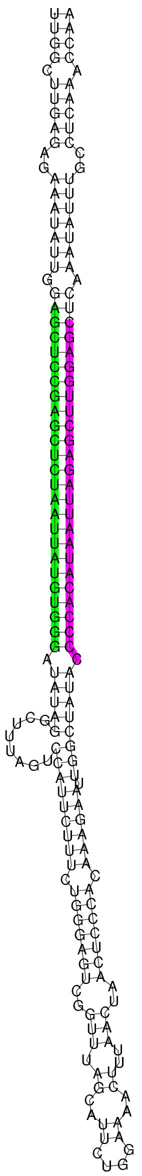

AGCTCCGAGCTCTAATTATGTGGG_LG_II_22547443-22547466

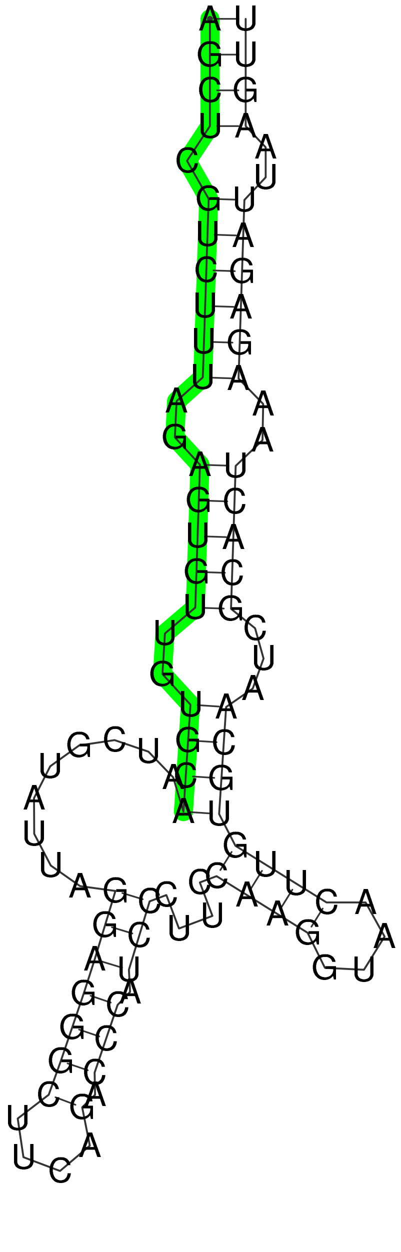

AGCTCGTCTTTAGAGTGTTGTGCA_LG_IX_9564913-9564936

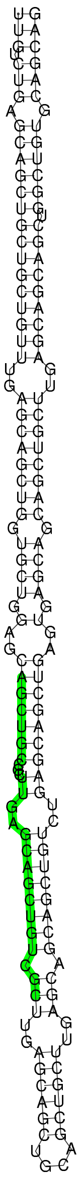

AGCTGCCGCTTGAGCAGCTGTCGC_scaffold_155_508600-508623

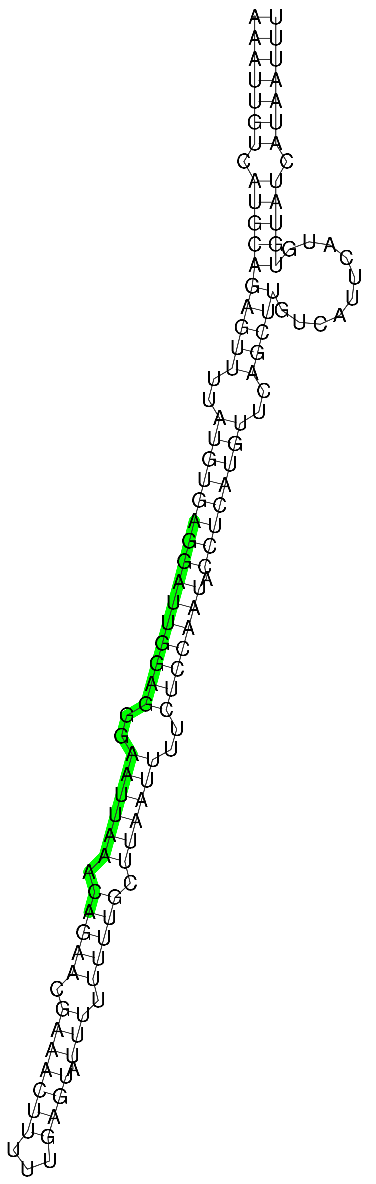

AGGATTGGAGGGAATTAAACA_LG_XII_12413507-12413527

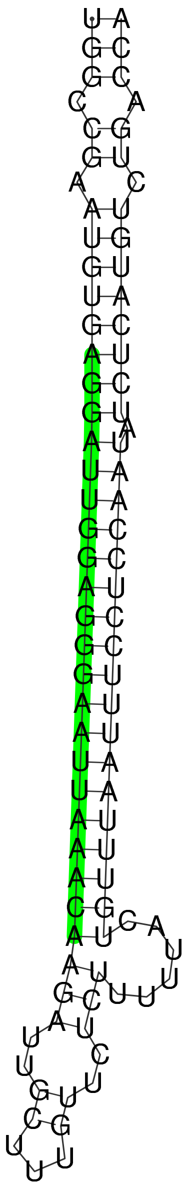

AGGATTGGAGGGAATTAAACA_LG_X_19416768-19416788

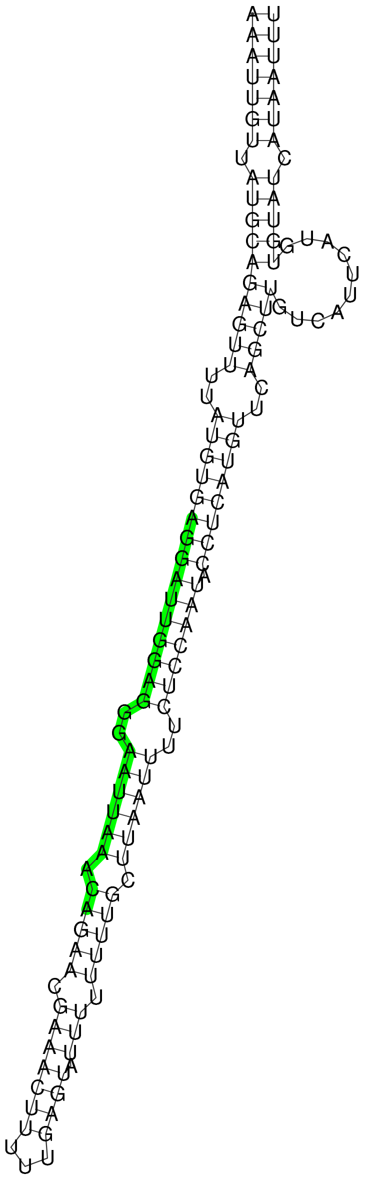

AGGATTGGAGGGAATTAAACA_scaffold_232_287536-287556

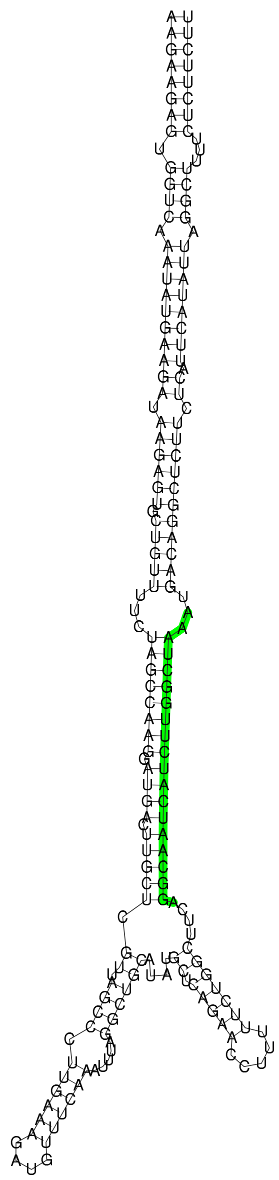

AGGCAATCATCTTGGCTAAA_LG_VII_6121337-6121356

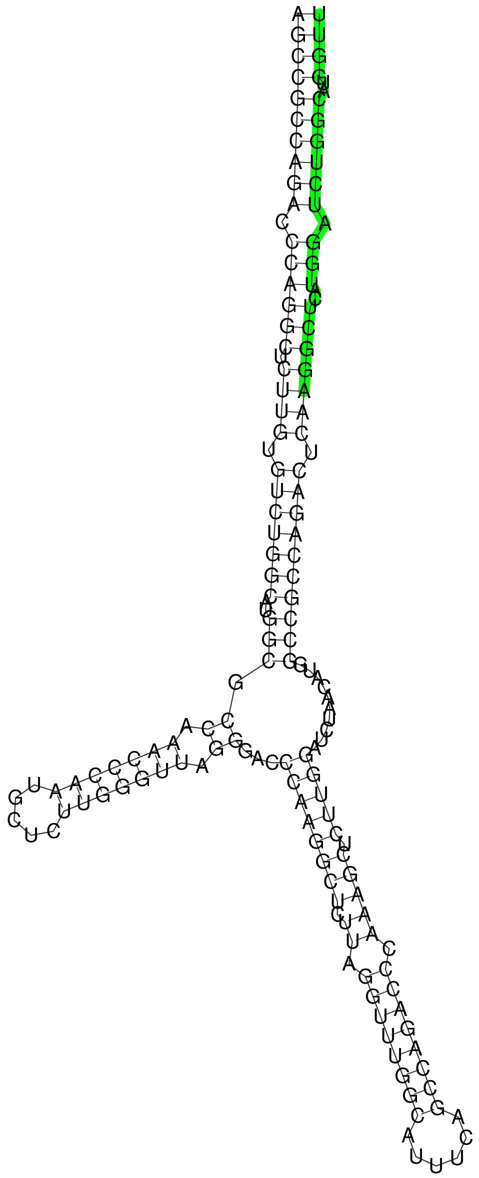

AGGCTCATGGATCTGGCATGGTTC_scaffold_44_442839-442862

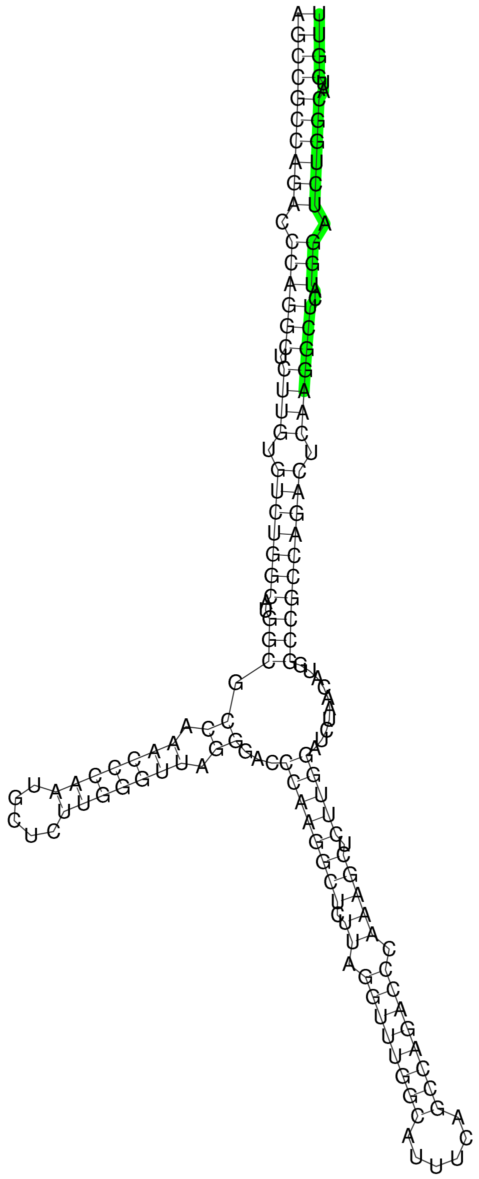

AGGCTCATGGATCTGGCATGGTTC_scaffold_4950_929-952

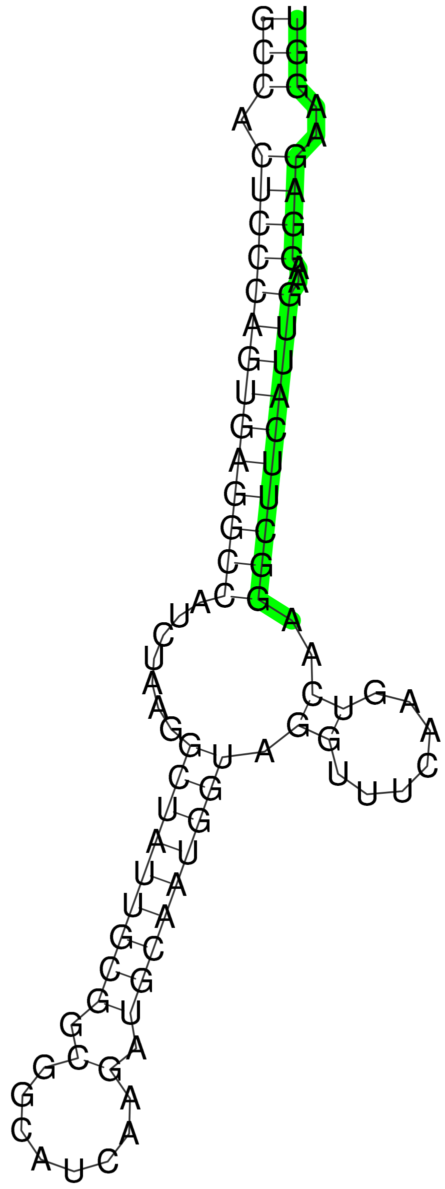

AGGCTTCATTGAAGGAGAAGGT_scaffold_145_107321-107342

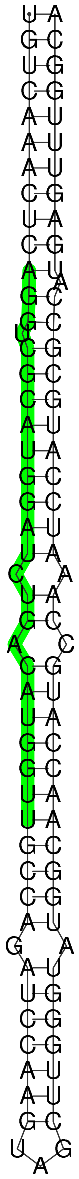

AGGTCGCATGGATCTGACATGGTT_LG_V_4919051-4919074

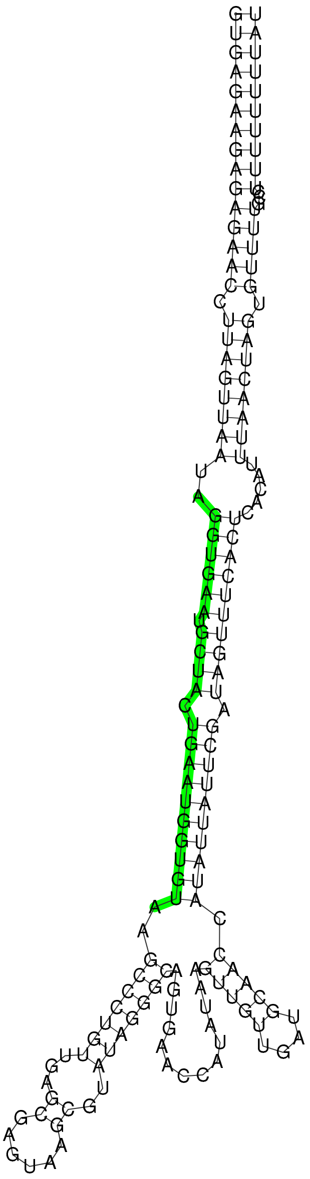

AGGTGAATGCTACTGAATGGTGTA_LG_X_31374-31397

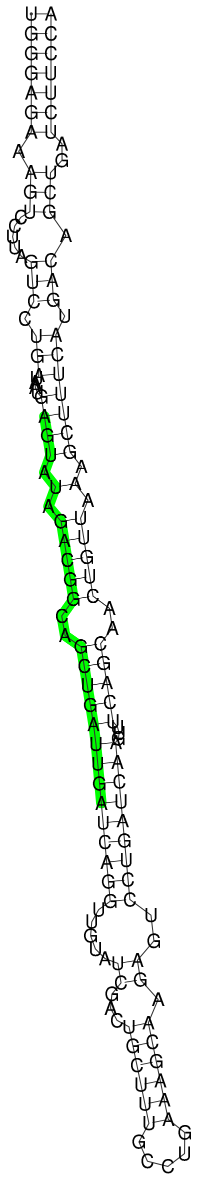

AGTATAGACGGCAGCTGATTGA_LG_I_20385506-20385527

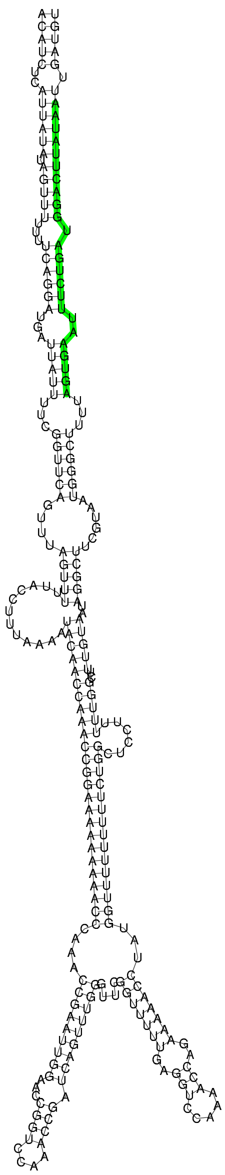

AGTGAATTTCTGATGGACTTATAA_LG_XI_7704439-7704462

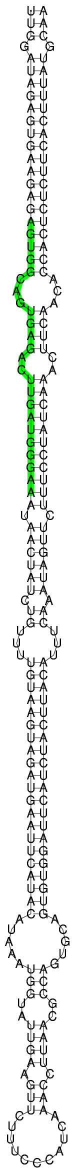

AGTGGCAGTGAGACTTGATGGGAA_scaffold_86_399999-400022

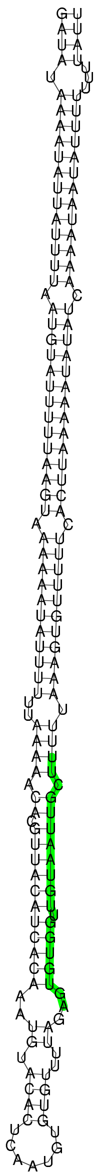

AGTGTGGTTGTAATTGCTT_scaffold_201_293334-293352

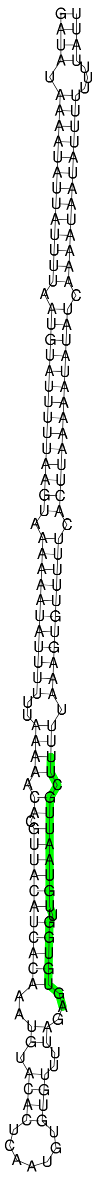

AGTGTGGTTGTAATTGCTT_scaffold_762_17485-17503

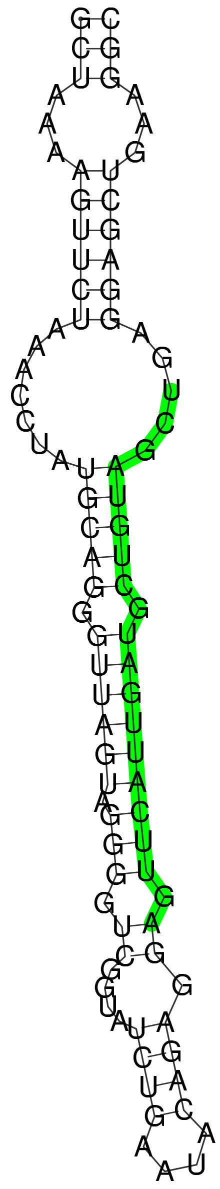

AGTTCATTGATGCTGTAGCT_LG_VIII_4984966-4984985

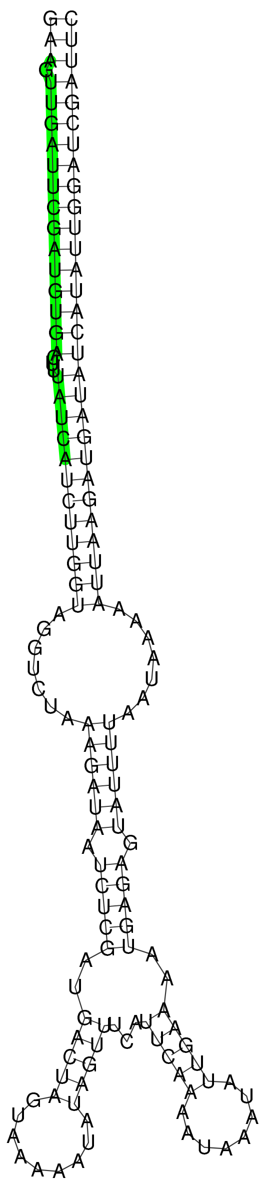

AGTTGATTCGATGTGACTTTATCA_LG_XVI_7434601-7434624

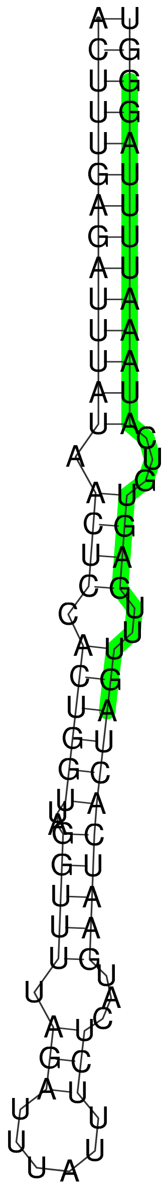

AGTTTGAGTGTCATAAATTTTAGG_LG_III_9741274-9741297

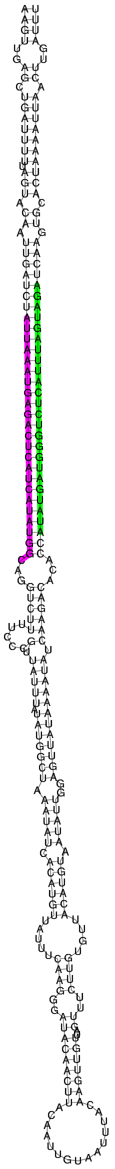

ATATGATGGGTCTCATTTAGTAGA_LG_XIV_2363320-2363343

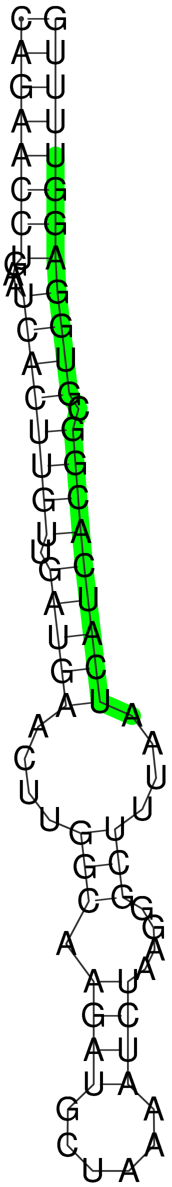

ATCATCACGGCGTGGAGGT_LG_I_1802474-1802492

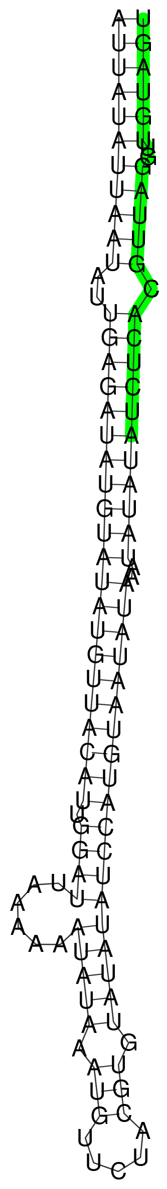

ATCTCACGTTAGGTTGTAGTCAGG_LG_X_3831981-3832004

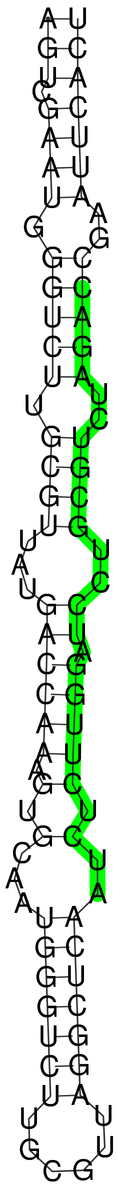

ATCTCTTGGATCCTGCGTCTAGAC_LG_IX_7987059-7987082

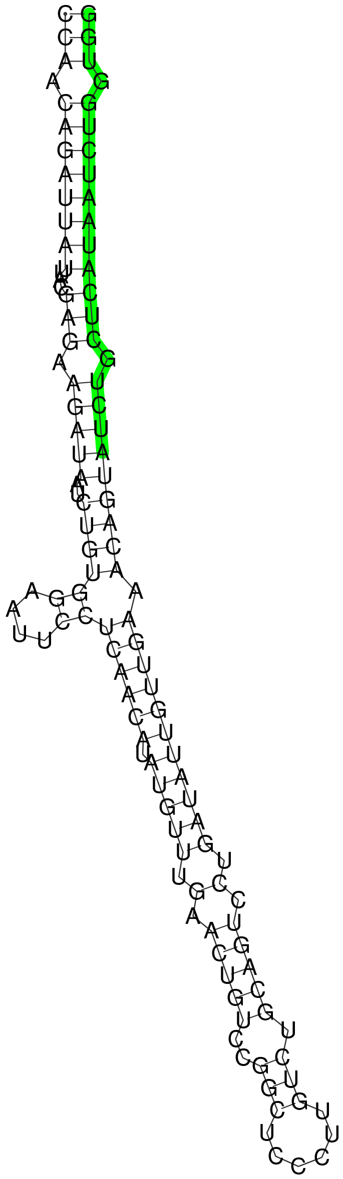

ATCTGCTCATAATCTGGTGGTAAA_LG_XIX_9765425-9765448

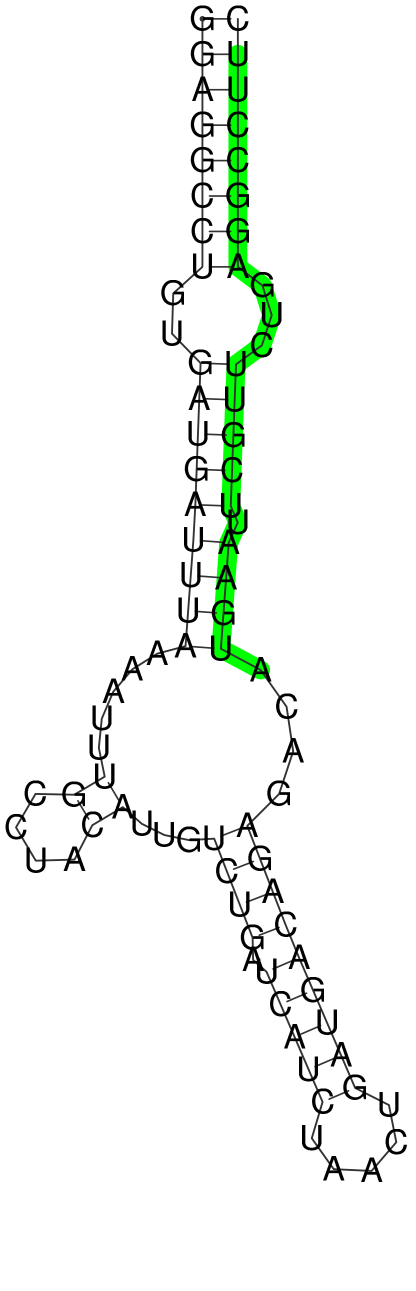

ATGAATTCGTTCTGAGGCCTT_LG_V_2608932-2608952

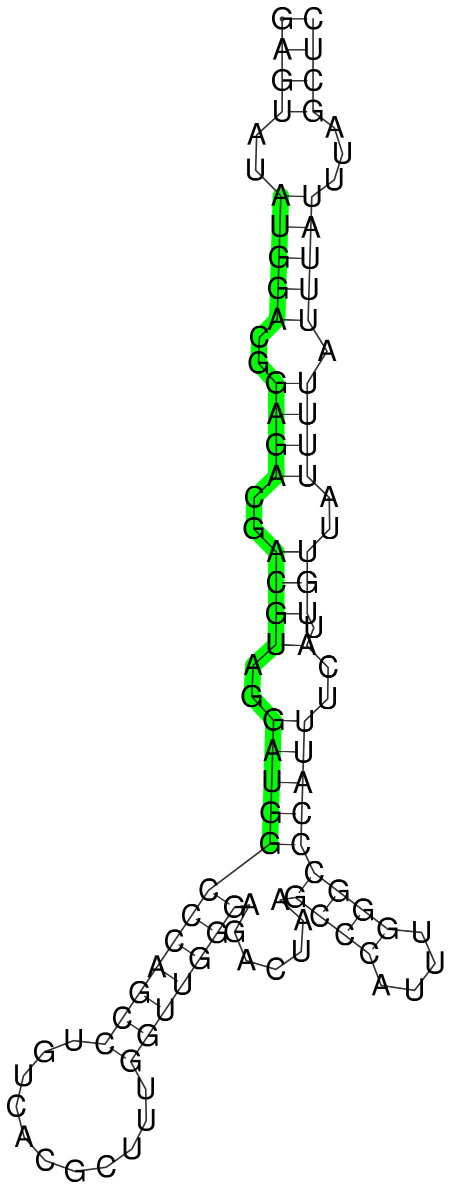

ATGGACGGAGACGACGTAGGATGG_scaffold_8879_909-932

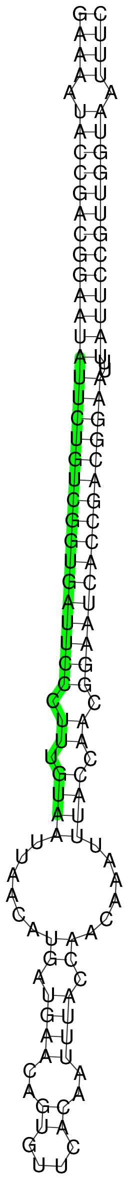

ATTCTGTCGGTGATTCCCTTTGTA_scaffold_3344_4773-4796

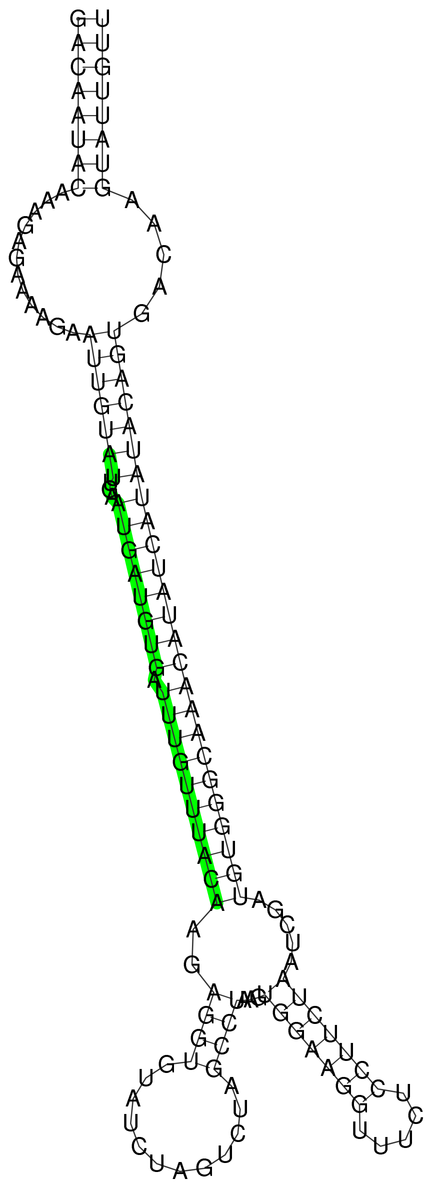

ATTGAATGATGTGATTTGTTTACA_scaffold_163_128560-128583

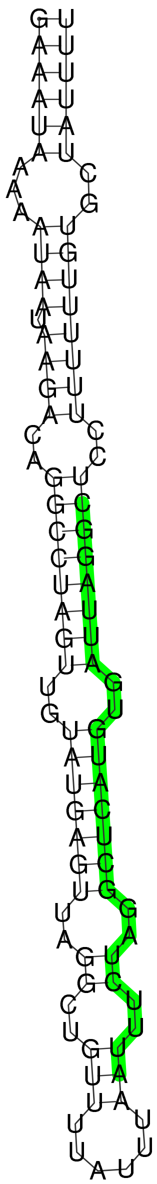

ATTTCTAGGCTCATGTGATTAGGC_LG_XVI_2192534-2192557

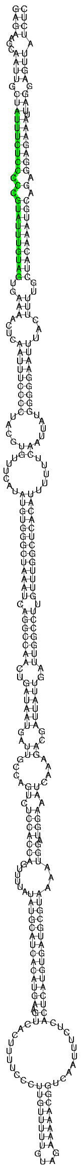

ATTTCTCCCCCGTATTTGTAG_LG_IX_11361312-11361332

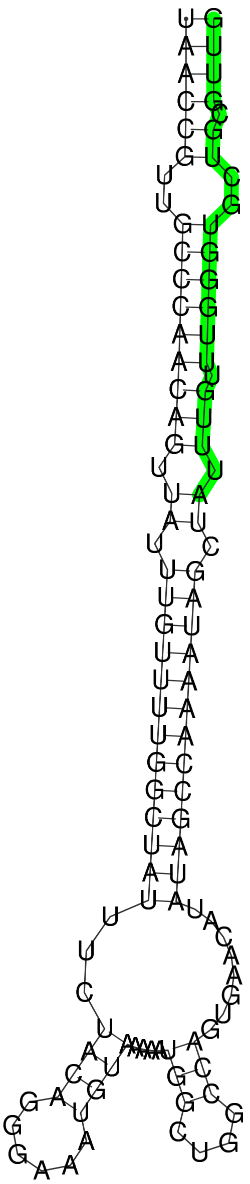

ATTTGTTTGGGTGCTGCGTTGGAG_LG_IX_7117475-7117498

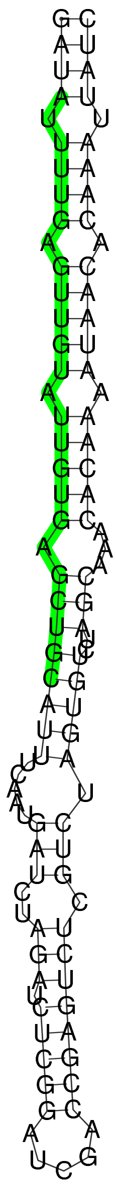

ATTTTGAGTTGTATTGTGAGCTGC_LG_VIII_2970005-2970028

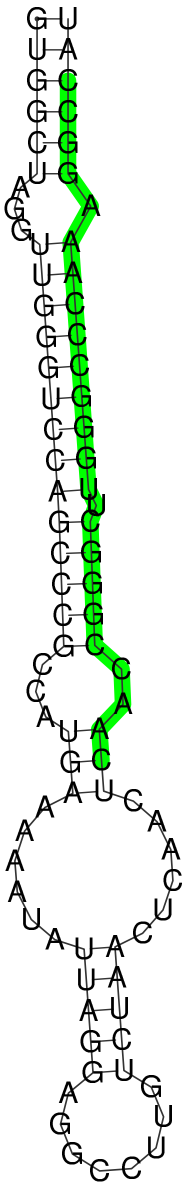

CAACCGGGCTTGGGCCCAAAGGCC_LG_I_30228661-30228684

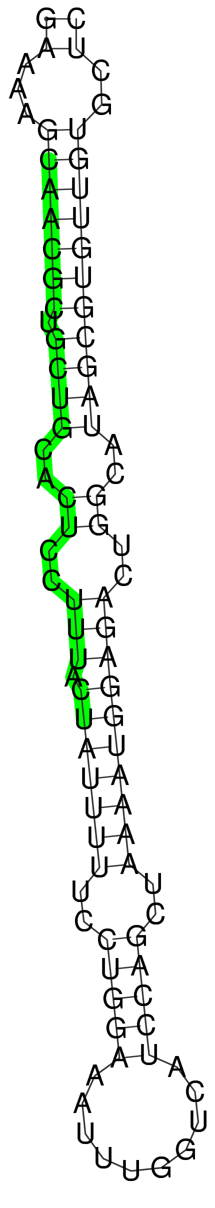

CAACGCTGCTGCACTCCTTTACT_LG_X_17189635-17189657

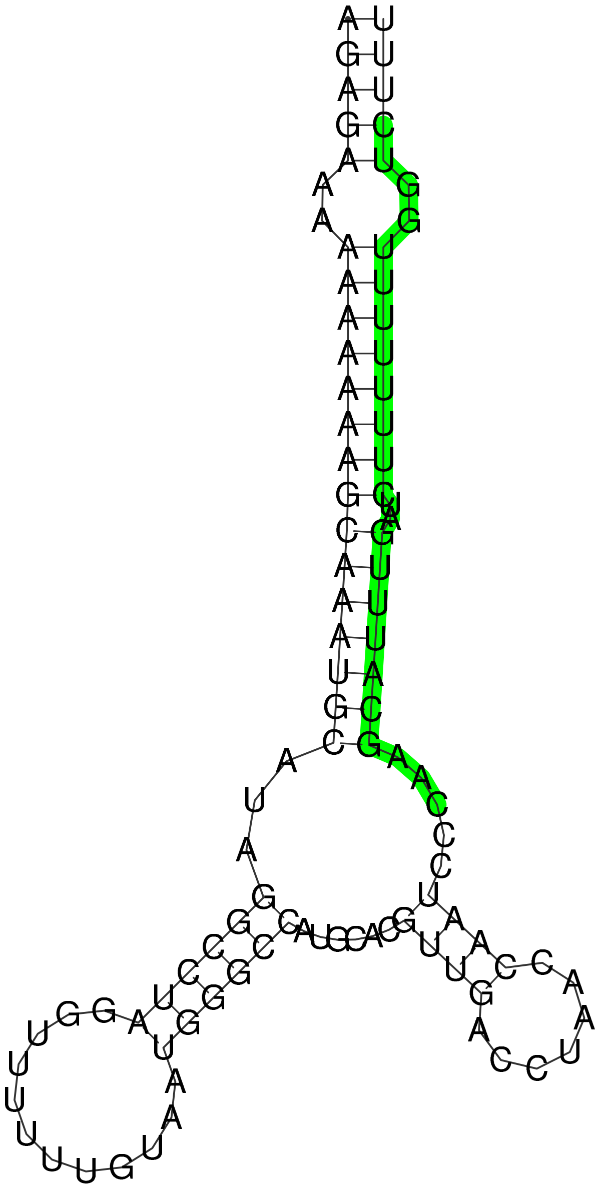

CAAGCATTTGATCTTTTTTTGGTC_LG_XIV_5473797-5473820

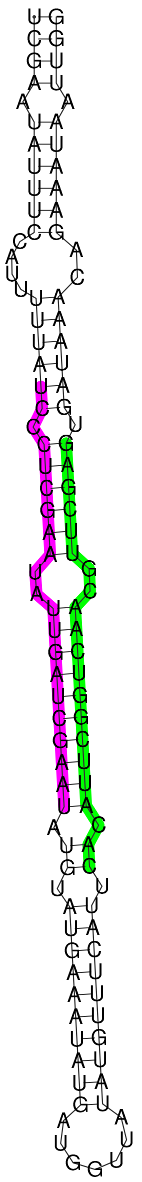

CACATTCGGTCAACGTTCGAG_LG_X_10569469-10569489

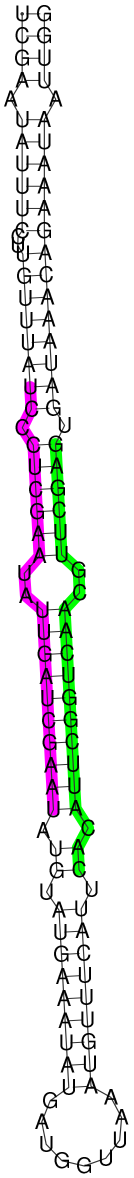

CACATTCGGTCAACGTTCGAG_scaffold_8959_1109-1129

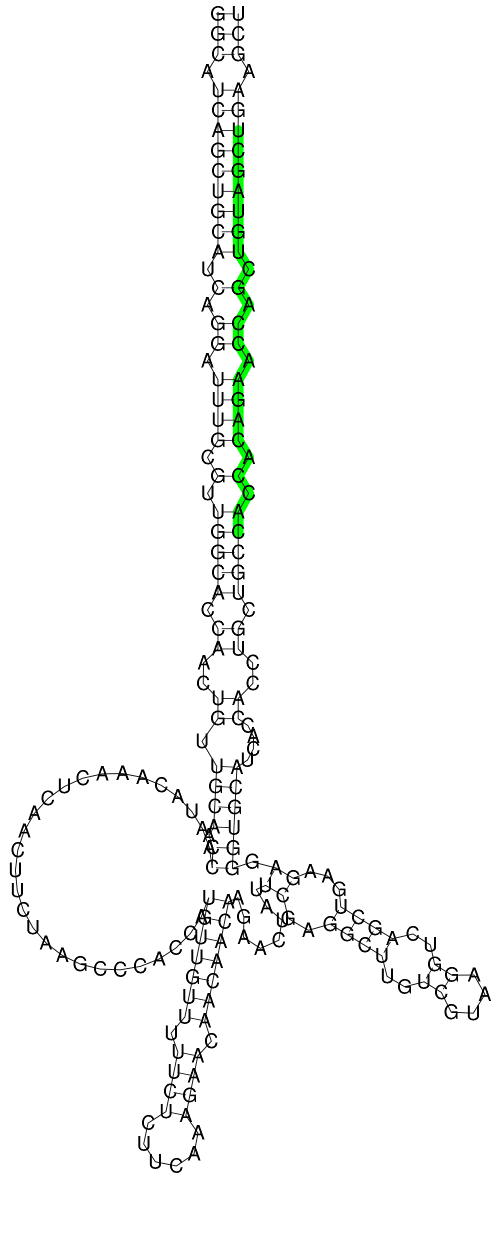

CACCACAGAACCAGCTGTAGCT_LG_II_23468298-23468319

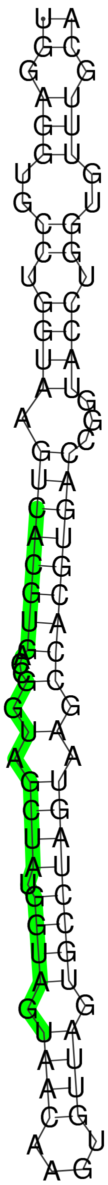

CACGTGACCGGTAGCTATGGTAGT_LG_VIII_12653588-12653611

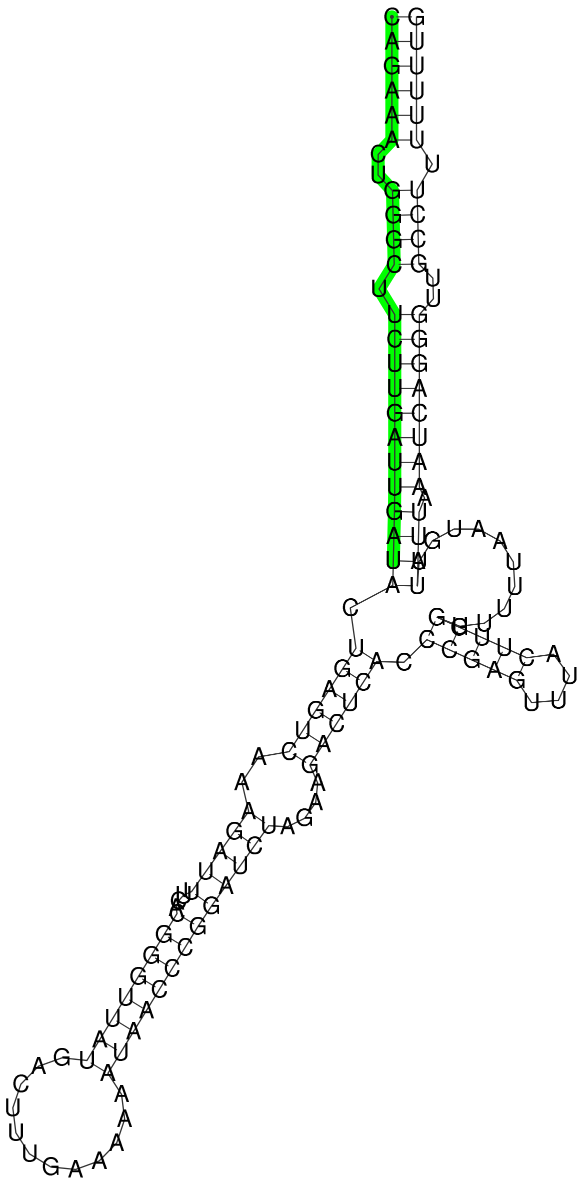

CAGAAACTGGGCTTCTTGATTGAT_LG_X_13663228-13663251

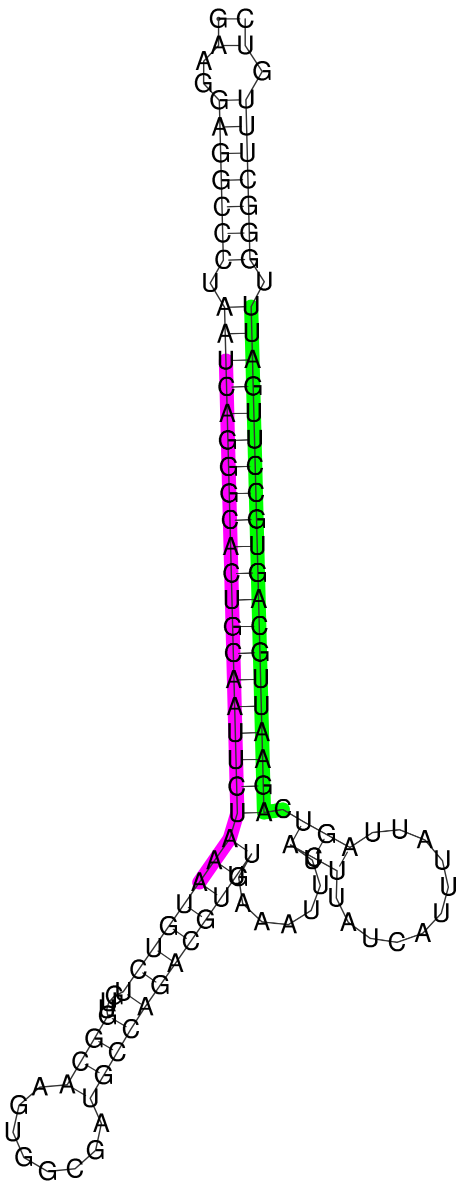

CAGAATTGCAGTGCCTTGATT_LG_XIII_11828114-11828134

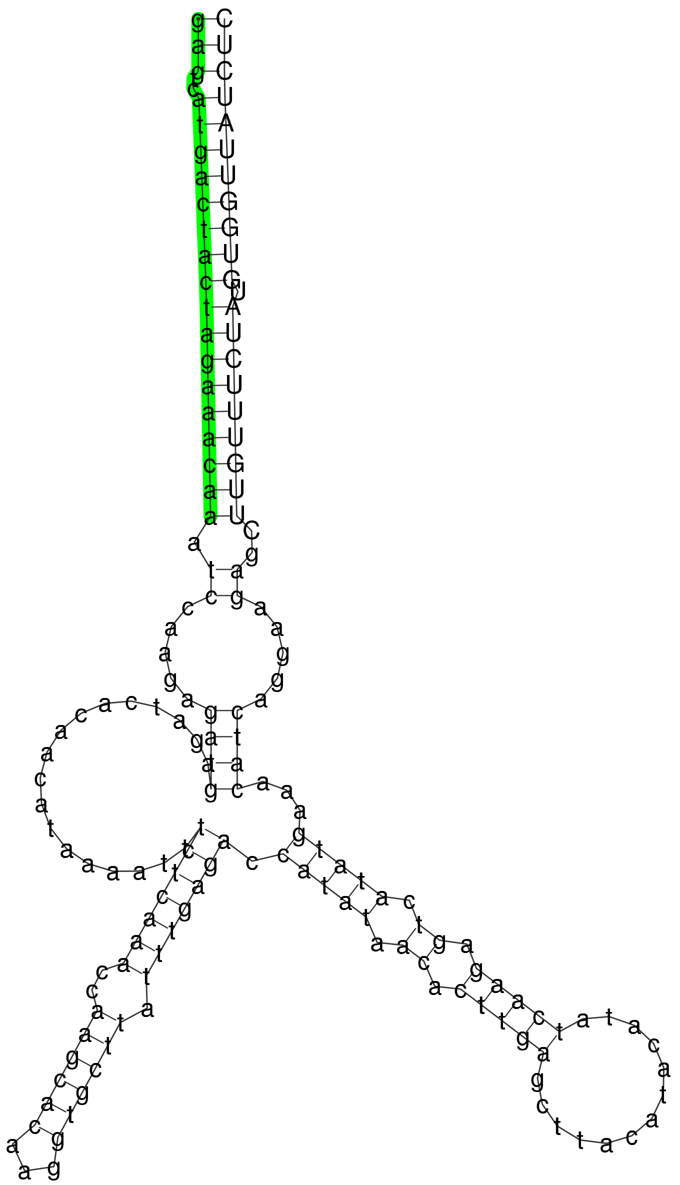

CAGAGTCATGACTACTAGAAACAA_scaffold_18412_584-607

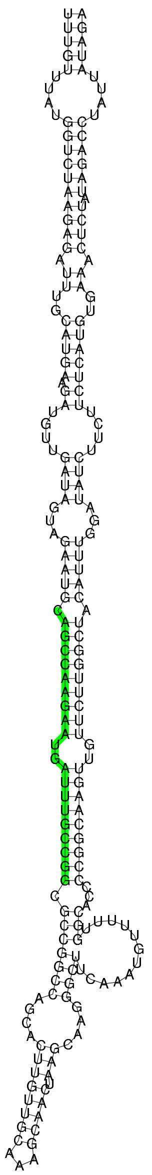

CAGCCAAGAATGATTTGCCGG_LG_XVIII_13381125-13381145

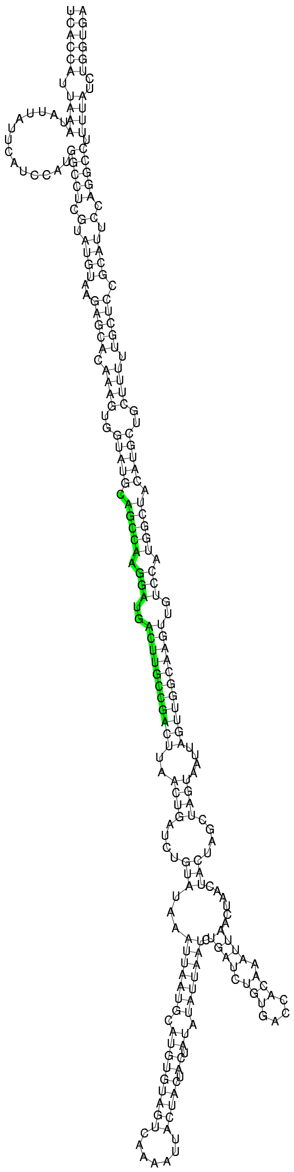

CAGCCAAGGATGACTTGCCGA_LG_I_83281-83301

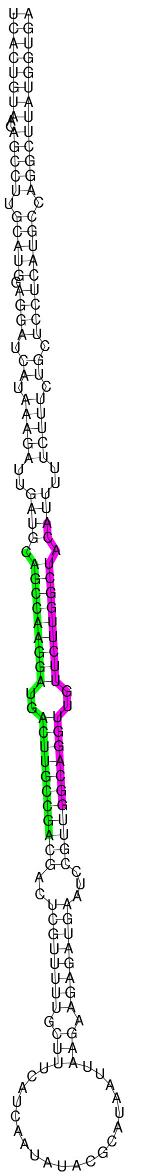

CAGCCAAGGATGACTTGCCGA_LG_XIII_4493432-4493452

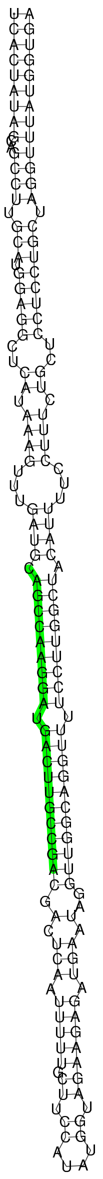

CAGCCAAGGATGACTTGCCGA_LG_XIX_4784314-4784334

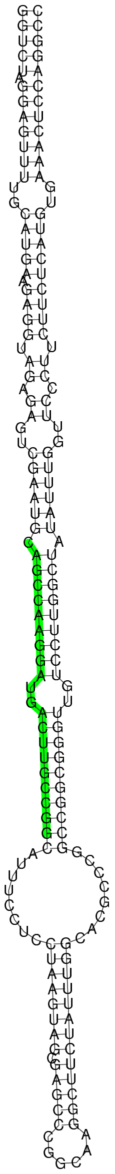

CAGCCAAGGATGACTTGCCGG_LG_VI_5032034-5032054

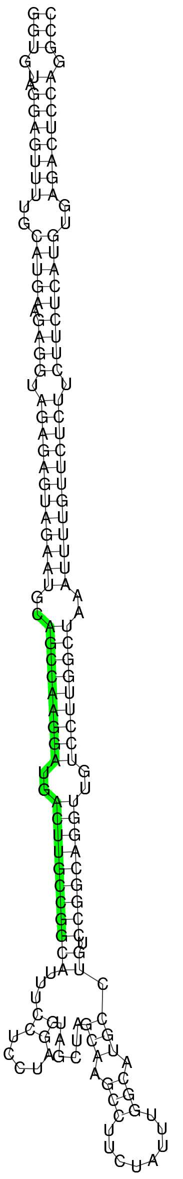

CAGCCAAGGATGACTTGCCGG_LG_XVIII_13384441-13384461

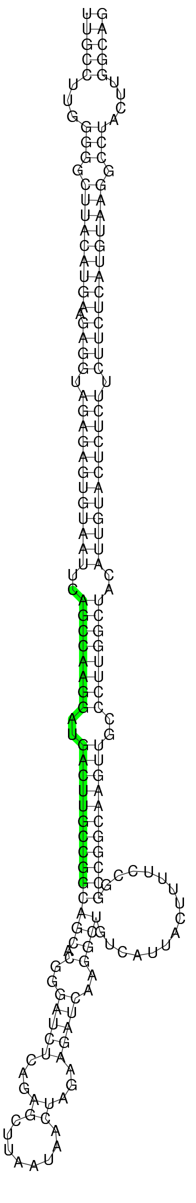

CAGCCAAGGATGACTTGCCGG_scaffold_147_428599-428619

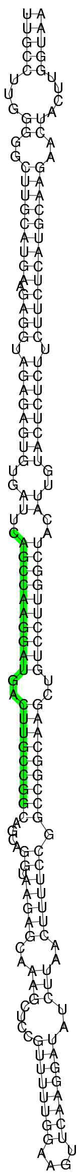

CAGCCAAGGATGACTTGCCGG_scaffold_193_175021-175041

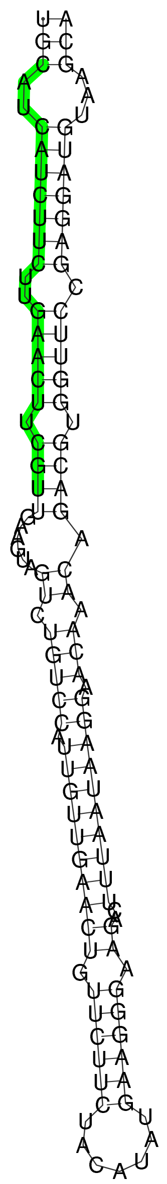

CATCATCTTCTTGAACTTCGT_LG_X_13642315-13642335

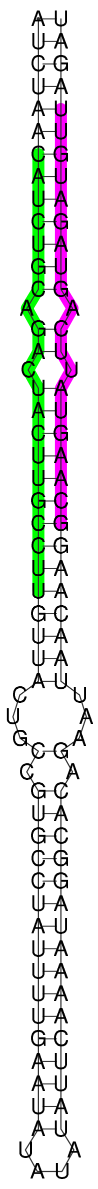

CATCTGCAGACTACTTGCCTT_LG_XIV_3657623-3657643

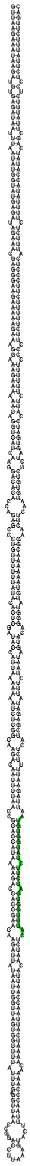

CATTTGGTGATCTTCTATGGTGTA_LG_IV_13376912-13376935

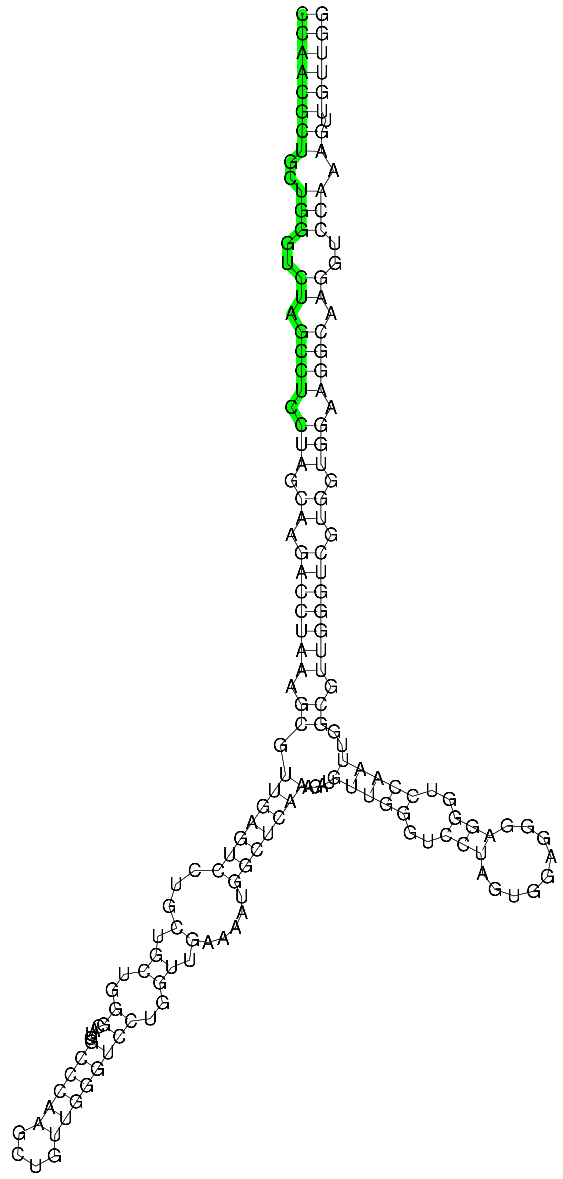

CCAACGCTGCTGGGTCTAGCCTCC_LG_XIV_6019248-6019271

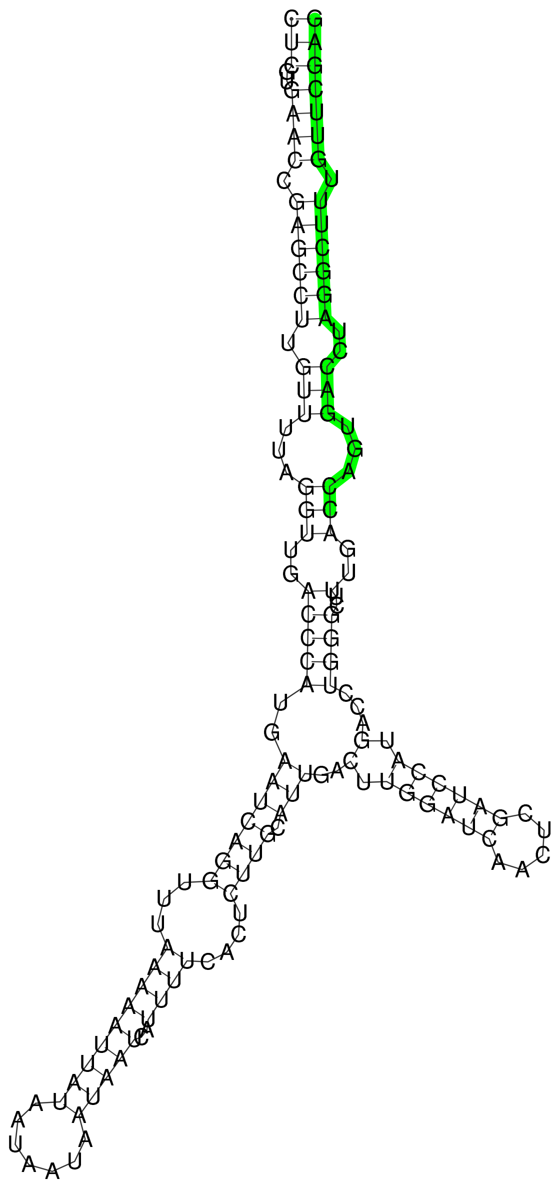

CCAGTGACCTAGGCTTTGTTCGAG_LG_XIX_8374887-8374910

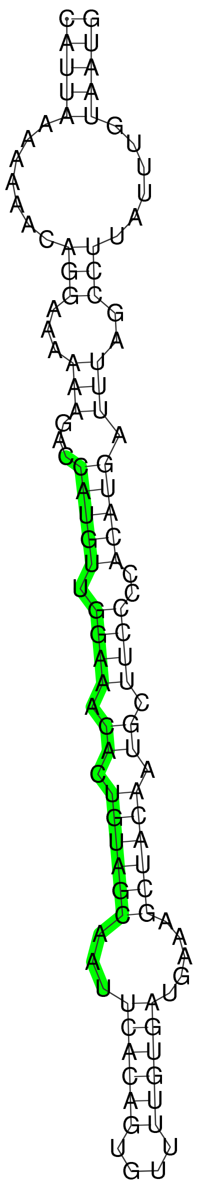

CCATGTTGGAAACACTGTAGCAAT_LG_IV_12516154-12516177

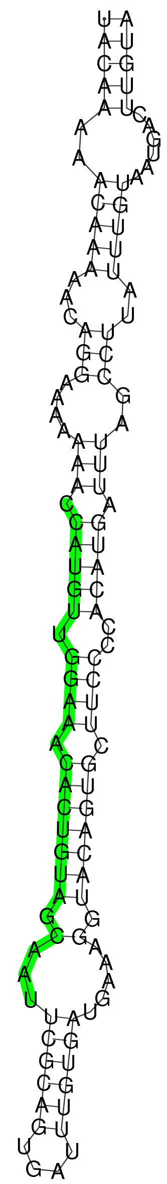

CCATGTTGGAAACACTGTAGCAAT_LG_XII_3558732-3558755

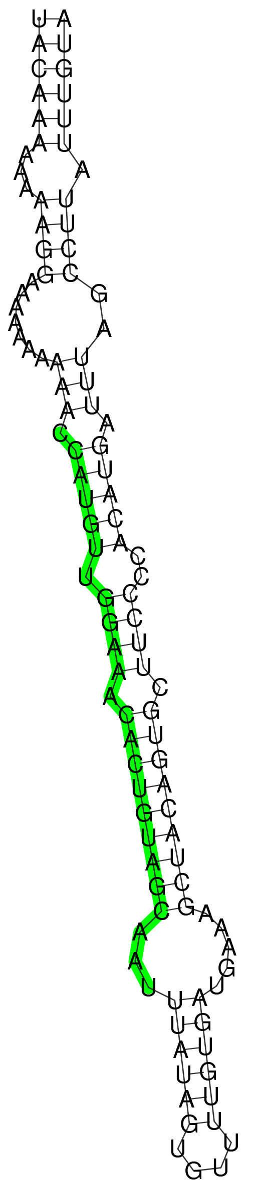

CCATGTTGGAAACACTGTAGCAAT_LG_XIV_1812360-1812383

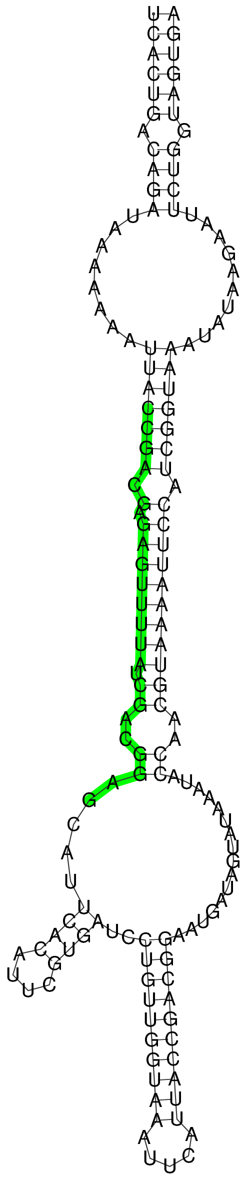

CCGACGAGAGTTTTATCGACGGAG_LG_VI_2310962-2310985

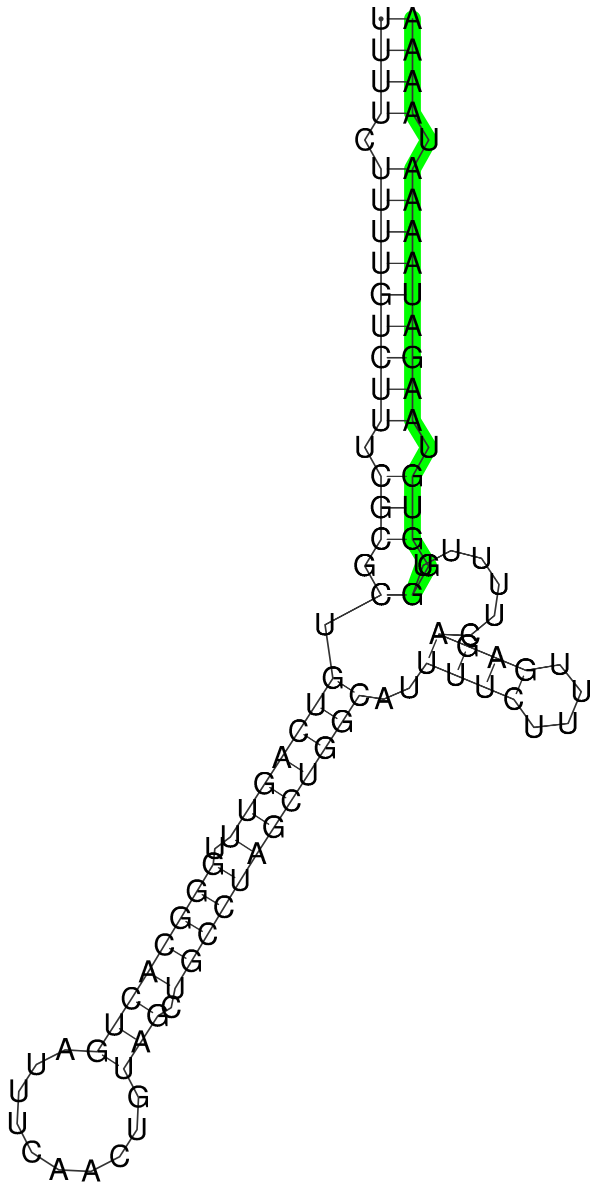

CCTGCATGGGCCATGATTCTGTTT_scaffold_1971_8937-8960

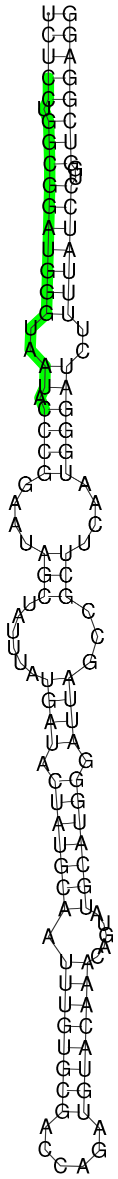

CCTGGCGGATGGGTAATAC_scaffold_14856_848-866

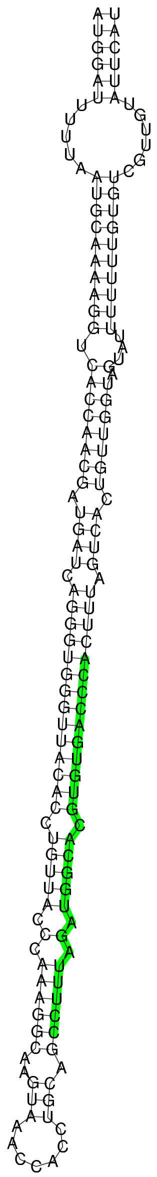

CCTTTAGATGGCACGTGTGACCCA_LG_XI_14319526-14319549

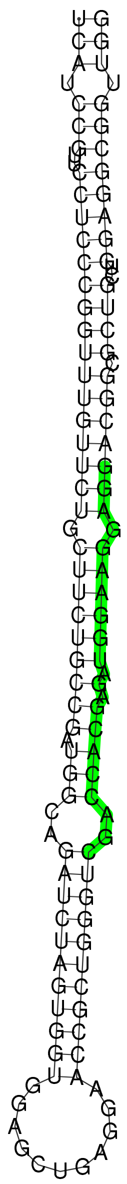

CGACCACGAGATGGAAGGAGG_LG_IV_1219678-1219698

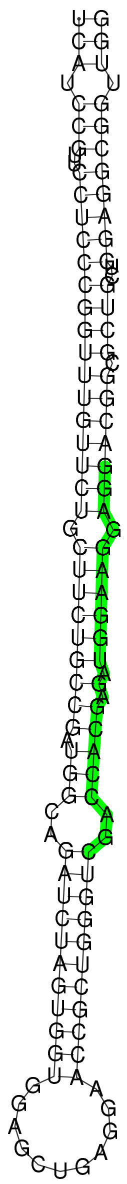

CGACCACGAGATGGAAGGAGG_LG_IV_980571-980591

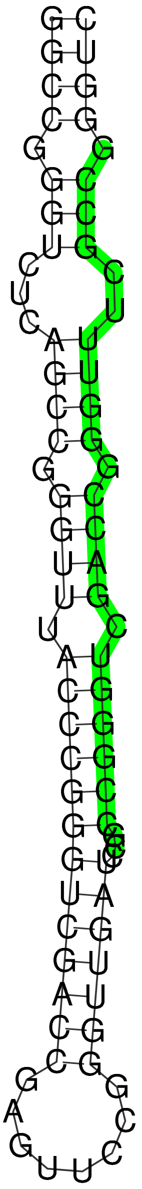

CGCCGGGTCGACCGGGTTTCGCCG_scaffold_1185_8233-8256

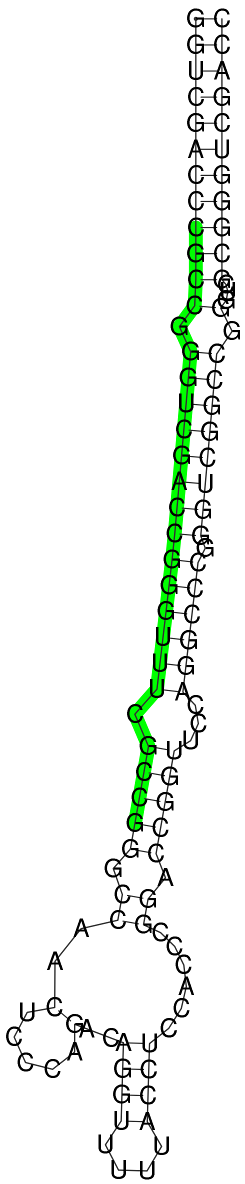

CGCCGGGTCGACCGGGTTTCGCCG_scaffold_8518_159-182

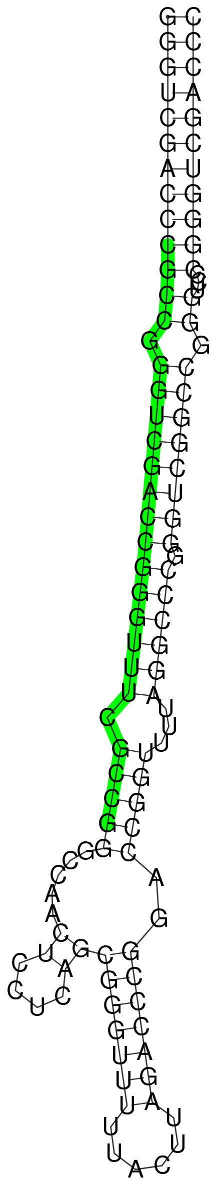

CGCCGGGTCGACCGGGTTTCGCCG_scaffold_97_32751-32774

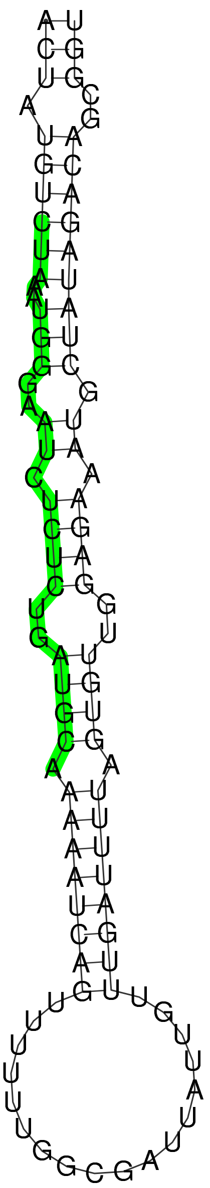

CTAAATGGGAATCTCTCTGATGCA_LG_XVIII_12025431-12025454

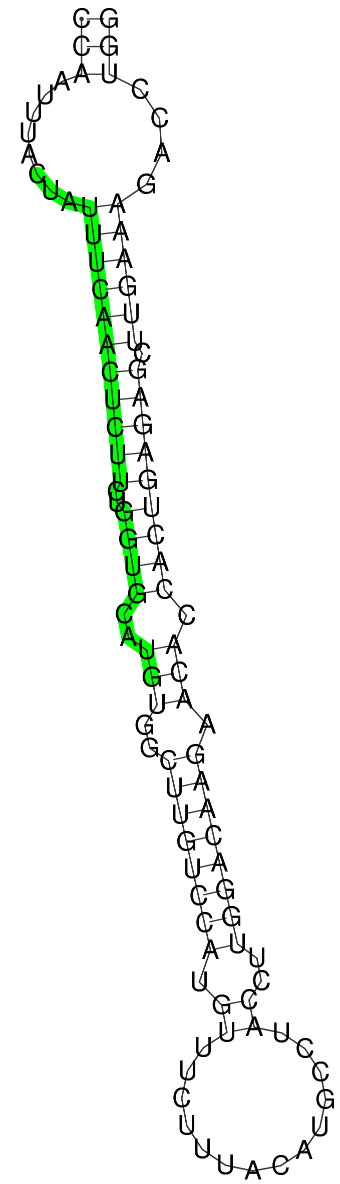

CTATTTCAACTCTTCTGGTGCATG_LG_XII_745801-745824

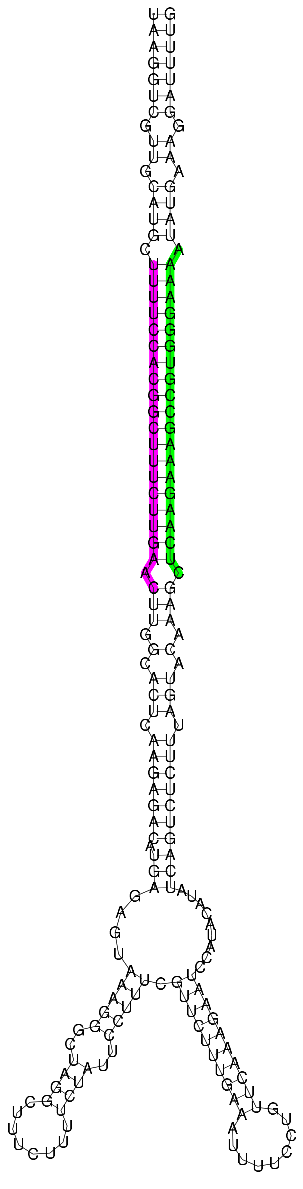

CTCAAGAAAGCCGTGGGAAAA_LG_VII_6490115-6490135

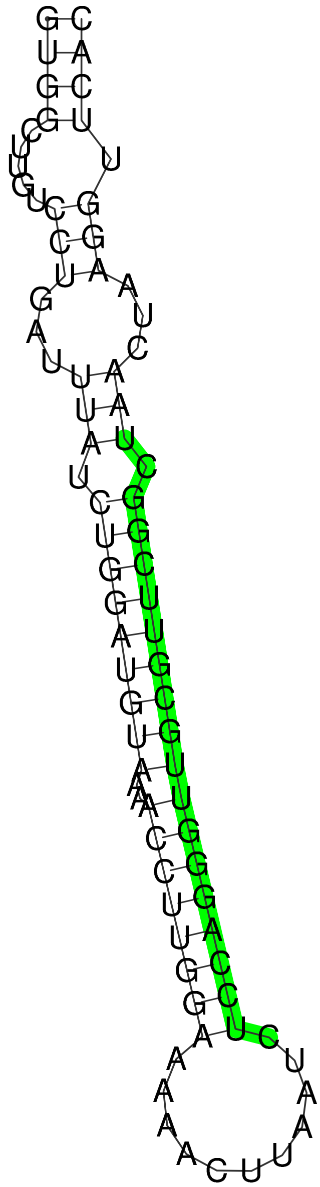

CTCCAGGGTTGCGTTCGGCT_LG_XV_3118235-3118254

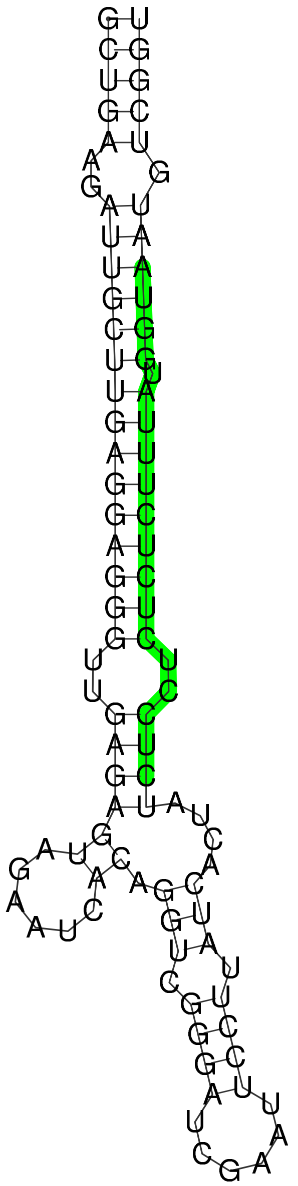

CTCCTCTCTCTTTATGGTA_scaffold_40_731022-731040

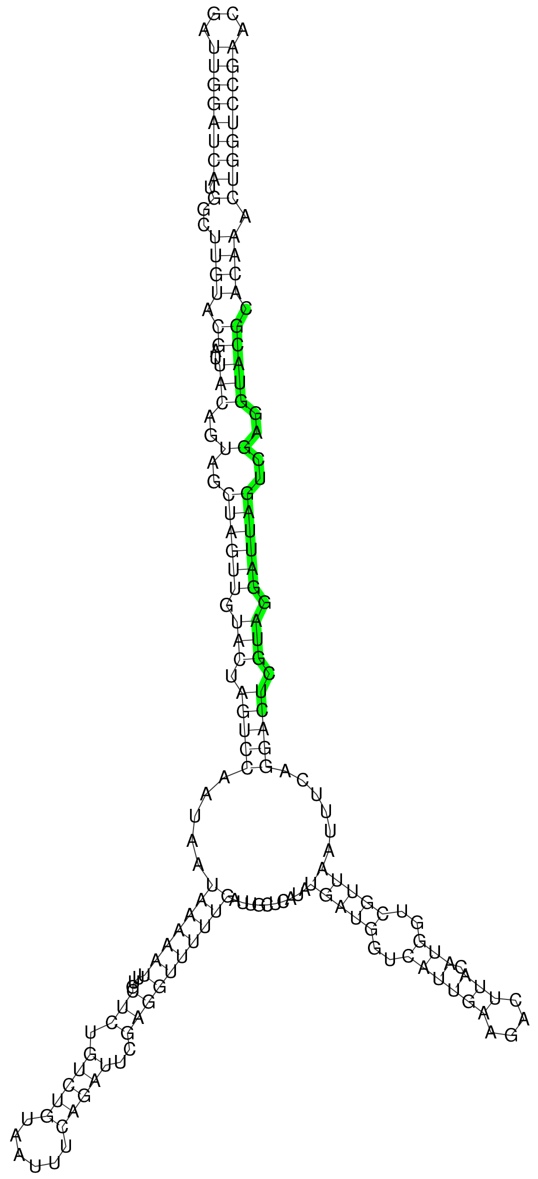

CTCGTAGGATTAGTCGAGGTACGC_scaffold_140_248347-248370

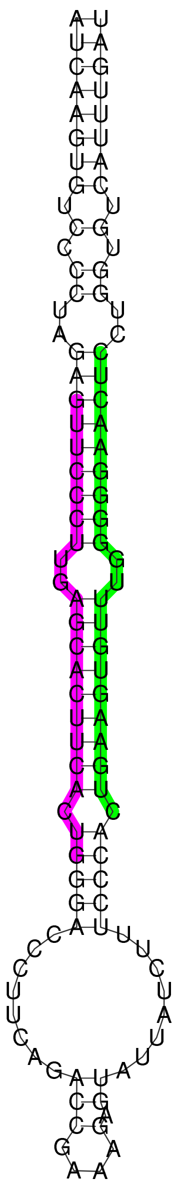

CTGAAGTGTTTGGGGGAACTC_LG_VI_6826412-6826432

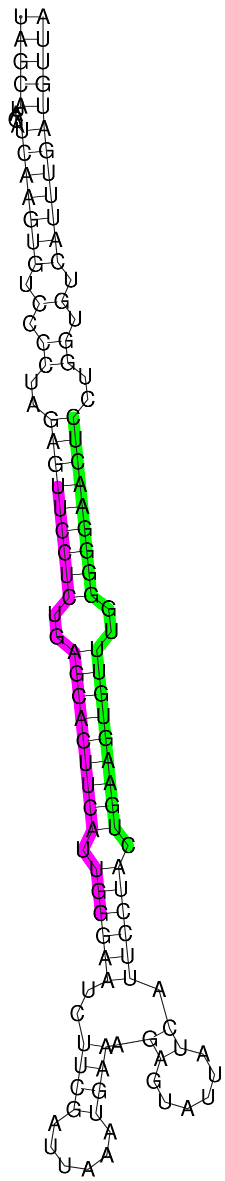

CTGAAGTGTTTGGGGGAACTC_LG_VI_7807450-7807470

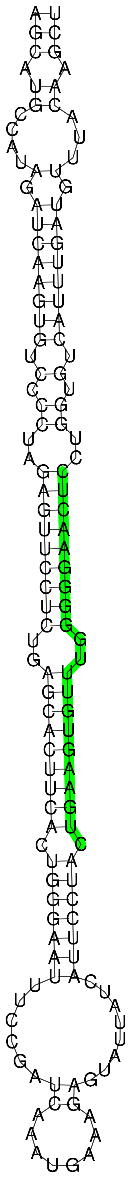

CTGAAGTGTTTGGGGGAACTC_LG_VI_7811571-7811591

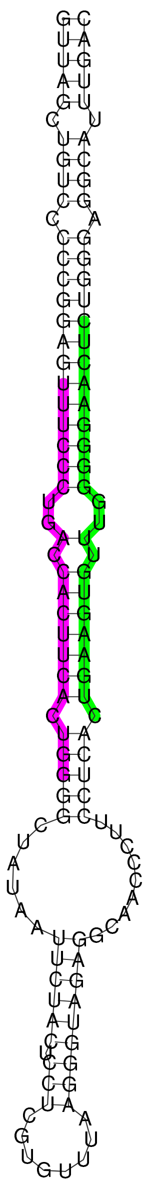

CTGAAGTGTTTGGGGGAACTC_LG_XVI_13302939-13302959

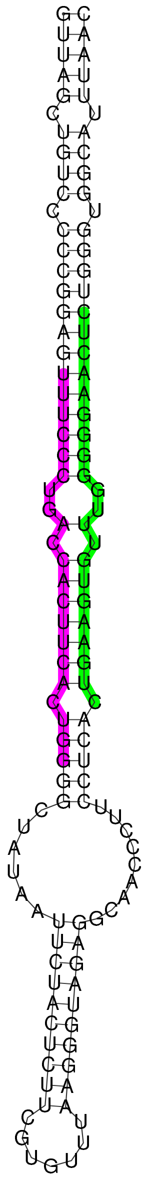

CTGAAGTGTTTGGGGGAACTC_LG_XVI_13308650-13308670

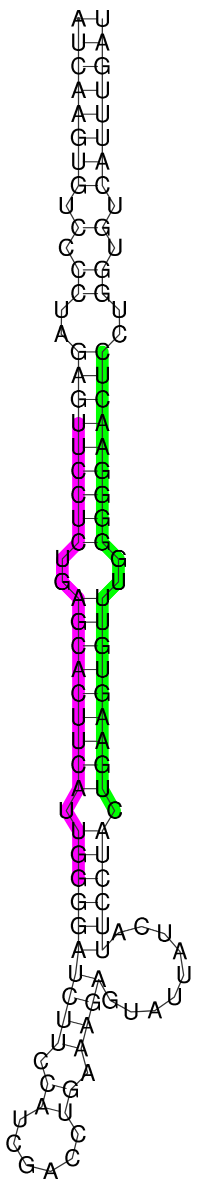

CTGAAGTGTTTGGGGGAACTC_LG_XVI_13309881-13309901

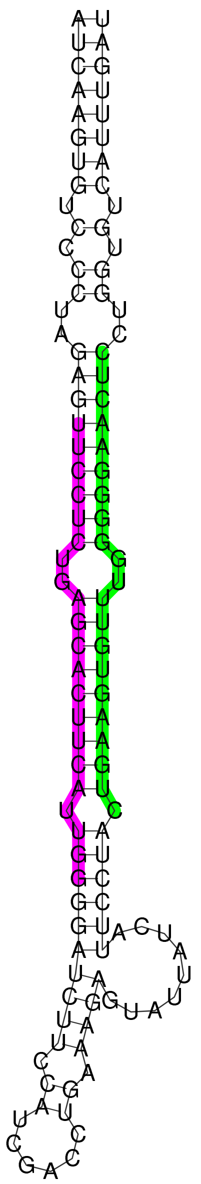

CTGAAGTGTTTGGGGGAACTC_LG_XVI_13313981-13314001

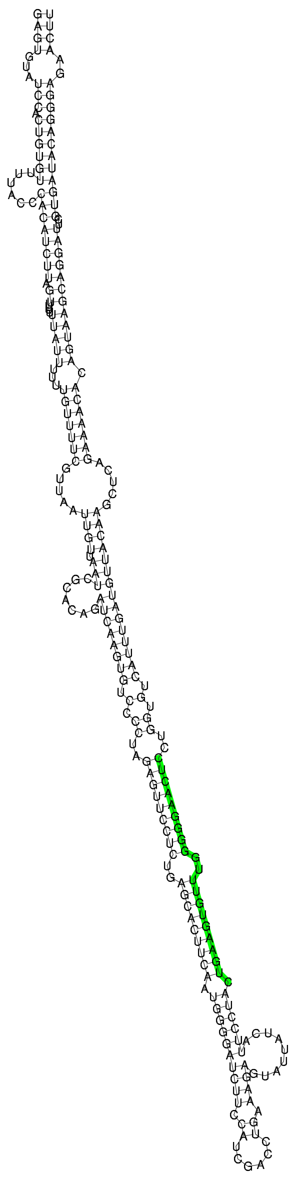

CTGAAGTGTTTGGGGGAACTC_LG_XVI_13316932-13316952

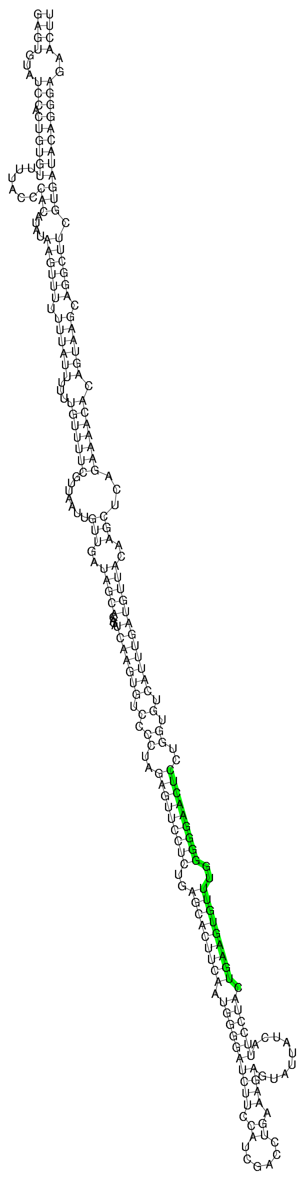

CTGAAGTGTTTGGGGGAACTC_LG_XVI_13325153-13325173

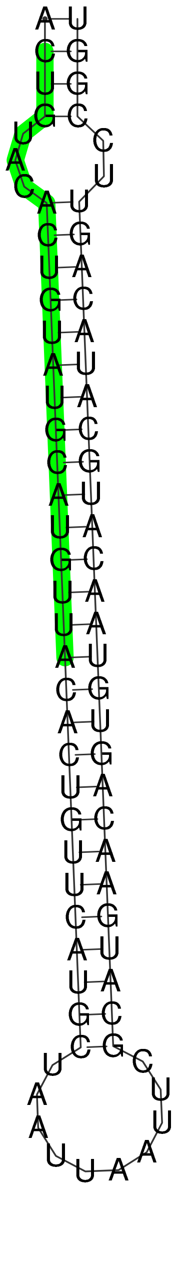

CTGTACACTGTATGCATGTTA_LG_II_16006717-16006737

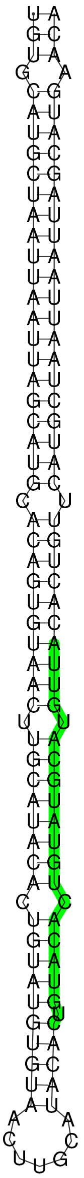

CTGTACACTGTATGCATGTTA_LG_IV_14490006-14490026

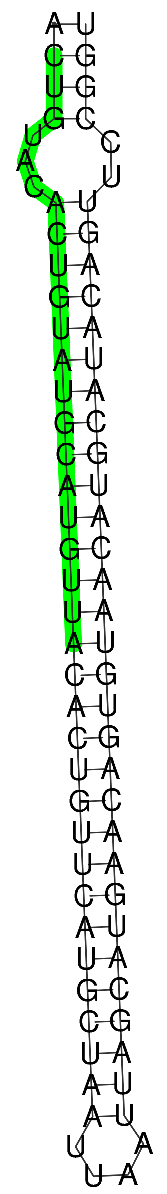

CTGTACACTGTATGCATGTTA_LG_I_19236113-19236133

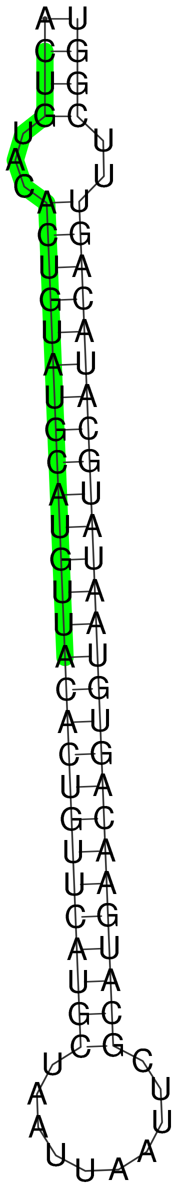

CTGTACACTGTATGCATGTTA_LG_I_24725995-24726015

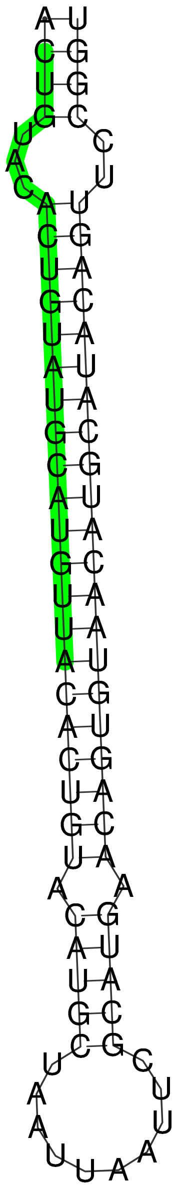

CTGTACACTGTATGCATGTTA_LG_VIII_2611317-2611337

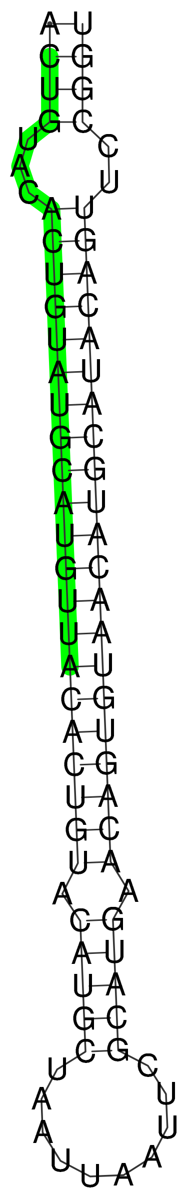

CTGTACACTGTATGCATGTTA_LG_VI_17678087-17678107

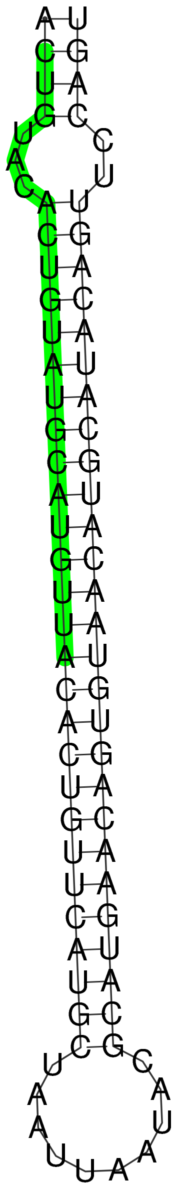

CTGTACACTGTATGCATGTTA_LG_XIV_6598445-6598465

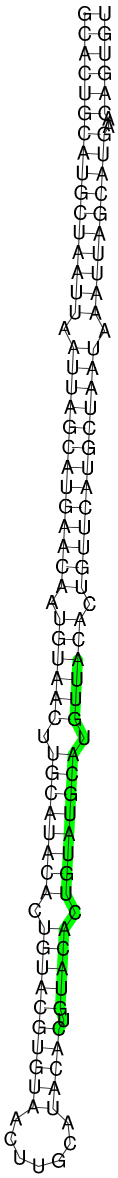

CTGTACACTGTATGCATGTTA_scaffold_15596_686-706

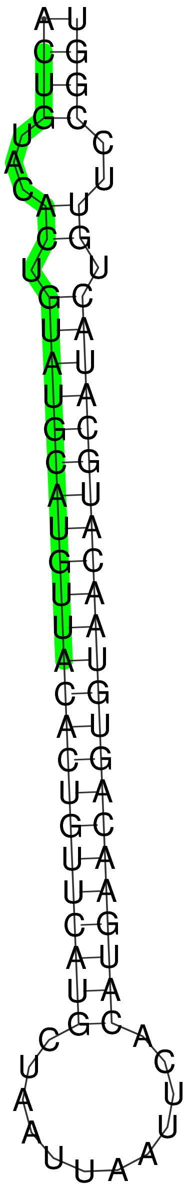

CTGTACACTGTATGCATGTTA_scaffold_15705_1012-1032

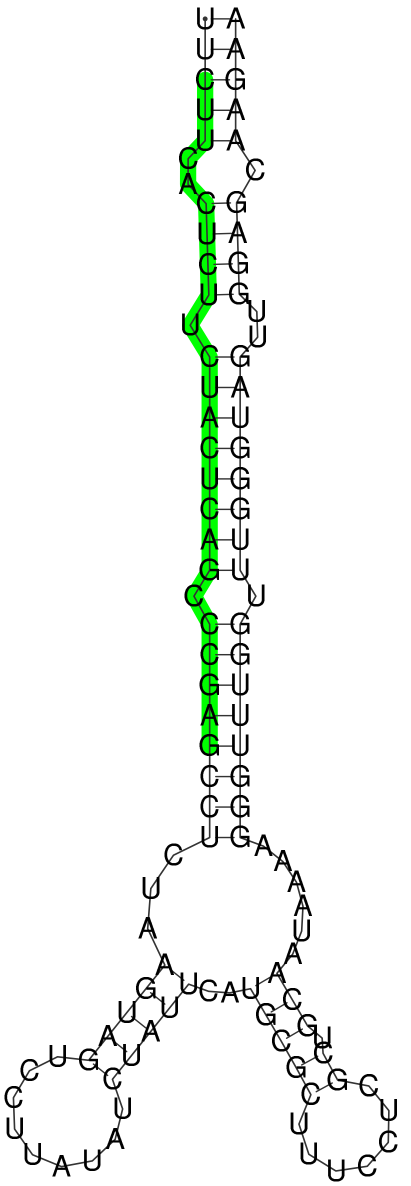

CTTCACTCTTCTACTCAGCCCGAG_scaffold_2619_2026-2049

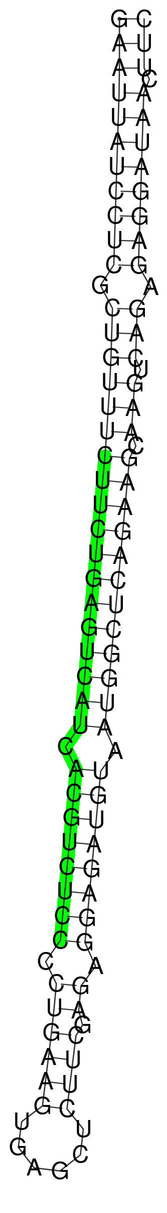

CTTCTGAGTCATCACGTCTCC_LG_XI_11528341-11528361

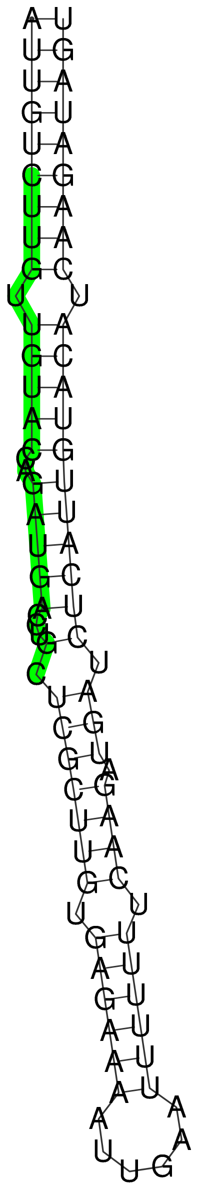

CTTGTTGTACCAGATGACCTGC_LG_IV_3019757-3019778

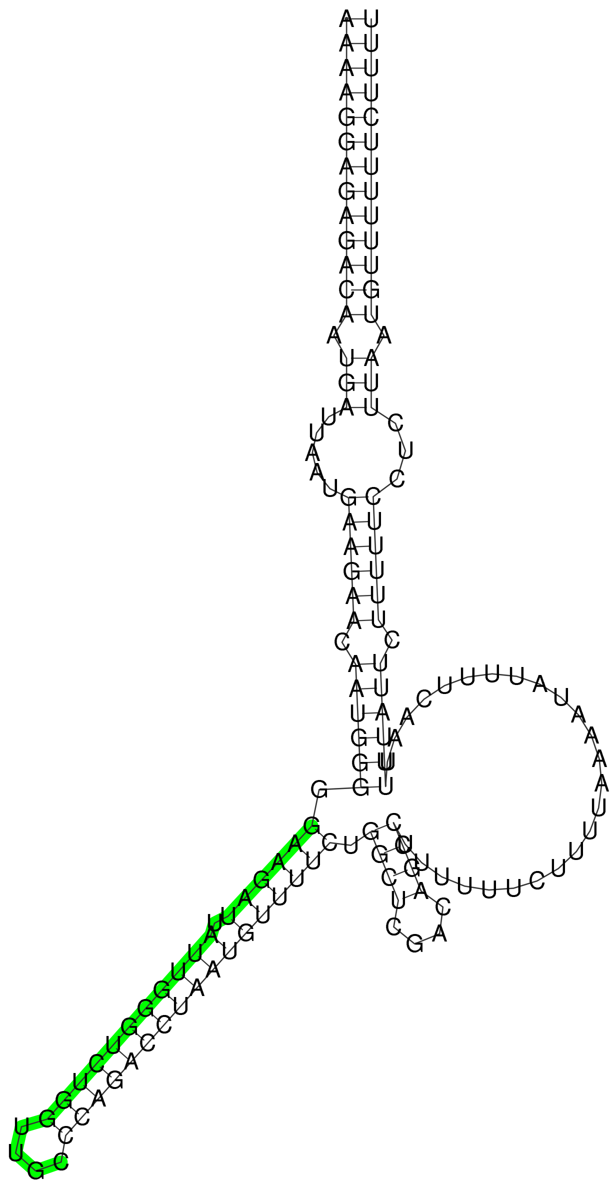

GAAGATTATTGGGTCTGGTTGC_scaffold_146_334776-334797

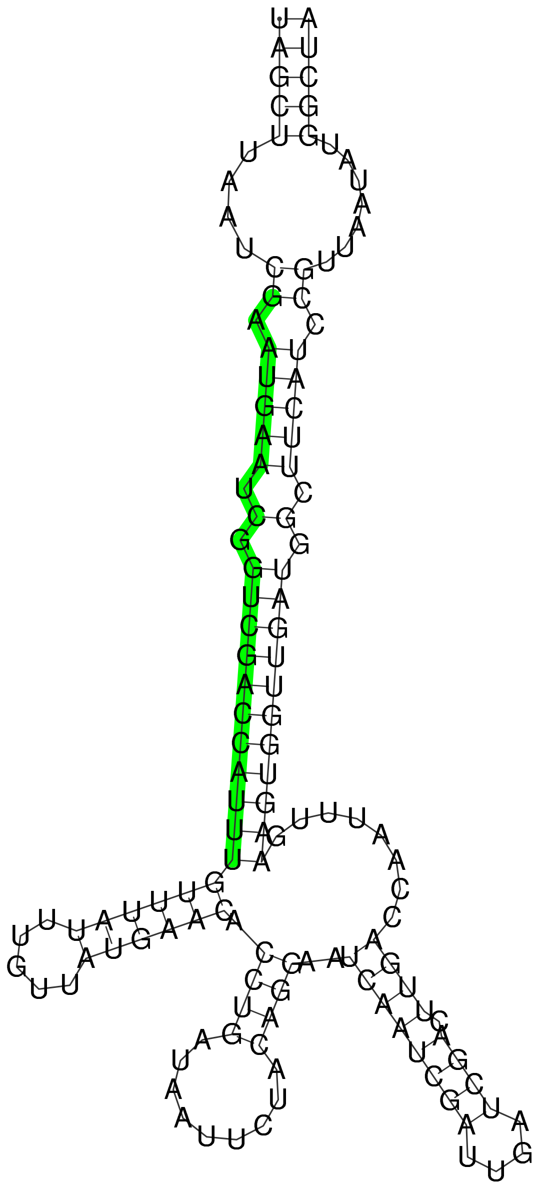

GAATGAATCGGTCGACCATTT_LG_XIII_7671991-7672011

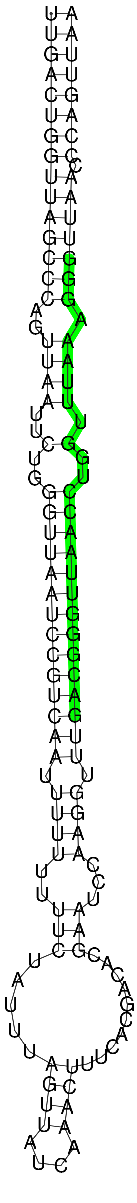

GACGGGTTAACCTGGTTTAAAGGG_LG_XII_5309652-5309675

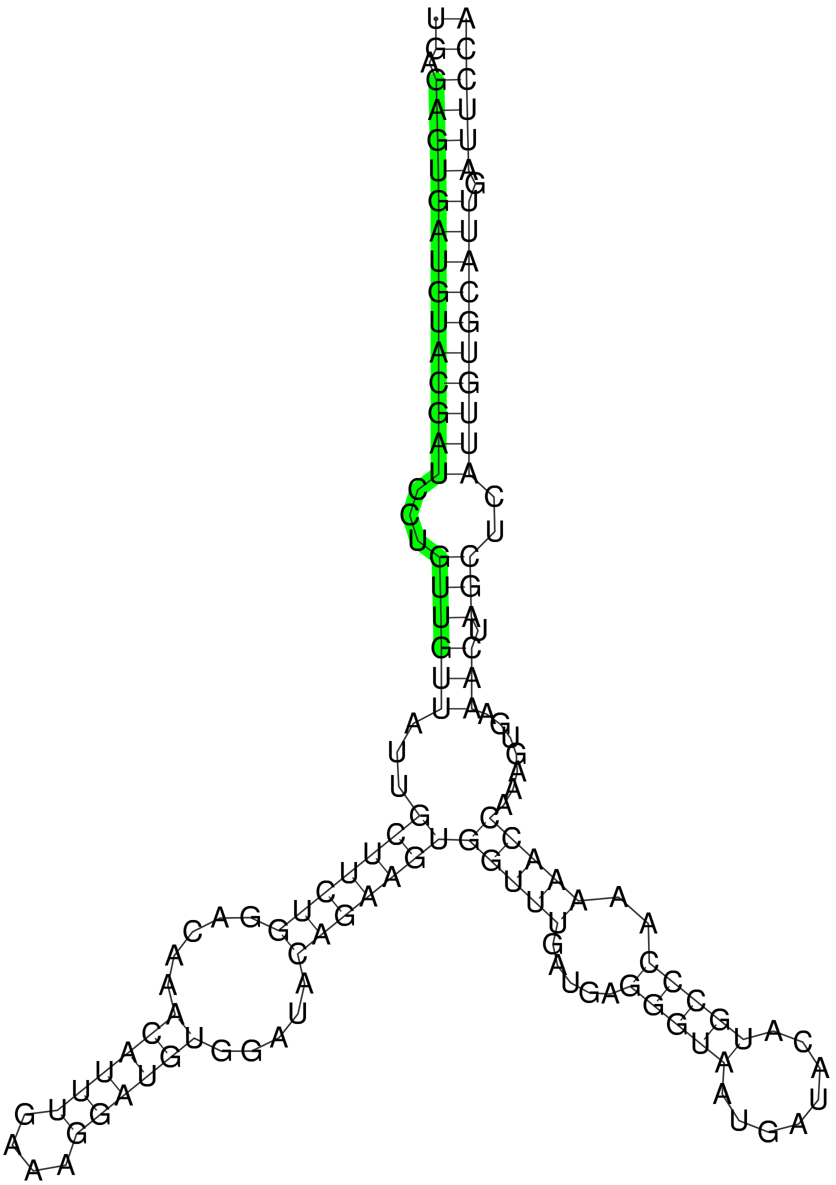

GAGTGATGTACGATCCTGTTG_scaffold_57_1440263-1440283

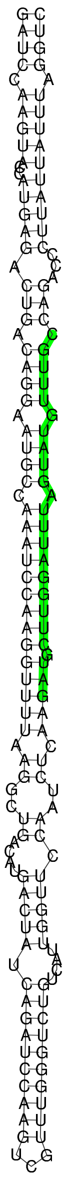

GATGCTTGGATTTAGTATGTTTGC_LG_XII_7774805-7774828

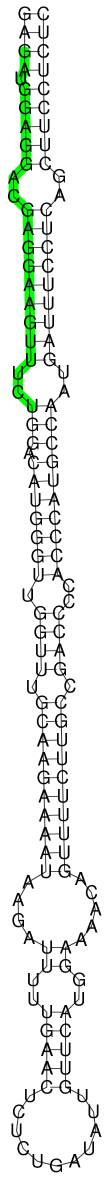

GATGGAGGACGAGGAAGTTTCT_LG_X_11953956-11953977

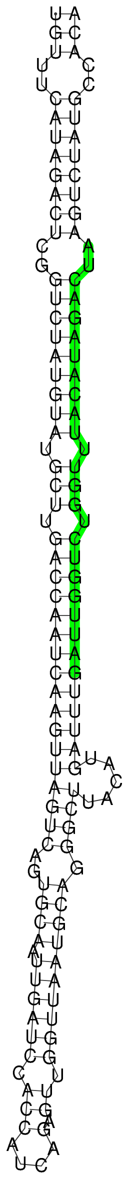

GATTGGTCTGGTTTACATAGACTA_LG_X_14475326-14475349

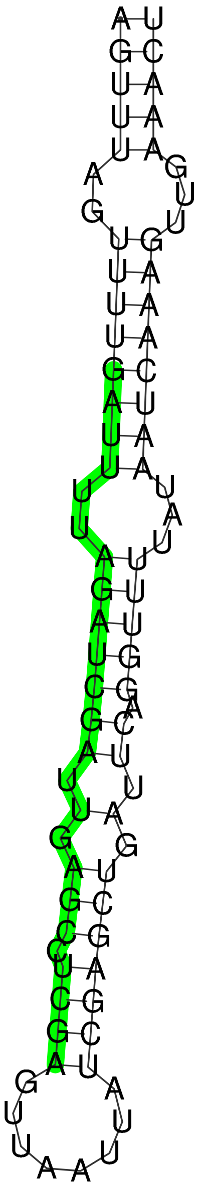

GATTTTAGATCGATTGAGCCTCGA_LG_XI_14585756-14585779

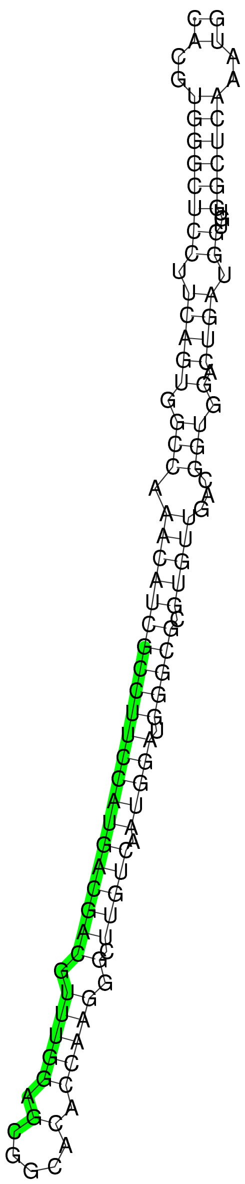

GCCTTCCATGACGACGTTTGGAGC_scaffold_66_1713857-1713880

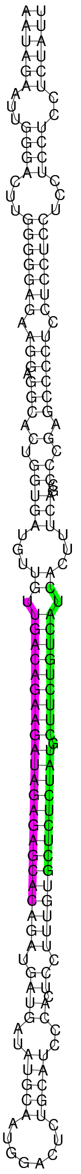

GCTCTCTATGCTTCTGTCATC_LG_XV_3242459-3242479

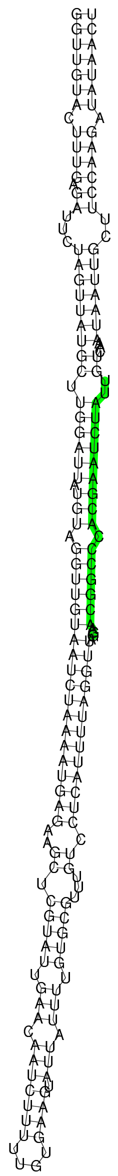

GGAACGGCCCACGAATCTATT_LG_VIII_15331904-15331924

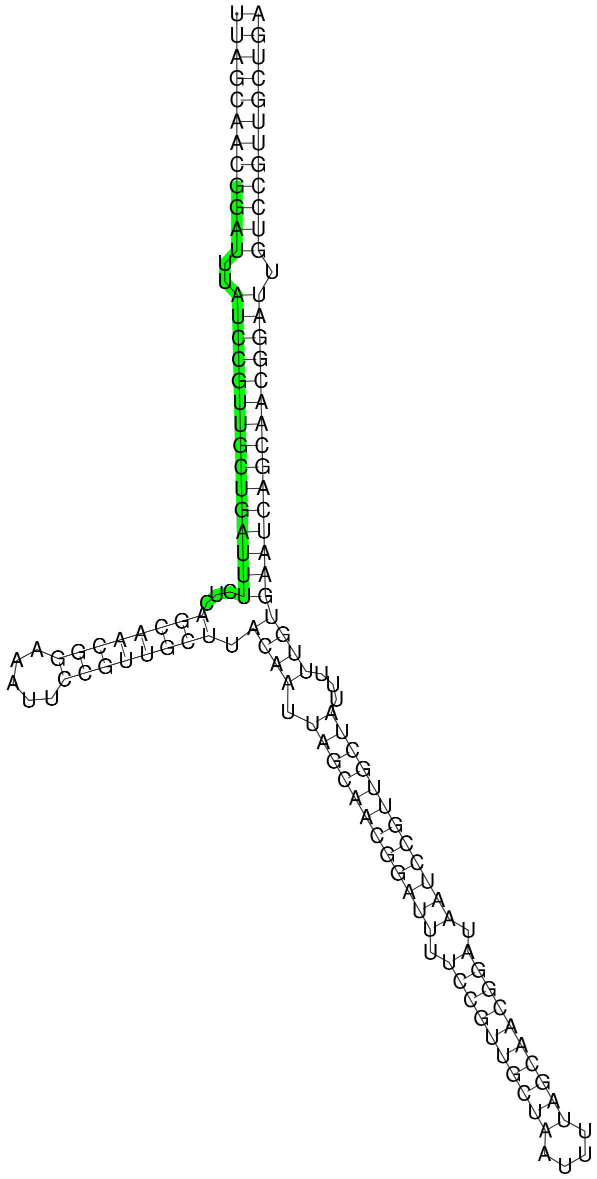

GGATTTATCCGTTGCTGATTTCTC_LG_III_9591403-9591426

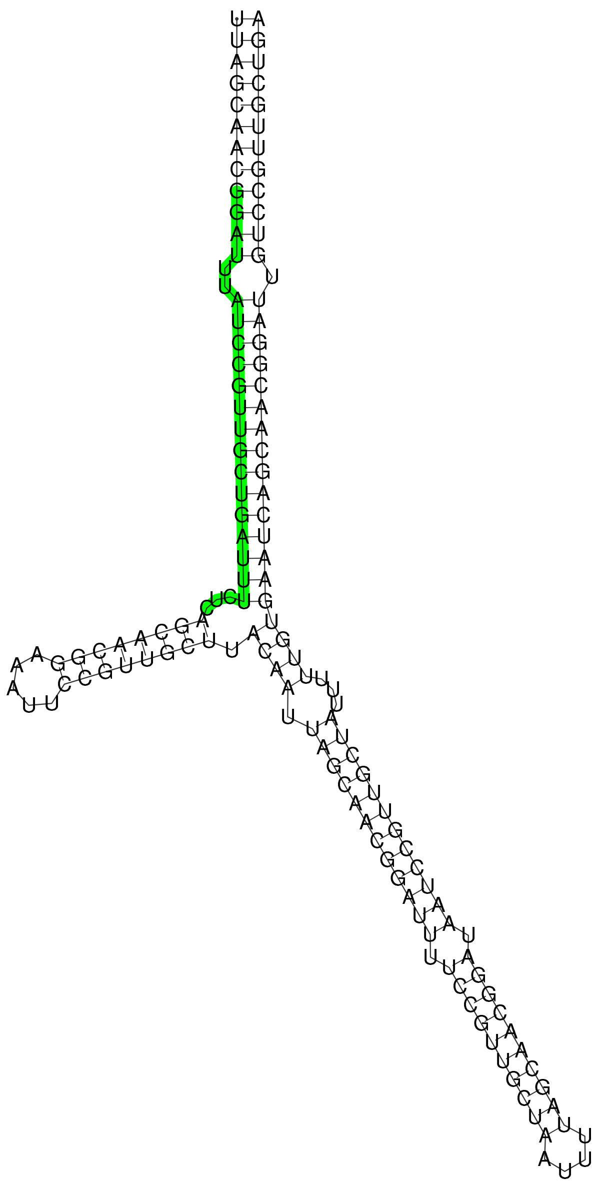

GGATTTATCCGTTGCTGATTTCTC_scaffold_1249_7673-7696

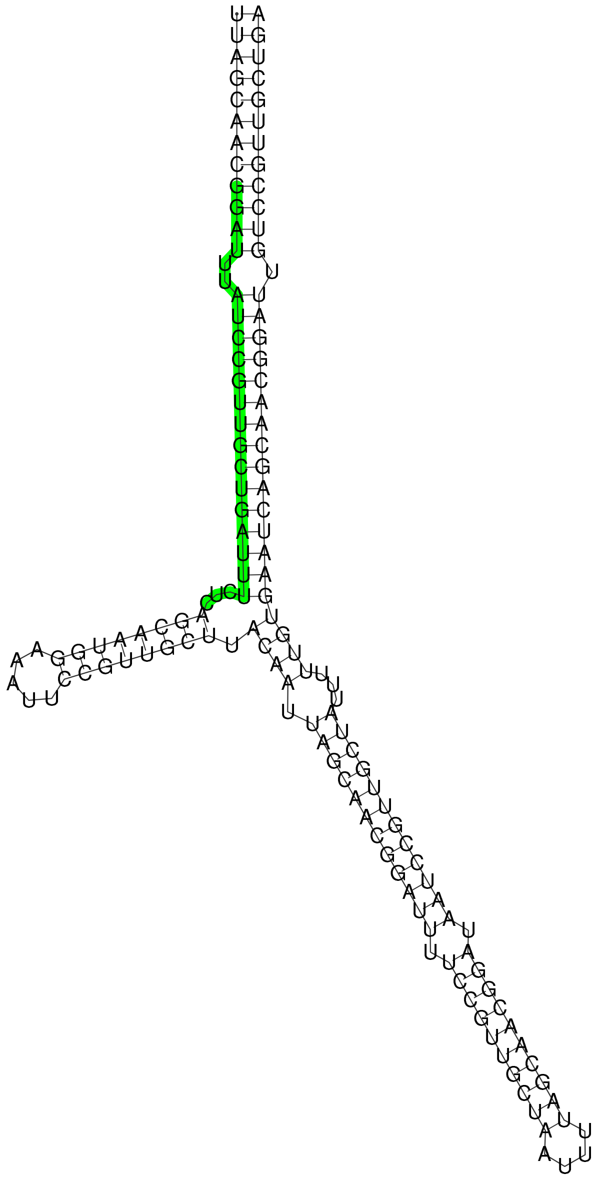

GGATTTATCCGTTGCTGATTTCTC_scaffold_3871_403-426

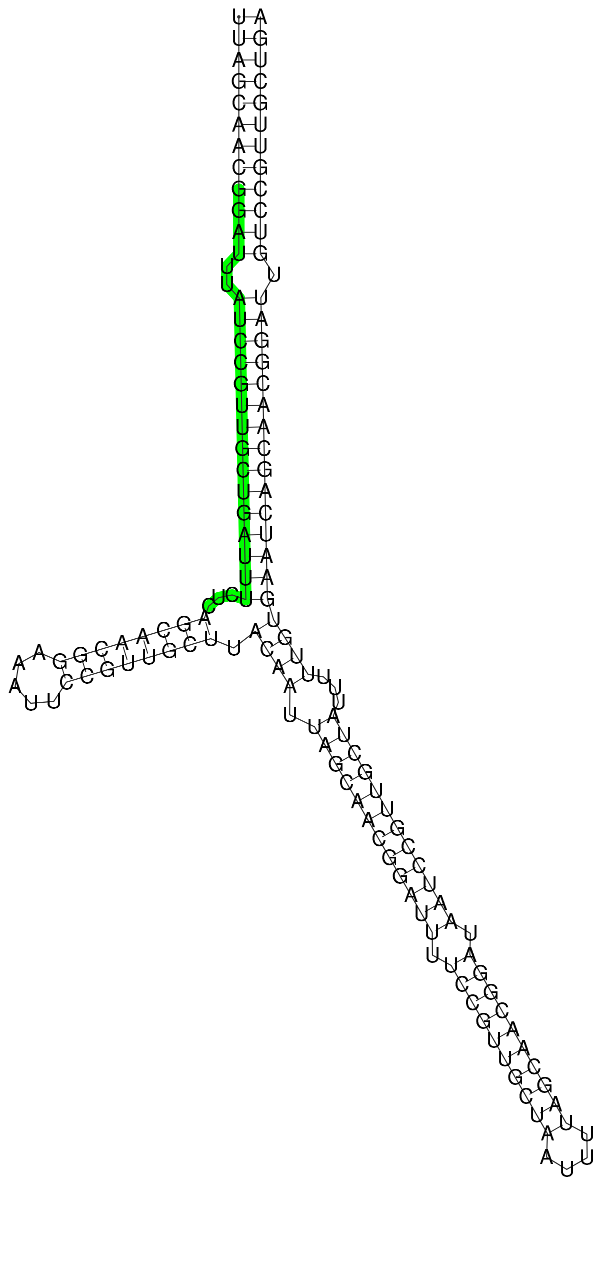

GGATTTATCCGTTGCTGATTTCTC_scaffold_40_1876856-1876879

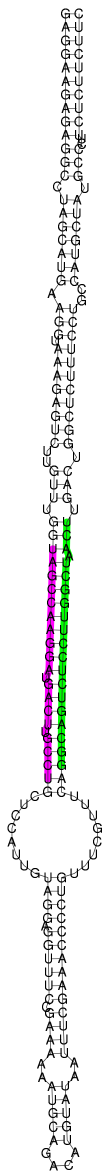

GGCAGTCTCCTTGGCTAACT_LG_I_24674628-24674647

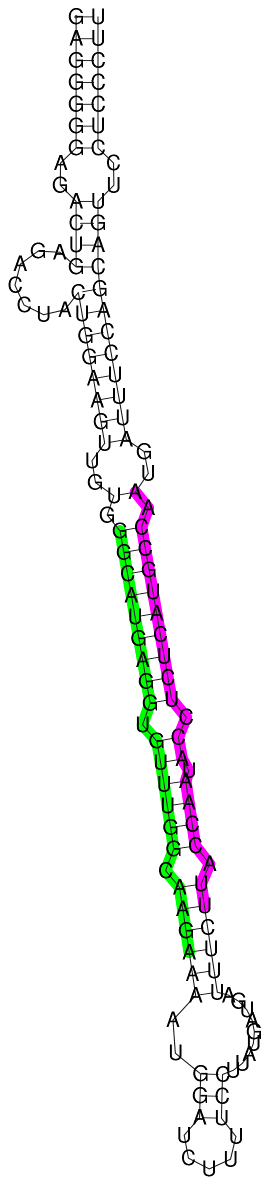

GGCATGAGGTGTTTGGCAAGA_LG_II_21846112-21846132

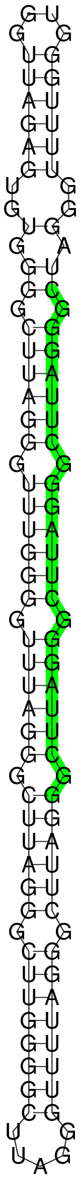

GGCTTAGGGCTTAGGGCTTAGGGC_scaffold_12686_286-309

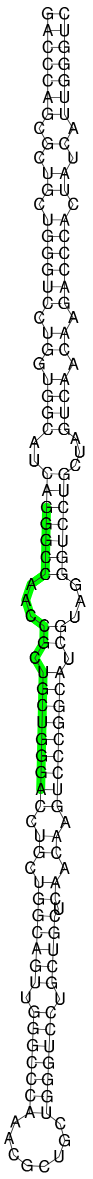

GGGCCAACCGCTGCTGGGA_LG_I_22795515-22795533

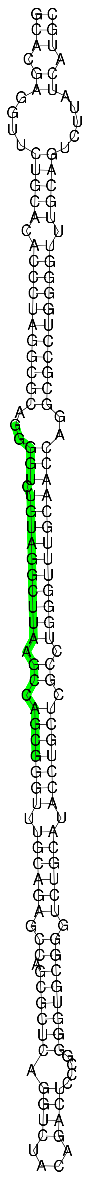

GGGGTCTGTAGGCTTAAGCCAGCG_scaffold_1489_10911-10934

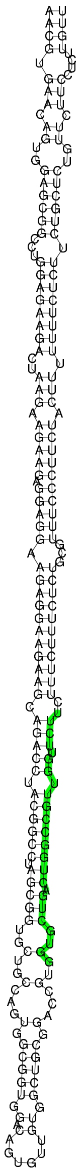

GGTGCTGACTGGCCGTTGGTTCTT_scaffold_44_33455-33478

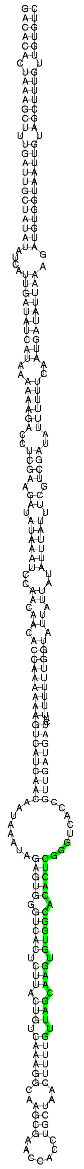

GTTAGCAAGTGTGGCACACTCGGG_LG_X_14731072-14731095

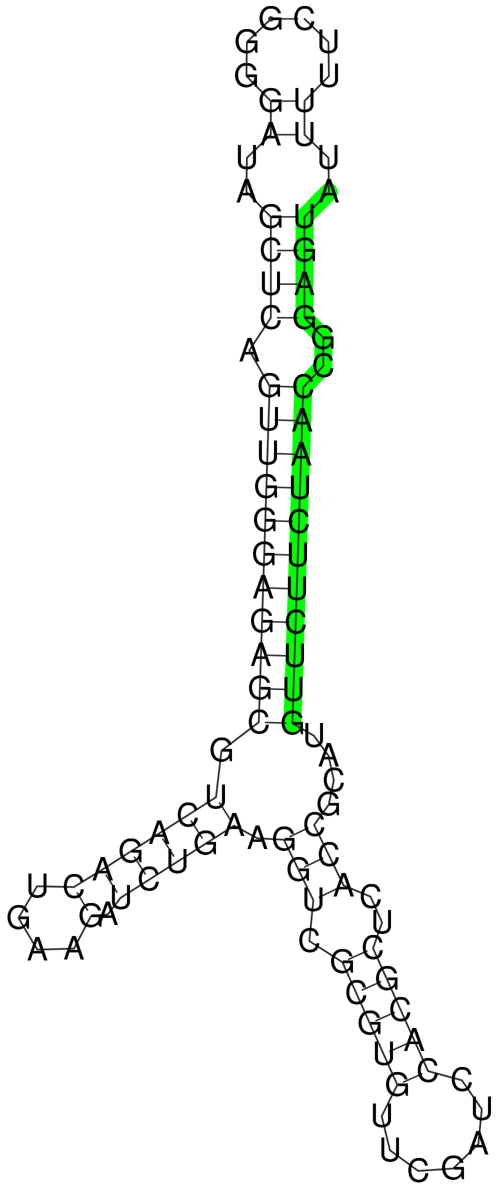

GTTCTTCTAACCGGAGTA_LG_XIV_4869488-4869505

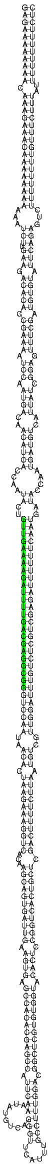

GTTGAAAAGATTTTGACGAGGCGG_LG_V_14074159-14074182

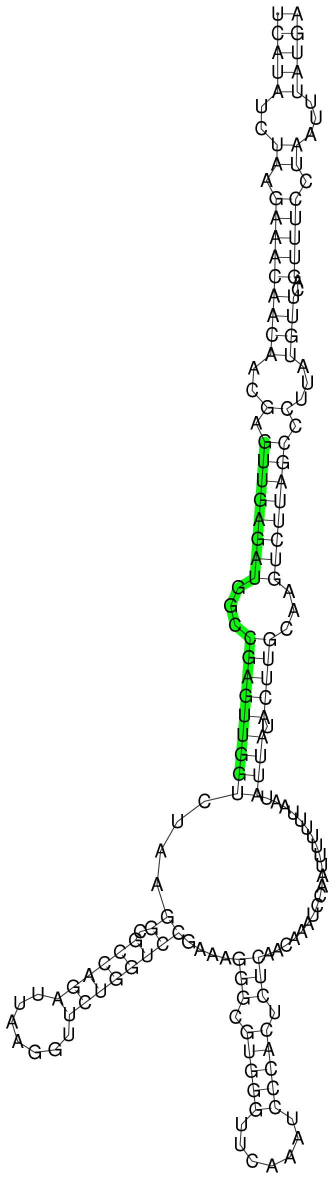

GTTGAGATGGCCGAGTTGG_LG_VII_5068707-5068725

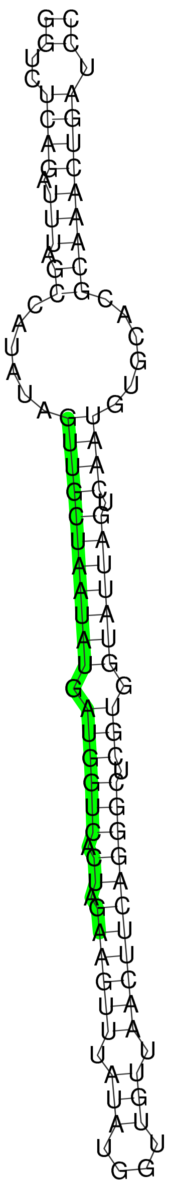

GTTGCTAATATGATGGTCACTAGA_LG_XVIII_4688632-4688655

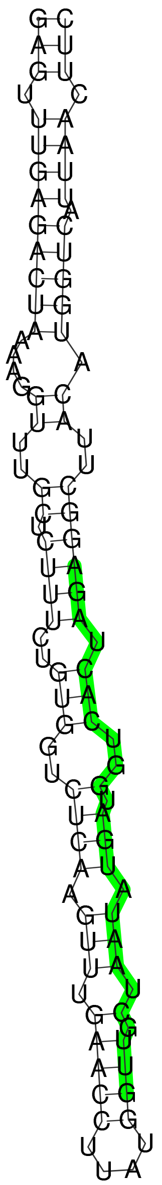

GTTGCTAATATGATGGTCACTAGA_LG_X_15473869-15473892

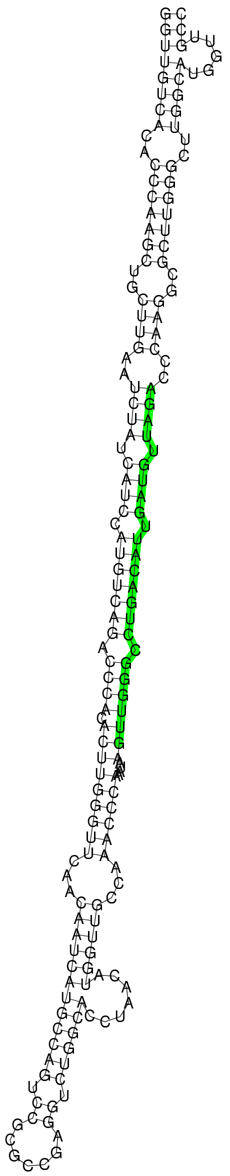

GTTGGGCCTGACATTGATGTTAGA_LG_XIX_4635506-4635529

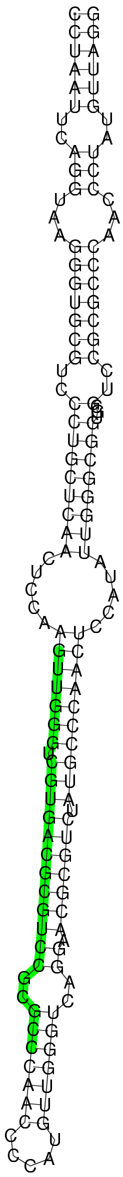

GTTGGGTCGTGACGCGTCCGCGCC_scaffold_28_1810949-1810972

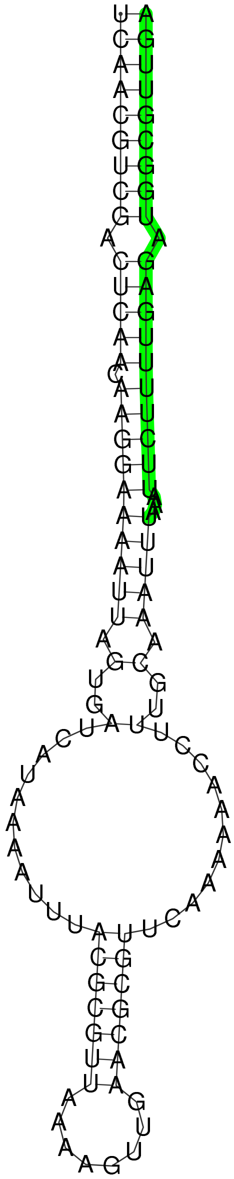

TAAATTCTTTTGAGATGGCGTTGA_LG_I_35517576-35517599

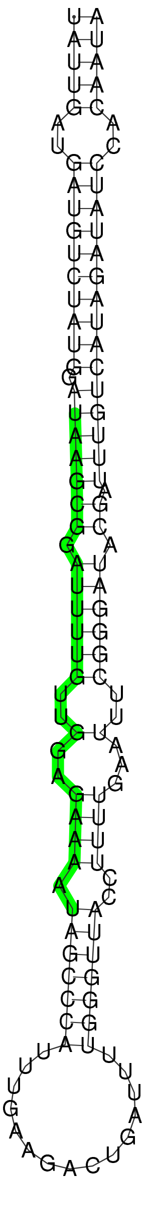

TAAGCGGATTTTGTTGGAGAAAAT_LG_II_12977375-12977398

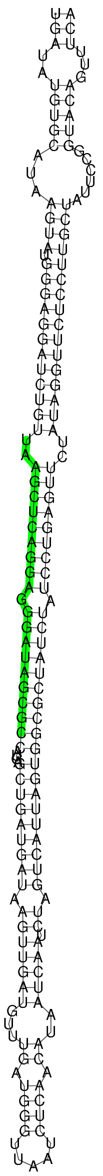

TAAGCTCAGGAGGGATAGCGC_LG_IX_5407909-5407929

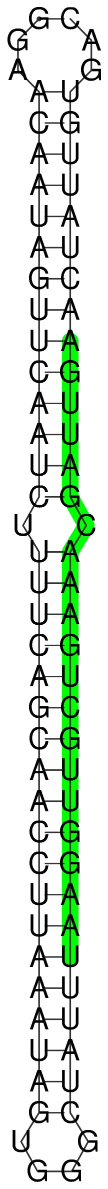

TAAGGTTGCTGAAACGATTGA_LG_IX_2107491-2107511

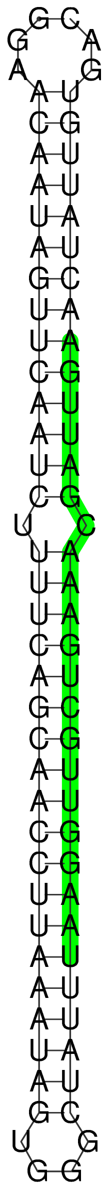

TAAGGTTGCTGAAACGATTGA_scaffold_5043_2074-2094

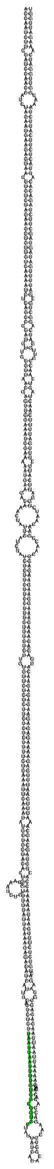

TAATTAATTTTGTGGCGGTCC_LG_XIX_2578002-2578022

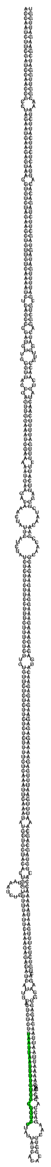

TAATTAATTTTGTGGCGGTCC_LG_XIX_2587887-2587907

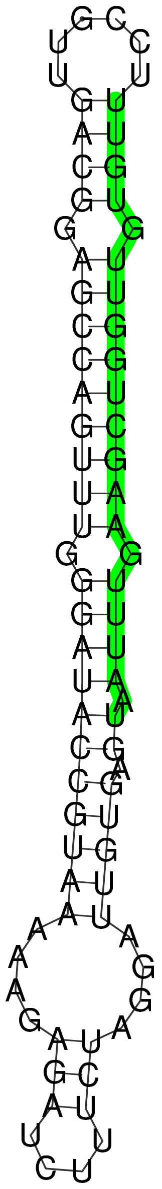

TAATTTGAAGCTGGTTGTGTT_LG_XVIII_9631042-9631062

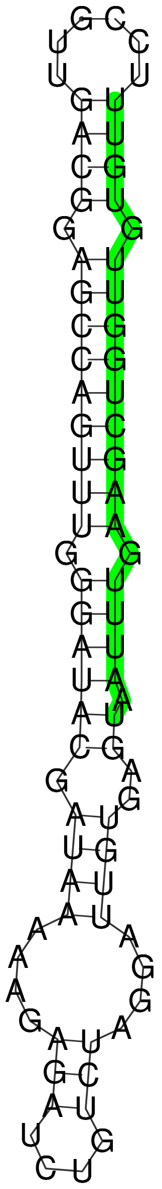

TAATTTGAAGCTGGTTGTGTT_scaffold_121_140019-140039

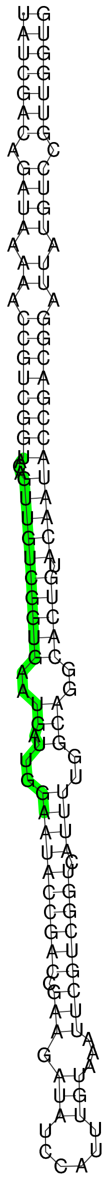

TACAGTTGTCGGTGAATGATTGGA_scaffold_871_17150-17173

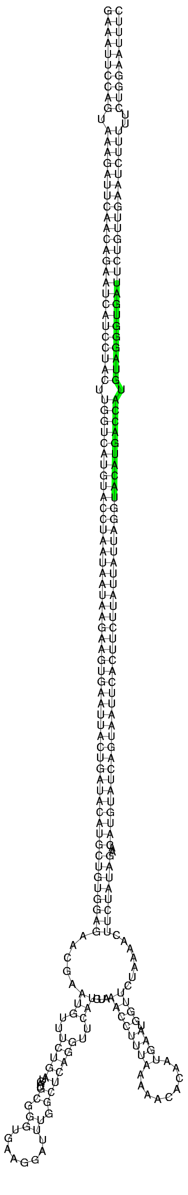

TACATGACCATGTAGGGTGAT_LG_I_21735212-21735232

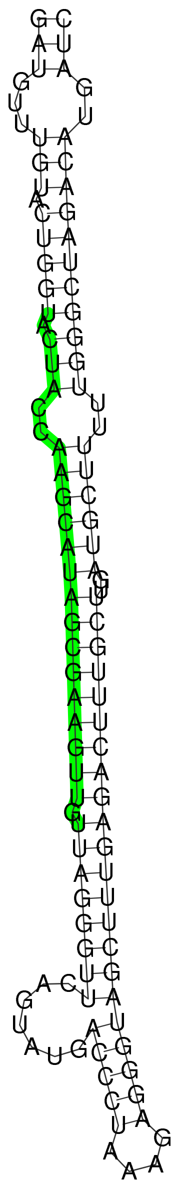

TACTACCAAGCATAGCGAAGTTGT_scaffold_232_134309-134332

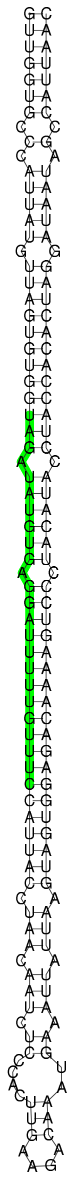

TAGATATGTGAGGATTTTTGTTTC_scaffold_125_453133-453156

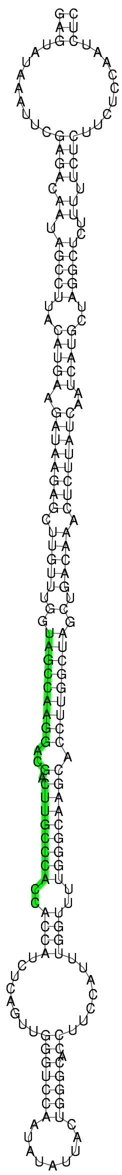

TAGCCAAGGACGACTTGCCCACC_LG_V_2516871-2516893

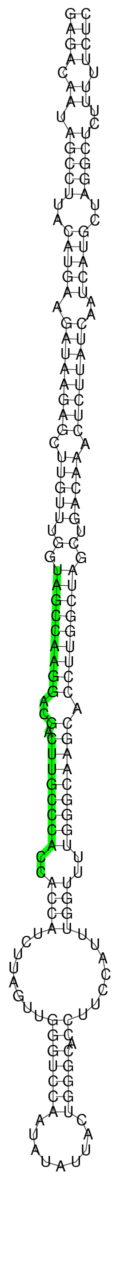

TAGCCAAGGACGACTTGCCCACC_LG_X_6859507-6859529

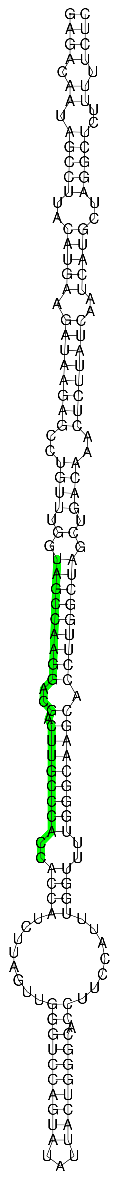

TAGCCAAGGACGACTTGCCCACC_LG_X_6867134-6867156

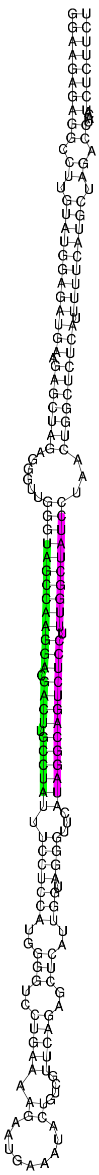

TAGCCAAGGACGACTTGCCTA_LG_XV_7670943-7670963

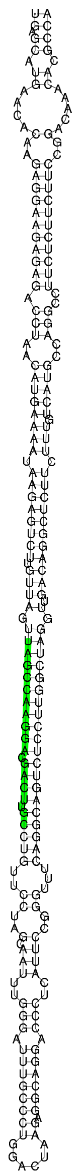

TAGCCAAGGACGACTTGC_LG_I_20793882-20793899

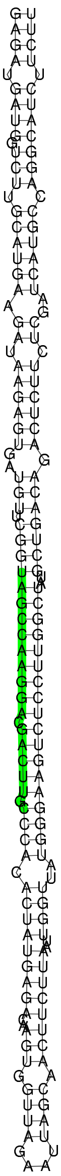

TAGCCAAGGACGACTTGC_LG_VII_6117604-6117621

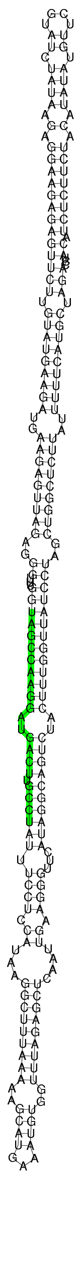

TAGCCAAGGATGACTTGCCT_LG_XV_7674111-7674130

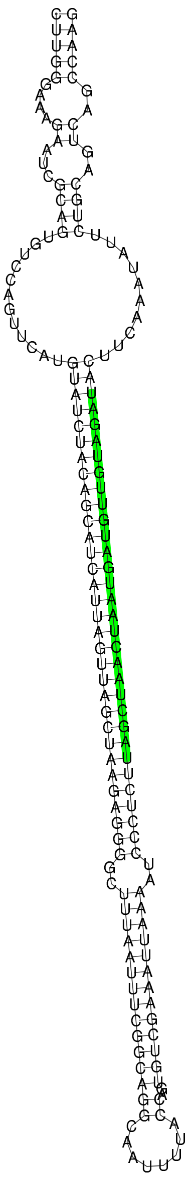

TAGCTAACTAATGATGTTGTAGAT_LG_I_23186188-23186211

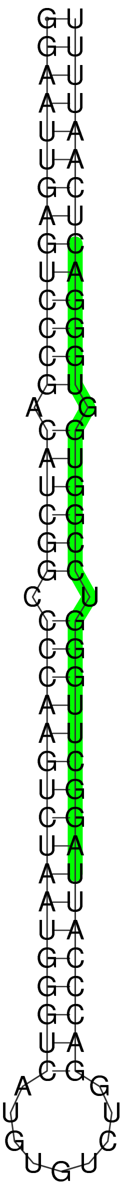

TAGGCTTGGGTCCGGTGGTGGGAC_LG_I_2559441-2559464

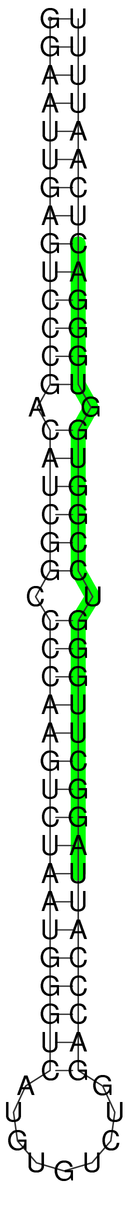

TAGGCTTGGGTCCGGTGGTGGGAC_scaffold_222_256193-256216

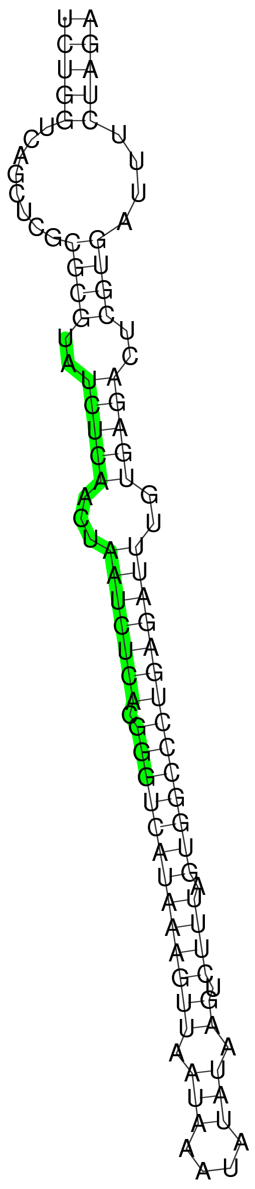

TATCTCAACTAATCTCACGGG_LG_XVIII_3766357-3766377

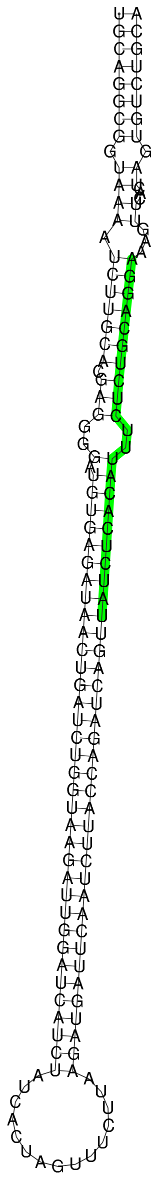

TATCTCACATTTCTCTGCAGGA_scaffold_6319_2152-2173

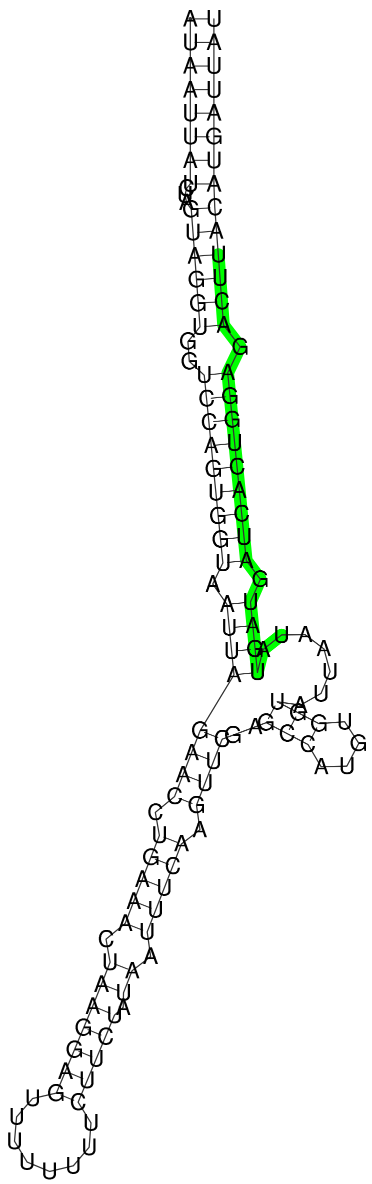

TATGATGATCACTGGAGACTT_LG_XV_7526016-7526036

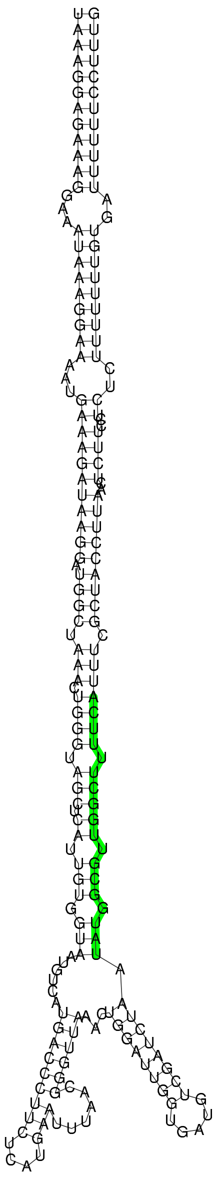

TATGGCGTTGGCTTTTCA_LG_VI_6335406-6335423

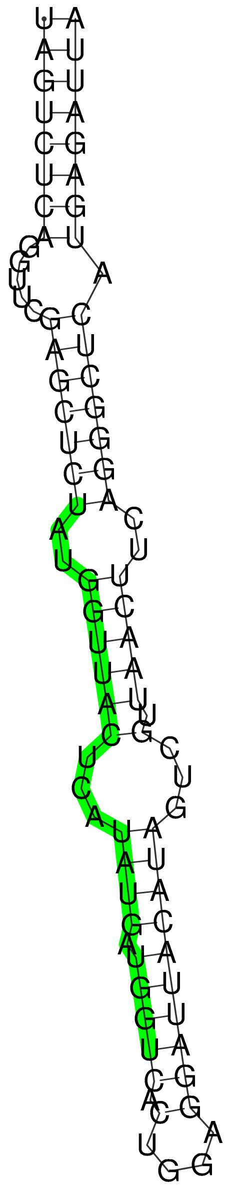

TATGGTTACTCATATGATGGT_scaffold_152_345188-345208

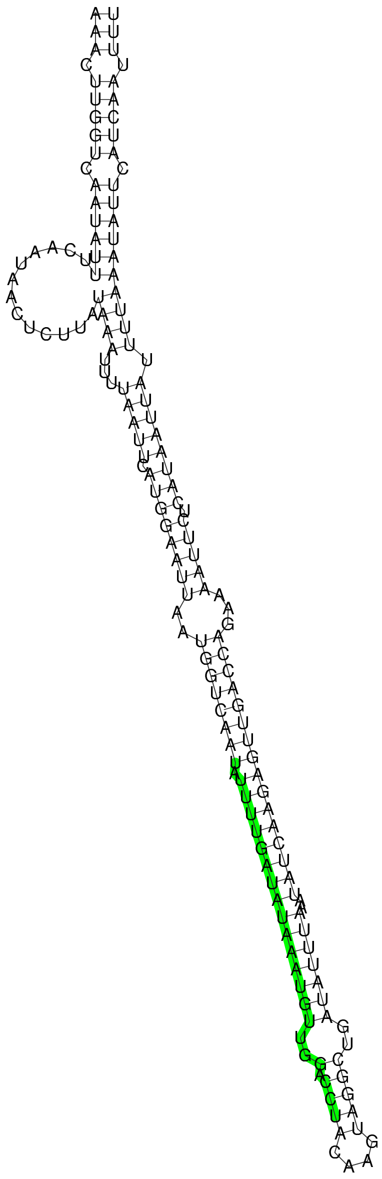

TATTTTGATATAAATGTTGGACCT_LG_XIII_5277141-5277164

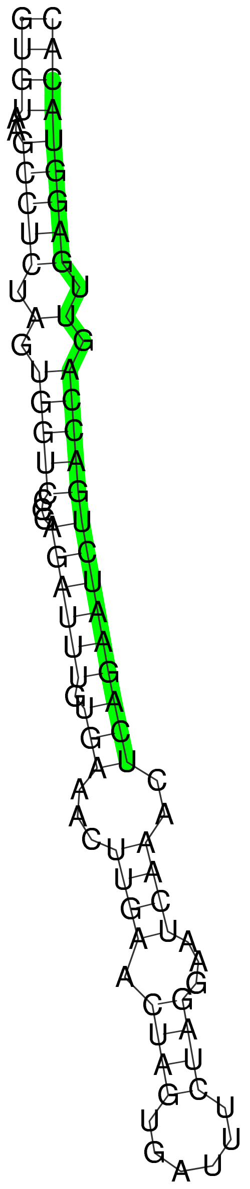

TCAGAATCTGACCAGTTGAGGTAC_LG_X_5391093-5391116

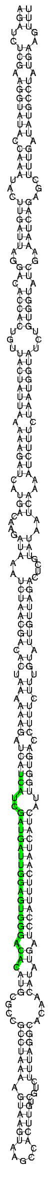

TCATCGATGATTGGAGTGGGACAC_LG_XII_8932089-8932112

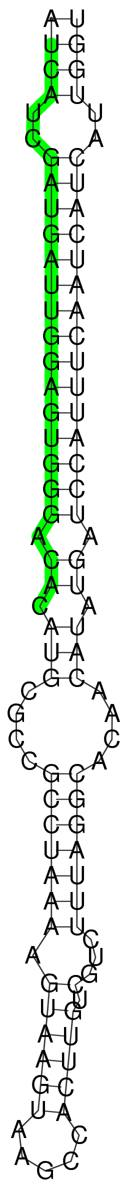

TCATCGATGATTGGAGTGGGACAC_scaffold_1705_10035-10058

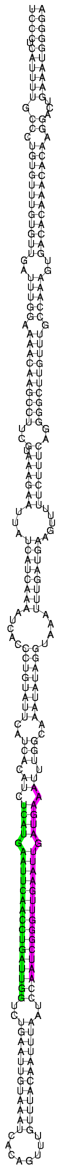

TCATGAATTCAACCTGATTGG_LG_II_14180794-14180814

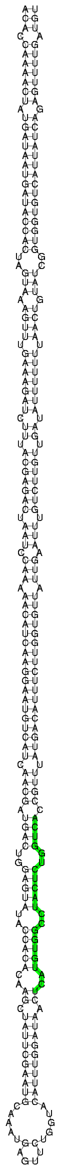

TCATGTGGCCTACTCTGGTCA_LG_X_15540217-15540237

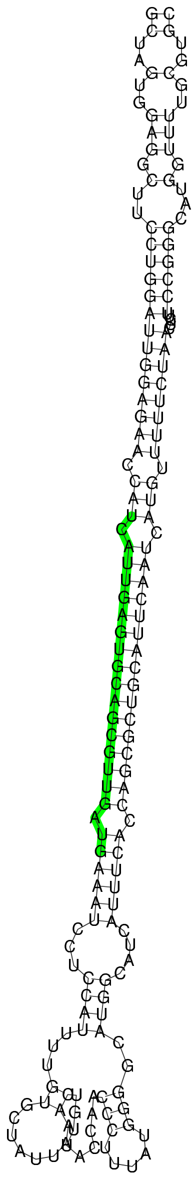

TCATTGAGTGCAGCGTTGATG_LG_XI_3295608-3295628

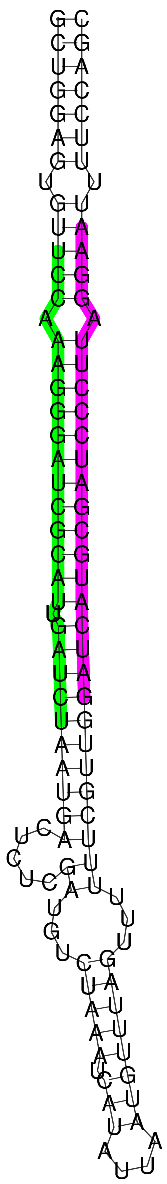

TCCAAAGGGATCGCATTGATCT_LG_XV_10351684-10351705

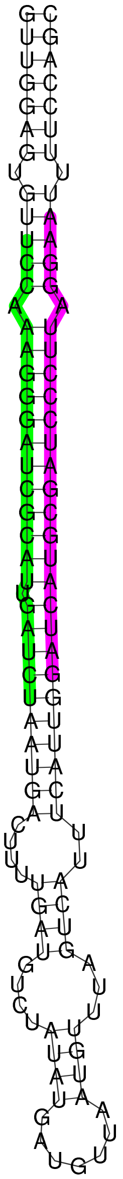

TCCAAAGGGATCGCATTGATCT_scaffold_470_10629-10650

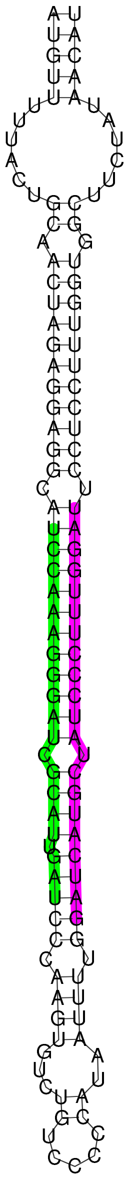

TCCAAAGGGATCGCATTGAT_LG_VIII_3573016-3573035

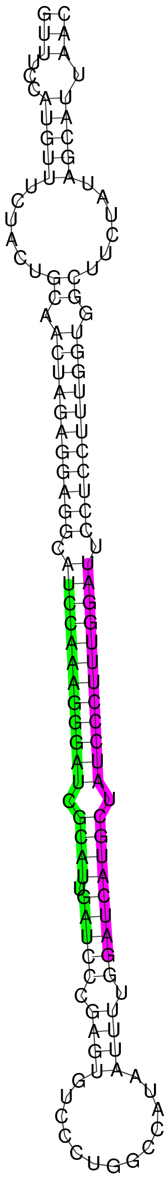

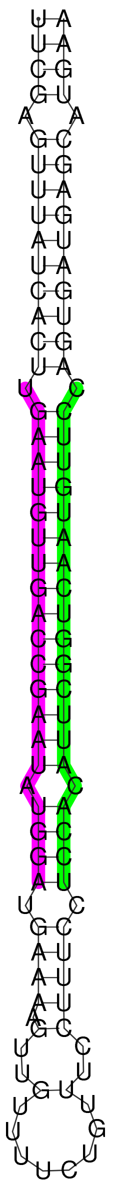

TCCACATTCGGTCAATGTTCC_LG_VIII_9369757-9369777

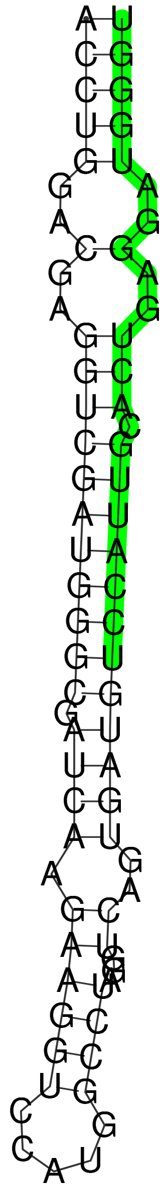

TCCATTGCACTGAGGATGGGT_scaffold_1952_9350-9370

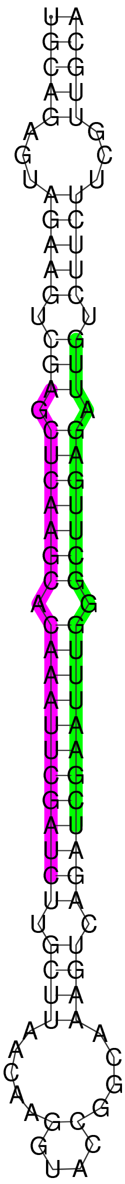

TCGAATTTGGGCTTGAGATTG_LG_III_7559439-7559459

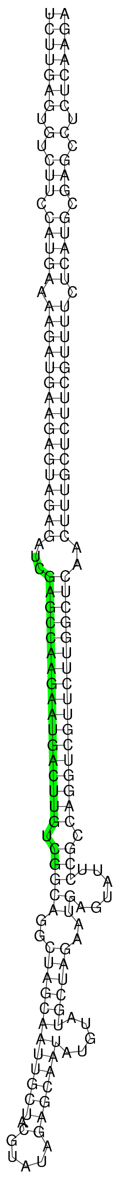

TCGAGCCAAGAATGACTTGTCG_LG_XVIII_13370751-13370772

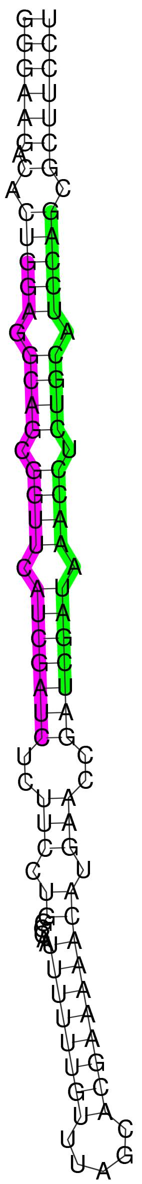

TCGATAAACCTCTGCATCCAG_LG_XII_5191536-5191556

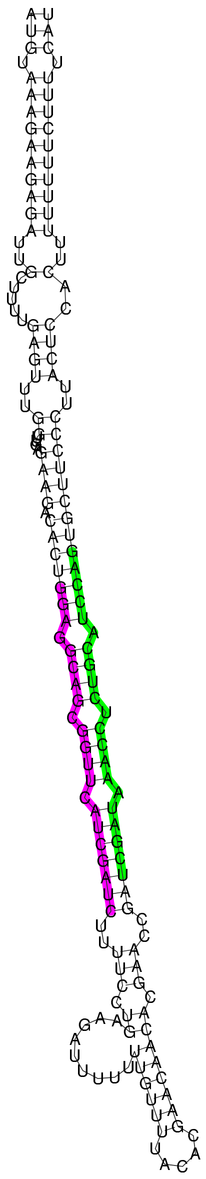

TCGATAAACCTCTGCATCCAG_scaffold_1061_6422-6442

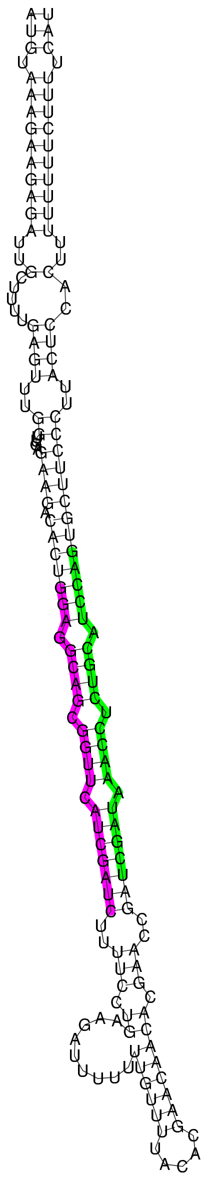

TCGATAAACCTCTGCATCCAG_scaffold_82_1335649-1335669

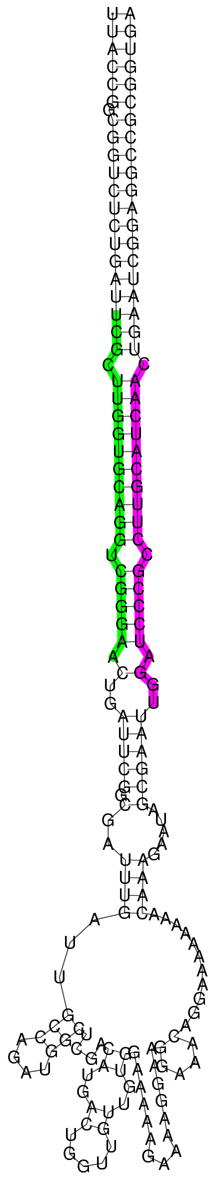

TCGCTTGGTGCAGGTCGGGAA_LG_III_2714691-2714711

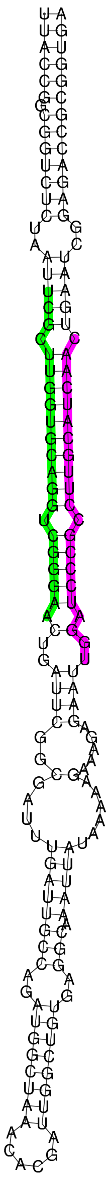

TCGCTTGGTGCAGGTCGGGAA_scaffold_86_1165679-1165699

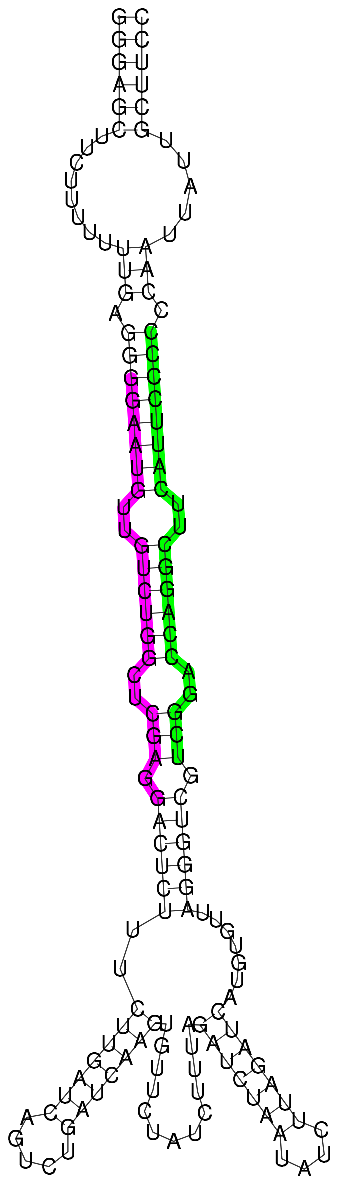

TCGGACCAGGCTTCATTCCCC_LG_II_13668251-13668271

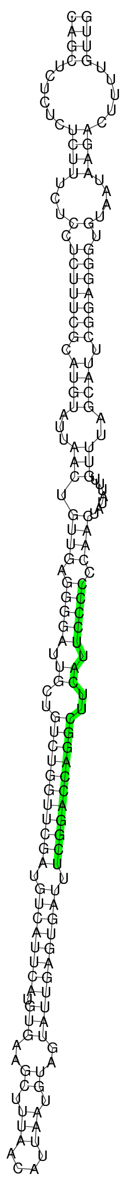

TCGGACCAGGCTTCATTCCCC_LG_II_2737702-2737722

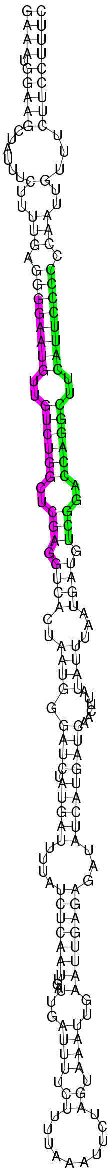

TCGGACCAGGCTTCATTCCCC_LG_I_6097059-6097079

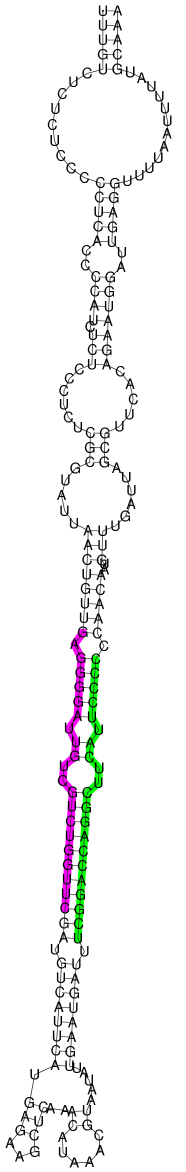

TCGGACCAGGCTTCATTCCCC_LG_V_15276190-15276210

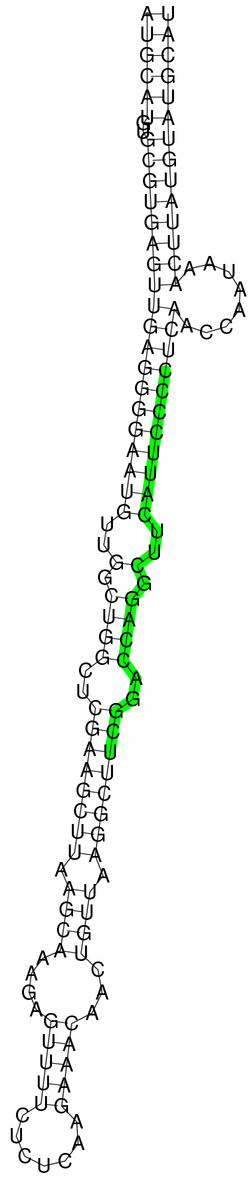

TCGGACCAGGCTTCATTCCCC_LG_V_824468-824488

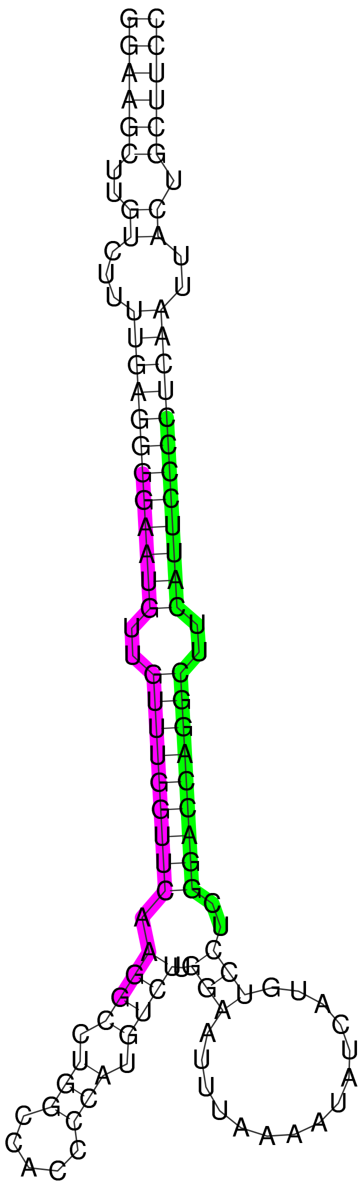

TCGGACCAGGCTTCATTCCCC_LG_XII_2546085-2546105

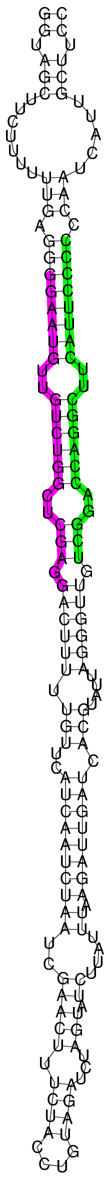

TCGGACCAGGCTTCATTCCCC_LG_XIV_3895190-3895210

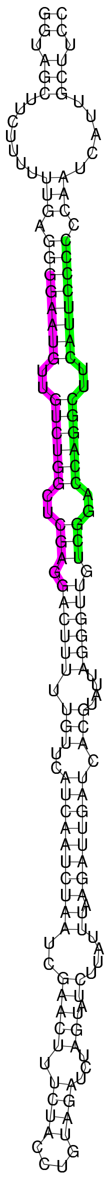

TCGGACCAGGCTTCATTCCCC_LG_XIV_3909159-3909179

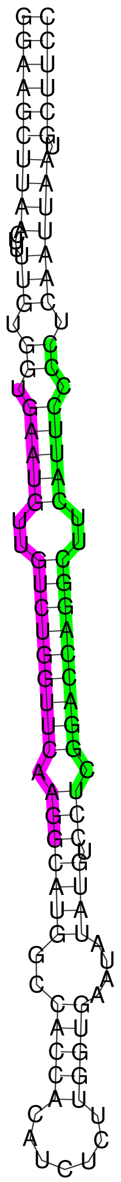

TCGGACCAGGCTTCATTCCCC_LG_XV_460376-460396

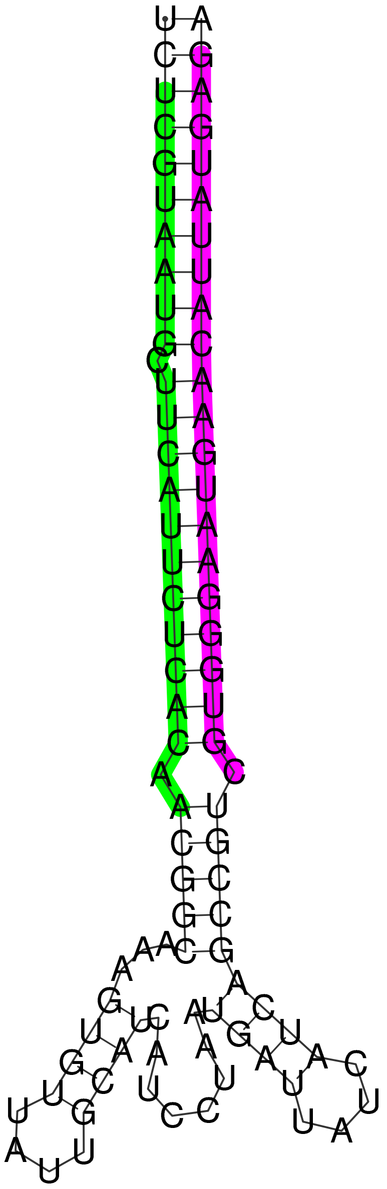

TCGTAATGCTTCATTCTCACAA_scaffold_148_201299-201320

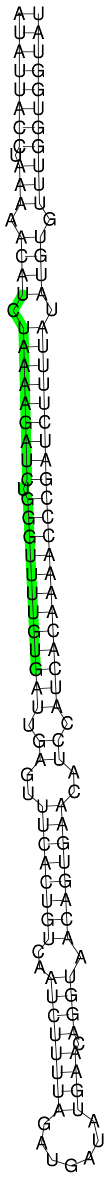

TCTAAAAGATCTGGGTTTTGTG_scaffold_64_35698-35719

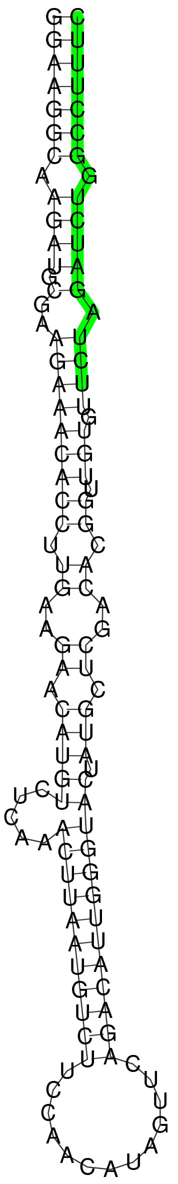

TCTAGATCTGGCCTTTCATT_scaffold_40_1939960-1939979

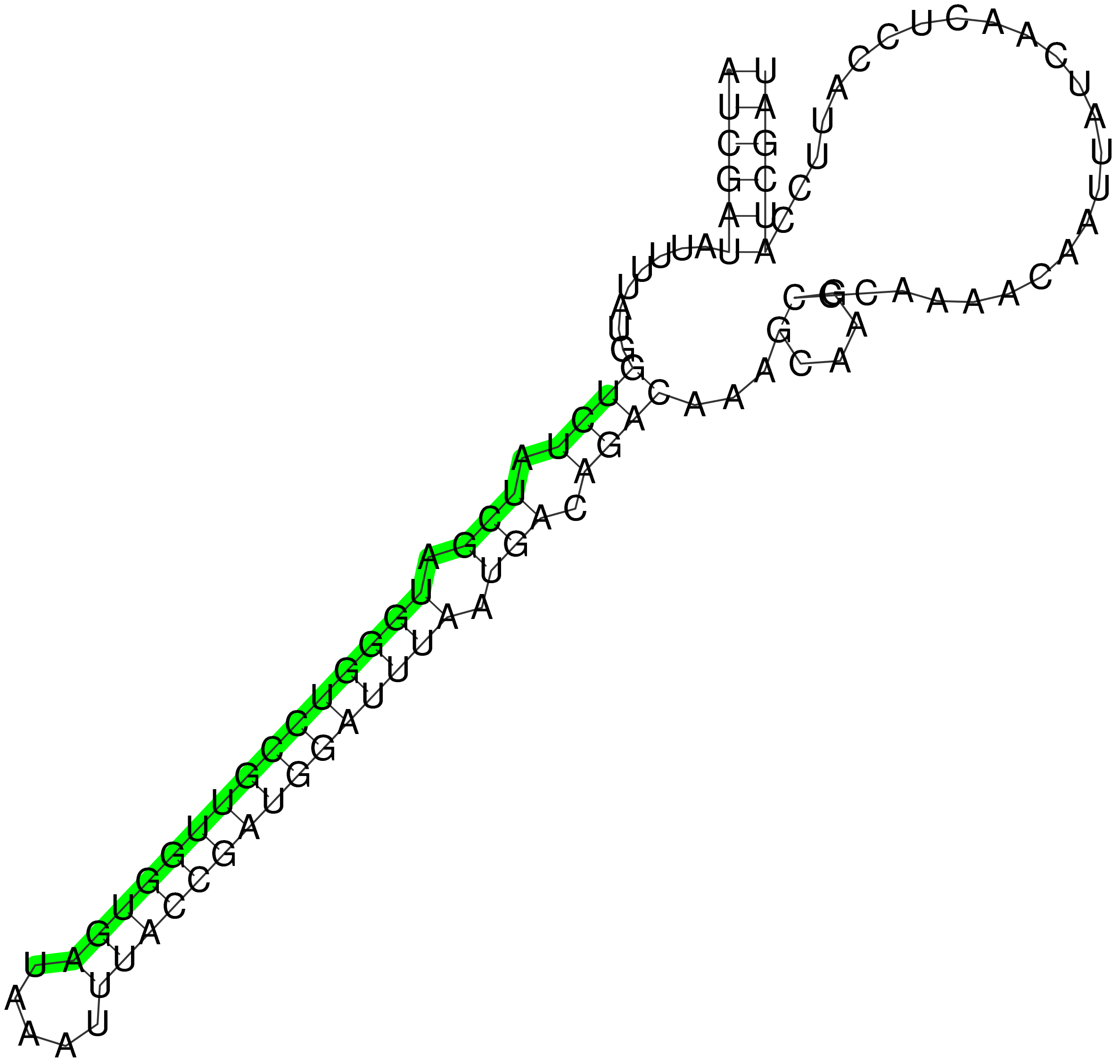

TCTATCGATGGGTCCGTTGGTGAT_LG_III_16952013-16952036

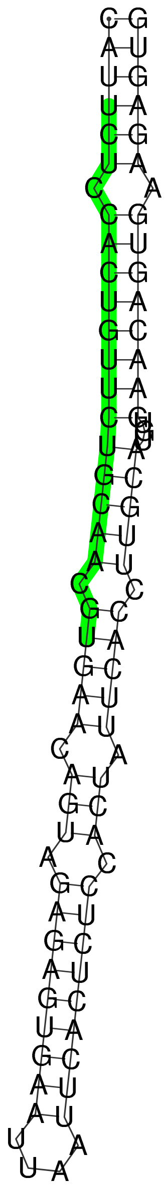

TCTCCACTGTTCTGCAACGT_LG_III_5893379-5893398

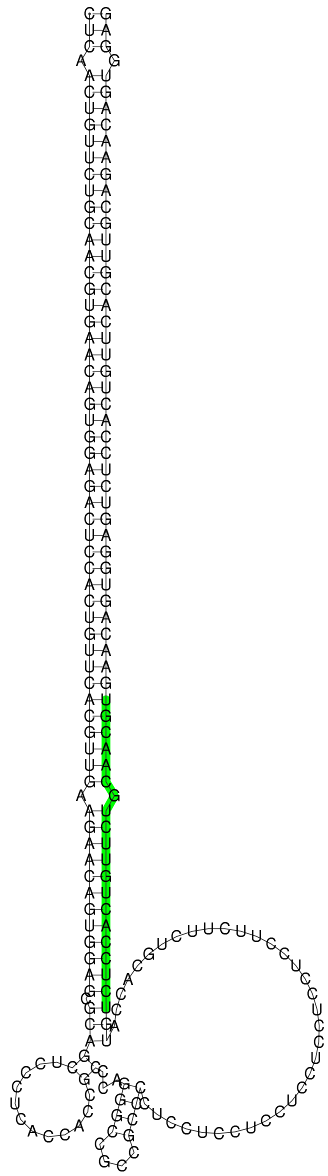

TCTCCACTGTTCTGCAACGT_LG_XVIII_3965638-3965657

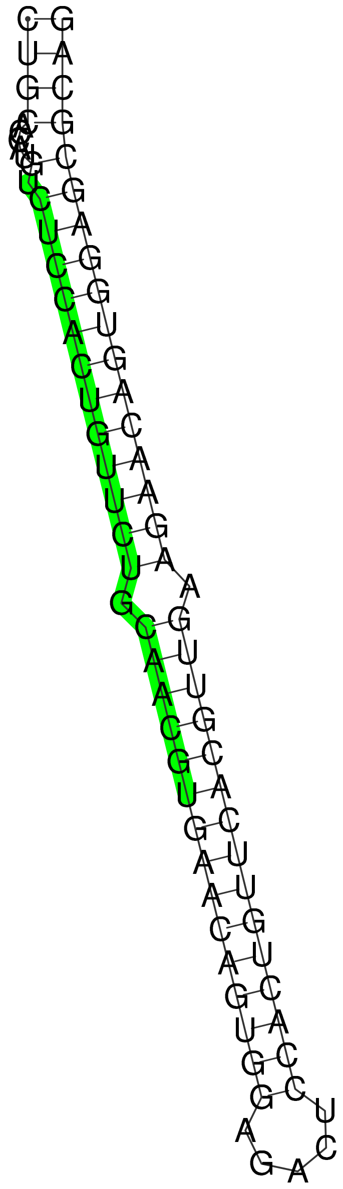

TCTCCACTGTTCTGCAACGT_LG_XVI_5585042-5585061

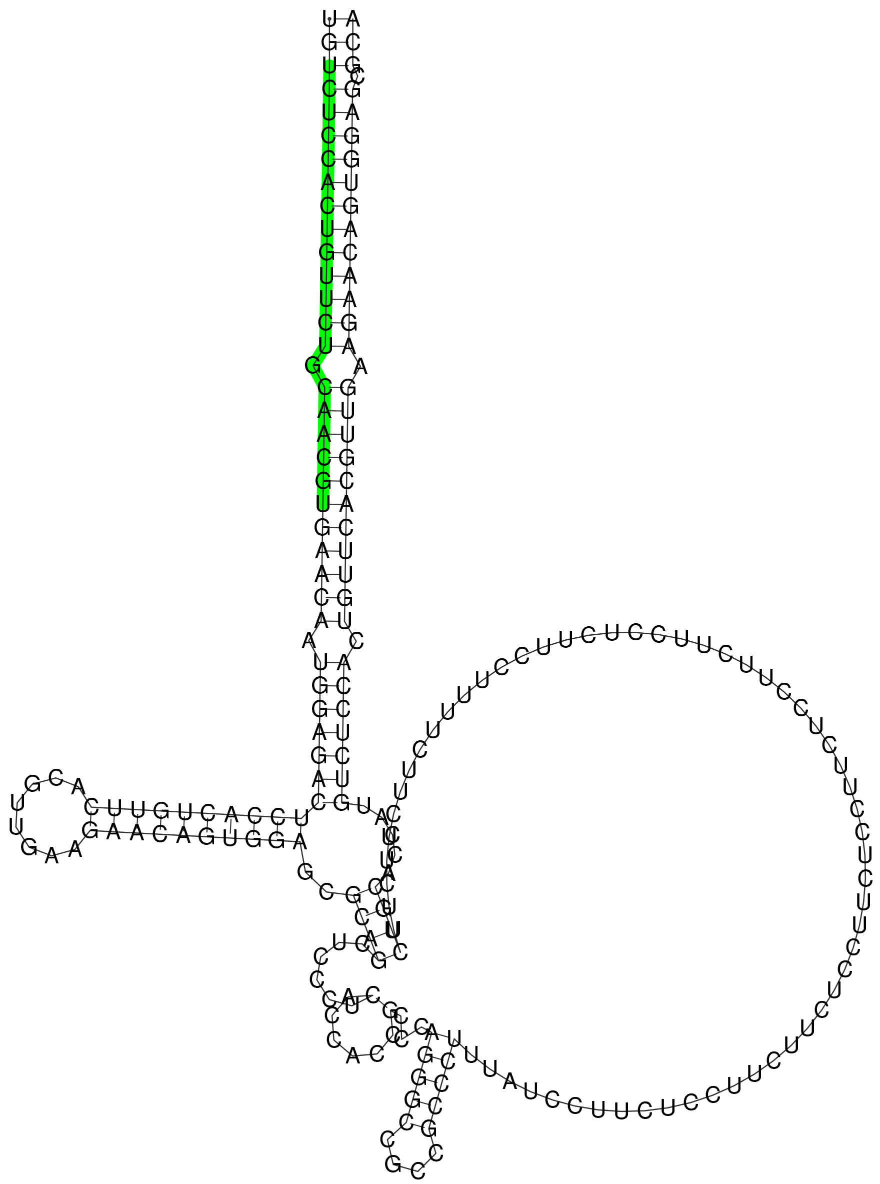

TCTCCACTGTTCTGCAACGT_LG_X_8696325-8696344

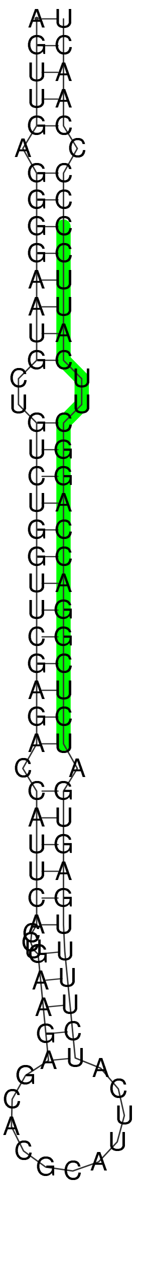

TCTCGGACCAGGCTTCATTCC_LG_VIII_11091596-11091616

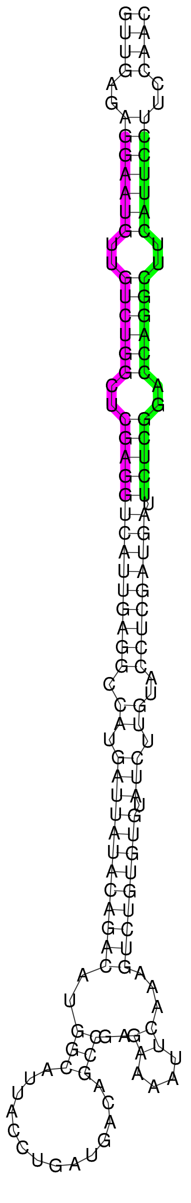

TCTCGGACCAGGCTTCATTCC_LG_VII_3394997-3395017

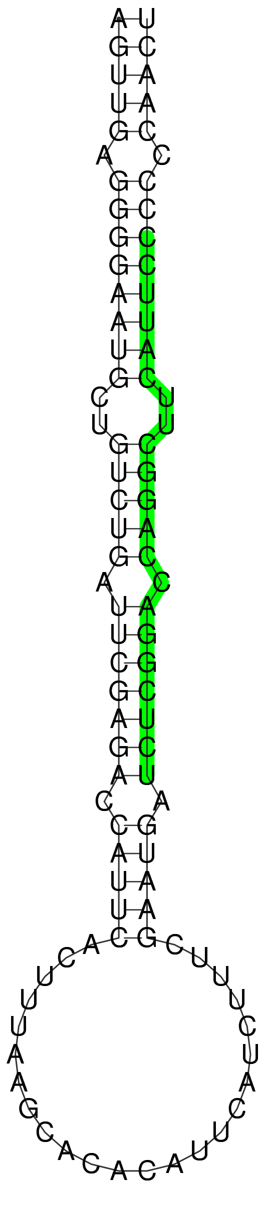

TCTCGGACCAGGCTTCATTCC_LG_X_8492340-8492360

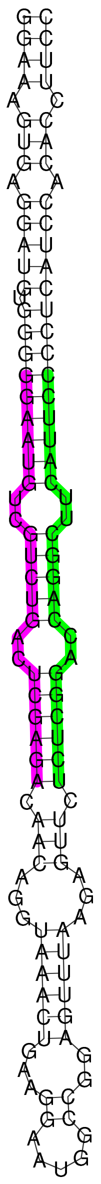

TCTCGGACCAGGCTTCATTCC_scaffold_122_445120-445140

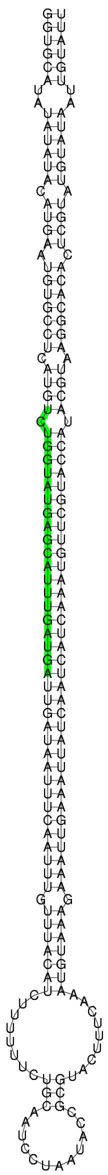

TCTGGTATGAGCATTTGATGA_LG_IV_12536788-12536808

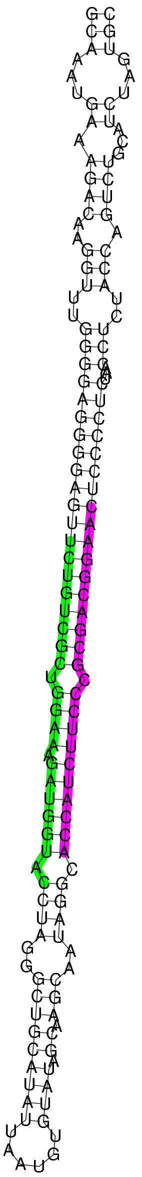

TCTGTCGCTGGAAAGATGGTAC_LG_XVII_3400803-3400824

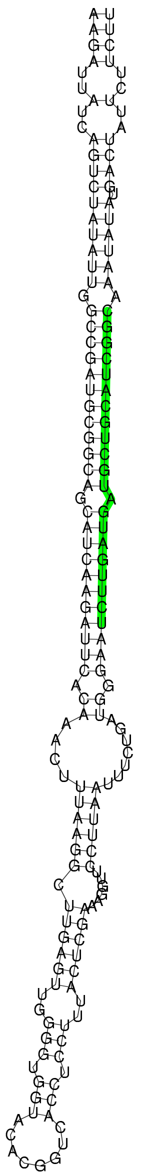

TCTTGATGATGCTGCATCGGC_LG_IX_8453677-8453697

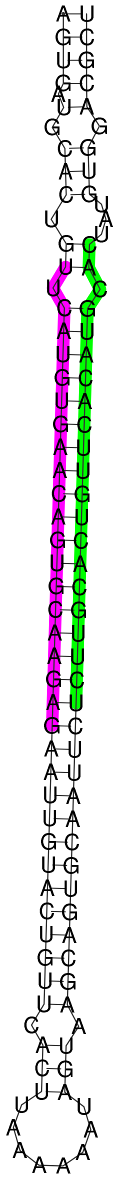

TCTTGCACTGTTCACATGCAC_LG_XIX_9631802-9631822

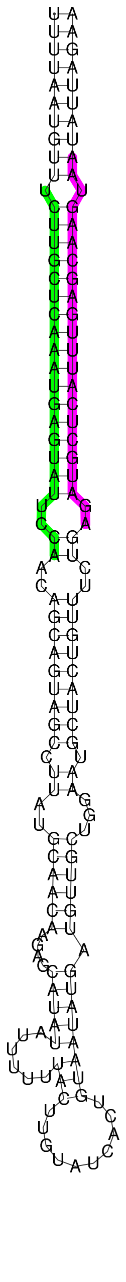

TCTTGCTCAAATGAGTATTCCA_LG_XII_2137739-2137760

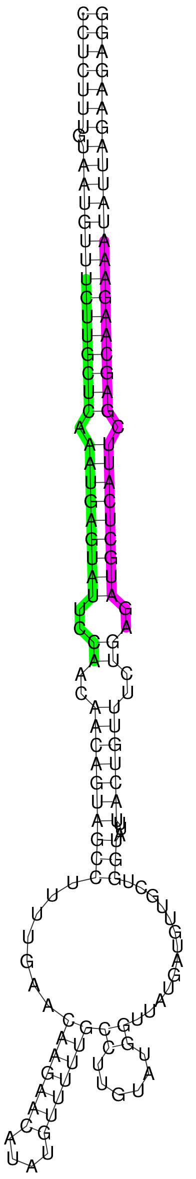

TCTTGCTCAAATGAGTATTCCA_LG_XV_698203-698224

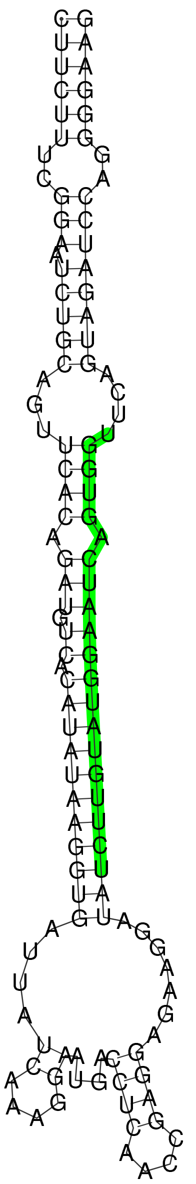

TCTTGTATGGAATCAGTGGT_LG_XVI_4514874-4514893

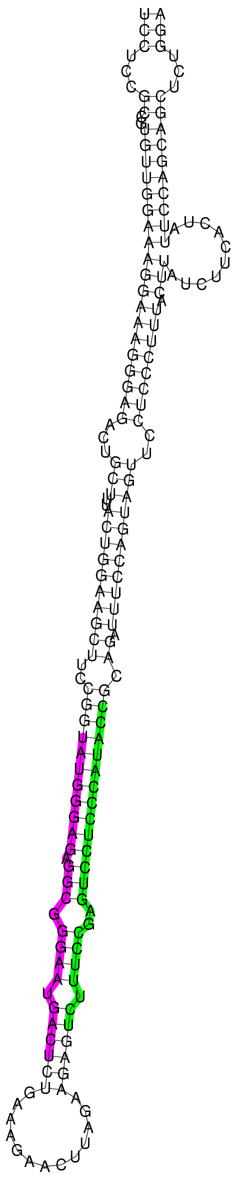

TCTTTCCGAGTCCTCCCATACC_scaffold_155_237816-237837

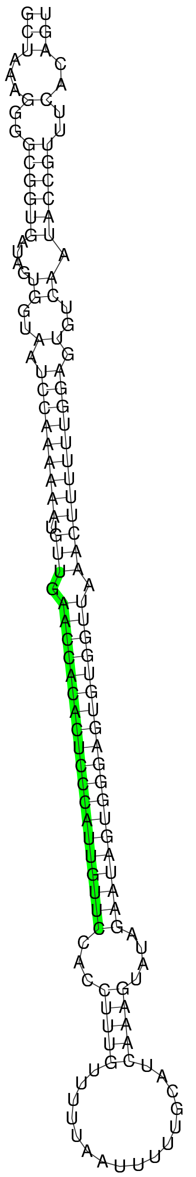

TGAACCACACTCCCATTGTTC_LG_II_22854585-22854605

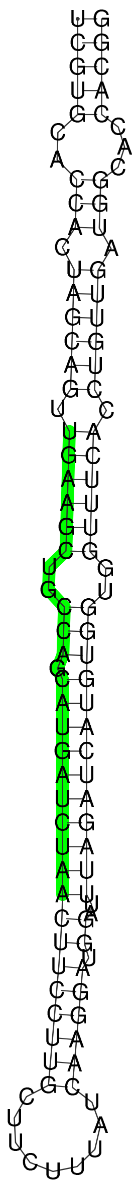

TGAAGCTGCCAGCATGATCTAA_LG_II_3054975-3054996

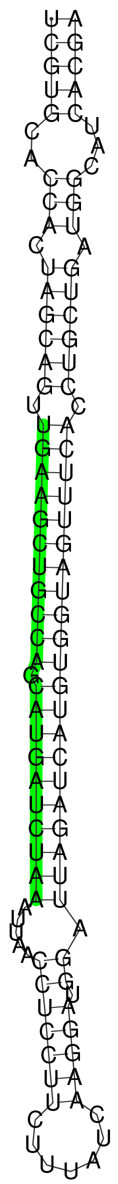

TGAAGCTGCCAGCATGATCTAA_LG_V_14949534-14949555

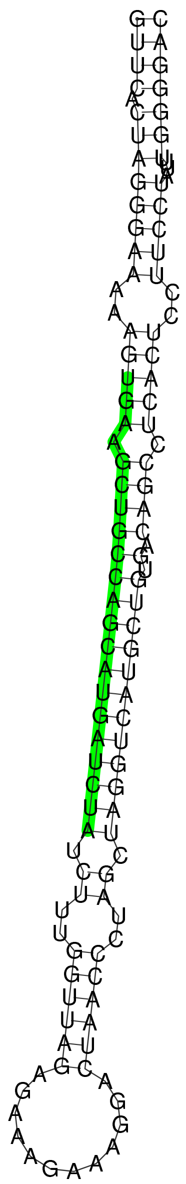

TGAAGCTGCCAGCATGATCTA_LG_II_3057574-3057594

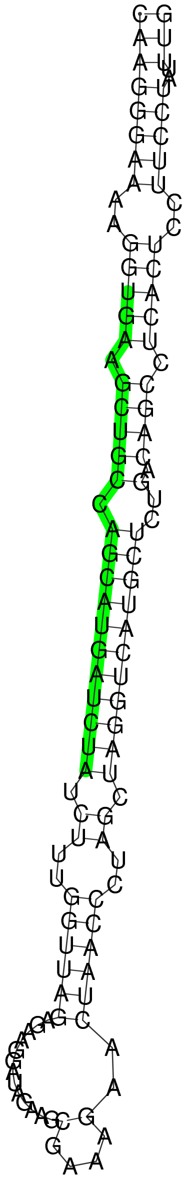

TGAAGCTGCCAGCATGATCTA_scaffold_14274_265-285

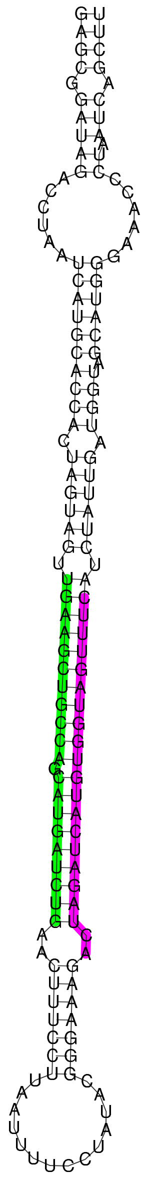

TGAAGCTGCCAGCATGATCTG_scaffold_70_484740-484760

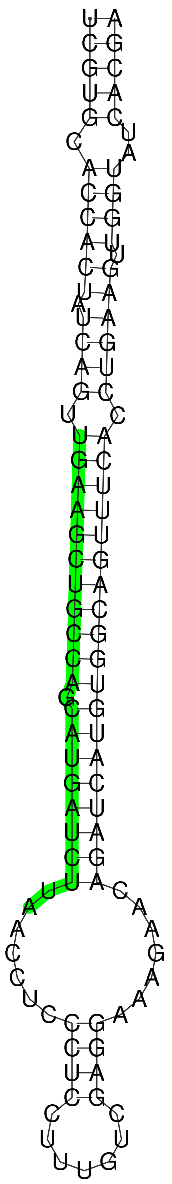

TGAAGCTGCCAGCATGATCTTA_LG_VIII_10761725-10761746

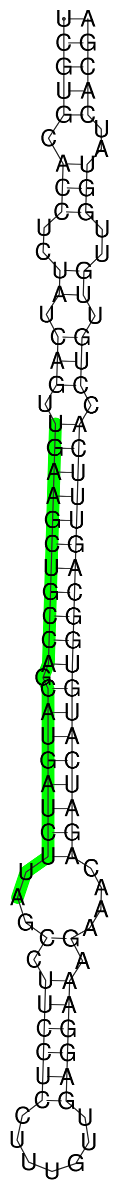

TGAAGCTGCCAGCATGATCTTA_LG_X_9042268-9042289

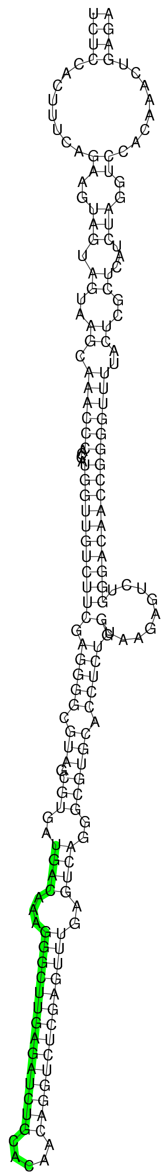

TGACAAAGGGCTTGAGATCTGCAC_LG_I_13848431-13848454

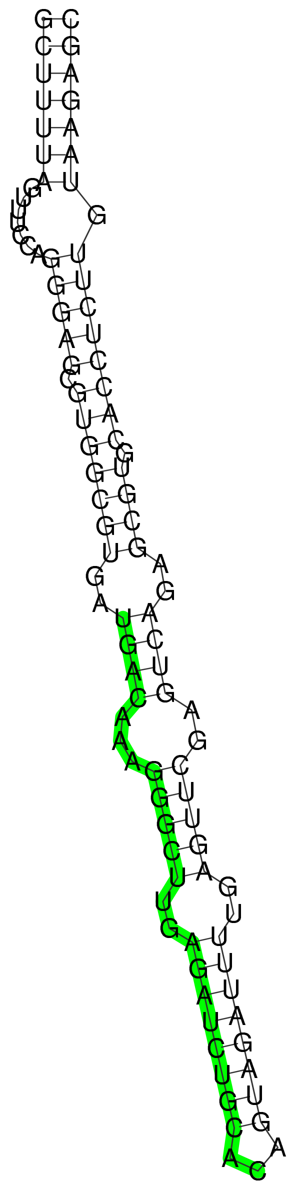

TGACAAAGGGCTTGAGATCTGCAC_LG_XI_8080050-8080073

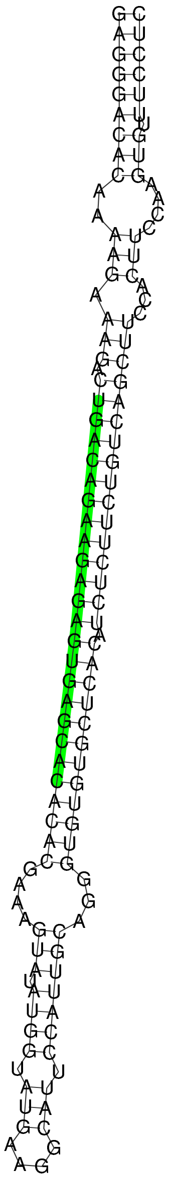

TGACAGAAGAGAGTGAGCAC_LG_VI_11740450-11740469

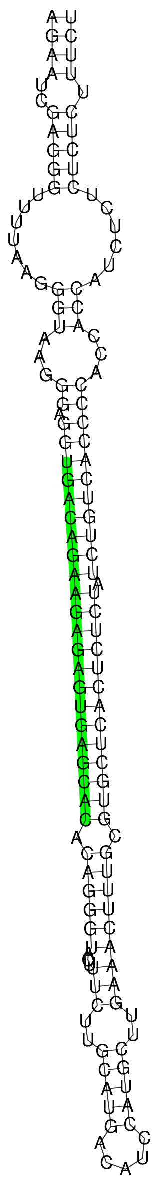

TGACAGAAGAGAGTGAGCAC_LG_VI_15099495-15099514

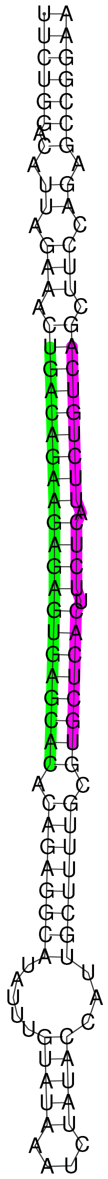

TGACAGAAGAGAGTGAGCAC_LG_VI_17927293-17927312

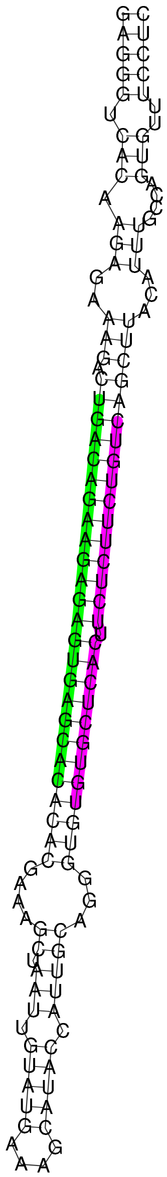

TGACAGAAGAGAGTGAGCAC_LG_XVIII_10944704-10944723

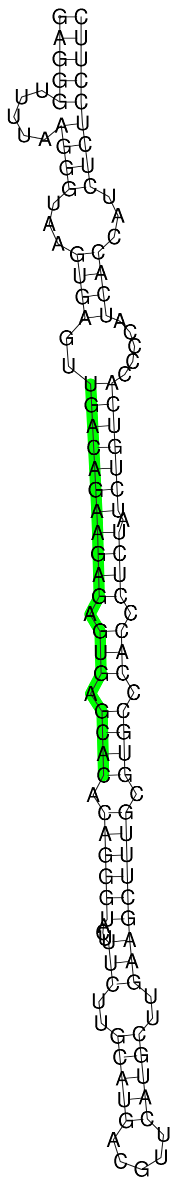

TGACAGAAGAGAGTGAGCAC_LG_XVIII_1365516-1365535

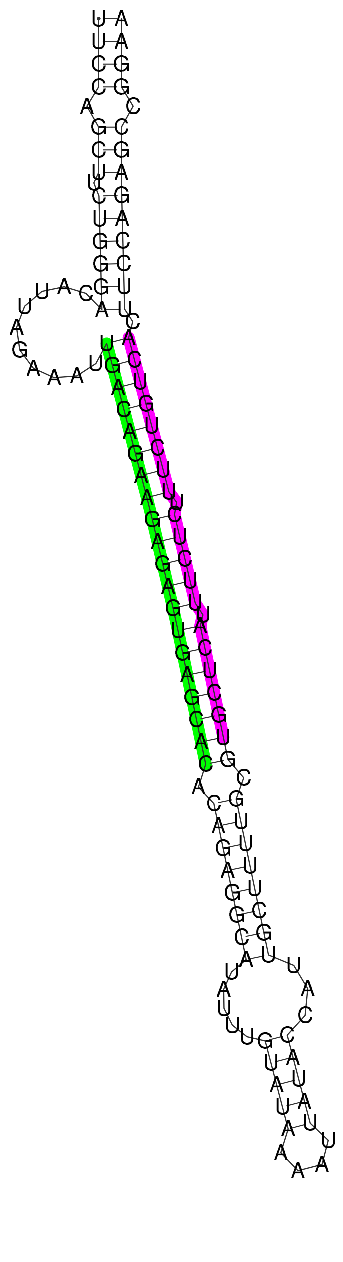

TGACAGAAGAGAGTGAGCAC_LG_XVIII_6181086-6181105

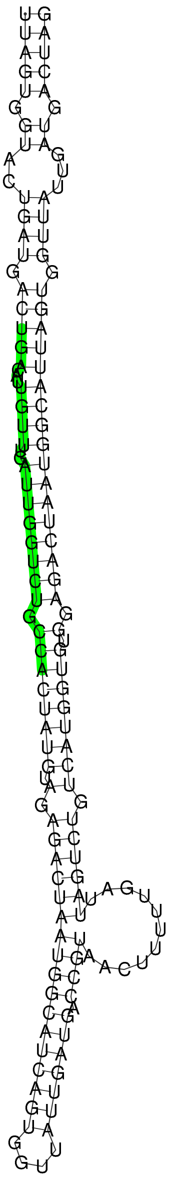

TGACATTGTTTGATTGGTCTGCCA_LG_XIV_12238435-12238458

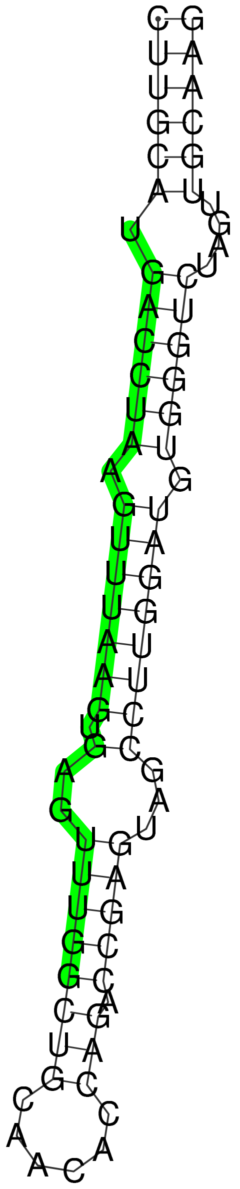

TGACCTAAGTTTAAGTGAGTTTGG_LG_VIII_15593921-15593944

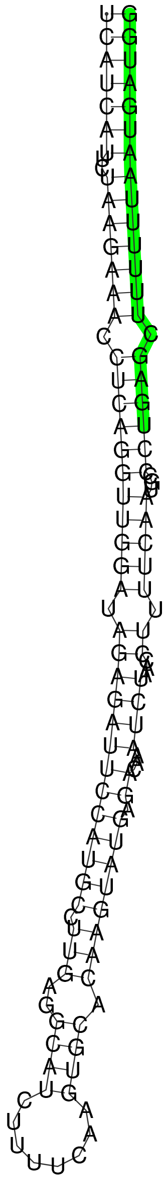

TGAGCTTTTTTAATGATGGTA_LG_XIX_191973-191993

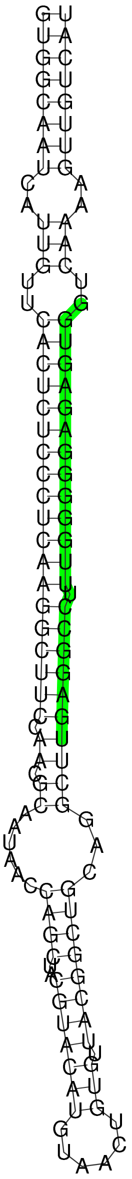

TGAGGCCTTTGGGGGAGAGTGG_LG_XIV_3370170-3370191

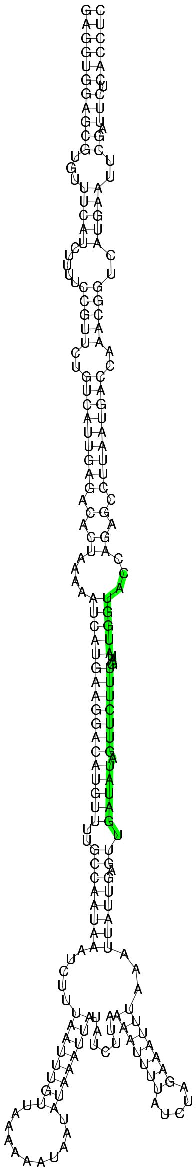

TGATATAGTTCTTTGATATGGTAC_LG_X_14442590-14442613

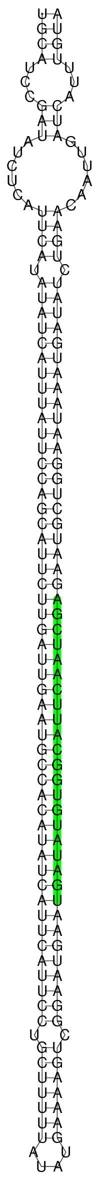

TGATATGTGGCATTCAATCGA_LG_III_13687073-13687093

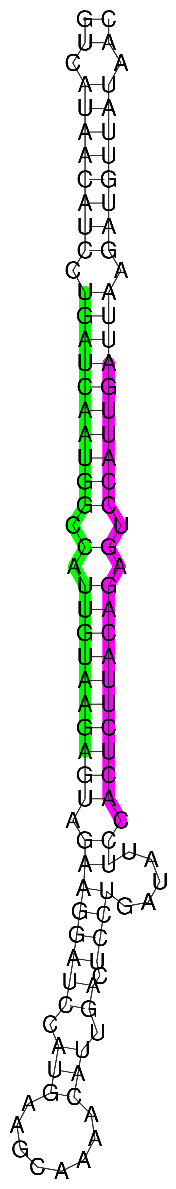

TGATCAATGGCCATTGTAAGA_LG_VIII_8026943-8026963

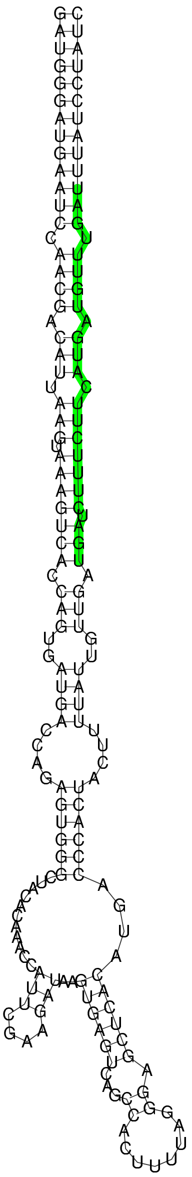

TGATCTTTCTTCATGATGTTTGAT_scaffold_97_323004-323027

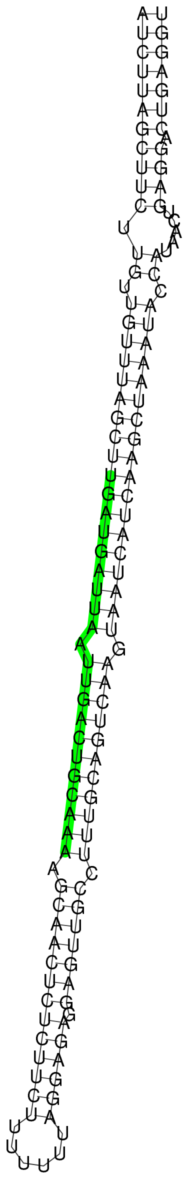

TGATGATTAATTGACTGCAAA_LG_I_22370578-22370598

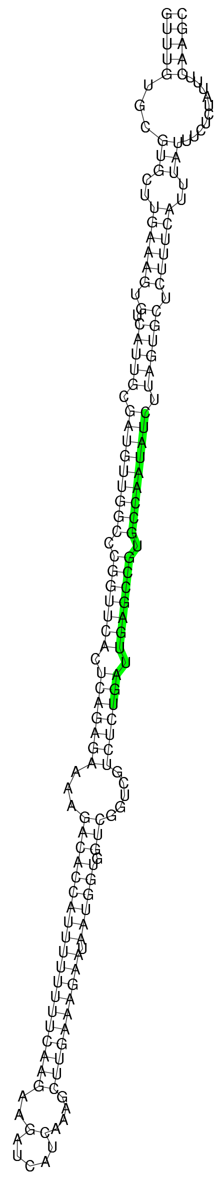

TGATTGAGCCGTGCCAATATC_LG_II_902914-902934

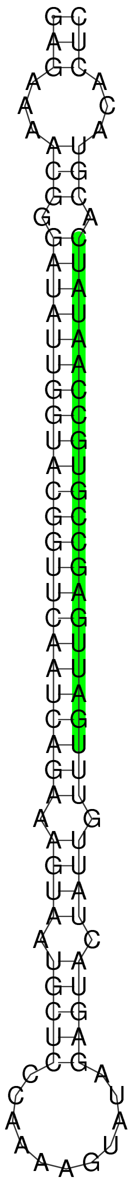

TGATTGAGCCGTGCCAATATC_LG_IV_185072-185092

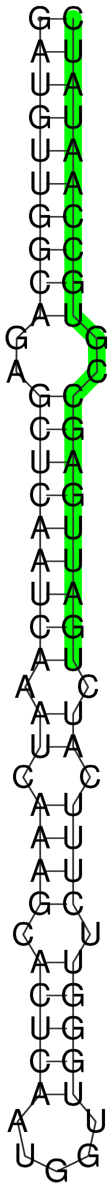

TGATTGAGCCGTGCCAATATC_LG_I_24172982-24173002

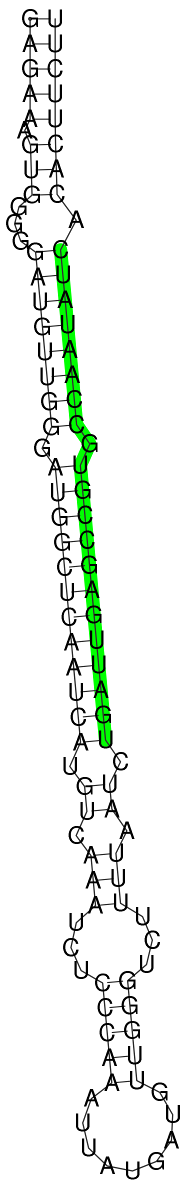

TGATTGAGCCGTGCCAATATC_LG_XII_11612120-11612140

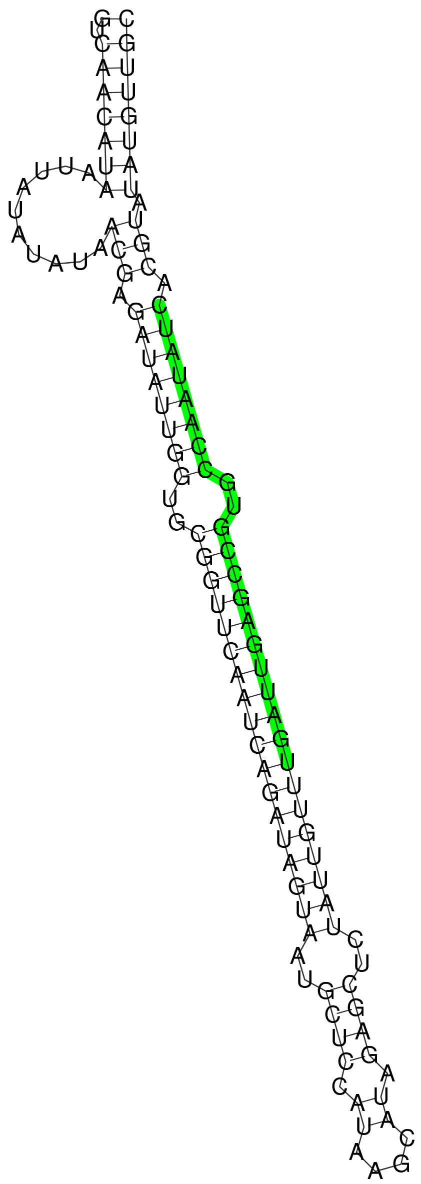

TGATTGAGCCGTGCCAATATC_LG_XI_286909-286929

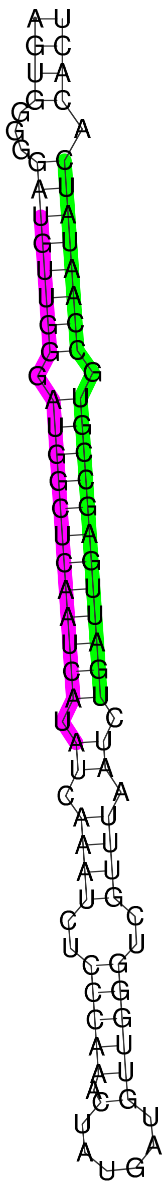

TGATTGAGCCGTGCCAATATC_LG_XV_7305205-7305225

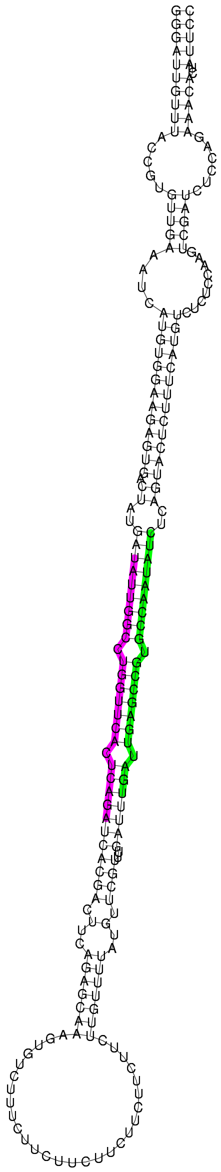

TGATTGAGCCGTGCCAATATC_scaffold_57_112845-112865

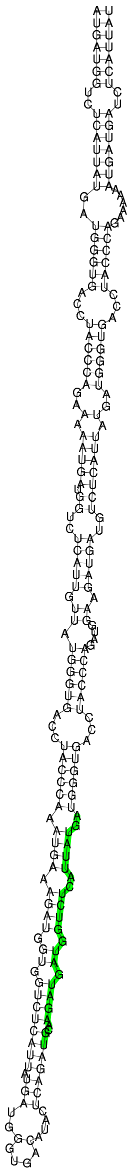

TGCAAGATGATGGTCTCATTATGA_scaffold_5480_1592-1615

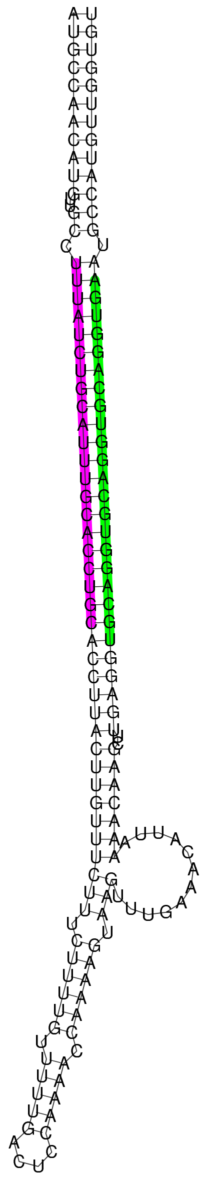

TGCAGGTGCAGGTGCAGGTGA_LG_IX_11430385-11430405

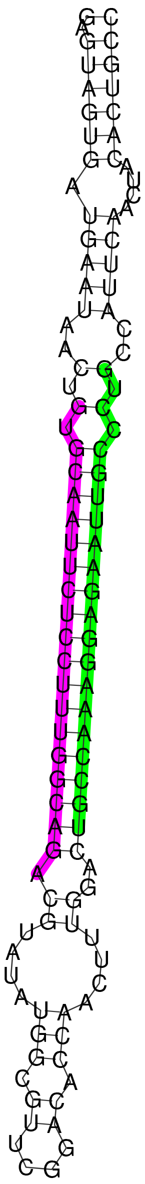

TGCCAAAGGAGAATTGCCCTG_LG_IV_3407431-3407451

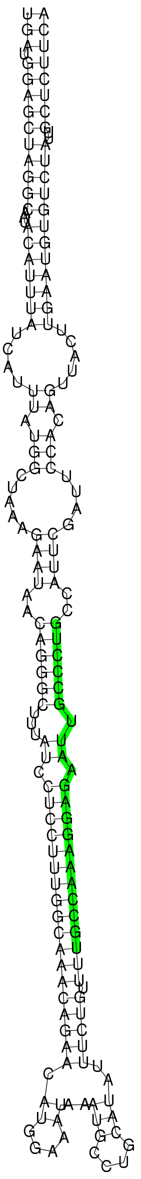

TGCCAAAGGAGAATTGCCCTG_LG_IV_3453467-3453487

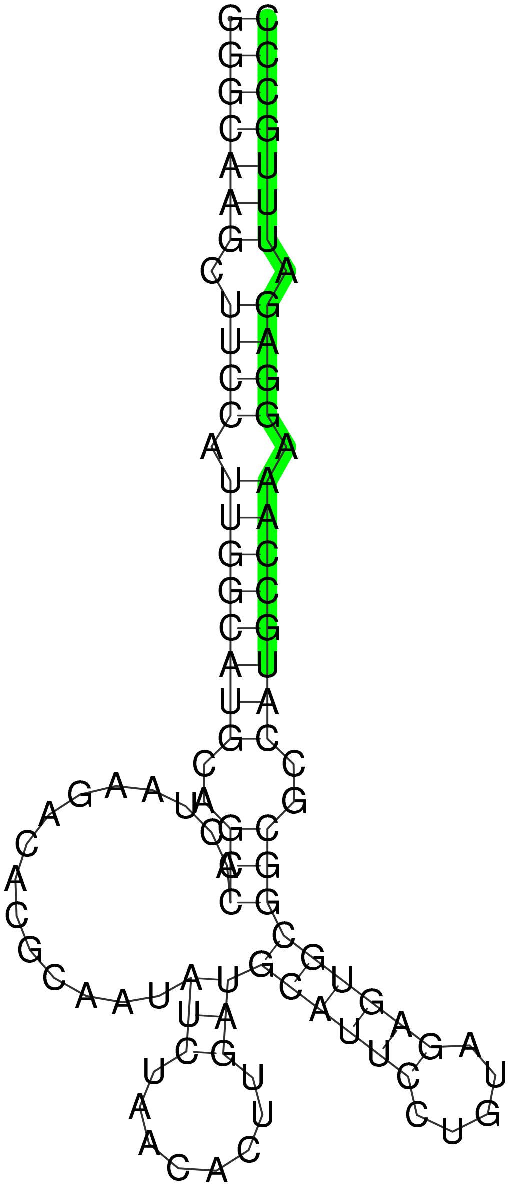

TGCCAAAGGAGATTTGCCCGG_LG_IV_3411452-3411472

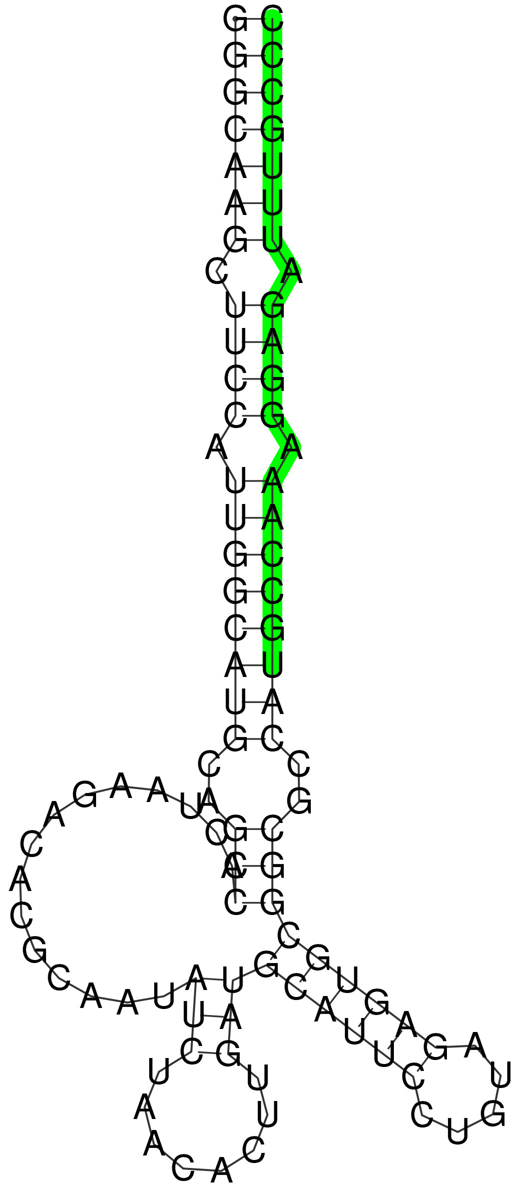

TGCCAAAGGAGATTTGCCCGG_scaffold_495_24571-24591

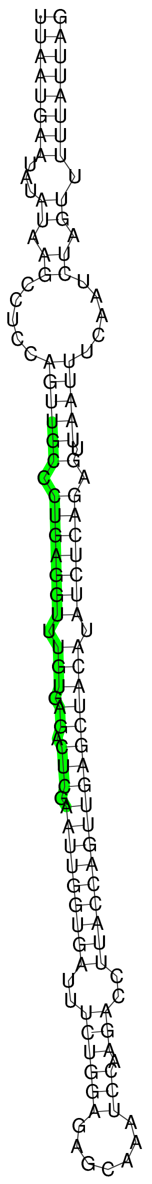

TGCCCTGAGGTTTGTGAGACTCGA_LG_XVII_233135-233158

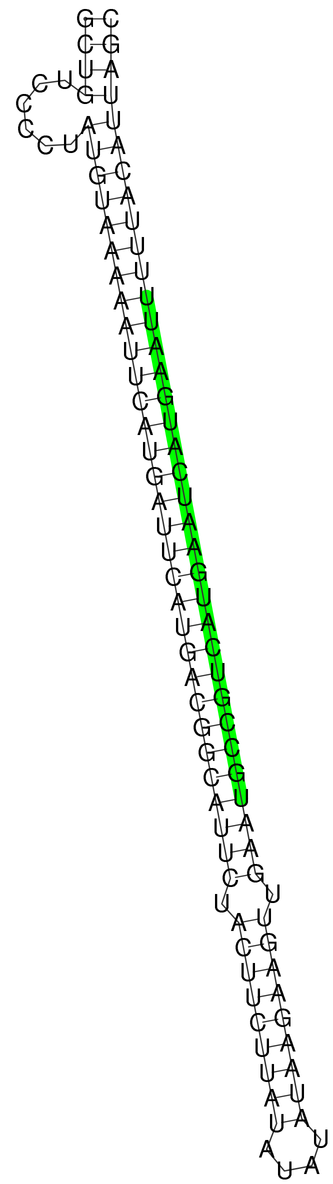

TGCCGTCATGAATCATGAATT_scaffold_202_227670-227690

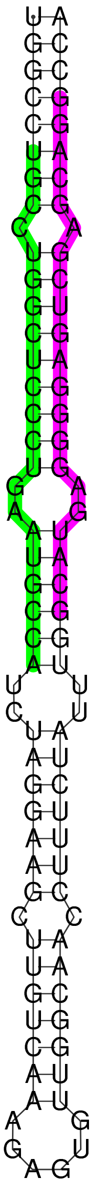

TGCCTGGCTCCCTGAATGCCA_LG_VI_181602-181622

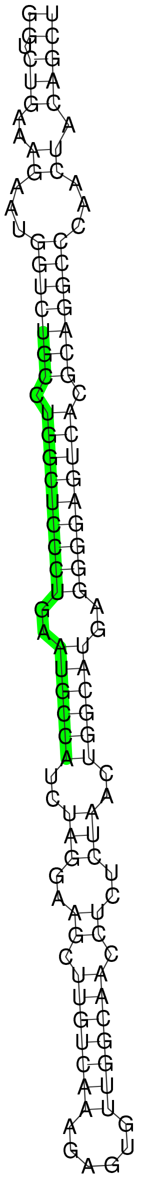

TGCCTGGCTCCCTGAATGCCA_scaffold_137_187285-187305

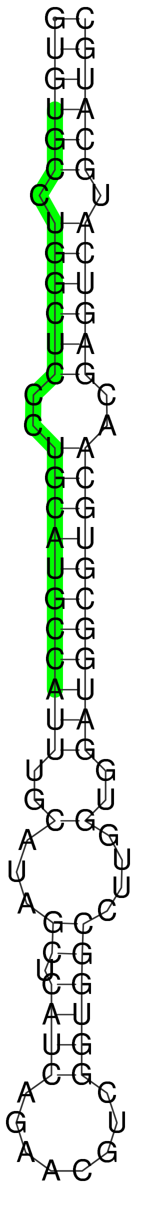

TGCCTGGCTCCCTGCATGCCA_LG_XVI_7014378-7014398

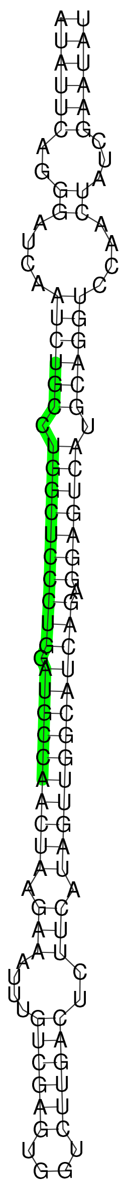

TGCCTGGCTCCCTGGATGCCA_LG_XIV_9829743-9829763

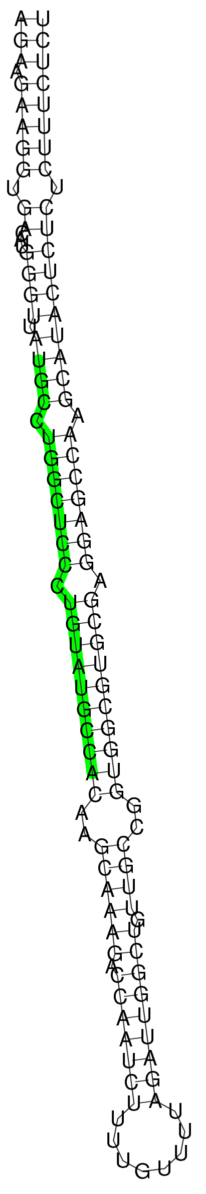

TGCCTGGCTCCCTGTATGCCA_LG_I_32813190-32813210

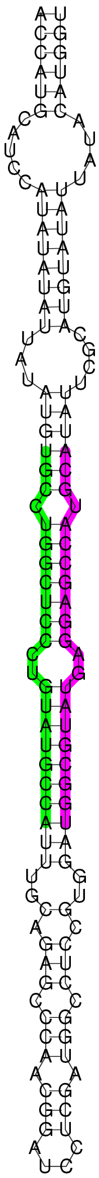

TGCCTGGCTCCCTGTATGCCA_LG_VIII_2097927-2097947

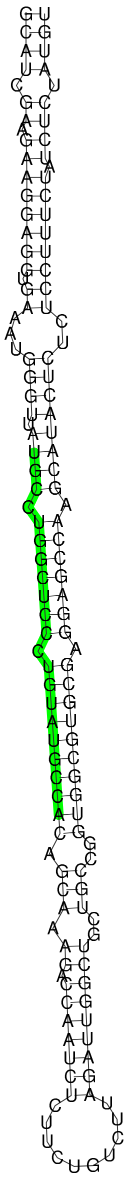

TGCCTGGCTCCCTGTATGCCA_LG_XI_14495641-14495661

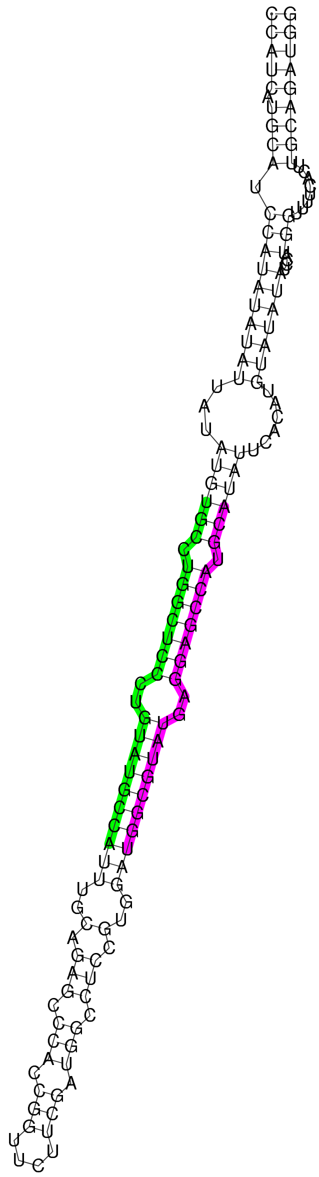

TGCCTGGCTCCCTGTATGCCA_LG_X_18982841-18982861

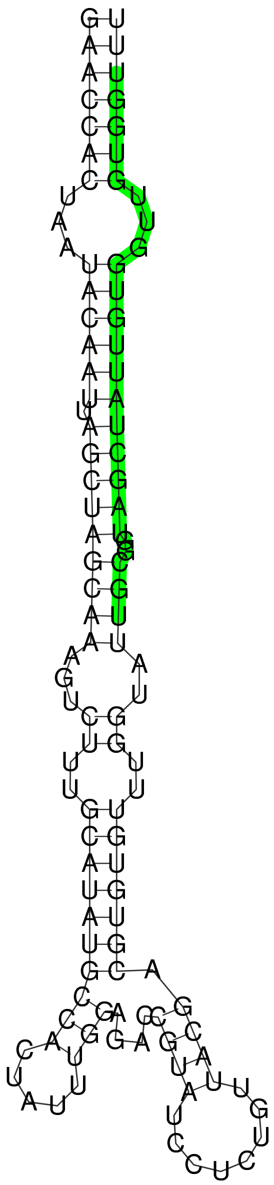

TGCGGTAGCTATTGTGGTTGTGGT_LG_IV_182972-182995

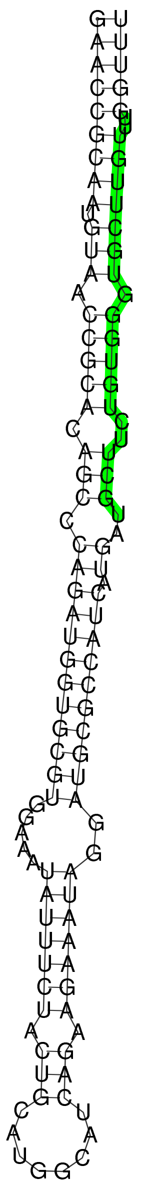

TGCTTCTGTGGGTGCTTGTTT_scaffold_118_304162-304182

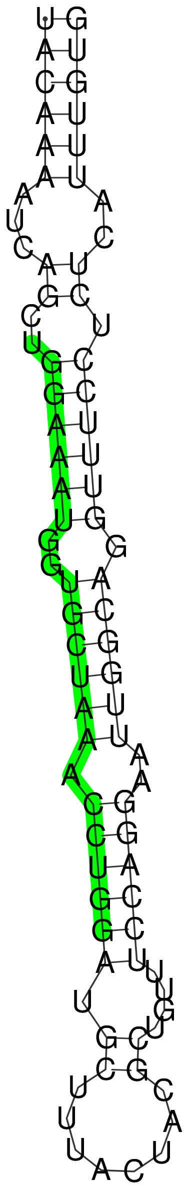

TGGAAATGGTGCTAAACCTGG_LG_XIV_7469686-7469706

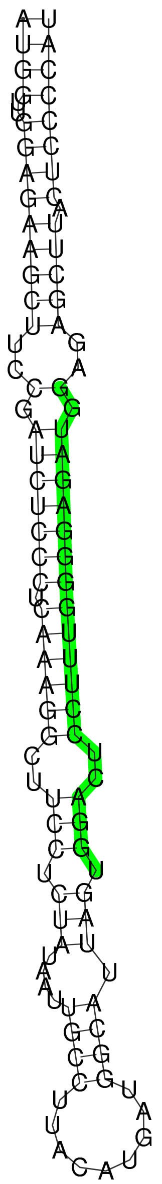

TGGACTCCTTTGGGGAGATGG_LG_XV_8088583-8088603

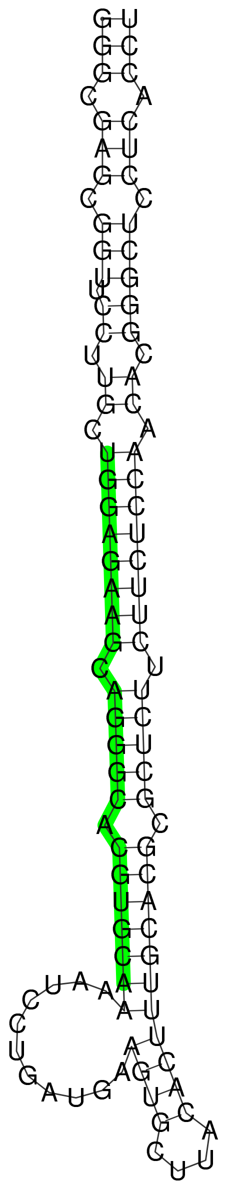

TGGAGAAGCAGGGCACGTGCA_LG_II_15132562-15132582

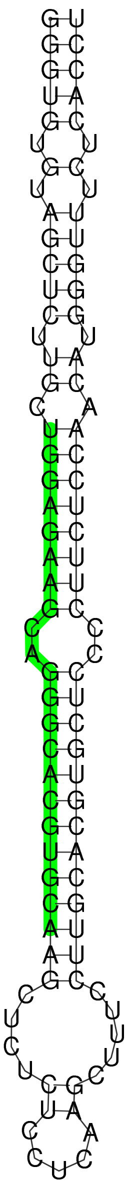

TGGAGAAGCAGGGCACGTGCA_LG_XIII_1040249-1040269

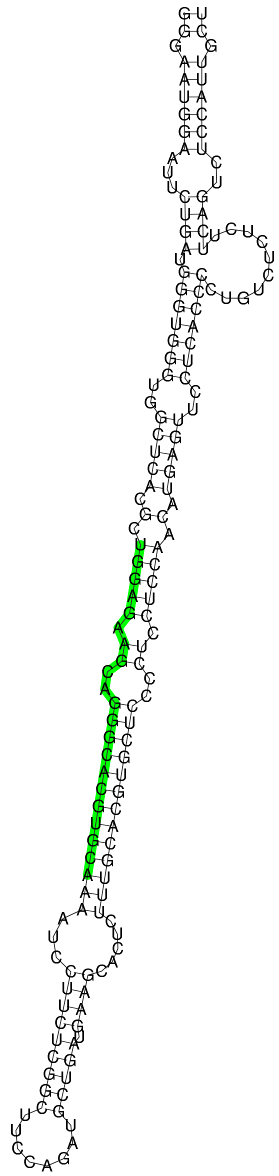

TGGAGAAGCAGGGCACGTGCA_LG_XIV_5728490-5728510

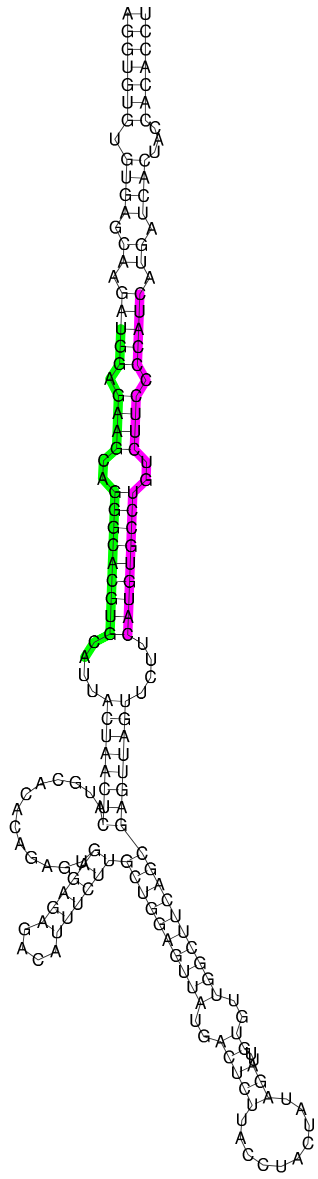

TGGAGAAGCAGGGCACGTGCA_LG_XVI_12388887-12388907

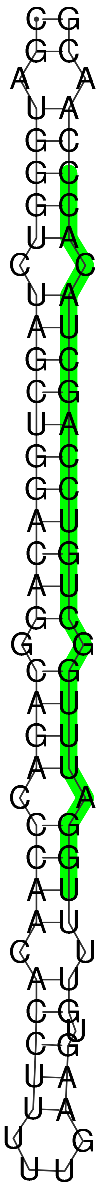

TGGATTTGGCTGTCCAGCTACACC_scaffold_15653_291-314

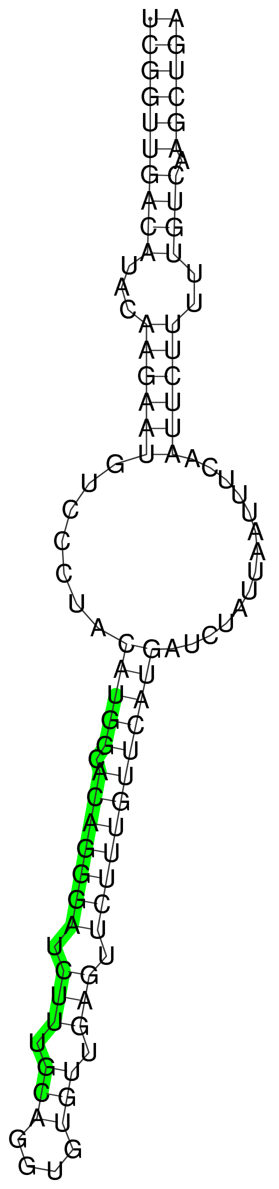

TGGCACAGGGATCTTTGC_LG_XVIII_5344048-5344065

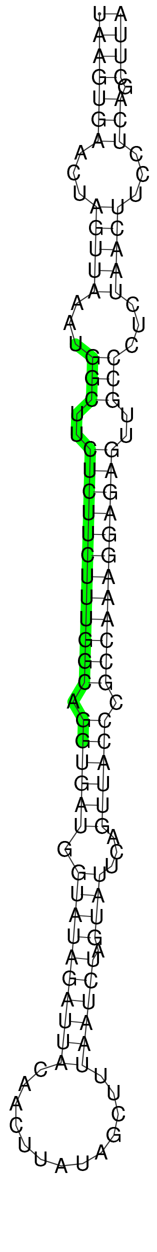

TGGCTTCTCTTCTTTGGCAGG_LG_III_11510241-11510261

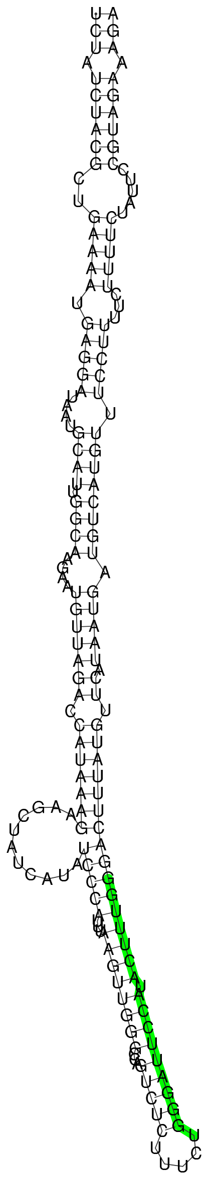

TGGGATTCCATACTTTGG_scaffold_286_24502-24519

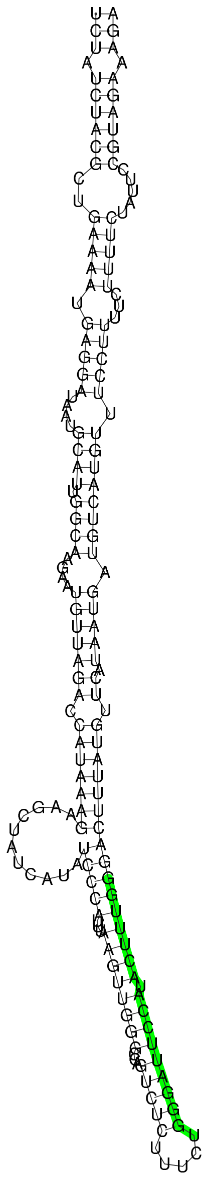

TGGGATTCCATACTTTGG_scaffold_451_43213-43230

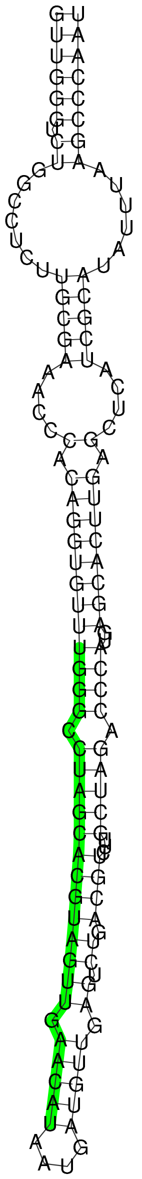

TGGGCCTAGCACGTAGTTGAACAT_LG_VII_4489225-4489248

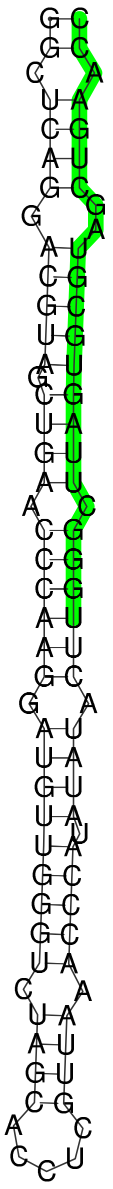

TGGGCTTAGTGCGTAGCTGAACC_LG_II_17506497-17506519

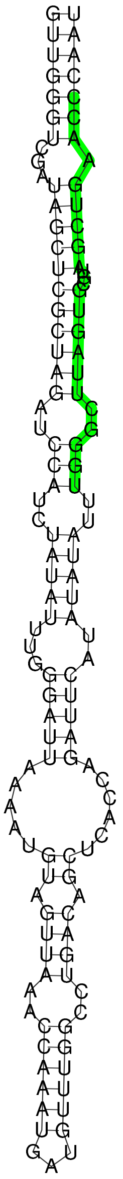

TGGGCTTAGTGCGTAGCTGAACC_LG_VII_4188297-4188319

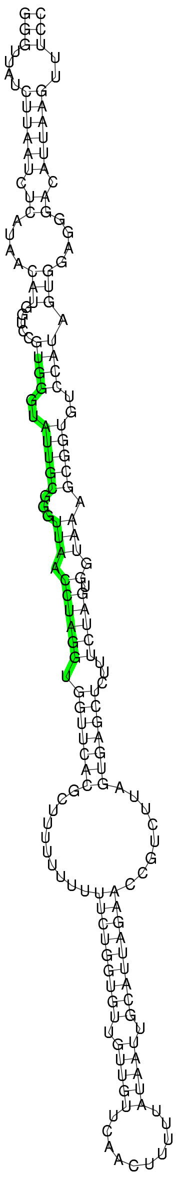

TGGGTATTGCGGGTTAACCTAGGT_LG_III_12473167-12473190

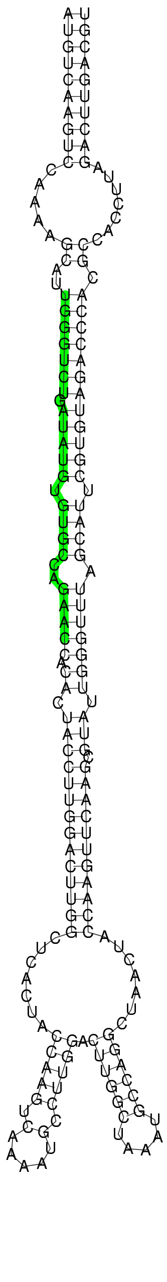

TGGGTCTGATATGTGTGCCAGAAC_scaffold_41_1725238-1725261

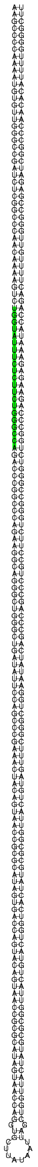

TGGTATTTCTCTTCTGGGCCA_scaffold_206_319099-319119

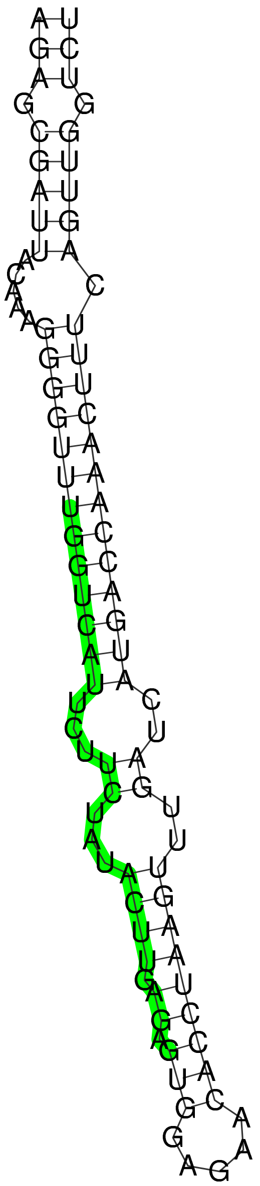

TGGTCATTCTTCTATACTTGAGAG_LG_XII_3300482-3300505

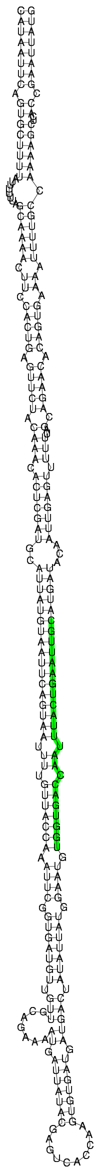

TGGTGACCAATTTACTGAATTGC_scaffold_64_1704184-1704206

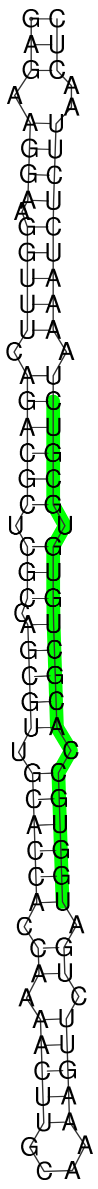

TGGTGCCACGCTGTGTGCGTC_LG_I_4481298-4481318

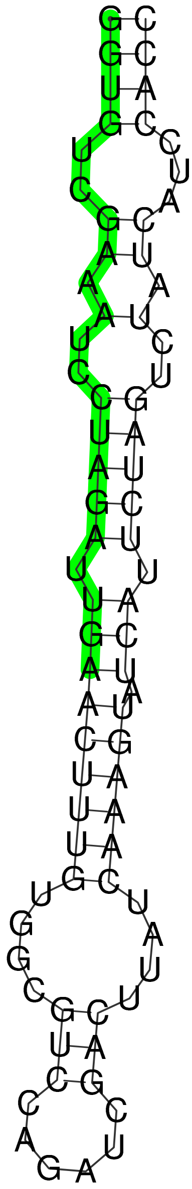

TGGTGTCGAAATCCTAGATTGA_LG_XII_6932248-6932269

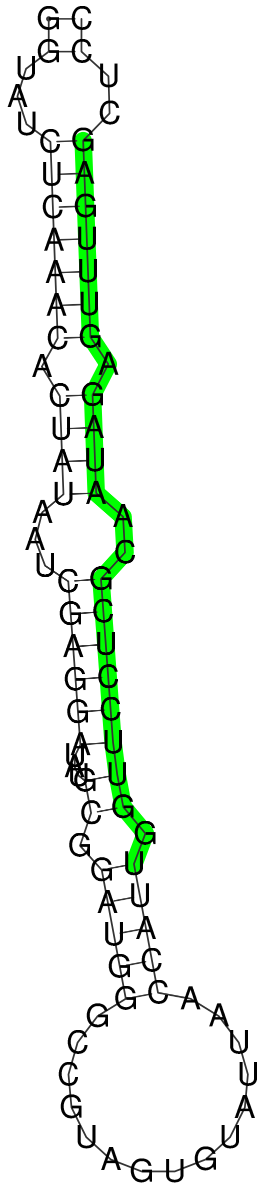

TGGTTCCTCGCAATAGAGTTTGAG_LG_XII_9288085-9288108

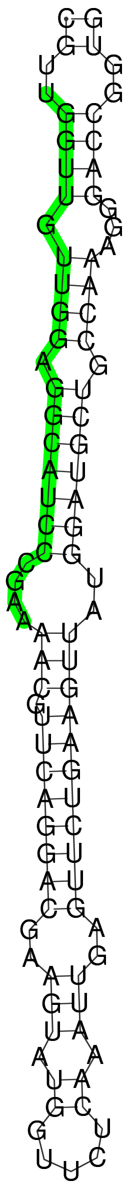

TGGTTGTTGGAGGCATCCCGAA_scaffold_130_767232-767253

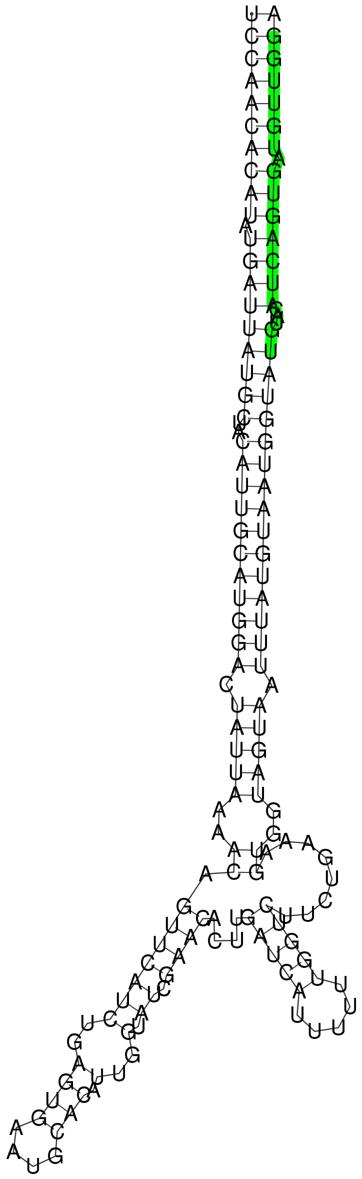

TGTACATCAGTGATGTTGG_LG_II_13066929-13066947

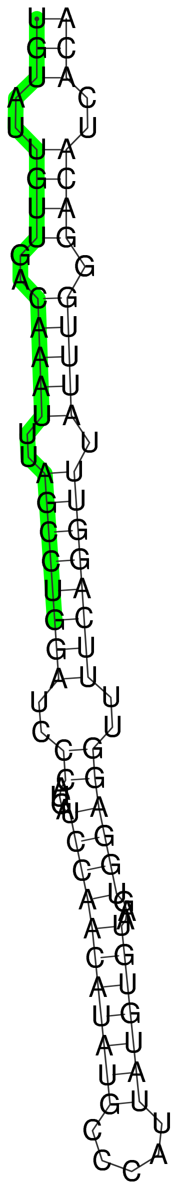

TGTATTGTTGACAAATTTAGCCTG_LG_V_12776990-12777013

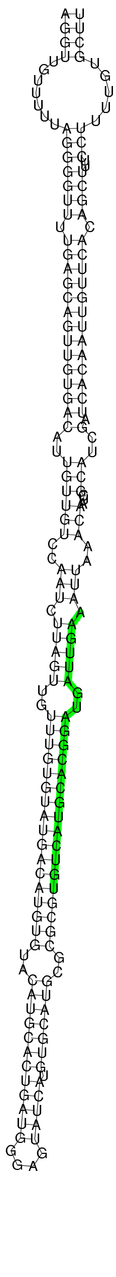

TGTCATGCACGGATGATTGAA_LG_VII_2540039-2540059

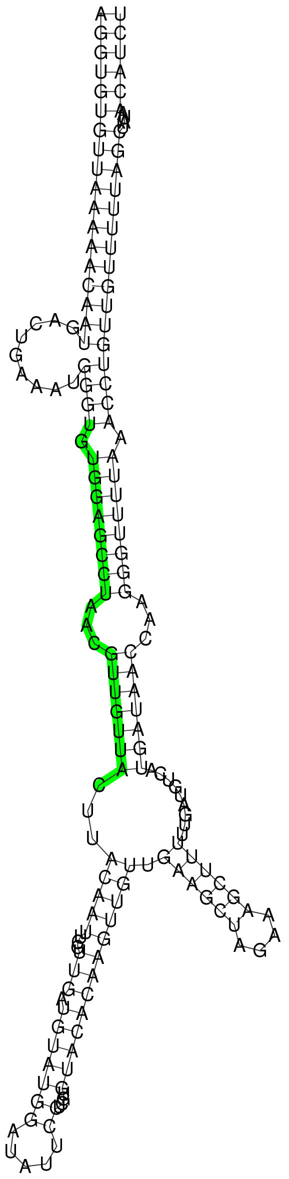

TGTGGAGCCTAACGTTGTTAC_scaffold_1064_5908-5928

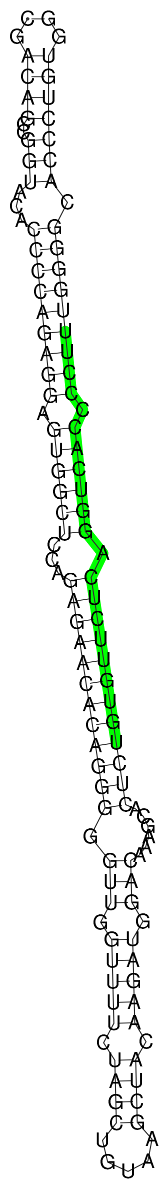

TGTGTTCTCAGGTCACCCCTT_LG_X_14819174-14819194

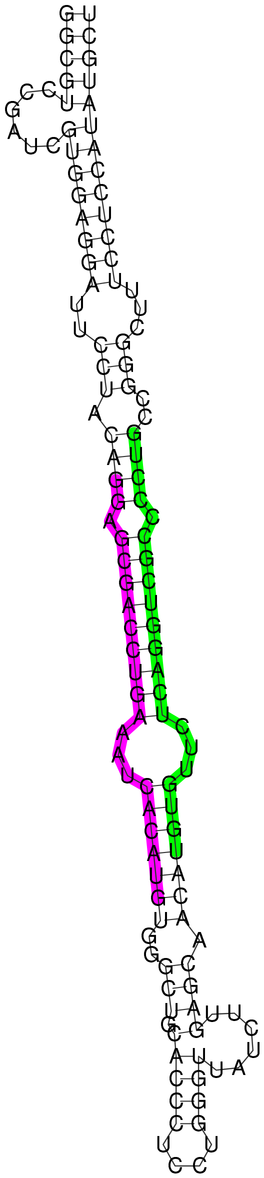

TGTGTTCTCAGGTCGCCCCTG_LG_IX_6181890-6181910

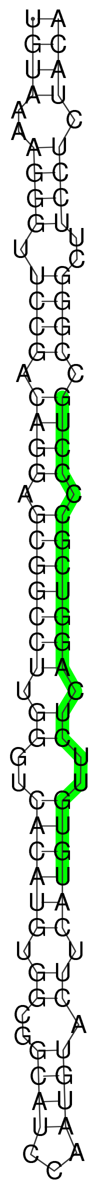

TGTGTTCTCAGGTCGCCCCTG_LG_I_19895237-19895257

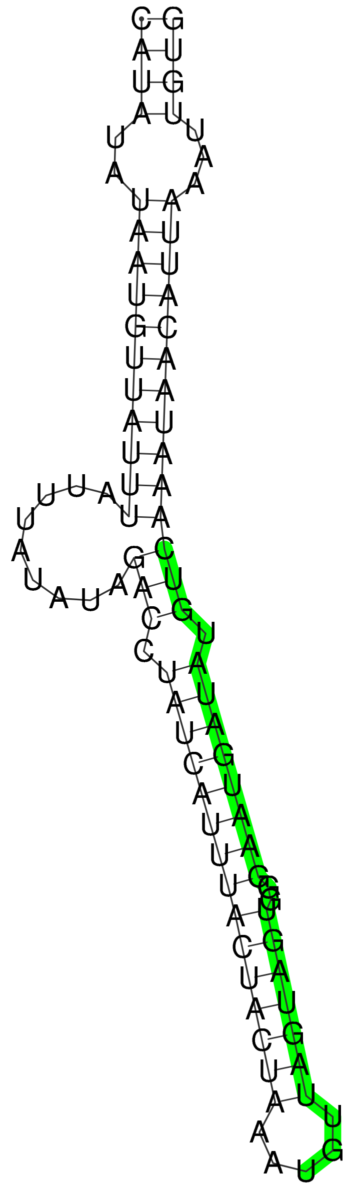

TGTTAGTAGTGGGAATGATATGTC_LG_XIX_4366411-4366434

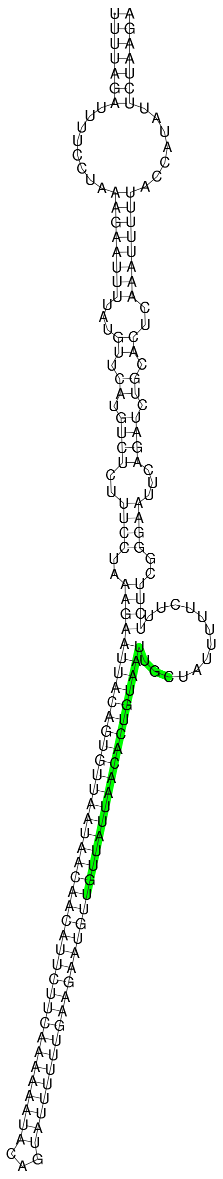

TGTTATTAACACTGTAATTGC_scaffold_66_1566196-1566216

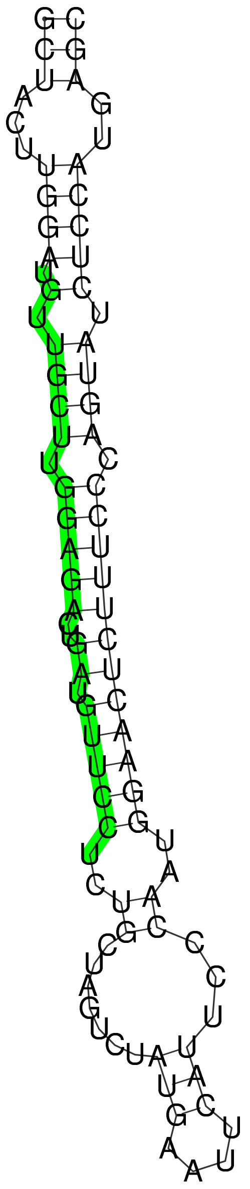

TGTTGCTTGGAGACTGATGTTCCT_LG_III_16341931-16341954

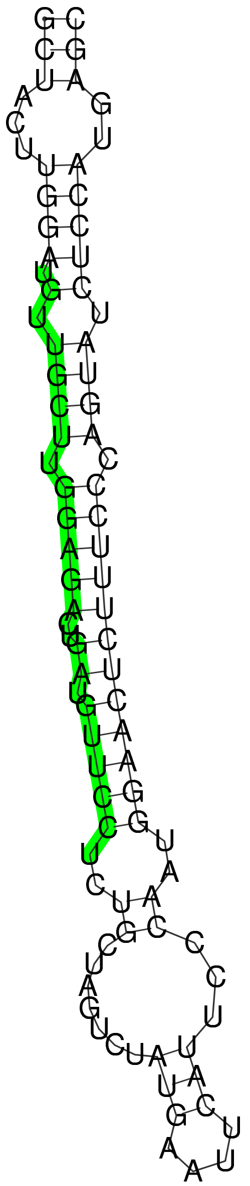

TGTTGCTTGGAGACTGATGTTCCT_scaffold_487_39929-39952

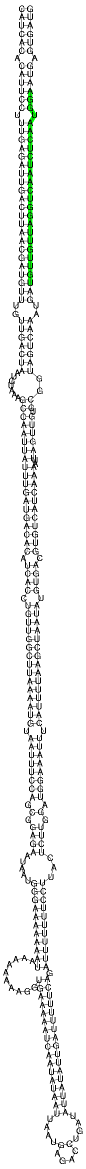

TGTTGTTAGGTCAATCTCAATGGA_LG_II_22026220-22026243

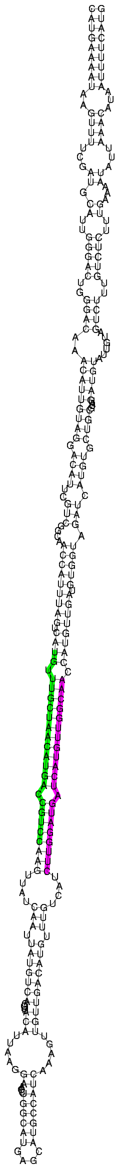

TGTTTGCTAACATGACCGTCC_LG_IV_7751070-7751090

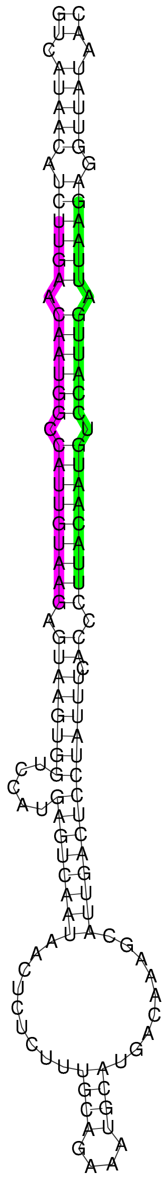

TTACAATGTCCATTGATTAAG_LG_X_12045864-12045884

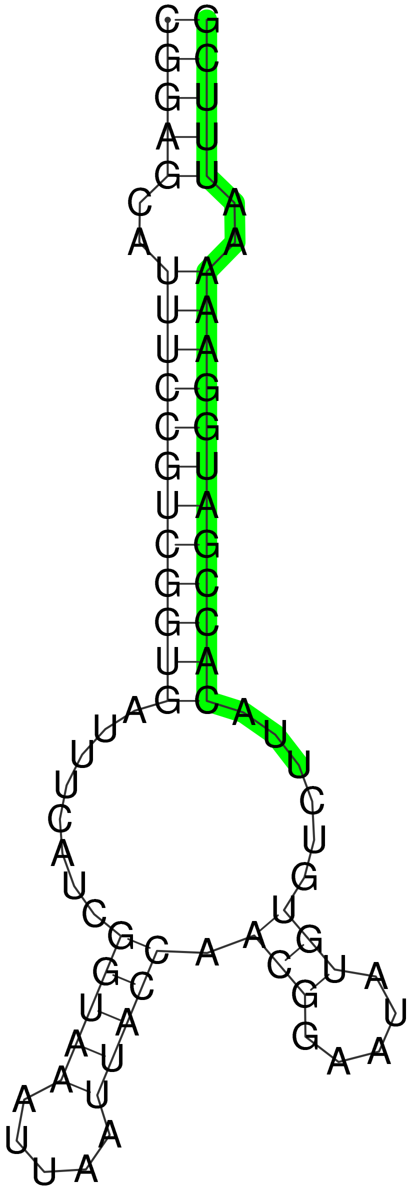

TTACACCGATGGAAAAATTTCGT_scaffold_117_629851-629873

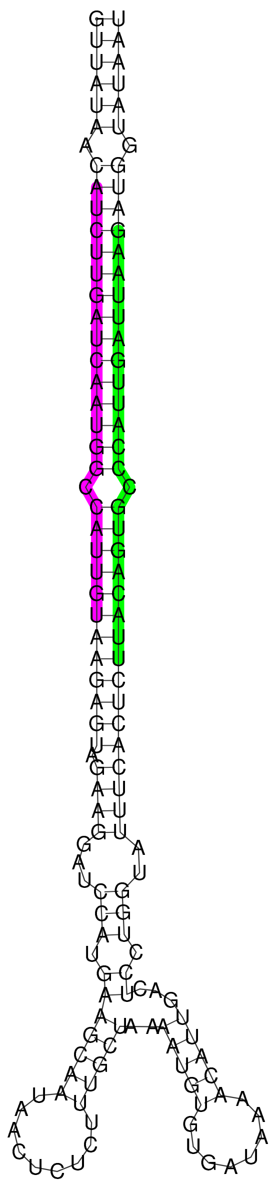

TTACAGTGCCCATTGATTAAG_LG_II_20949518-20949538

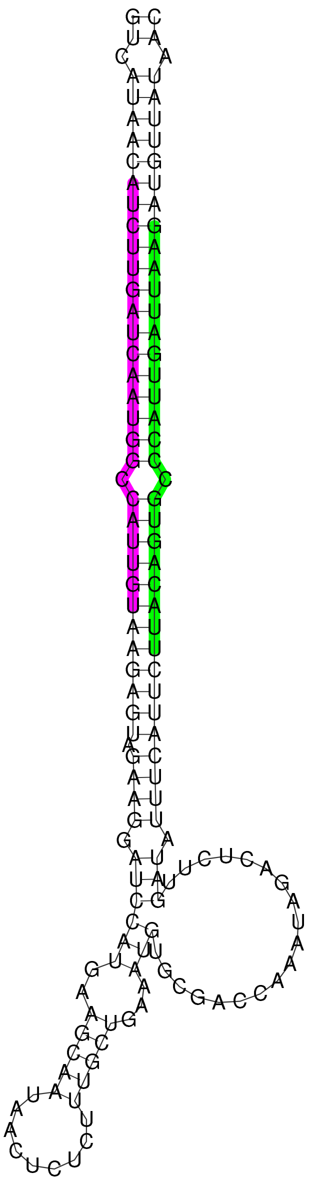

TTACAGTGCCCATTGATTAAG_LG_VIII_13883558-13883578

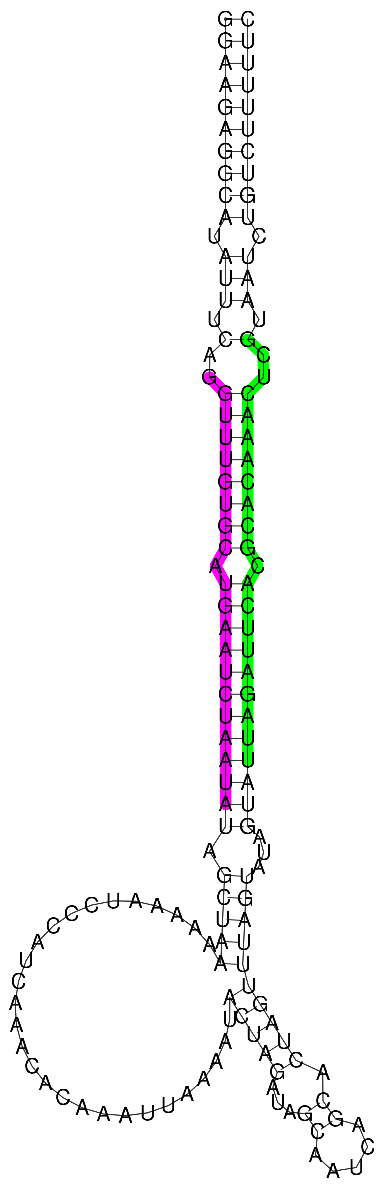

TTAGATTCACGCACAAACTCG_LG_II_16310531-16310551

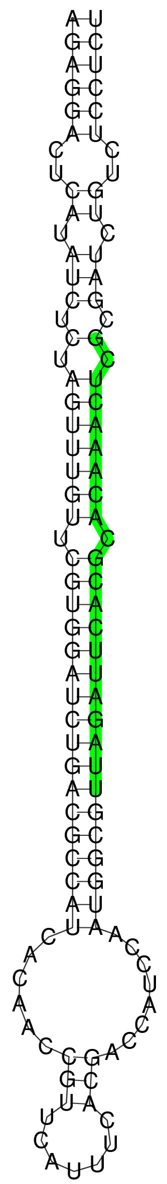

TTAGATTCACGCACAAACTCG_LG_X_13974082-13974102

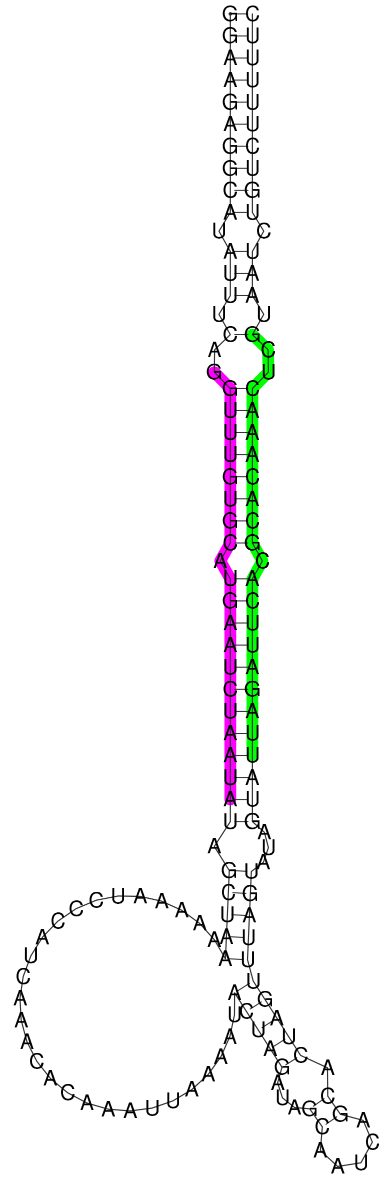

TTAGATTCACGCACAAACTCG_scaffold_3689_5219-5239

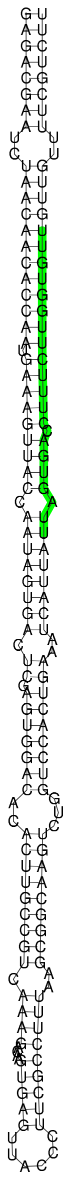

TTAGTGACCTTTCTTGGTGTT_LG_XII_11818898-11818918

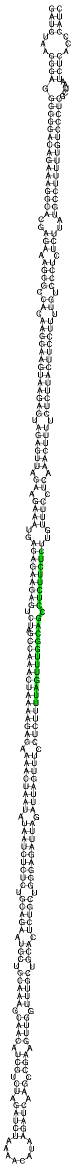

TTAGTTTGGCAGCCTCTTCTC_LG_II_1274599-1274619

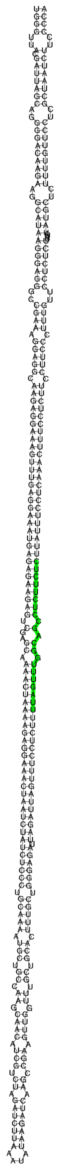

TTAGTTTGGCAGCCTCTTCTC_LG_V_16743224-16743244

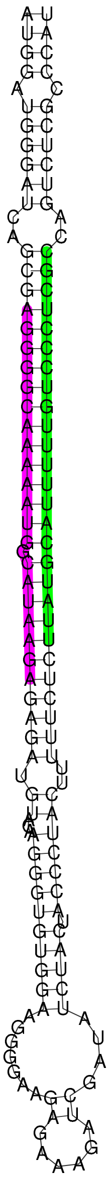

TTATGCATTTTTGTCCCTCGC_LG_V_16746861-16746881

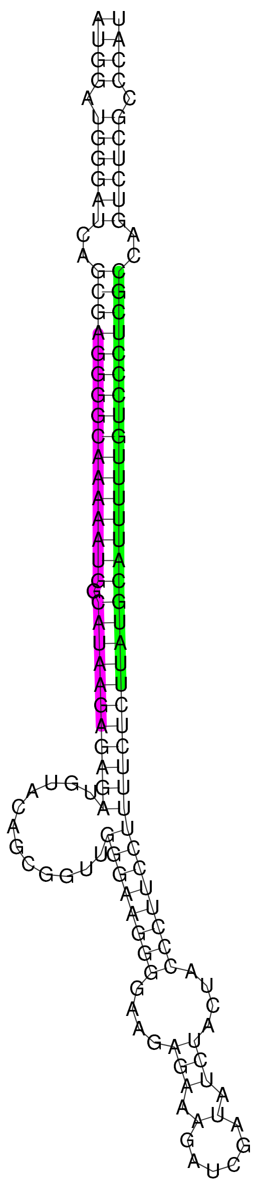

TTATGCATTTTTGTCCCTCGC_LG_V_16751017-16751037

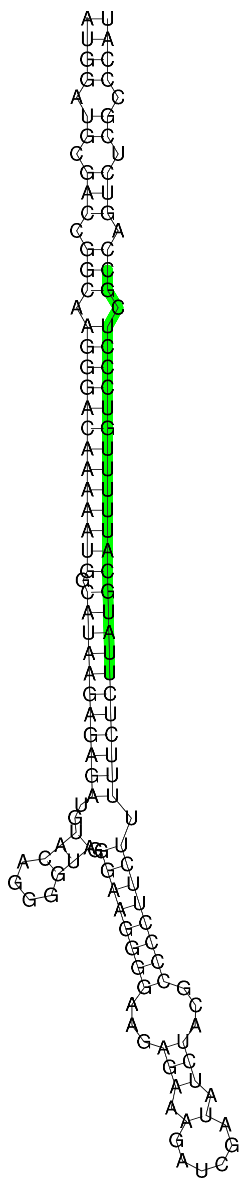

TTATGCATTTTTGTCCCTCGC_LG_V_16755196-16755216

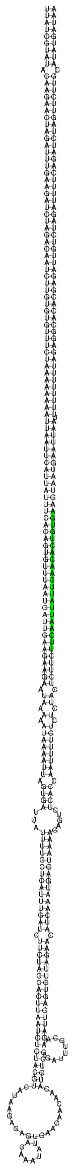

TTCAATTATTGAACACTGTGA_scaffold_44_2312570-2312590

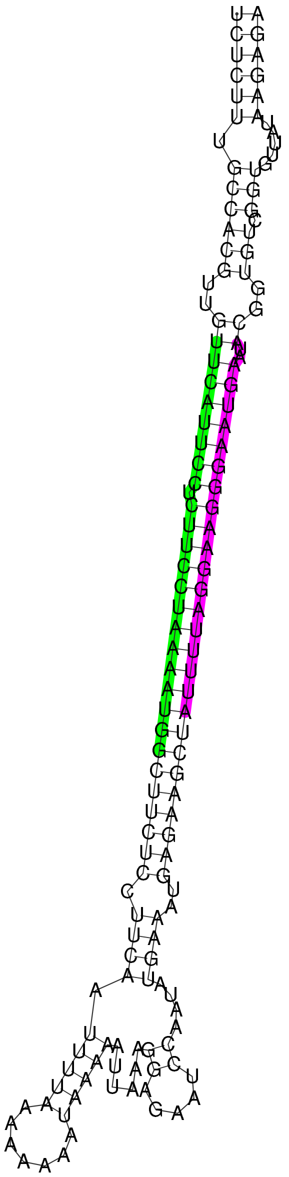

TTCATTCCTCTTCCTAAAATGG_LG_XII_8278996-8279017

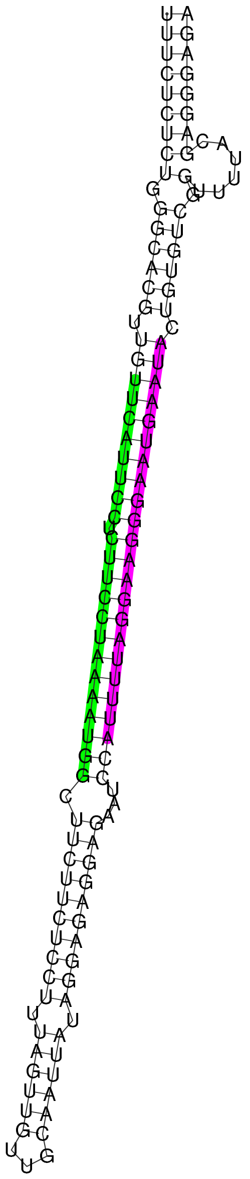

TTCATTCCTCTTCCTAAAATGG_scaffold_129_380652-380673

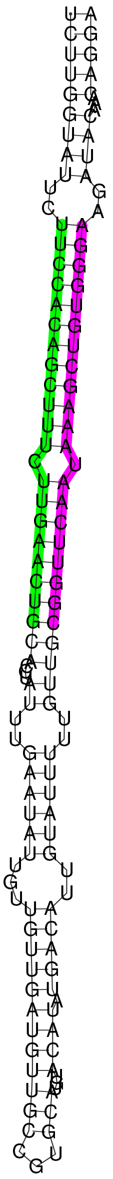

TTCCACAGCTTTCTTGAACTG_LG_VI_4075654-4075674

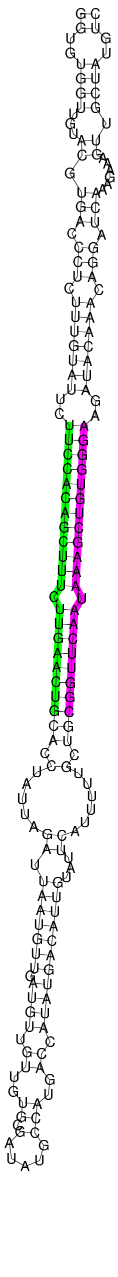

TTCCACAGCTTTCTTGAACTG_LG_XVIII_12249334-12249354

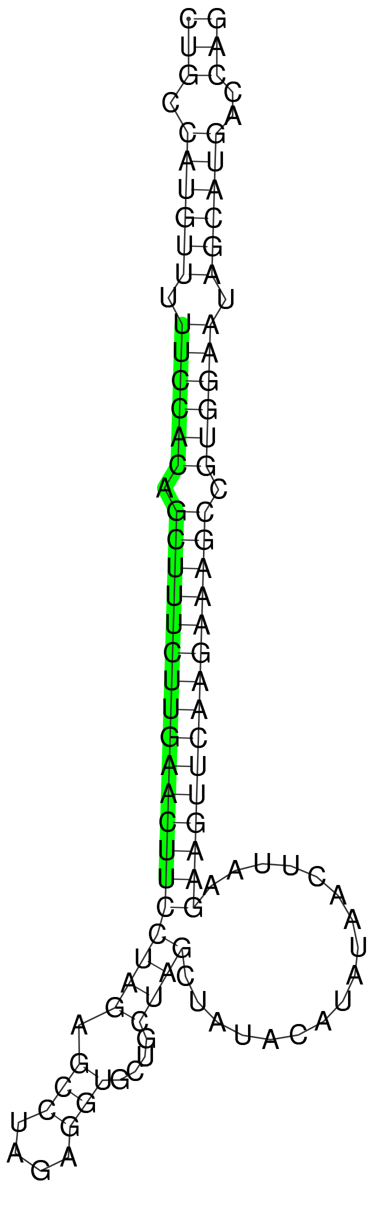

TTCCACAGCTTTCTTGAACTT_LG_III_14406868-14406888

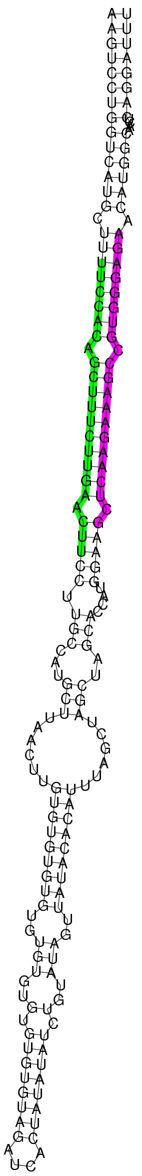

TTCCACAGCTTTCTTGAACTT_LG_VI_4082523-4082543

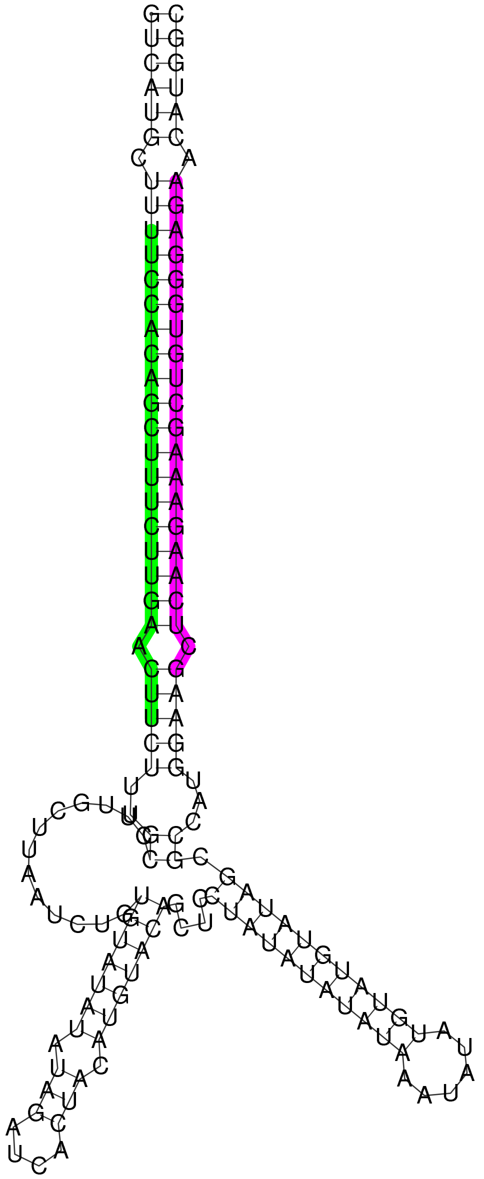

TTCCACAGCTTTCTTGAACTT_LG_XVIII_12256336-12256356

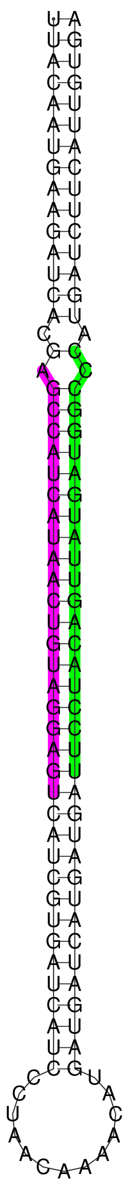

TTCCTACAGTTATGATGGCCC_LG_VI_6175922-6175942

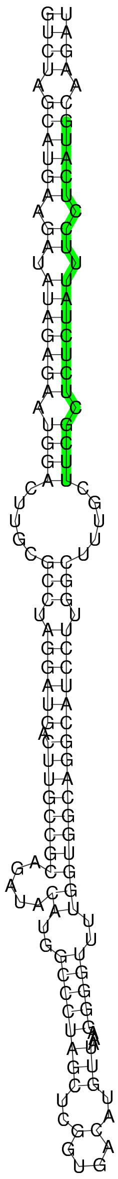

TTCGCTCTCTATTTCCTCATG_LG_VI_5024533-5024553

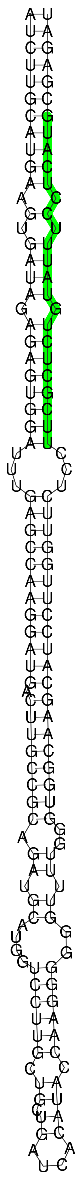

TTCGCTCTGTATTTCCTCATG_LG_XVIII_13375490-13375510

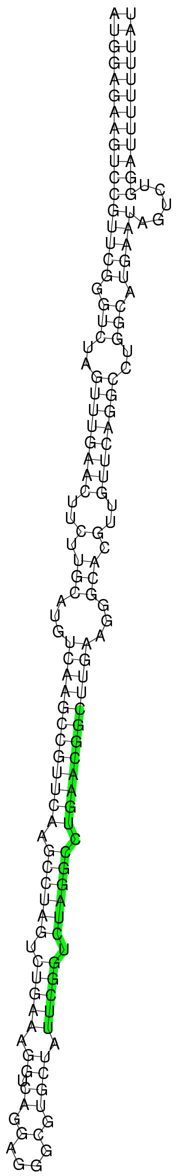

TTCGGTCTAGGCCTGAACGGC_LG_XII_881717-881737

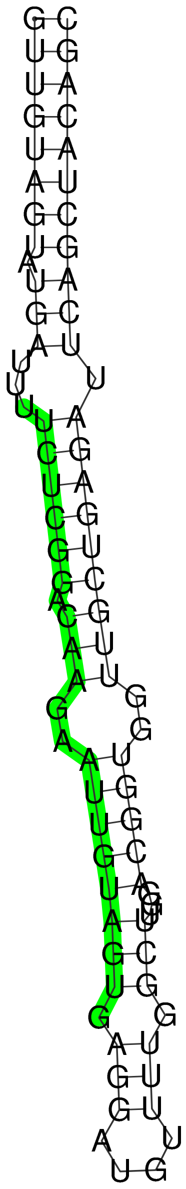

TTCTCGGACAAGAATTGTAGTG_LG_XVI_12897537-12897558

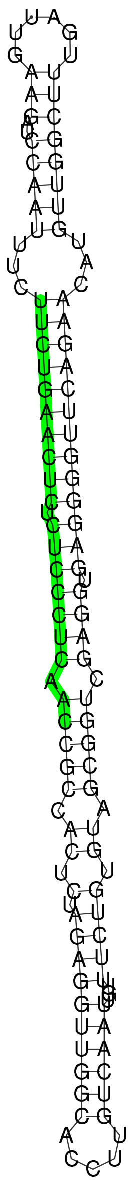

TTCTGAACTCTCTCCCTCAAC_LG_XII_12244741-12244761

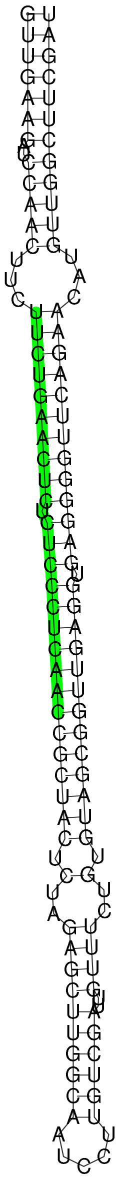

TTCTGAACTCTCTCCCTCAAC_LG_XII_12249407-12249427

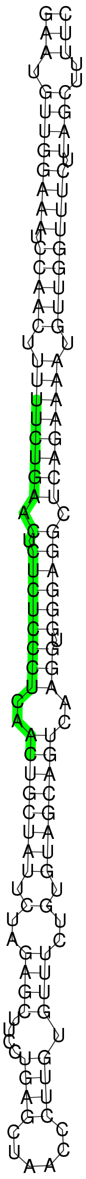

TTCTGAACTCTCTCCCTCAAC_LG_XII_12359326-12359346

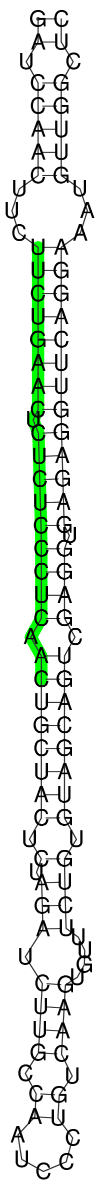

TTCTGAACTCTCTCCCTCAAC_LG_XV_7922468-7922488

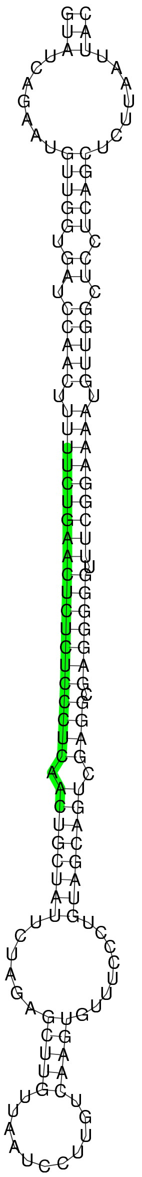

TTCTGAACTCTCTCCCTCAAC_LG_XV_8090324-8090344

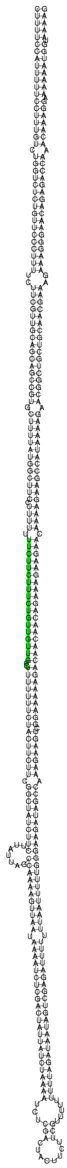

TTCTTCTTTCTGTTGTTGCCT_LG_XI_1698938-1698958

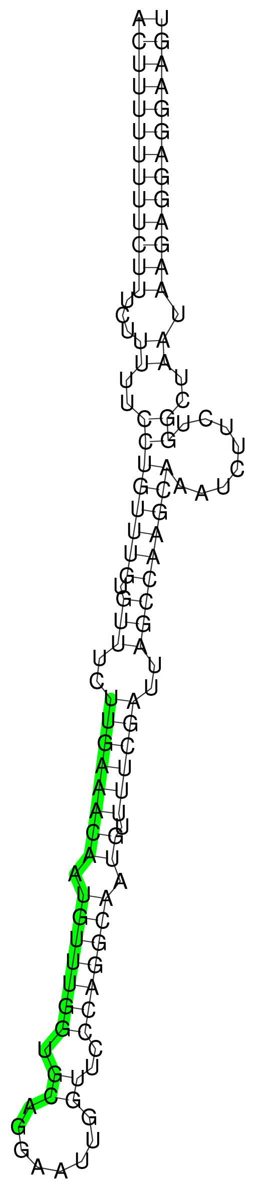

TTGAAACAATGTTTGGTGCAG_LG_VIII_10901322-10901342

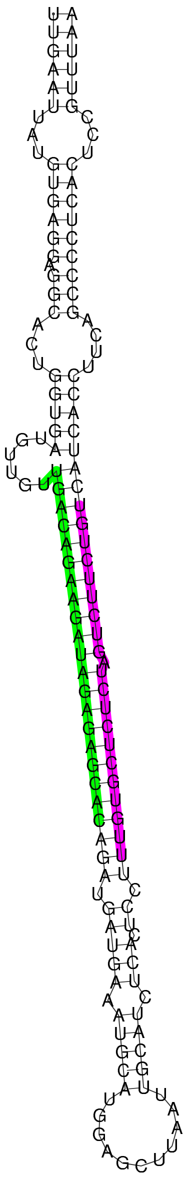

TTGACAGAAGATAGAGAGCAC_LG_II_21820407-21820427

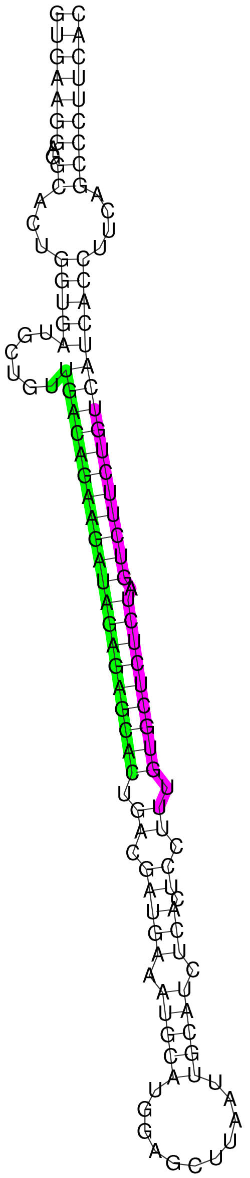

TTGACAGAAGATAGAGAGCAC_LG_IV_6006028-6006048

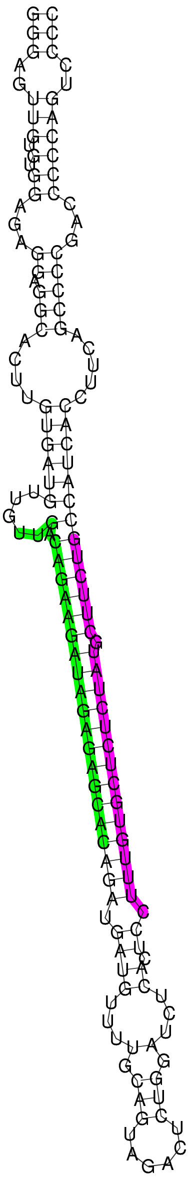

TTGACAGAAGATAGAGAGCAC_LG_XII_4450893-4450913

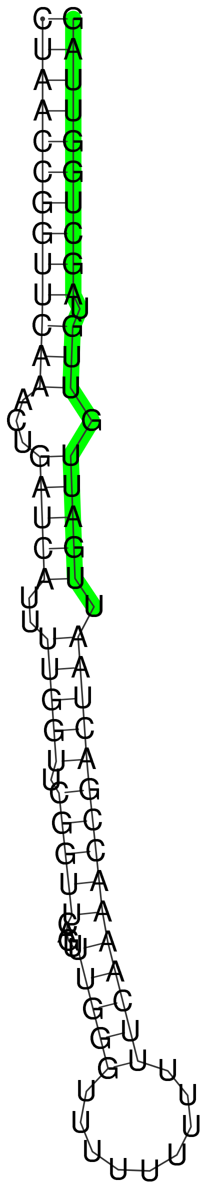

TTGATTGTTGTAGCTGGTTAGGTA_LG_IX_11887408-11887431

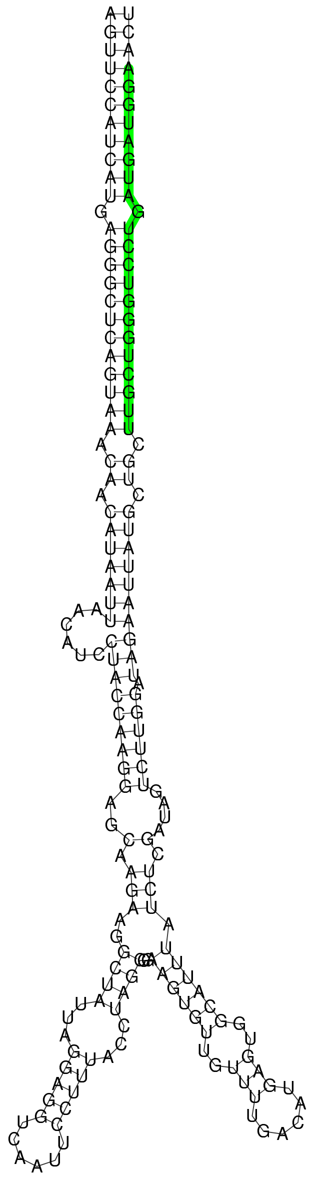

TTGCTGGGTCCTGATGATGGA_LG_XII_13376696-13376716

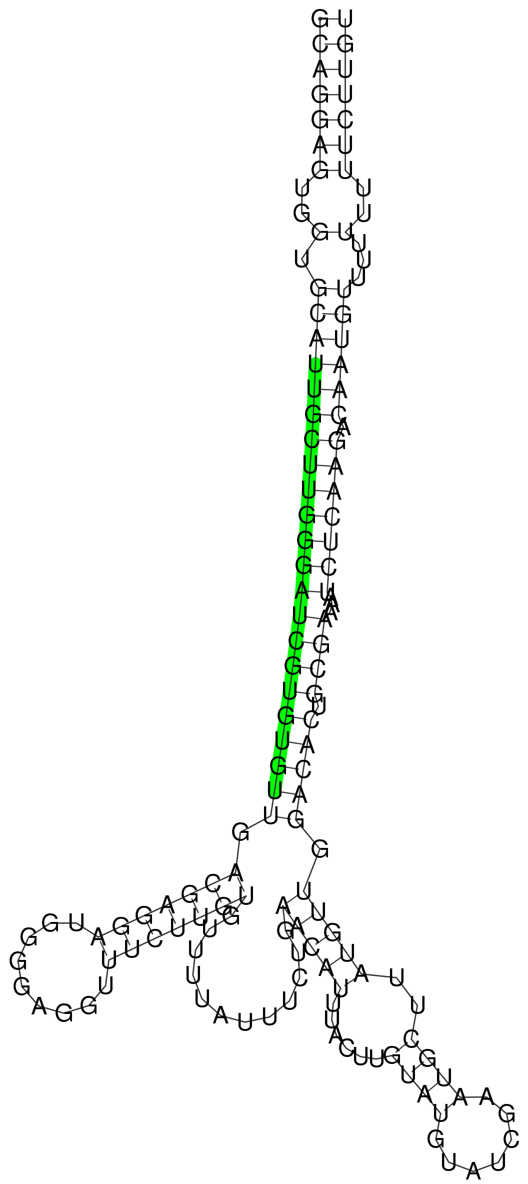

TTGCTTGGGATCGTGTGT_LG_I_5919047-5919064

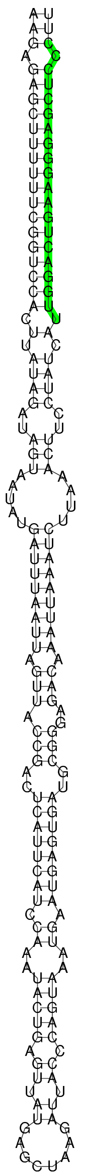

TTGGACTGAAGGGAGCTCCC_LG_III_12635218-12635237

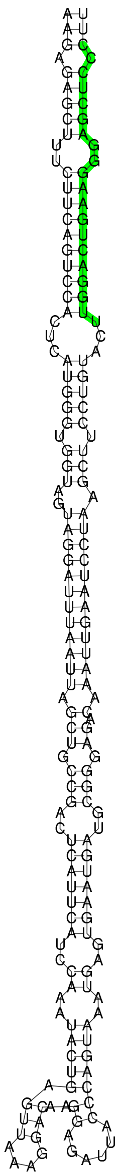

TTGGACTGAAGGGAGCTCCC_LG_I_7044536-7044555

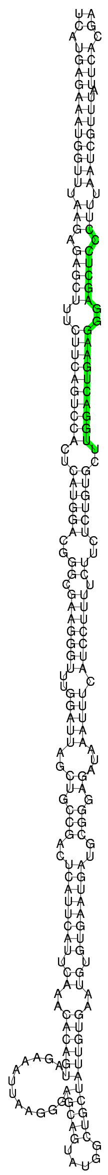

TTGGACTGAAGGGAGCTCCC_LG_XIII_12992460-12992479

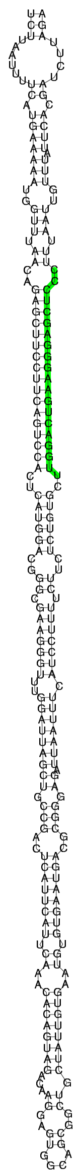

TTGGACTGAAGGGAGCTCCC_LG_XIX_10852733-10852752

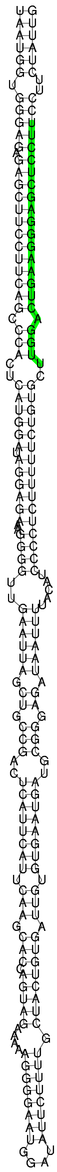

TTGGACTGAAGGGAGCTCCTT_LG_XIII_9782577-9782597

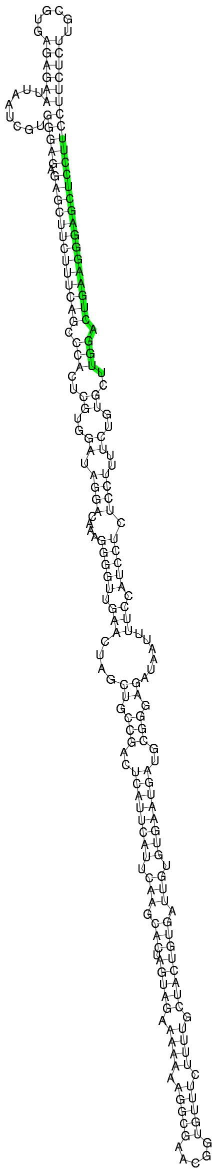

TTGGACTGAAGGGAGCTCCTT_scaffold_125_778447-778467

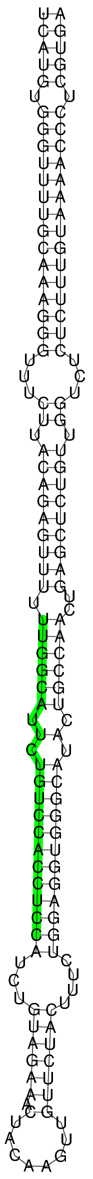

TTGGCATTCTGTCCACCTCC_LG_II_826092-826111

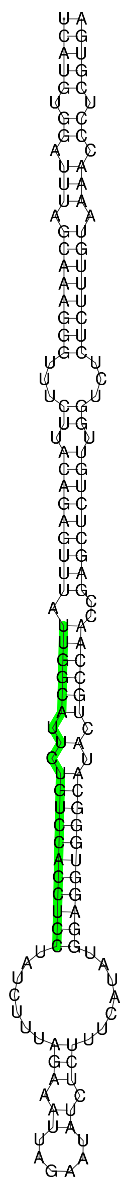

TTGGCATTCTGTCCACCTCC_LG_V_17178740-17178759

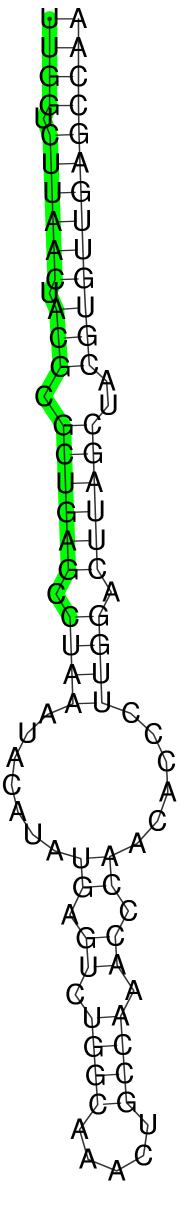

TTGGTCTTAACTACGCGCTGAGCC_LG_IV_14561800-14561823

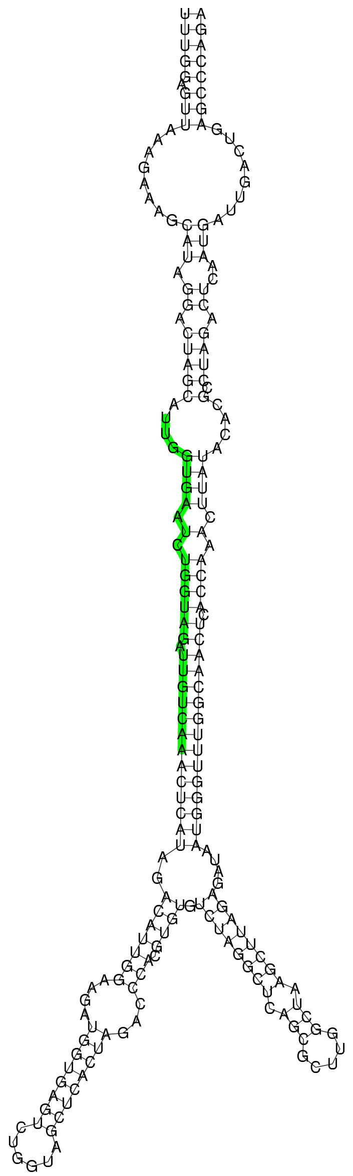

TTGGTGAATCTGGTAGATTGTCAA_LG_I_5845445-5845468

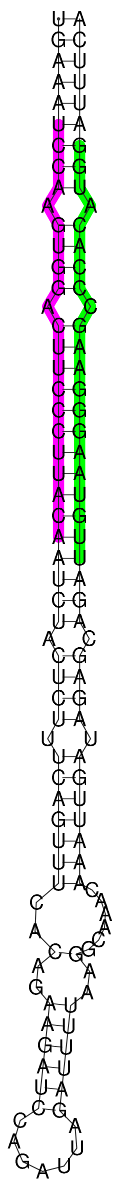

TTGTAAGGGAAGCCCACATGG_LG_I_1810419-1810439

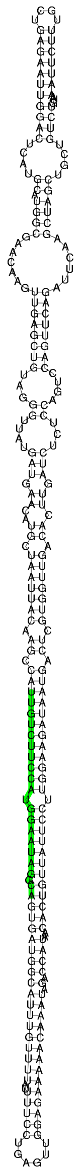

TTGTCTTCCATGGAATAGGCA_scaffold_228_146072-146092

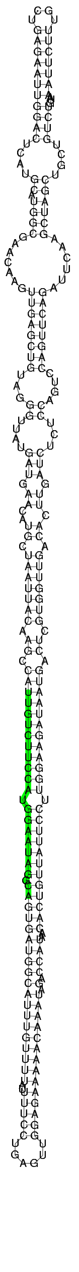

TTGTCTTCCATGGAATAGGCA_scaffold_228_153891-153911

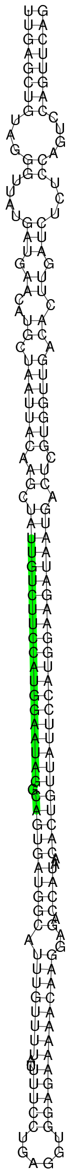

TTGTCTTCCATGGAATAGGCA_scaffold_4803_3250-3270

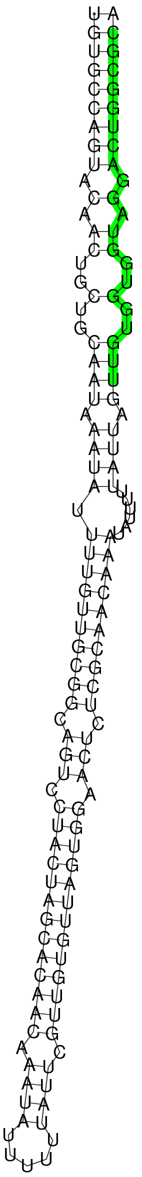

TTGTGGTGGTAGGACTGGCGC_LG_VI_4485658-4485678

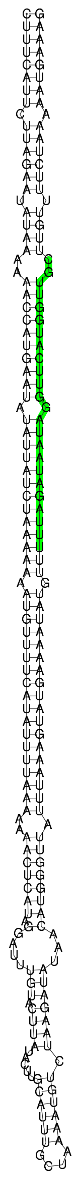

TTTAGATAATAGGTTCATGGTTGC_LG_XIX_9521426-9521449

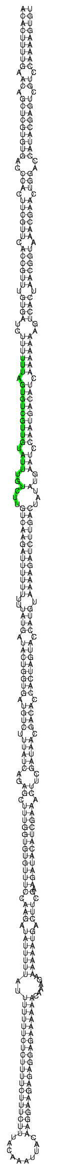

TTTAGTGTCGTTGTATTTGTCTT_LG_VII_8148067-8148089

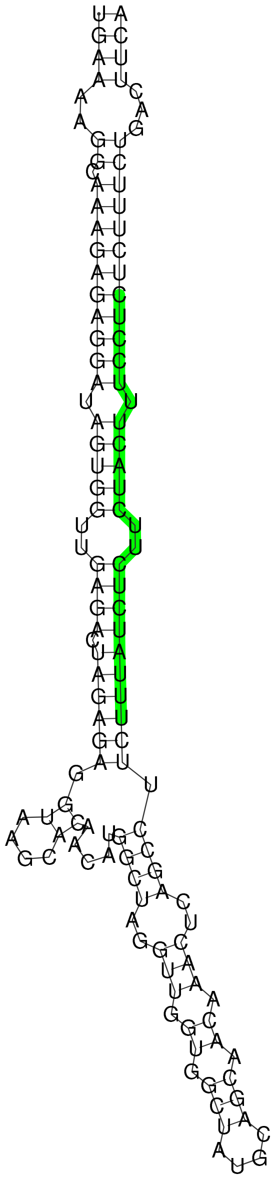

TTTATCTCTTCTACTTTCCTC_scaffold_131_591229-591249

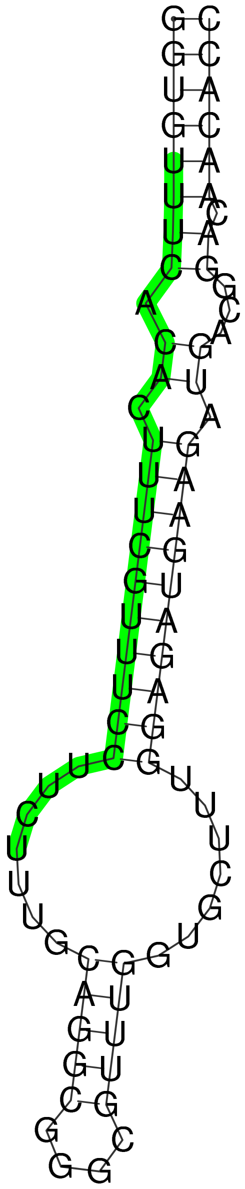

TTTCACACTTTCGTTTCCTTCT_scaffold_29_896883-896904

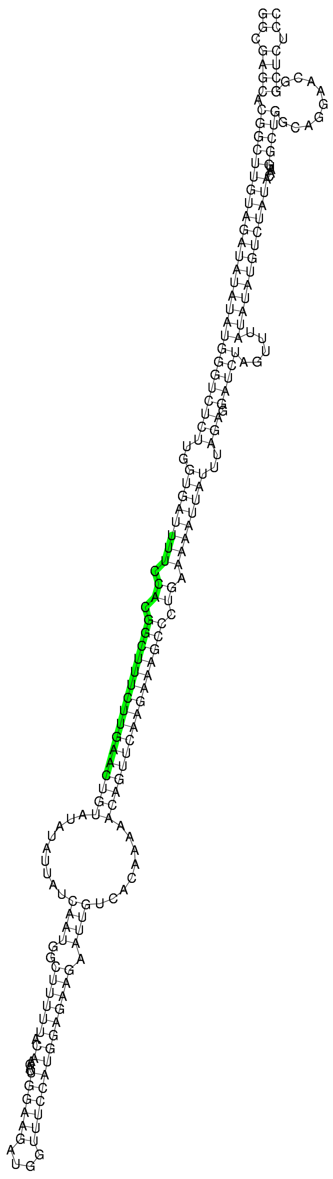

TTTCCACGGCTTTCTTGAAC_LG_VI_17347034-17347053

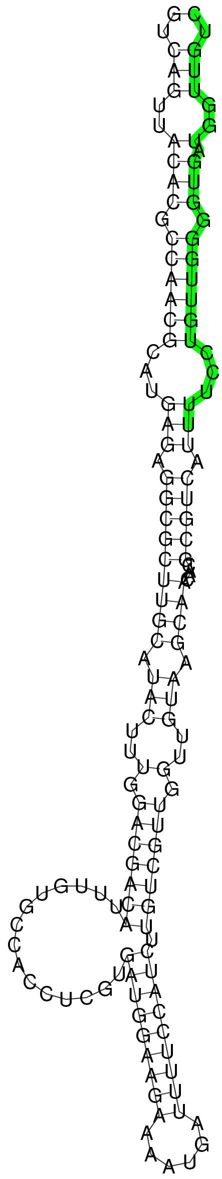

TTTCCTGTTGGGGTGATGGTTGTC_LG_XVI_10991177-10991200

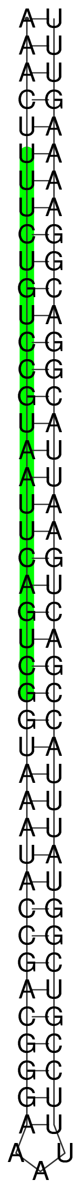

TTTCTGTCCGTAATTCAGTCG_scaffold_7779_790-810

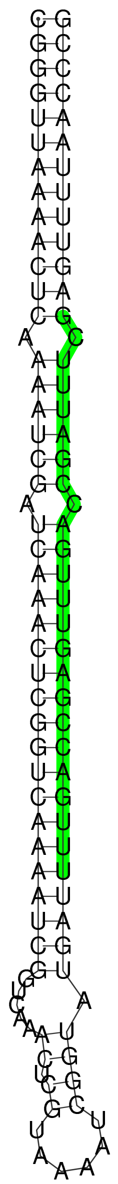

TTTGACCGAGTTTGACCGATTTCG_LG_II_21365460-21365483

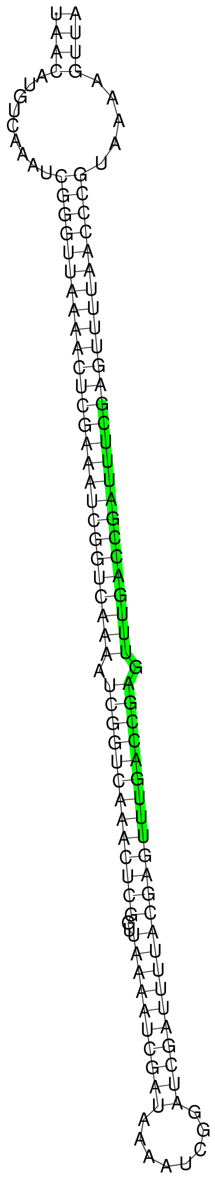

TTTGACCGAGTTTGACCGATTTCG_LG_II_22396437-22396460

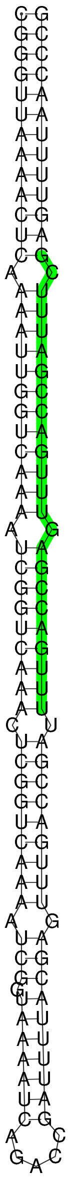

TTTGACCGAGTTTGACCGATTTCG_LG_XII_5029266-5029289

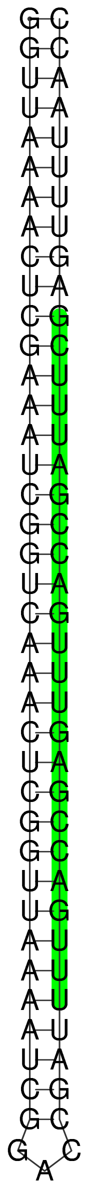

TTTGACCGAGTTTGACCGATTTCG_LG_XIV_7667336-7667359

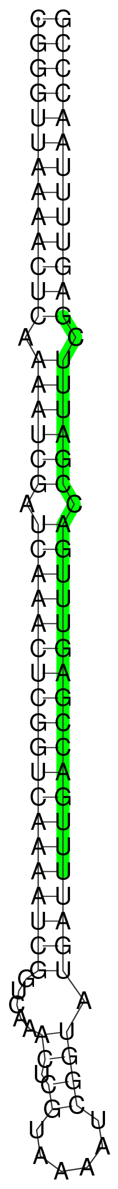

TTTGACCGAGTTTGACCGATTTCG_scaffold_960_8212-8235

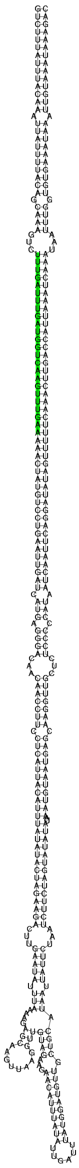

TTTGATTTGATGGTCAAGTTTGAA_LG_XIX_9510781-9510804

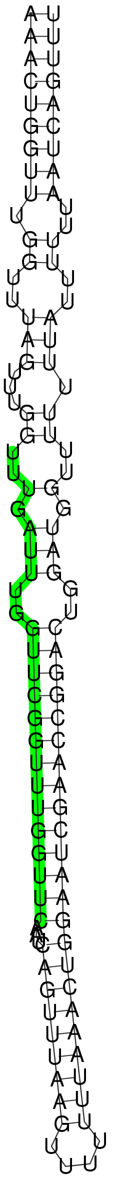

TTTGATTTGGTTCGGTTTGGTTC_scaffold_210_165156-165178

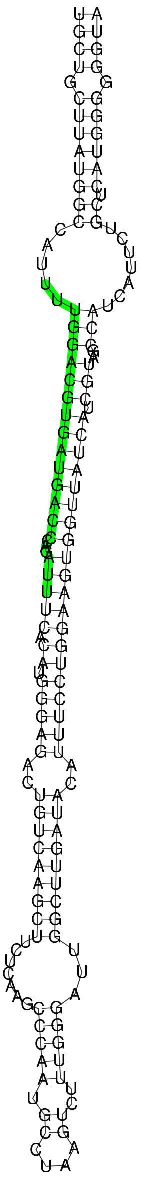

TTTGGACGTGATGACCCAGATT_scaffold_28_2405294-2405315

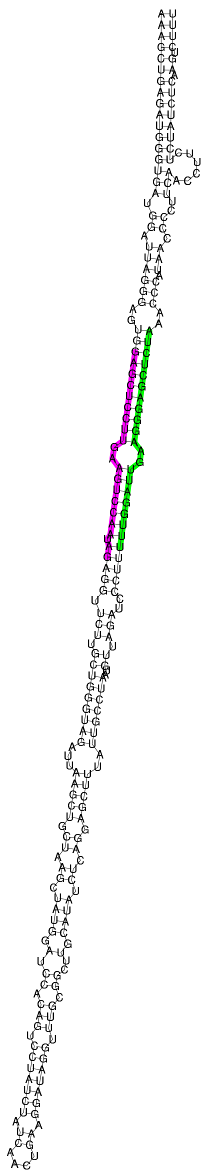

TTTGGATTGAAGGGAGCTCTA_LG_XII_4201677-4201697

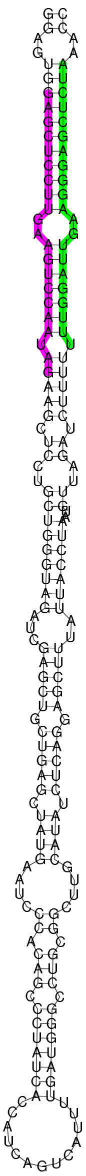

TTTGGATTGAAGGGAGCTCTA_LG_XV_3478615-3478635

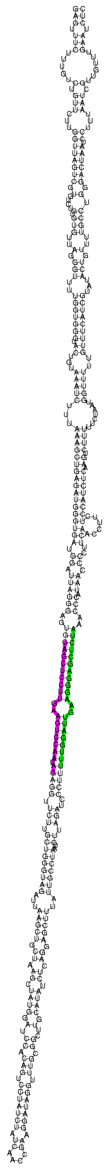

TTTGGATTGAAGGGAGCTCTA_scaffold_1140_8367-8387

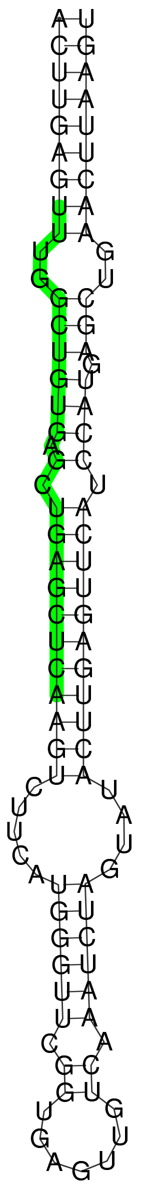

TTTGGCTGTGAGCTGAGCTCA_LG_XIV_8932167-8932187

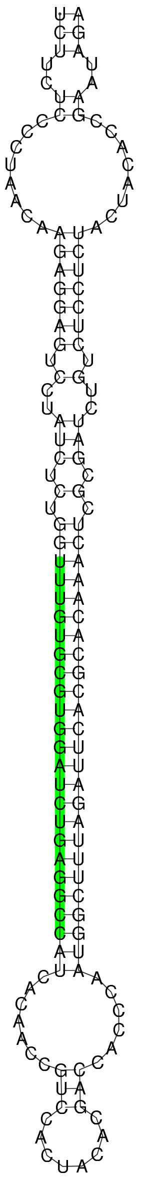

TTTGTGCGTGGATCTGAGGCC_LG_VIII_6549853-6549873

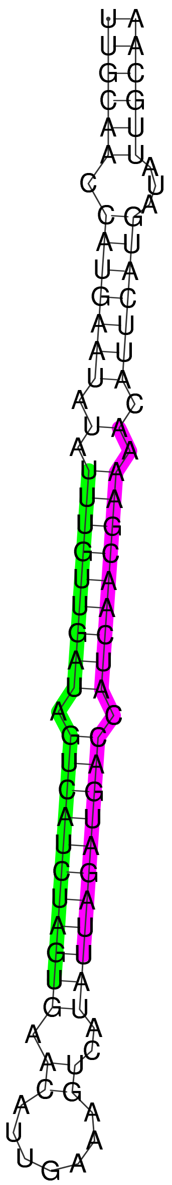

TTTGTTGATAGTCATCTAGT_LG_VII_120937-120956

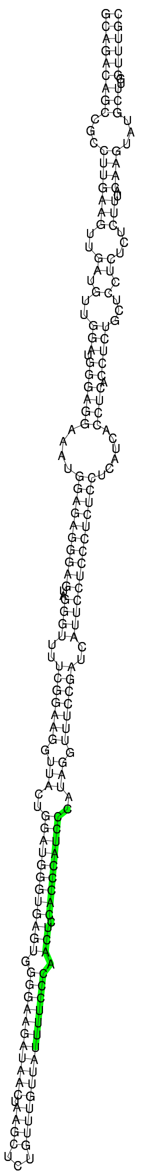

TTTTCCCAACTCCACCCATCCC_LG_XIII_203728-203749

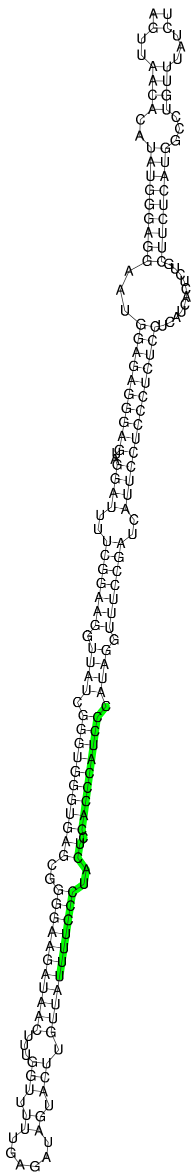

TTTTCCCTACTCCACCCATCCC_scaffold_131_138364-138385

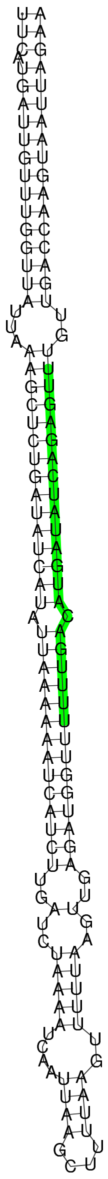

TTTTGACATGATATCAGAGTT_LG_VI_11274605-11274625
