# Supplementary material for: Genome-wide profiling of Populus small RNAs
Source: BMC Genomics. 2009 Dec 20;10:620. doi: 10.1186/1471-2164-10-620 (PMC2811130; doi:10.1186/1471-2164-10-620)
Supplement: Additional file 16 — Frequency of starting base pairs for different classes of predicted miRNAs. The frequency of predicted miRNAs starting with an A, U, C or G for miRNA loci matching existing miRBase entries, not-matching and with predicted targets and not-matching with no predicted targets. [file 1471-2164-10-620-S16.PDF]

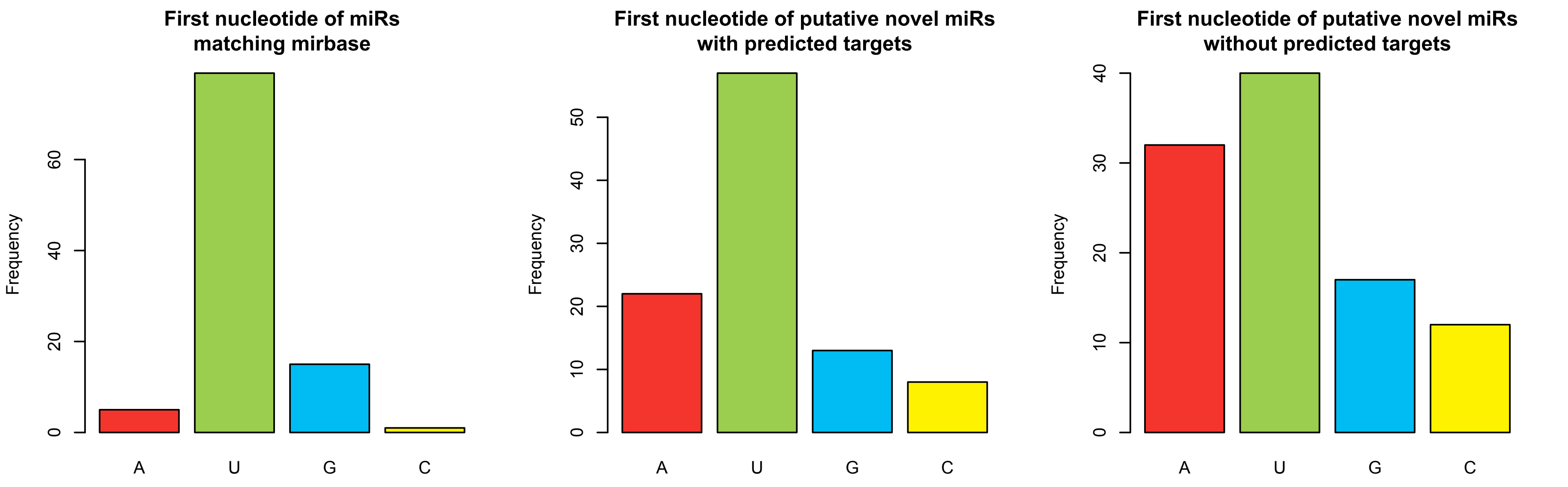
First nucleotide of miRs matching mirbase
First nucleotide of putative novel miRs with predicted targets
First nucleotide of putative novel miRs without predicted targets
Frequency
0
20
40
60
0
10
20
30
40
50
A
U
G
C
